# Structure and bonding patterns in heterometallic organometallics with linear Ln–Pd–Ln motifs†

**DOI:** 10.1039/d2sc06933d

**Published:** 2023-01-20

**Authors:** Valeriu Cemortan, Thomas Simler, Jules Moutet, Arnaud Jaoul, Carine Clavaguéra, Grégory Nocton

**Affiliations:** a LCM, CNRS, Ecole Polytechnique, Institut Polytechnique de Paris Route de Saclay Palaiseau 91120 France gregory.nocton@polytechnique.edu; b Université Paris-Saclay, CNRS, Institut de Chimie Physique UMR8000 Orsay 91405 France carine.clavaguera@universite-paris-saclay.fr

## Abstract

Complexes with short intermetallic distances between transition metal fragments and lanthanide (Ln) fragments are fascinating objects of study, owing to the ambiguity of the nature of the interaction. The addition of the divalent lanthanide fragments Cp*_2_Ln(OEt_2_) (Ln = Sm or Yb) to a Pd(ii) complex bearing the deprotonated form of the redox-active, non-symmetrical ligand, 2-pyrimidin-2-yl-1*H*-benzimidazole (Hbimpm), leads to two isostructural complexes, of the general formula (Cp*_2_Ln)_2_[μ-Pd(pyridyl)_2_] (Ln = Sm (4) and Yb (5)). These adducts have interesting features, such as unique linear Ln–Pd–Ln arrangements and short Ln–Pd distances, which deviate from the expected lanthanide contraction. A mixed computational and spectroscopic study into the formation of these adducts gathers important clues as to their formation. At the same time, thorough characterization of these complexes establishes the +3 oxidation state of all the involved Ln centers. Detailed theoretical computations demonstrate that the apparent deviation from lanthanide contraction is not due to any difference in the intermetallic interaction between the Pd and the Ln, but that the fragments are joined together by electrostatic interactions and dispersive forces. This conclusion contrasts with the findings about a third complex, Cp*_2_Yb(μ-Me)_2_PdCp* (6), formed during the reaction, which also possesses a short Yb–Pd distance. Studies at the CASSCF level of theory on this complex show several orbitals containing significant interactions between the 4f and 4d manifolds of the metals. This demonstrates the need for methodical and careful analyses in gauging the intermetallic interaction and the inadequacy of empirical metrics in describing such phenomena.

## Abstract 2

The heterotrinuclear linear arrangement Ln–Pd–Ln has short Ln–Pd distances: does it imply a covalent bond?

## Graphical abstract

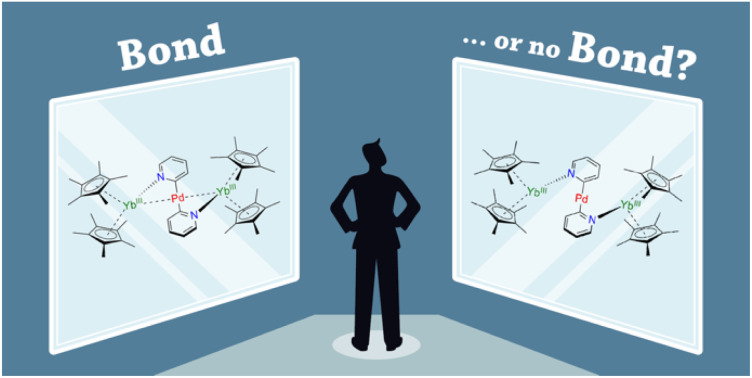

## Introduction

The f-elements are known for their large size, high number of electrons and unquenched orbital moment.^1,2^ These typical characteristics provide them with peculiar physical properties. Consequently, rare-earth-based materials are important assets for technology,^3,4^ especially those which use their magnetic and optical properties.^5–8^ Recently, the field has strongly evolved with breakthrough reports of record magnetic properties for Dy^9,10^ and Er,^11^ unusual tetravalent oxidation states for Tb and Pr,^12–14^ divalent^15–18^ and monovalent^19^ for U, and intermediate valence states in 4f-elements.^20–22^ Among these singularities, Kempe reported unsupported metal–metal bonds between 4f elements and transition metals as molecular mimics for intermetallic compounds, which constitute rare examples of lanthanide ions in direct interaction with other metals.^23^ These new examples raised an important question about the nature of the metal–metal interaction, knowing that 4f-orbitals are mostly core orbitals, meaning that the bonding in 4f-elements is principally of an electrostatic nature. Testament to this large ionic contribution remains the typical lanthanide contraction of the metal–ligand distance, which is usually observed in coordination or organometallic compounds when increasing the number of f-electrons from La to Lu.^24^ A break in this lanthanide contraction would suggest a larger orbital contribution and lead to discrepancies in the distances. The question remains if the lanthanide contraction remains applicable in metal–metal interactions.

Early studies on bimetallic complexes with lanthanide-transition metal bonds were performed by Beletskaya *et al.* and featured Lu–Ru interactions.^25^ A distance of 2.955(2) Å was reported in the solid state while IR and NMR data were in agreement with a metal–metal interaction not supported by bridging CO ligands.^25^ Kempe expanded on these results with the description of metal–metal communication in heterobimetallic complexes with Nd and Rh or Pd; the reported intermetallic distance was very similar, at 3.0345(12) Å.^26^ A series of relevant studies followed,^27,28^ leading to the report of remarkable molecules with rare-earth atoms solely bonded to Re and obtained by protonolysis of alkyl rare-earths with ReCp_2_H.^23^ The reported distances were only slightly larger than the sum of the covalent radii of both metals.^29^ In these molecules, the density analyses indicated a polar covalent bond between the metals. If, in the later examples, the bond was unsupported, a series of examples also featured adapted ligands to approach the lanthanide (Ln) and the transition metal ions. A relevant recent example was reported by Roesky and coworkers: a PN ligand was used to form a heterometallic complex with Lu and Pd with a reported short distance of 2.9031(11) Å,^30^ showing that bridging ligands can help decrease the intermetallic distance. Following this trend, Lu *et al.* reported very short contact Lu–Ni complexes with a distance as short as 2.4644(2) Å by reacting a Ni(0) precursor with an adapted NP ligand coordinated to Lu.^31^ The comparison with similar tripodal ligands clearly exemplified the large modulation of the intermetallic distance as a function of the chemical environment. An important message of this article is that this modulation is also expressed in the chemical reactivity of the compounds towards the hydrogenation of alkenes, as the CASSCF computations also showed a polarized bonding interaction between the Ni 3d_z_^2^ and the Lu 5d_z_^2^ orbitals. A very recent example by the group of Pinkowicz employed Kempe's synthetic strategy to obtain similar Er-based tetranuclear adducts, whose promising magnetic properties could open new avenues in the field of f-element SMMs.^32^

With the multiplication of short distances between lanthanide metal and transition metal fragments,^33–38^ the discussion on the intermetallic distance is often contentious. A short distance is often associated with strong metal–metal bonding, although this notion merits precision; the bridging and surrounding ligands can deeply influence the metrics without necessarily increasing the mutual interaction between the two metal fragments. This is an important note, since most of the “short-contact” metal–metal interactions are found within or below the sum of the covalent radii.^29,33,39^ As such, the use of this empirical metric as a guide for the bonding strength can be counterintuitive: reporting a short distance does not inform on the nature of the bonding, and the net definition of a lanthanide-transition metal bond can then be somewhat confusing in the literature. Based on these important remarks, Hall and co-workers recently studied a series of unsupported bimetallic complexes containing lanthanide and transition metal ions,^40^ which were designed by the group of Nippe.^41^ This study is an important addition to the discussion on the nature of metal–metal bonding and insists on the relative comparison along the series to validate the theoretical results.

In this article, we report access to a unique trinuclear linear arrangement between Pd and two lanthanide ions, Sm and Yb. Although both complexes are isostructural and despite the large differences in the reported covalent and ionic radii between Sm and Yb, up to 0.11 Å,^42^ the two complexes possess a similar metal–metal distance, which then appears as a break in the lanthanide contraction. The article describes and discusses the formation of these unique compounds, analyzes the bonding situation resulting from this singularity, and compares the situation with a dinuclear heterometallic Yb/Pd complex with a similar short distance.

## Results and discussion

### Synthesis and spectroscopic characterization

**Scheme 1:**
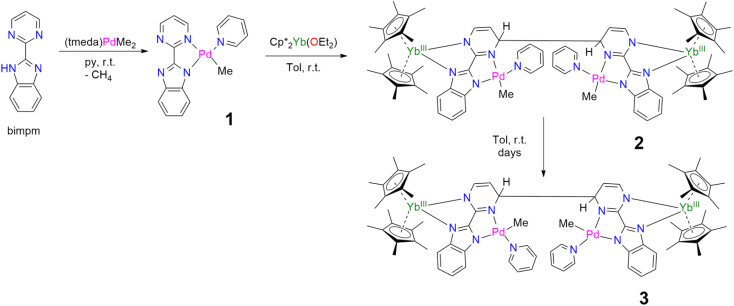
Syntheses of 1–3.

**Fig. 1:**
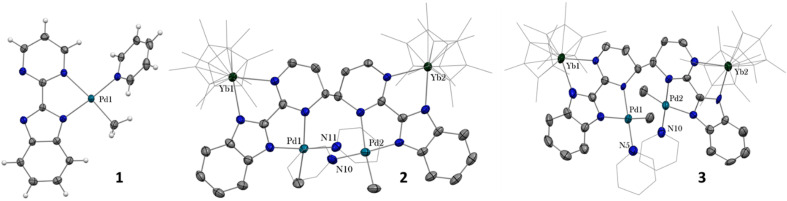
X-ray crystal structures of 1–3. ORTEPs are given with ellipsoids at a 50% probability level. For 2 and 3, the hydrogen atoms are not shown and the Cp* and pyridine ligands are depicted in a wireframe. Main distances (Å) for 1: Pd–N(pyrimidine), 2.176(2); Pd–N(imidazole), 2.035(2); Pd–N(py), 2.041(2) Å; Pd–C(Me), 2.021(3). 2: C(*ipso*)–C(*ipso*), 1.583(9); Cp*(ctr)–Ln, 2.328(12); Pd–N(imidazole), 2.033(4); Pd–N(pyrimidine), 2.16(2); mean deviation (of all atoms) from the NNPdN(py)C(Me) plane, 0.025(7). 3: C(*ipso*)–C(*ipso*) 1.556(15); Cp*(ctr)–Ln 2.336(2); Pd–N(imidazole), 2.187(7); Pd–N(pyrimidine), 2.042(4); mean deviation (of all atoms) from the NNPdN(py)C(Me) plane, 0.09(1).

In our recent series of articles, we have been interested in synthesizing heterometallic compounds by combining lanthanide and transition metals with the aim of modulating the chemical behavior of transition metal fragments.^21,43,44^ We have proposed the use of an unsymmetrical ligand, the 2-pyrimidin-2-yl-1*H*-benzimidazole (Hbimpm), as a bridge between the lanthanide and the transition metal fragment.^45^ When deprotonated, this ligand acts as an L_3_X ligand, with three neutral L-sites and one anionic X site coordinated to the lanthanide ion and to the transition metal. The palladium precursor is prepared from the protonated ligand and the known (tmeda)PdMe_2_ complex in pyridine (Scheme 1). A gas evolution indicates the deprotonation of the ligand and the subsequent formation of methane. The (bimpm)Pd(py)Me complex, 1, is obtained as golden crystals suitable for X-ray diffraction (Fig. 1). In this complex, the bimpm ligand features an LX coordination mode to the palladium ion – as shown by the different distances of 2.176(2) Å for the Pd–N(pyrimidine) and 2.035(2) Å for the Pd–N(imidazole). The departure of methane results in a vacant coordination site on the palladium +II fragment, occupied by a pyridine solvent molecule with a Pd–N distance of 2.041(2) Å. The remaining methyl is *trans* relative to the nitrogen of the pyrimidine (Pd–C(Me), 2.021(3) Å). The coordination angles around the Pd are close to 90° (see Tables S2 and S3† for more details), with a sum of coordination angles of 360.0° in support of a slightly distorted square-planar coordination geometry.

Compound 1 reacts with one equivalent of the Cp*_2_Yb fragment to immediately form a black suspension (Scheme 1). A rapid filtration of the mixture (within one minute) is necessary to obtain an analytically pure compound as a dark brown powder once the volatiles are evaporated. The dark brown powder can be dissolved in toluene to form X-ray suitable dark brown plates when the solution is cooled to −40 °C, and is analyzed as [Cp*_2_Yb(bimpm)Pd(py)(Me)]_2_, 2, in which two fragments have coupled on the pyrimidine moieties (Fig. 1). This coupling reaction was previously shown to proceed with a similar Ni species using the bimpm ligand^45^ and has been well documented with the phenanthroline ligand.^22^ This coupling is induced by a single electron transfer from the reductive divalent lanthanide species to the ligand. Strong localization of the spin density in one specific position leads to radical homo-coupling. The ^1^H NMR spectrum indicates two dominant peaks at 5.31 and 3.21 ppm for two magnetically different Cp* signals, a sharp signal at −4.46 ppm attributed to the methyl and 9 signals for the non-equivalent protons of the bimpm and pyridine ligands. We can note that 2 signals are then missing, which might be due to overlapping signals that are sometimes difficult to locate in paramagnetic species. At room temperature, the ^1^H NMR spectrum of 2 slowly evolves to a spectrum with a similar pattern, but different chemical shifts: the Cp* signals are at 5.48 and 2.49 ppm, the methyl is at −33.38 ppm and 8 signals can be detected for the coupled bimpm and pyridine ligands. Interestingly, the methyl chemical shift is significantly moved away, indicating a change in its position relative to the lanthanide paramagnetic center. The resulting species can be crystallized from the cold toluene solution and was analyzed as [Cp*_2_Yb(bimpm)Pd(Me)(py)]_2_, 3, in which the relative positions of the methyl and the pyridine have been inverted (Scheme 1). Fig. S4† shows a slow kinetic evolution of 2 to 3 in a pure diffusion regime and under mechanical stirring with approximate half-lives of 60 h and 25 h, respectively. Both compounds were found to be thermally sensitive and degraded upon heating to unknown species.

Compounds 2 and 3 have comparable solid-state structures exhibiting a Cp*_2_Yb(bimpm)Pd(Me)(py) moiety, which is coupled to another similar moiety, leading to the formation of a tetranuclear structure (Fig. 1). The coupling occurs on the pyrimidine ring in the *para* position relative to the nitrogen atom coordinated to the ytterbium metal center. The principal difference between the structures is the inversion of the position of the methyl groups relative to the nitrogen of the pyrimidine ring: in 2, the methyl is in *trans*, while it is in *cis* in 3. The *ipso* carbon is then tetragonalized and the corresponding *N*-heterocycle loses aromaticity. The coupling occurs in *exo* and the C(*ipso*)–C(*ipso*) distances are 1.583(9) Å and 1.556(15) Å in 2 and 3, respectively. The Cp*(ctr)–Ln average distance is 2.328(12) and 2.336(2) Å in 2 and 3, respectively, indicating the +III oxidation state of the ytterbium ion, which results from the electron transfer from the divalent Cp*_2_Yb fragment to the bimpm ligand, followed by the radical coupling of two ligands. The Pd–N and Pd–C coordination distances remain similar to those of 1 (see Tables S4 and S6†) and do not indicate any oxidation state modification. However, it is noteworthy that the inversion of the methyl position dramatically influences the Pd–N(imidazole) *vs.* Pd–N(pyrimidine) distances, which are also inverted: average Pd–N(imidazole) of 2.033(4) Å in 2 (similar to those in 1) and 2.187(7) Å in 3 and average Pd–N(pyrimidine) of 2.16(2) Å in 2 and 2.042(4) Å in 3, which can be traced back to the large *trans* influence of the methyl group.^46^ The mean deviation (of all atoms) from the NNPdN(py)C(Me) plane is 0.025(7) and 0.09(1) Å in 2 and 3, respectively. Compound 3 is slightly more distorted than 2, which may be due to the more pronounced steric crowding in this configuration, with possible π interactions between the two pyridines, which are almost parallel to one another and separated by less than 4 Å. The coordination geometry around the palladium remains square planar with similar angles as in 1 (Tables S5 and S7†).

**Scheme 2:**
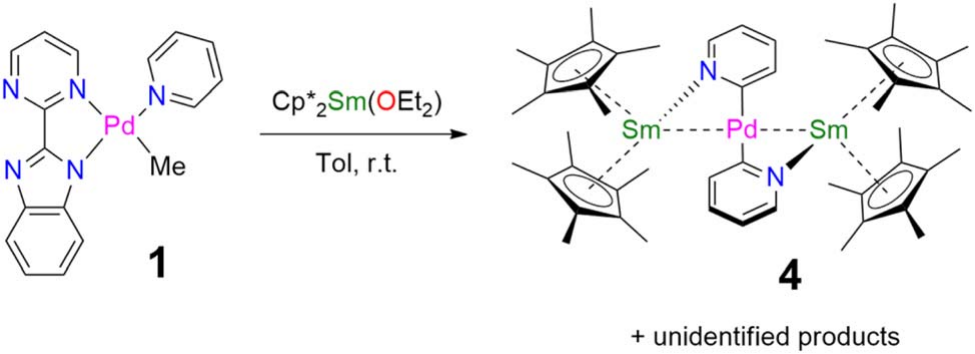
Synthesis of 4.

**Fig. 2:**
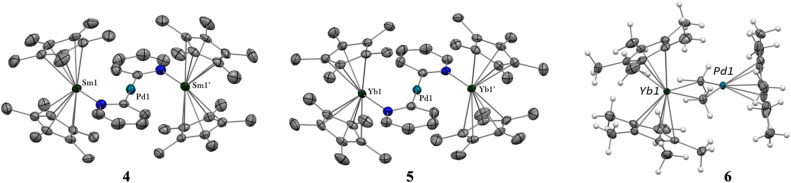
X-ray crystal structures of 4–6. ORTEPs are given with ellipsoids at a 50% probability level. Hydrogen atoms are not shown for 4 and 5. Main distances (Å) and angle (deg) for 4–6: Ln–Cp*(ctr), 2.45 Å (4), 2.34 Å (5), and 2.33 Å (6); Ln-*N*(pyridyl), 2.386(4) (4) and 2.275(3) (5); Pd–C(pyridyl), 2.080(5) (4) and 2.079(4) (5); C(Me)–Yb 2.559(2) and 2.552(2) (6); Ln–Pd, 2.9078(3) (4), 2.9244(2) (5), 2.8766(3) (6); Ln–Pd–Ln 175.61(2) (4) and 177.25(2) (5).

When the reaction of one equivalent of 1 with the more reducing samarocene Cp*_2_Sm was performed, the corresponding coupling products were not observed. Similarly to the addition of ytterbocene, a gaseous evolution was observed while the solution turned black. However, the ^1^H NMR spectrum was poorly informative. Concentrating and cooling the solution to −40 °C led to brown crystals in very low yield, which were X-ray suitable and analyzed as a unique trinuclear species with a remarkable linear Sm–Pd–Sm pattern with two Cp* ligands on each lanthanide ion and two pyridyl C_5_H_4_N ligands on the Pd ion and identified as (Cp*_2_Sm)_2_[μ-Pd(pyridyl)_2_], 4 (see Scheme 2 and Fig. 2). Since the stoichiometry was given as one transition metal for two lanthanide ions, the reactions were repeated with 2 equivalents of samarocene and resulted in a relatively higher yield (10%). An insoluble precipitate was obtained as a by-product of this reaction, which we tentatively attribute to Pd^0^ species.

The difference in reactivity observed between the Yb and Sm divalent lanthanide fragments posed the question of the possibility of forming a similar compound with ytterbium. Two reactions were attempted: the addition of 2 or 3 to one more equivalent of Cp*_2_Yb(OEt_2_) or a direct reaction between Cp*_2_Yb(OEt_2_) and 1, but with an extended reaction time (as opposed to the fast formation of 2). Both of these reactions led to a gaseous evolution, indicative of methane formation, and yielded an ytterbium complex isostructural to 4. X-ray-suitable brown crystals were formed by crystallization in cold toluene in both cases and were analyzed as (Cp*_2_Yb)_2_[μ-Pd(pyridyl)_2_], 5. Performing the reaction of 2 with an excess of Cp*_2_Yb(OEt_2_) at different temperatures or solvents did not affect the formation of 5.

The X-ray crystal structures of 4 and 5 are isostructural in *C*2/*c* with two Cp*_2_Ln (Sm = Yb and Sm) fragments bridged by a linear Pd(pyridyl)_2_ fragment (Fig. 2). The anionic pyridyl ligand results from a C–H activation of the pyridine ligand and concomitant departure of methane. One pyridyl nitrogen is coordinated to one lanthanide atom and the other nitrogen to the second lanthanide. Overall, the point symmetry is *D*_2h_. The Ln–Cp*(ctr) distance is 2.45 Å in 4 and 2.34 Å in 5, in agreement with the lanthanide contraction and the 0.11 Å covalent radius difference between the early Sm and the late Yb.^42^ The distance is consistent with the corresponding distances of 2.45 Å in trivalent Cp*_2_SmI(thf)^47^ and 2.31 Å in [Cp*_2_Yb(phen)I].^22^ The Ln-*N*(pyridyl) distances also agree with the lanthanide contraction and are 2.386(4) Å for 4 and 2.275(3) Å for 5. The Pd–C(pyridyl) distances are 2.080(5) Å and 2.079(4) Å in 4 and 5, respectively. These distances are longer than those in the relevant reported Pd^II^-pyridyl fragments found in the literature that are in the 1.95–2.01 Å range.^48–50^ The Ln–Pd–Ln angles are 175.61(2) (4) and 177.25(2) (5), confirming the linearity of the Ln–Pd–Ln pattern. The most striking feature of these structures lies in the Ln–Pd distances, which are short in both cases: 2.9078(3) Å and 2.9244(2) Å for 4 and 5, respectively. The distances are within the sum of the covalent radii of Ln and Pd.^39^ Most reports of Ln–Pd interactions in the literature are larger than 3 Å,^33^ with two notable exceptions: the Pd–Lu and Pd–Y series of compounds published by Roesky and co-workers,^30^ in which the reported metal–metal distances are 2.9031(11) Å, 2.9712(8) Å and 2.9898(6) Å and the series of Ln–Pd phosphinoamido complexes with Ln–Pd (Ln = Sc, Y, Sm, Yb, Gd, Lu) distances ranging from 2.432(2) to 2.862(3) Å that have been reported by Cui and co-workers.^51–53^ It is worth noting that a shorter Pd–U distance of 2.69 Å was reported, in which a covalent interaction was suggested.^54^ A feature that merits attention is that the Ln–Pd distances in 4 and 5 differ by only 0.017 Å, significantly smaller than the 0.11 Å difference of the covalence radii between both lanthanide ions. This highlights an apparent break in the lanthanide contraction. This rare occurrence raises the question of a possible covalent interaction between the metals to explain this singularity.

The temperature-dependent magnetic data for 4 and 5 are shown in Fig. S28 and S29.† The *χT vs. T* plot of 4 evolves linearly from 0.113 to 0.969 cm^3^ K mol^−1^ in the 5 to 300 K range, indicating a Van-Vleck type of paramagnetism (temperature independent paramagnetism) typical of the +III oxidation state of samarium.^55^ Below 5 K, the *χT* value drops to 0.06 cm^3^ K mol^−1^, which results from possible dipolar antiferromagnetic coupling. The *χT vs. T* value of 5 at 300 K is 5.36 cm^3^ K mol^−1^, which means 2.68 cm^3^ K mol^−1^ per ytterbium center, and is slightly higher than the corresponding theoretical value of 2.54 cm^3^ K mol^−1^, in good agreement with the +III oxidation state of ytterbium.

Thus, from the X-ray and the magnetic data, the lanthanide centers are in the +III oxidation state, and the Pd center is in the zero-oxidation state. The bridging Pd fragment is thus a unique example of a linear d^10^ bis-pyridyl Pd^0^ fragment. Although a few complexes based on group 11 centers with bis-*p*-tetrafluoropyridyl architectures have been isolated,^56,57^ to the best of our knowledge, the only structurally authenticated unsubstituted bis-pyridyl motifs are in mercury-supported adducts, where the pyridyls are coordinated in the *para* position.^58,59^

The ^1^H NMR spectra of 4 and 5 were recorded in THF-d_8_, showing five main signals at 4.87, 4.64, 1.11, −0.81, and −9.06 ppm in a 2 : 2 : 60 : 2 : 2 ratio for 4 and 228.3, 116.8, 25.78, 24.89, and 9.02 in a 2 : 2 : 2 : 2 : 60 ratio for 5. The identity of the pyridyl signals was verified by synthesizing the deuterated C_5_D_5_N analog of 1 and then engaging it in a reaction with 2 equivalents of Cp*_2_Sm(OEt_2_) to form the deuterated analog of 4 (the resulting ^1^H NMR spectrum is shown in Fig. S13†).

**Scheme 3:**
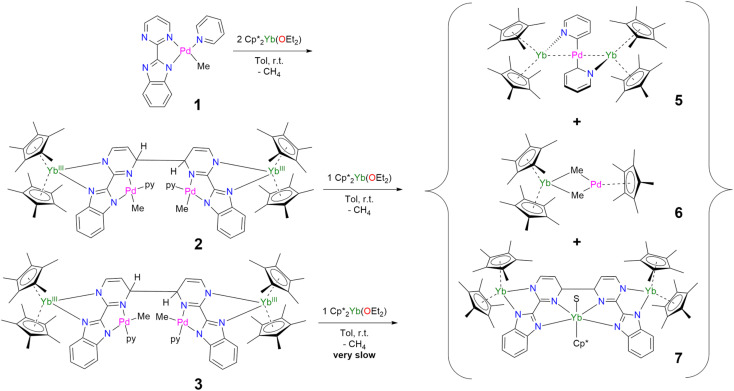
Syntheses and isolation of 5–7.

The formation of 4 and 5 was tracked *in situ* and it is rather clear that they correspond to the major products (Fig. S17, S18 and S23–S26†). Furthermore, other significant signals could also be monitored in the case of 5. Two side-products issued from the formation of 5 (but none from 4) have been isolated by modifying the treatment of the reaction (Scheme 3). The supernatant resulting after multiple crystallizations of 5 contained two main products, which could be isolated cleanly after washing with cold pentane. The ensuing brown powder was extracted with a minimum amount of diethyl ether at room temperature and redissolved in toluene, resulting in a brown solution, which, upon cooling, yielded X-ray suitable crystals of Cp*_2_Yb(μ-Me)_2_PdCp*, 6. In this complex, the bimpm ligand is removed and two methyl groups bridge the ytterbium and palladium ions. The average Cp*(ctr)–Yb distance is 2.33 Å, in agreement with that in a trivalent ytterbium fragment. The C(Me)–Yb distance is 2.559(2) Å and is similar to previously reported values in the literature.^60^ The Pd is in the +II oxidation state with one Cp* ring coordinated and an intermetallic Pd–Yb distance of 2.8766(3) Å is reported. The distance can be compared with the short reported Pd–Yb distances of 2.7879(6) Å^51,53^ and 2.8397(4) Å.^52^

The remaining brown powder can be crystallized from cold toluene and corresponds to a coupling product, already published by our group,^45^ in which the palladium fragments have been removed and replaced by a Cp*Yb(S) (S = THF or Et_2_O) fragment, and is given as Cp*Yb(S)[Cp*_2_Yb(bimpm)]_2_, 7 (Scheme 3).

Thus, the reaction of 1 with divalent Cp*_2_Ln (Ln = Yb, Sm) fragments leads to the formation of multiple products, five of which are identified. When the reaction of two equivalents of Cp*_2_Yb(OEt_2_) and 2 (containing 10% of 3) was monitored by ^1^H NMR spectroscopy (Fig. S6†), the relative ratios of the products after 10 h are the following: 0% of 2, 42% of 5, 25% of 7, 13% of 6, 7% of 3 and 10% of unreacted Cp*_2_Yb(OEt_2_) (Fig. S7 and S8†). Overall, the conversion is high in ytterbium and the reaction forms multiple products from ligand rearrangements. Performing the addition of Cp*_2_Yb(OEt_2_) to 3, on the other hand, results in significantly longer reaction times (the reaction is incomplete after two weeks) and very different relative ratios of the final complexes 5–7 (Fig. S9 and S10†). The reduction of 2 and 3 with other non-coordinating reductants (CoCp*_2,_ sodium naphthalene) did not lead to the formation of 4 and 5, but to unidentified products, demonstrating the importance of the divalent lanthanide fragment in the occurrence of this reduction reactivity.

### Theoretical studies

**Fig. 3:**
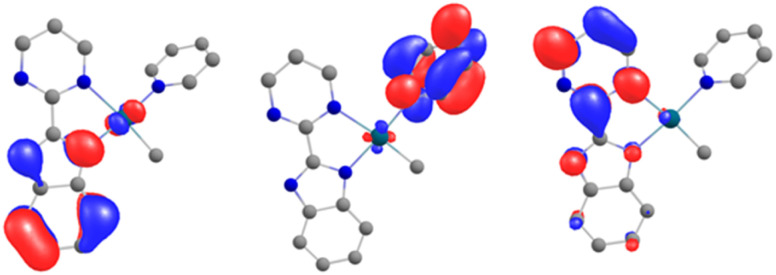
The Kohn–Sham frontier orbitals of 1: HOMO (left), LUMO (center), and LUMO+1 (right).

In order to explain the formation of 4 and 5, we turned to theoretical computations. The electronic structure of 1 was investigated at the DFT level. The Kohn–Sham frontiers orbitals are shown in Fig. 3. The HOMO is localized on the imidazolium site, in agreement with the anionic X nature of the ligand. The energy difference between the LUMO and LUMO+1 (Fig. 3) is 0.44 eV in PBE0-D3. The implication is particularly interesting – either of the orbitals may be populated when the electron transfer occurs since the coordination of the lanthanide fragment can modulate their relative energy.^22,61–63^ If the shape of the LUMO+1 suggests the dimerization of the pyrimidine ring (1 to 2) owing to the large density coefficient at this site, the LUMO indicates a possible transfer to the pyridine ring, thus triggering the observed C–H activation (2 to 5).

**Fig. 4:**
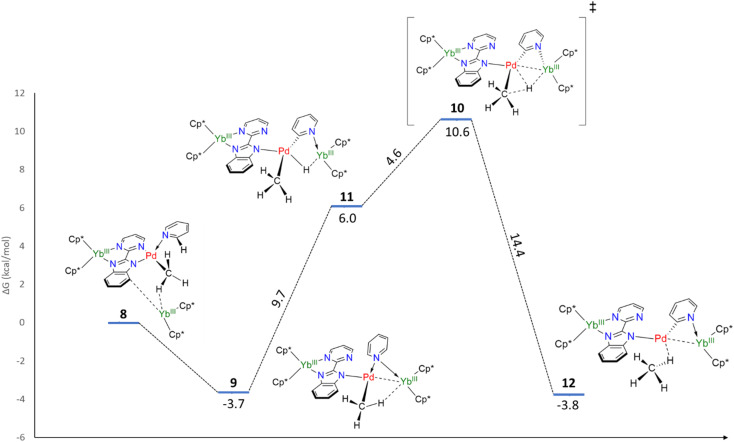
Free energy profile of the C–H activation step.

Without the presence of an excess of Cp*_2_Yb, the formation of 5–7 is not observed. However, incorporating a further organolanthanide fragment into either 2 or 3 (dimers) is computationally unfeasible. Therefore, adding a Cp*_2_Yb fragment to a monomeric heterobimetallic Yb–Pd complex (equivalent to half of 2) is an adequate compromise. Although this approximation is not perfect, it would allow us to locate the energy of a possible C–H activation transition state and verify if the pathway is feasible. This species, 8, was optimized as a triplet, in agreement with the relevant literature.^60^ The computations successfully reproduced the two-electron transfers (one from each ytterbium), as the main structural features for each Yb center are consistent with the trivalent state. Owing to the steric repulsion, the second Yb fragment cannot approach the Pd center and nestles in between the methyl group and the imidazolyl moiety of the bimpm ligand. The placement of the second organolanthanide fragment – significantly tilted with respect to the bimpm-Pd plane^60^ – validates the choice of 8 as a scaffold for computing the C–H activation because the other half of the dimeric species could be accommodated, despite the addition of the bulky Cp*_2_Yb fragment. 8 relaxes towards a more stable configuration, 9, where the Pd center decoordinates from the pyrimidyl moiety and accommodates the approach of the second Yb fragment. The computed transition state (TS), 10, is the synergistic H transfer from the pyridine to the methyl group nearby. From the TS saddle point, the closest stable intermediates show that the activated H atom bridges the two metal atoms (11) before transitioning towards the methyl moiety (the TS, 10) and ultimately forming a methane molecule (12). The energy profile of this transformation is shown in Fig. 4. The release of the methane molecule leads to a μ_2_-pyridyl Pd fragment (13, not shown in Fig. 4). The formation of the pyridyl is the starting point of the ligand rearrangement, which ultimately forms the compounds 5 to 7 (see the ESI† for computational details).

The magnetic characterization of 4 and 5 leaves no doubt as to the electronic configuration of the metal centers over a large range of temperatures. Therefore, the computations were run considering non-interacting Ln^III^ metal centers. This implies a triplet for 5 and 10 unpaired spins for 4. The computed structures reproduce the main features of the trimetallic adducts. Of particular interest is the relative distance between the Yb and Sm adducts. The computed Sm–Pd and Yb–Pd distances of 2.925 Å *vs.* 2.902 Å, respectively, are similar to those reported in the XRD structures, reproducing the apparent break in the lanthanide contraction. The electronic structures of 4 and 5 were screened for orbitals with contributions from the Ln 4f electrons and the Pd 4d. These sub-levels are low-lying in energy – the 4f electrons are approximately 4.33 and 6.07 eV below the SOMO in 4 and 5, respectively. There were no definitive signs of orbital interaction in either case, despite a Pd 4d_z_^2^ orbital pointed towards the Ln^III^ centers (see Fig. S38 and S40†).

**Fig. 5:**
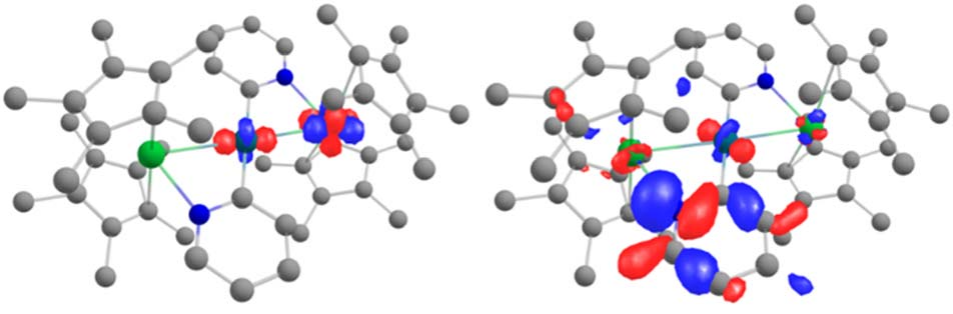
CASSCF orbitals 254 and 255 for 5, where contributions from both Yb and Pd are observed. See the ESI† for relative energies.

Further analyses were carried out at the CASSCF level, using the DFT-optimized geometries (see details for the active spaces of 4 and 5 in the ESI†). From the CASSCF computations, no significant interactions were noted between the palladium and the lanthanide ions, and only a few orbitals show both metallic contributions (4% of d contribution in CASSCF orbital 254, Fig. 5, left).

ELF analyses found valence basins, integrating a larger number of electrons, for the Ln–N_pyr_ and Pd–C_pyr_ bonds for both complexes (Fig. 7, left). Two valence basins were also found for Yb–Pd (no equivalent basin was found for 4), accounting for approximately 2 electrons, with a strong localization index (Fig. 7, center). This indicates that the electronic density is principally concentrated on the Yb^III^ center, with low participation from the Pd. Overall, the forces that ensure that the atypical structures of the trimetallic complexes are kept in place include a number of covalent interactions. Another component that may contribute is the stabilizing effect of dispersive forces. To this end, the non-covalent interaction mapping was performed in the density overlap region indicator (DORI) framework. In both cases, the [Pd(pyr)_2_]^2−^ fragment is englobed by a significant amount of non-covalent interactions with the Cp* co-ligands on the Ln centers. Stabilizing interactions are also found between the Cp* groups on the same and on the opposite metal centers. This is not surprising, as the large and relatively encumbered structures are conducive to these types of interactions.

Thus, the molecule can best be described as two [Cp*_2_Ln^III^]^+^ fragments interacting with a [Pd^0^(pyr)_2_]^2−^ fragment. The obtention and stabilization of the latter following the degradative mechanism triggered by the addition of Cp*_2_Yb to 2 or 3 is particularly remarkable, as such species would not be stable in solution. Two types of interactions should be distinguished: the ones between the fragments (the two [Cp*_2_Ln^III^]^+^ and one [Pd^0^(pyr)_2_]^2−^) and the ones within the fragments themselves. Within each fragment, strong interactions are present, that maintain each of the fragments together, notably the covalent Pd–C_pyr_ bond. The dominant interactions in between the fragments are electrostatic, alongside other attractive forces between the fragments themselves, which are not particularly strong (Ln–N_pyr_ coordination bonds and relatively weak Ln–Pd interactions).

The electronic structure of 6, computed for the doublet state at the DFT/PBE0-D3 level, does not suggest any interaction between the two metal centers near the frontier orbitals. The most striking feature is that the Pd 4d_z_^2^ orbital is pointed towards the Yb (HOMO−8 in Table S29†), which would create the spatial conditions for an orbital interaction. Indeed, the electronic structure at the CASSCF (9,12) level is significantly different. In the active space, the 9 electrons populate only Cp*-based antibonding orbitals, while the vacant orbitals involve similar interactions.

**Fig. 6:**
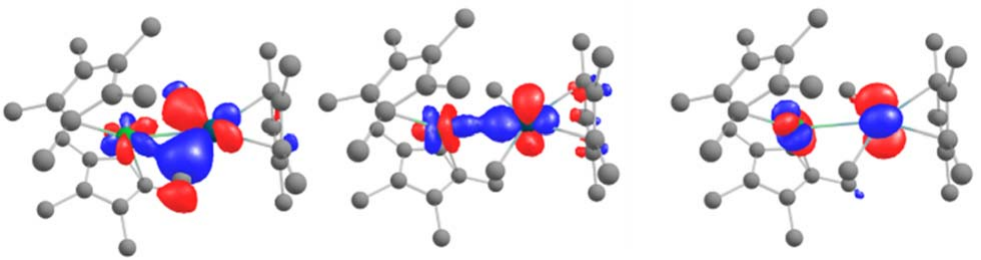
CASSCF orbitals 161, 162 and 164 and for 6, displaying orbital intermetallic interaction between Yb 4f and Pd 4d electrons. See the ESI† for relative energies.

The remarkable feature of this compound is found within a series of closely packed (all within 1.1 eV) orbitals below the active space, containing significant participation from both the Pd 4d and Yb 4f shells. Some of the orbitals involve an interaction over the bridging methyl groups (Fig. 6, left). Overall, these orbitals amount to 3 pairs of bonding and anti-bonding orbitals. This suggests a global bond order of zero between Yb and Pd. It is worth noting that, contrary to the DFT calculations, the Pd 4d_z_^2^ is involved in both bonding orbitals with the Yb and with the Cp*. This orientation of the 4d_z_^2^ orbital seems to be a key element in enabling the intermetallic interaction and could be construed to be the effect of the Cp* co-ligand, whose centroid is aligned along the Yb–Pd axis (see Fig. S36†), directing the orbital towards the Yb center.

**Fig. 7:**
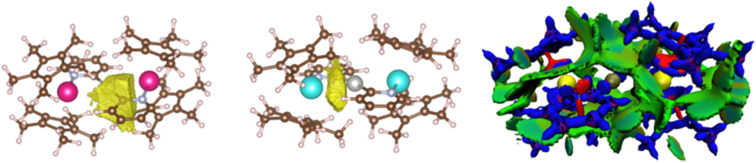
Electronic density analysis of the trimetallic complexes: the ELF basin of 4 showing the Pd–C_pyr_ interaction (left), basin corresponding to a Yb–Pd interaction on 5 (center) and DORI plot of 4 (right). Color-codes for the DORI plot: repulsive forces – red, covalent interaction – blue, and non-covalent interaction – green.

Previous examples of heterometallic complexes where wave-function methods were used found an interaction that placed the contribution of the lanthanide at less than 10%,^31^ a figure which is clearly exceeded in this case. The bonds in the cited examples were qualified as dative.

The analysis of the orbitals shown in the center and the right of Fig. 6 shows Yb/Pd orbital contribution ratios of 63/37 and 60/40, figures which are unheard of in the literature. To better quantify the contributions of each type of force, energy decomposition analysis (EDA) was carried out for 6 by considering a [Cp*_2_Yb^III^]^+^ fragment and a (Cp*PdMe_2_)^−^ fragment. When the fragments are computed as neutral species, the orbital interactions play the dominant role in the overall bonding energy – they constitute 66% of the attractive interaction, compared to 31% when the fragments are computed as charged species. The EDA analysis carried out by the group of Hall in a recent study^40^ resulted in comparable differences in the relative contribution of the attractive orbital energy, depending on the charge of the fragments. In this study, the authors marked the difference between a situation with 26% of orbital interaction, analyzed as predominantly electrostatic, and another with 35% of orbital interaction, analyzed as predominantly covalent. In our study, the percentage is 31% for 6 – an intermediate value between predominantly ionic and covalent interactions.

Other indicators commonly used to quantify intermetallic interactions are bond indices. Two of the more frequently used are the Wiberg bond order (WBO)^64,65^ and the formal shortness ratio (FSR).^66^ In both metrics, the complexes presented in this work are competitive with the references in the field (Table S38†). There are some inconsistencies worth highlighting, since the complex with the highest apparent bond order, 4, offers no sign of any orbital interaction in the electronic structure and its ELF data shows no valence basin corresponding to the Sm–Pd interaction. On the other hand, the complex with the lowest WBO, 6, displays a set of orbitals, showing strong participation from both metals – a unique situation in lanthanide-based intermetallics. These results highlight the difficulty in assessing the nature and strength of the interaction on the basis of such empirical metrics.

## Conclusions

This work presents the reactivity of an unsymmetrical ligand bearing a Pd^II^ fragment with divalent lanthanide fragments. Adding one equivalent of Cp*_2_Yb(OEt_2_) results in an electron transfer and a clean coupling reaction. However, using an excess of Cp*_2_Yb(OEt_2_) or the more reductive Cp*_2_Sm(OEt_2_) complex results in the formation of isostructural complexes, 4 and 5, with linear Ln–Pd–Ln arrangements accompanied by a methyl-bridged adduct also featuring a short Yb–Pd distance, 6. In all cases, the Ln–Pd distance is under 3 Å and below the sum of covalent radii.

The magnetic analyses performed for the heterotrinuclear complexes revealed that these adducts are best described as Ln^III^–Pd^0^–Ln^III^ complexes. Within this context, the nearly identical Ln–Pd distances reported in both complexes are surprising, given the expected difference in the ionic radii of trivalent Sm and Yb.

The theoretical computations performed on 4 and 5 showed no significant orbital interaction – *i.e.,* sharing of electrons – between the metals. Instead, the interactions should be qualified as weak and dative. The trinuclear edifices are held together by a comparable mixture of dispersive forces, covalent interactions between Ln–N_pyr,_ and electrostatic interactions between the two [Cp*_2_Yb]^+^ cations and the dianionic [Pd(pyr)_2_]^2−^ fragment, which results in similar Ln–Pd distances in both 4 and 5.

In contrast, the electronic structure of 6, whose Yb–Pd distance is similar to that in 5, shows an intriguing series of orbitals, displaying strong and equitable interactions between the Yb 4f and the Pd 4d electrons. The multireference electronic structure is consistent with the overall low bond order between the two metals, which is corroborated by the topological analyses.

Ultimately, these three complexes help illustrate the difficulty in assessing the inter-metallic interaction in such adducts and show that the short distance between the metal centers is not indicative of the nature of the chemical interaction between them. Accordingly, the same is true for the computed bond indices, which are extrapolated on the basis of covalent radii, the latter consisting of empirically created averages for each element. This is due to the fact that the coordination environment of either of the metal fragments can be tailored to approach the metals together without necessarily increasing the intermetallic interaction.

Without extensive experimental and theoretical support, the bonding schemes that the short intermetallic distances infer should be interpreted cautiously. The continuous evolution of the theoretical bases of the organometallic chemistry of lanthanides would suggest that there is much yet to learn about their behavior in complex situations, such as lanthanide-based intermetallics.

## Data availability

The datasets supporting this study are available from the authors upon reasonable request and other relevant information is given in the provided ESI.†

## Author contributions

V. C. and A. J. carried out the synthetic experiments. V. C, T. S. and J. M. analyzed the experimental data. V. C. and C. C. carried out and analyzed the computational data. G. N. originated the central idea, coordinated the work and analyzed all data. G. N., C. C. and V. C. wrote the manuscript with significant inputs from all co-authors.

## Conflicts of interest

There are no conflicts to declare.

## Supplementary Material

SC-014-D2SC06933D-s001

SC-014-D2SC06933D-s002

## References

[cit1] Luzon J., Sessoli R. (2012). Dalton Trans..

[cit2] Rinehart J. D., Long J. R. (2011). Chem. Sci..

[cit3] Balaram V. (2019). Geosci. Front..

[cit4] Van Gosen B. S. , Verplanck P. L., Long K. R., Gambogi J. and Seal II R. R., The rare-earth elements: vital to modern technologies and lifestyles, U.S. Geological Survey, 2014

[cit5] Han S., Yi Z., Zhang J., Gu Q., Liang L., Qin X., Xu J., Wu Y., Xu H., Rao A., Liu X. (2021). Nat. Commun..

[cit6] Su Q., Wei H.-L., Liu Y., Chen C., Guan M., Wang S., Su Y., Wang H., Chen Z., Jin D. (2021). Nat. Commun..

[cit7] Joos J. J., Van der Heggen D., Martin L. I. D. J., Amidani L., Smet P. F., Barandiarán Z., Seijo L. (2020). Nat. Commun..

[cit8] Gao C., Genoni A., Gao S., Jiang S., Soncini A., Overgaard J. (2020). Nat. Chem..

[cit9] Goodwin C. A. P., Ortu F., Reta D., Chilton N. F., Mills D. P. (2017). Nature.

[cit10] Guo F.-S., Day B. M., Chen Y.-C., Tong M.-L., Mansikkamäki A., Layfield R. A. (2018). Science.

[cit11] Münzfeld L., Schoo C., Bestgen S., Moreno-Pineda E., Köppe R., Ruben M., Roesky P. W. (2019). Nat. Commun..

[cit12] Palumbo C. T., Zivkovic I., Scopelliti R., Mazzanti M. (2019). J. Am. Chem. Soc..

[cit13] Willauer A. R., Palumbo C. T., Fadaei-Tirani F., Zivkovic I., Douair I., Maron L., Mazzanti M. (2020). J. Am. Chem. Soc..

[cit14] Rice N. T., Popov I. A., Russo D. R., Bacsa J., Batista E. R., Yang P., Telser J., La Pierre H. S. (2019). J. Am. Chem. Soc..

[cit15] MacDonald M. R., Fieser M. E., Bates J. E., Ziller J. W., Furche F., Evans W. J. (2013). J. Am. Chem. Soc..

[cit16] Guo F.-S., Tsoureas N., Huang G.-Z., Tong M.-L., Mansikkamäki A., Layfield R. A. (2020). Angew. Chem., Int. Ed..

[cit17] La Pierre H. S., Scheurer A., Heinemann F. W., Hieringer W., Meyer K. (2014). Angew. Chem., Int. Ed..

[cit18] Billow B. S., Livesay B. N., Mokhtarzadeh C. C., McCracken J., Shores M. P., Boncella J. M., Odom A. L. (2018). J. Am. Chem. Soc..

[cit19] Barluzzi L., Giblin S. R., Mansikkamäki A., Layfield R. A. (2022). J. Am. Chem. Soc..

[cit20] Halbach R. L., Nocton G., Booth C. H., Maron L., Andersen R. A. (2018). Inorg. Chem..

[cit21] Tricoire M., Mahieu N., Simler T., Nocton G. (2021). Chem.–Eur. J..

[cit22] Nocton G., Lukens W. W., Booth C. H., Rozenel S. S., Medling S. A., Maron L., Andersen R. A. (2014). J. Am. Chem. Soc..

[cit23] Butovskii M. V., Döring C., Bezugly V., Wagner F. R., Grin Y., Kempe R. (2010). Nat. Chem..

[cit24] Pöttgen R. , in Rare earth chemistry, ed. R. Pöttgen, T. Jüstel and C. A. Strassert, De Gruyter, Berlin, 2020, pp. 75–82

[cit25] Beletskaya I. P., Voskoboynikov A. Z., Chuklanova E. B., Kirillova N. I., Shestakova A. K., Parshina I. N., Gusev A. I., Magomedov G. K. I. (1993). J. Am. Chem. Soc..

[cit26] Spannenberg A., Oberthür M., Noss H., Tillack A., Arndt P., Kempe R. (1998). Angew. Chem., Int. Ed..

[cit27] Kempe R., Noss H., Fuhrmann H. (2001). Chem.–Eur. J..

[cit28] Butovskii M. V., Tok O. L., Wagner F. R., Kempe R. (2008). Angew. Chem., Int. Ed..

[cit29] Cordero B., Gómez V., Platero-Prats A. E., Revés M., Echeverría J., Cremades E., Barragán F., Alvarez S. (2008). Dalton Trans..

[cit30] Völcker F., Mück F. M., Vogiatzis K. D., Fink K., Roesky P. W. (2015). Chem. Commun..

[cit31] Ramirez B. L., Sharma P., Eisenhart R. J., Gagliardi L., Lu C. C. (2019). Chem. Sci..

[cit32] Magott M., Brzozowska M., Baran S., Vieru V., Pinkowicz D. (2022). Nat. Commun..

[cit33] Liddle S. T., Mills D. P. (2009). Dalton Trans..

[cit34] Shi K., Douair I., Feng G., Wang P., Zhao Y., Maron L., Zhu C. (2021). J. Am. Chem. Soc..

[cit35] Kempe R., Noss H., Irrgang T. (2002). J. Organomet. Chem..

[cit36] Oelkers B., Butovskii M. V., Kempe R. (2012). Chem.–Eur. J..

[cit37] Butovskii M. V., Kempe R. (2015). New J. Chem..

[cit38] Fang W., Zhu Q., Zhu C. (2022). Chem. Soc. Rev..

[cit39] Pyykkö P., Atsumi M. (2009). Chem.–Eur. J..

[cit40] Yang X., Burns C. P., Nippe M., Hall M. B. (2021). Inorg. Chem..

[cit41] Burns C. P., Yang X., Sung S., Wofford J. D., Bhuvanesh N. S., Hall M. B., Nippe M. (2018). Chem. Commun..

[cit42] Shannon R. D. (1976). Acta Crystallogr., Sect. A: Cryst. Phys., Diffr., Theor. Gen. Crystallogr..

[cit43] Goudy V., Jaoul A., Cordier M., Clavaguéra C., Nocton G. (2017). J. Am. Chem. Soc..

[cit44] Wang D., Moutet J., Tricoire M., Cordier M., Nocton G. (2019). Inorganics.

[cit45] Wang D., Tricoire M., Cemortan V., Moutet J., Nocton G. (2021). Inorg. Chem. Front..

[cit46] Appleton T. G., Clark H. C., Manzer L. E. (1973). Coord. Chem. Rev..

[cit47] Evans W. J., Grate J. W., Levan K. R., Bloom I., Peterson T. T., Doedens R. J., Zhang H., Atwood J. L. (1986). Inorg. Chem..

[cit48] Newkome G. R., Evans D. W., Fronczek F. R. (1987). Inorg. Chem..

[cit49] Poulain A., Neels A., Albrecht M. (2009). Eur. J. Inorg. Chem..

[cit50] Stander-Grobler E., Schuster O., Heydenrych G., Cronje S., Tosh E., Albrecht M., Frenking G., Raubenheimer H. G. (2010). Organometallics.

[cit51] Du J., Zhang Y., Huang Z., Zhou S., Fang H., Cui P. (2020). Dalton Trans..

[cit52] Cui P., Wu C., Du J., Luo G., Huang Z., Zhou S. (2021). Inorg. Chem..

[cit53] Du J., He X., Hong D., Zhou S., Fang H., Cui P. (2022). Dalton Trans..

[cit54] Hlina J. A., Pankhurst J. R., Kaltsoyannis N., Arnold P. L. (2016). J. Am. Chem. Soc..

[cit55] Kahn O. , Molecular magnetism, Wiley-VCH, New York, 4, 2001

[cit56] Ferrer M., Gutiérrez A., Mounir M., Rodríguez L., Rossell O., Font-Bardia M., Gómez-Sal P., Martín A., Solans X. (2011). Organometallics.

[cit57] Tyrra W., Aboulkacem S., Pantenburg I. (2006). J. Organomet. Chem..

[cit58] Mocanu T., Kiss L., Sava A., Shova S., Silvestru C., Andruh M. (2019). Polyhedron.

[cit59] Partyka D. V., Gray T. G. (2009). J. Organomet. Chem..

[cit60] Jaoul A., Tricoire M., Moutet J., Cordier M., Clavaguéra C., Nocton G. (2019). Chem. Sq..

[cit61] Nocton G., Booth C. H., Maron L., Ricard L., Andersen R. A. (2014). Organometallics.

[cit62] Nocton G., Booth C. H., Maron L., Andersen R. A. (2013). Organometallics.

[cit63] Boyce S. A. J., Moutet J., Niederegger L., Simler T., Nocton G., Hess C. R. (2021). Inorg. Chem..

[cit64] Liddle S. T., Mills D. P., Gardner B. M., McMaster J., Jones C., Woodul W. D. (2009). Inorg. Chem..

[cit65] Feng G., McCabe K. N., Wang S., Maron L., Zhu C. (2020). Chem. Sci..

[cit66] Lu E., Wooles A. J., Gregson M., Cobb P. J., Liddle S. T. (2018). Angew. Chem., Int. Ed..

