# Supplementary material for: Structure and bonding patterns in heterometallic organometallics with linear Ln–Pd–Ln motifs
Source: Chem Sci. 2023 Jan 20;14(10):2676–85. doi: 10.1039/d2sc06933d (PMC9993901; doi:10.1039/d2sc06933d)
Supplement: SC-014-D2SC06933D-s001 [file SC-014-D2SC06933D-s001.pdf]

Electronic Supplementary Material (ESI) for Chemical Science.
This journal is © The Royal Society of Chemistry 2023

Supporting Information for the manuscript:

Structure and Bonding Patterns in Heterometallic Organometallics with linear Ln-Pd-Ln motifs

Valeriu Cemortan,[a,b] Thomas Simler,[a] Jules Moutet,[a] Arnaud Jaoul,[a] Carine Clavaguéra[b]* and Grégory Nocton[a]*

a. LCM, CNRS, Ecole polytechnique, Institut Polytechnique de Paris, Route de Saclay, 91120 Palaiseau, France

b. Université Paris-Saclay, CNRS, Institut de Chimie Physique, UMR8000, 91405 Orsay, France.

Table of contents

I. Syntheses S4

II. ^{1}H NMR S7

1. Complex **1** S7
2. Compounds **2** and **3** S8
   a) NMR of compounds S8
   b) *In situ* studies of addition of $Cp^*_2Yb(OEt_2)$ S11
3. Complex **4** S16
   a) NMR of compounds S16
   b) Variable temperature NMR of complex **4** S18
   c) Formation reactions of compound **4** S20
4. Complex **5** S22
   a) NMR of compounds S22
   b) Variable temperature NMR of complex **5** S23
   c) *In situ* formation reactions of **5** S25
5. Compound **6** S29

III. Magnetism S30

IV. Crystallographic data S31

V. Theoretical calculations S66

1. Geometry optimisations and electronic structure S67
   a) Compound **1** S67
   b) Compound **4** S69
      1) DFT calculations S69
      2) CASSCF calculations S75
   c) Compound **5** S77
      1) DFT calculations S77
      2) CASSCF calculations S83
   d) Compound **6** S87
2. Topological analyses S93
   a) **Density Overlap Regions Indicator (DORI) results** S93
   b) **QTAIM** analysis S95
   c) **ELF** analysis S96
3. C-H activation – calculating the transition state S100
   1. Computations on the compound **1** S100
      a) Transition state calculations S100
      b) Second possible transition state S101

c) Computing the departure of the methyl S102
d) Comparison between the two possible transition states S102
2. Computations with addition of lanthanides S104
a) Modelling an interaction between Cp^*_2Ln and compound **1** S104
3. Computations with the explicit addition of another lanthanide S109
a) Modelling the addition of a further lanthanide S109
b) Species on the full C-H activation energy profile S113
References S117

I. Syntheses

All reactions were performed using standard Schlenk-line techniques or in nitrogen-filled gloveboxes (MBraun, Garching, Germany). All glassware was dried at 140 °C for at least 12 h prior to use. Tetrahydrofuran (THF), THF-d_8, toluene, and toluene-d_8 were dried over sodium, degassed, and transferred under reduced pressure in a cold flask. Acetonitrile (MeCN), MeCN-d_3, pyridine (pyr), pyr-d_5 were dried over CaH_2, distilled and degassed prior to use.^{1}H NMR spectra were recorded in 5-mm tubes adapted with a J. Young valve on Bruker AVANCE II or III-300 MHz (Bruker, Billerica, MA, USA) spectrometers. ^{1}H chemical shifts were expressed relative to TMS (tetramethylsilane) in ppm. Temperature dependent magnetic susceptibility measurements were made with a SQUID in sealed quartz tube on a SQUID at 0.5 and 2 T. Diamagnetic corrections were made using Pascal's constants. Elemental analyses were obtained from Mikroanalytisches Labor Pascher (Remagen, Germany). 2-(benzimidazol-2-yl)pyrimidine (Hbimpm),[1] (tmeda)Pd(Me)$_2$,[2] Cp*$_2$Sm(OEt$_2$)[3] and Cp*$_2$Yb(OEt$_2$)[4] were synthetized according to described procedures.

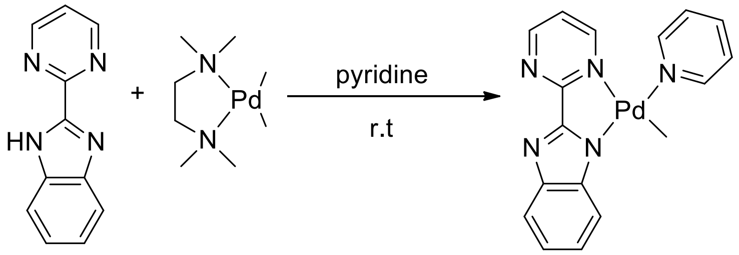

(bimpm)Pd(pyr)(Me) (1). Hbimpm (390 mg, 2.0 mmol, 1 eq) and (tmeda)Pd(Me)$_2$ (520 mg, 2.05 mmol, 1.03 eq) were dissolved in pyridine. The initially colourless suspension gradually turned to a golden solution within 30 minutes. An intense gaseous evolution was concurrently observed. The mixture was stirred for an hour. Adding pentane to the solution precipitated the product as a beige solid. The solid was recovered by filtering the mixture. Further washing with pentane and diethyl ether yielded analytically pure (bimpm)Pd(py)(Me), **1** (700 mg, 1.77 mmol, 88% yield). Crystals suitable for XRD studies were obtained by dissolving the compound in pyridine (golden solution), filtering it through a MF-Millipore membrane filter and performing a low vapour diffusion of pentane in the pyridine solution at room temperature. **^{1}H NMR (300 MHz, THF-d_8, 60 °C): δ (ppm):** 8.51 (2H, pyridine), 8.30 (1H, pyrimidine), 8.07 (2H, pyrimidine), 7.62 (1H, py), 7.20 (2H, py), 7.15 (4H, benzimidazole), 1.10 (3H, CH_3). Anal. calculated for $C_{17}H_{15}N_5Pd$: C, 51.59; H, 3.82; N, 17.70; Pd, 26.89. Found: C, 45.87, H, 3.89, N, 11.4.

(bimpm)Pd(py-d_5)(Me) (1-py-d_5). Acetonitrile was poured over Hbimpm (80 mg, 0.4 mmol, 1 eq) and (tmeda)Pd(Me)$_2$ (110 mg, 0.43 mmol, 1.06 eq) under stirring, yielding a beige suspension. A few drops of pyr-d_5 were then added to the mixture. Within 30 minutes, the suspension acquired a rich golden hue. The mixture was stirred for 4 h. A gaseous evolution was concurrently observed. At the end of the reaction, all the volatiles were evaporated, after which a yellow powder was obtained. Further washing with pentane and diethyl ether yielded (bimpm)Pd(py-d_5)(Me) (150 mg, 0.36 mmol, 91% yield).

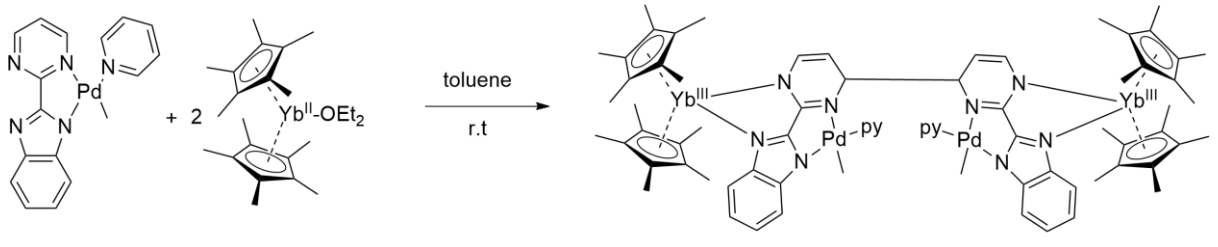

2

[Cp*$_2$Yb(bimpm)Pd(py)(Me)]$_2$ (2). Toluene was added to a mixture of (bimpm)Pd(Pyr)(Me) (**1**) (54 mg, 0.14 mmol, 1.4 eq) and Cp*$_2$Yb(OEt$_2$) (52 mg, 0.10 mmol, 1.0 eq). The resulting black suspension was stirred for approximately 30 s before the supernatant was filtered. The black solution was evaporated under vacuum without stirring. The black-brown powder was washed thrice with cold pentane. Evaporating the volatiles yielded [Cp*$_2$Yb(bimpm)Pd(py)(Me)]$_2$ (**2**) as a brown powder (91 mg, 0.054 mmol, 95% yield). Crystals suitable for XRD analysis were grown by dissolving the powder in toluene. Adequate plates were formed after a few days by storing the solution at -40 °C. The product is thermally sensitive above 70 °C. **^{1}H NMR (300 MHz, Tol-d$_8$): δ (ppm):** 210.62 (s, 2H), 188.21 (s, 2H), 50.12 (s, 2H), 30.29 (s, 2H), 26.43 (s, 2H), 16.95 (s, 2H), 5.31 (s, Cp*, 30H), 3.21 (s, Cp*, 30H), -3.08 (s, 2H), -4.46 (s, Me, 6H), -9.29 (s, 2H), -24.80 (s, 2H). Anal. calculated for $C_{74}H_{90}N_{10}Pd_2Yb_2$: C, 52.89; H, 5.52; N, 8.33. Found: C, 53.04, H, 5.48, N, 8.45.

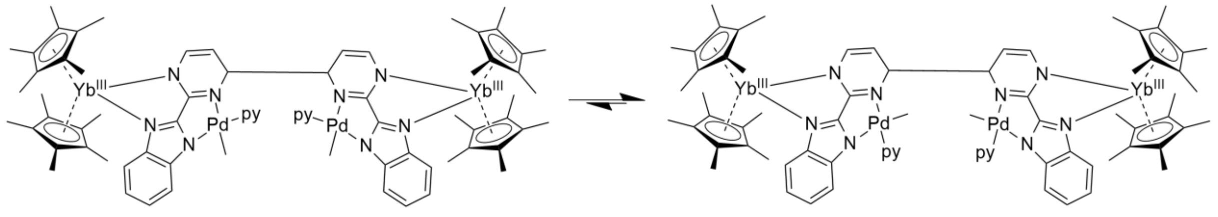

2 3

[Cp*$_2$Yb(bimpm)Pd(Me)(py)]$_2$ (3) : Toluene was added to 30 mg of **2**. The dark red-brown solution was stirred at room temperature for 3 days, during which the solution acquired a darker hue. The solution was evaporated under reduced pressure, quantitatively yielding **3** as an analytically pure brown powder. Crystals suitable for XRD analysis were grown by dissolving the powder in toluene. Thin red-brown needles were formed after a few days by storing the solution at -40 °C. **^{1}H NMR (300 MHz, Tol-d$_8$): δ (ppm):** 229.08 (s, 2H), 189.21 (s, 2H), 49.59 (s, 2H), 39.99 (s, 2H), 25.16 (s, 2H), 13.44 (s, 2H), 5.48 (s, Cp*, 30H), 2.49 (s, Cp*, 30H), -0.08 (s, 4H, py), -14.45 (s, 2H), -33.38 (s, Me, 6H). Anal. calculated for $C_{74}H_{90}N_{10}Pd_2Yb_2$: C, 52.89; H, 5.52; N, 8.33. Found: C, 51.98, H, 5.26, N, 7.95. The product is thermally sensitive above 70 °C.

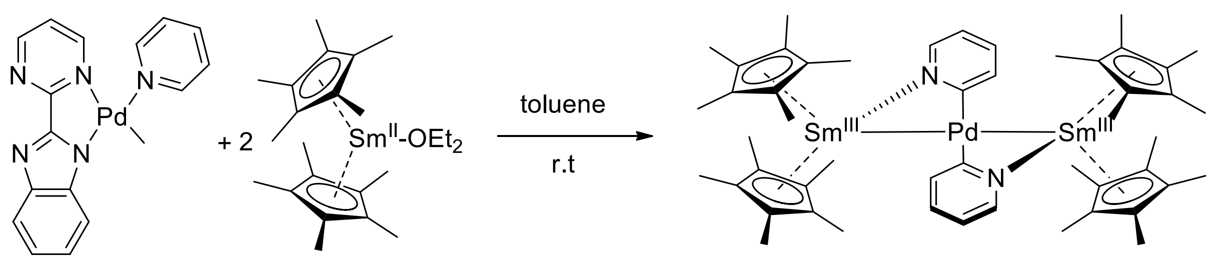

(Cp*$_2$Sm)$_2$[μ-Pd(pyridyl)$_2$] (4). Toluene was added to a mixture of (bimpm)Pd(Pyr)(Me) (**1**) (100 mg, 0.25 mmol, 1.0 eq) and Cp*$_2$Sm(OEt$_2$) (250 mg, 0.52 mmol, 2.1 eq). A black solution with yellow hues was formed instantly. A gaseous evolution was also concurrently observed. The solution was stirred for 30 minutes at room temperature before being filtered. The solution was concentrated under

reduced pressure and left overnight in the freezer at -40 °C to obtain brown blocks, suitable for XRD studies. The crystals were dried, washed 5 times with cold pentane, dried again to yield **4** (28.4 mg, 10% yield). The washing solutions were evaporated and the solid was re-dissolved in toluene. Upon concentration and crystallisation, further clean crystals were obtained at -40 °C. **^{1}H NMR (300 MHz, THF-d$_8$): δ (ppm):** 4.87 (s, 2H, pyridyl), 4.64 (s, 2H, pyridyl), 1.11 (s, Cp*, 60H), -0.81 (s, pyridyl, 2H), -9.06 (s, pyridyl, 2H). Anal. Calculated for $C_{50}H_{68}N_2PdSm_2$: C, 54.39; H, 6.21; N, 2.54; Pd, 9.64; Sm, 27.23. Found: C, 54.83; H, 6.45; N, 3.33.

(Cp*$_2$Sm)$_2$[μ-Pd(pyridyl-d$_4$)$_2$] (4-py-d$_4$). The procedure used was identical to that used for the synthesis of **4**, using **1-py-d$_5$** instead of **1.**

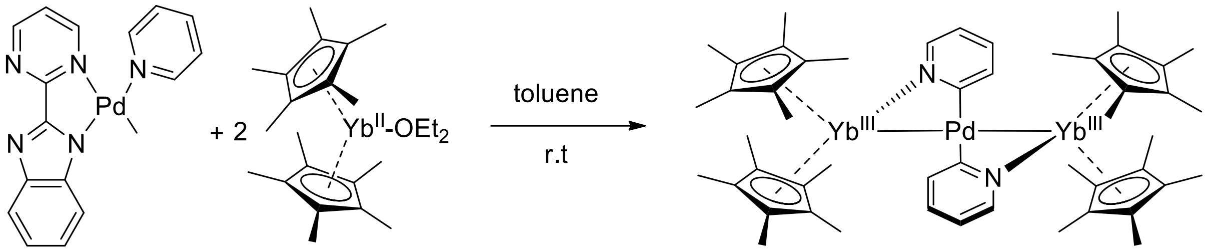

(Cp*$_2$Yb)$_2$[μ-Pd(pyridyl)$_2$] (5). Toluene was added to a mixture of (bimpm)Pd(Py)(Me) (**1**) (100 mg, 0.25 mmol, 1 eq) and Cp*$_2$Yb(OEt$_2$) (265 mg, 0.51 mmol, 2.0 eq). Within 5 minutes, the resulting brown suspension gave a black solution. A gaseous evolution was also concurrently observed. The solution was stirred for 30 minutes at room temperature before being filtered. The solution was concentrated and left overnight in the freezer at -40 °C to obtain brown blocks of **5** suitable for XRD studies. The crystals were dried, washed 5 times with cold pentane, dried again to yield another crop of crystals (total amount of crystalline material: 36 mg, 0.03 mmol, 12% yield). The washing solutions were evaporated and the solid was re-dissolved in toluene. Upon concentration and crystallisation, an additional amount of clean crystals can be obtained. **^{1}H NMR (300 MHz, THF-d$_8$): δ (ppm):** 228.25 (s, 2H, pyridyl), 116.84 (s, 2H, pyridyl), 25.78 (s, 2H, pyridyl), 24.89 (s, 2H, pyridyl), 9.02(s, 60H, Cp*). Anal. Calculated for $C_{50}H_{66}N_2PdYb_2$: C, 52.24; H, 5.96; N, 2.44. Found: C, 52.08; H, 6.07; N, 3.53.

[Cp*$_2$Yb(μ-Me)$_2$PdCp*] (6) and **Cp*Yb(thf)[Cp*$_2$Yb(bimpm)]$_2$ (7)** are obtained as side-products in the above reaction. The products **6** and **7** were isolated from the last crystallization fractions in toluene. **6** can be obtained as the major product following an extraction in diethyl ether and evaporation of the solvent under reduced pressure. **^{1}H NMR (300 MHz, Tol-d$_8$): δ (ppm):** 388.22 (s, 6H, Me), 4.10 (s, 30H, Cp*Yb), -11.34 (s, 15H, Cp*Pd). No elemental analysis have been obtained.

II. ^{1}H NMR

1. Complex **1**

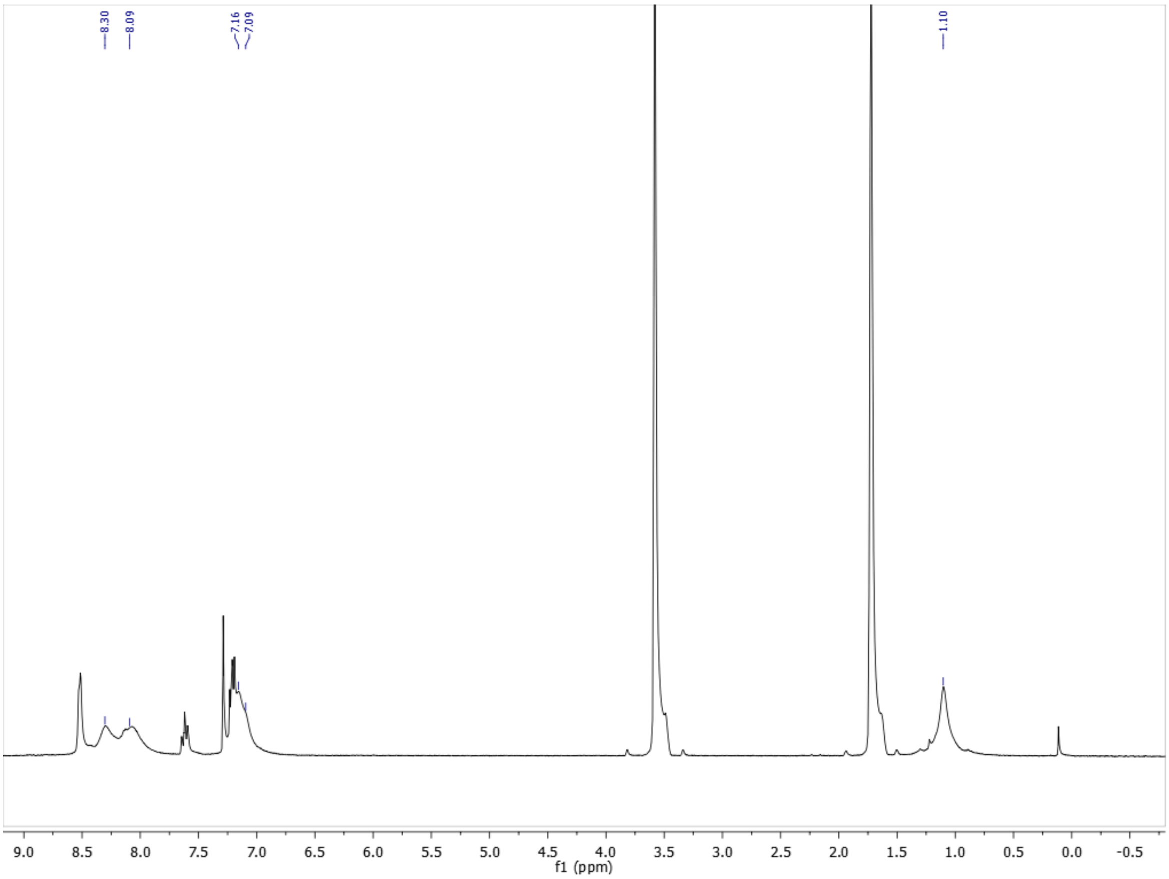

Figure S1. *^{1}H NMR of complex **1** in THF-d_8 at 333 K. The NMR spectrum was recorded at this temperature to sharpen the signals.*

2. Compounds **2** and **3**

a) NMR of compounds

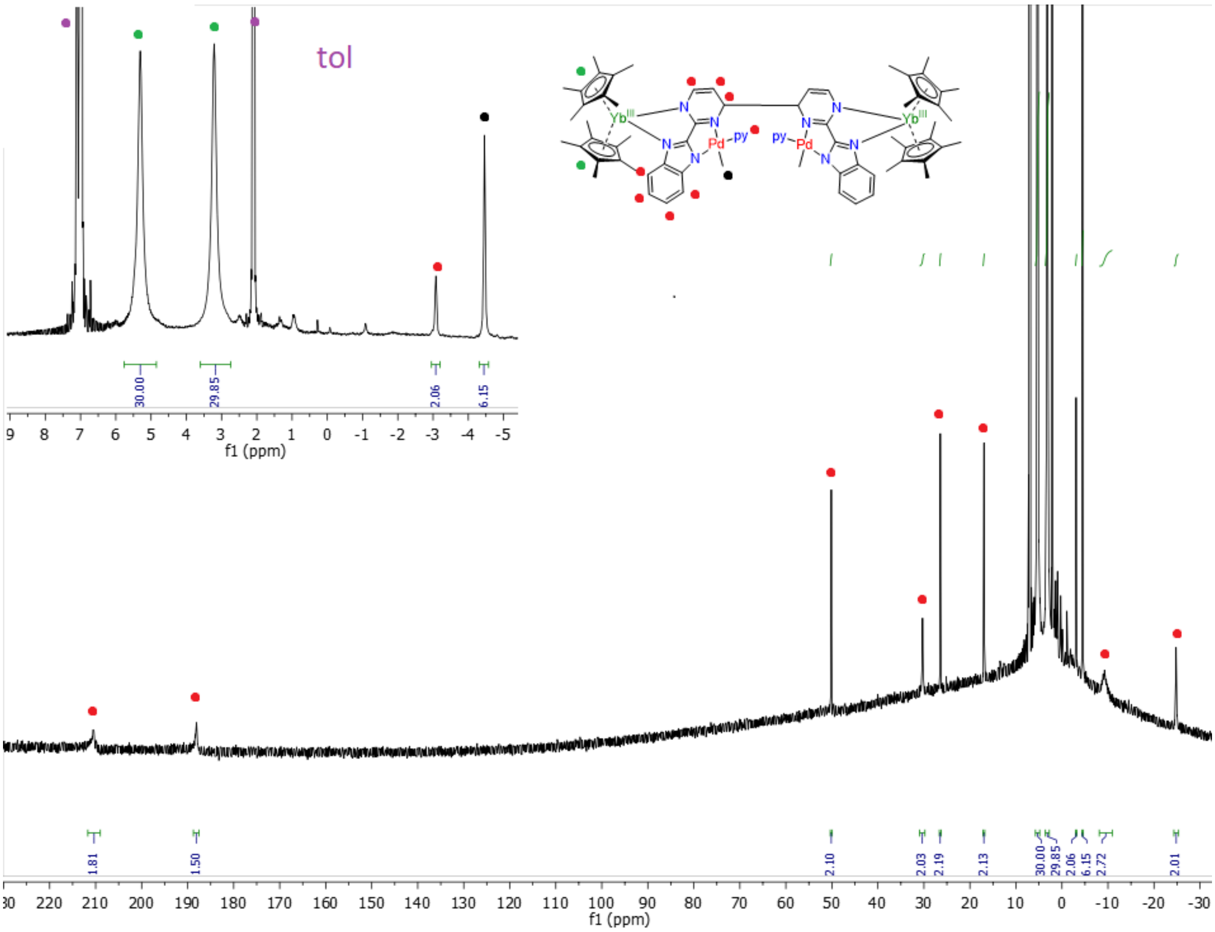

Figure S2. *^{1}H NMR of **2** in tol-d_8 at 293 K.*

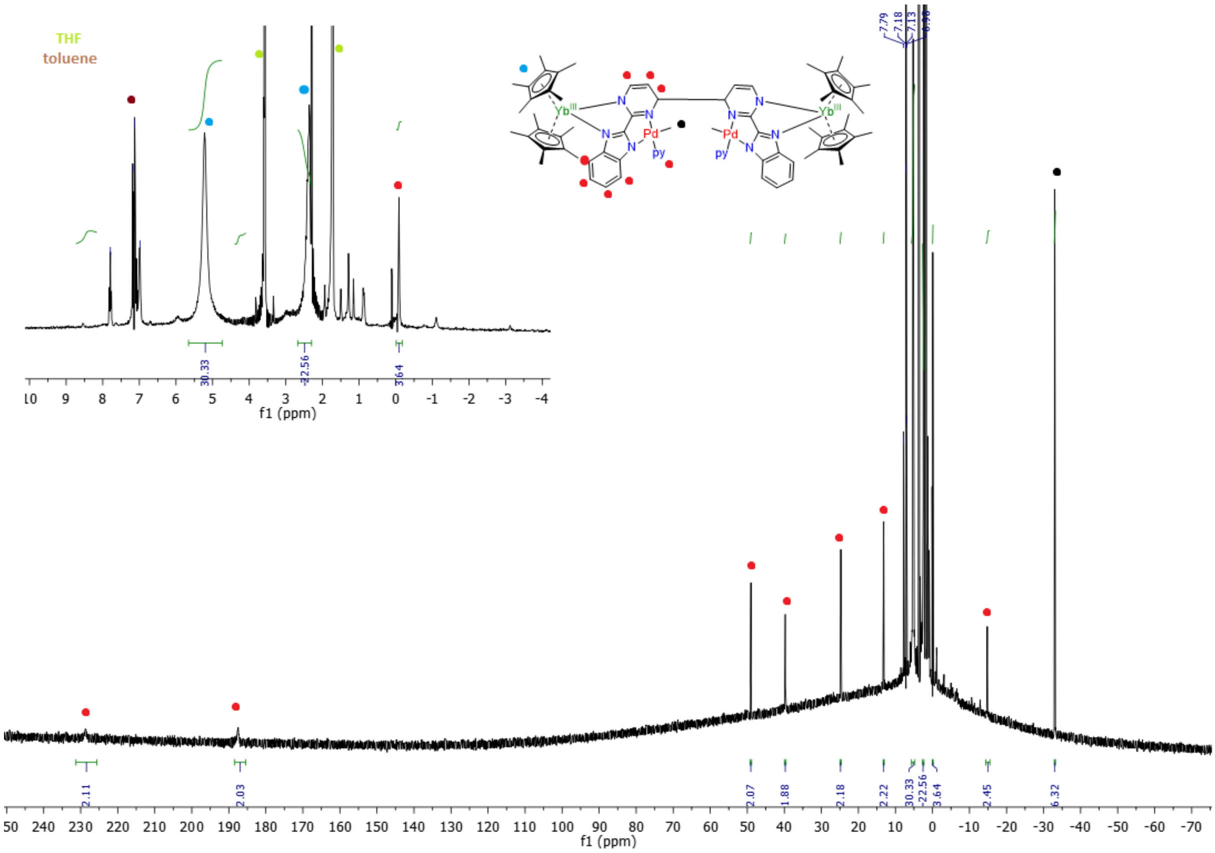

Figure S3. *^{1}H NMR of **3** in THF-d$_8$ at 293 K.*

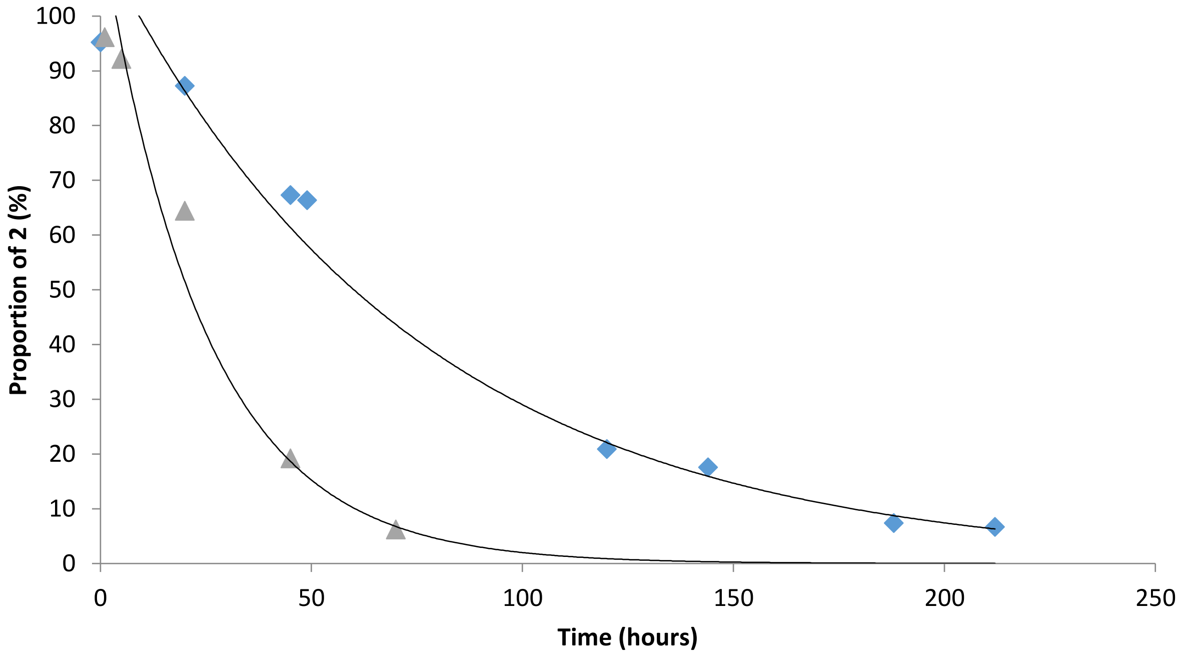

Figure S4. *Time evolution (hours) of the clean isomerisation of **2** to **3** without (blue lozenges) and with stirring (grey triangles).*

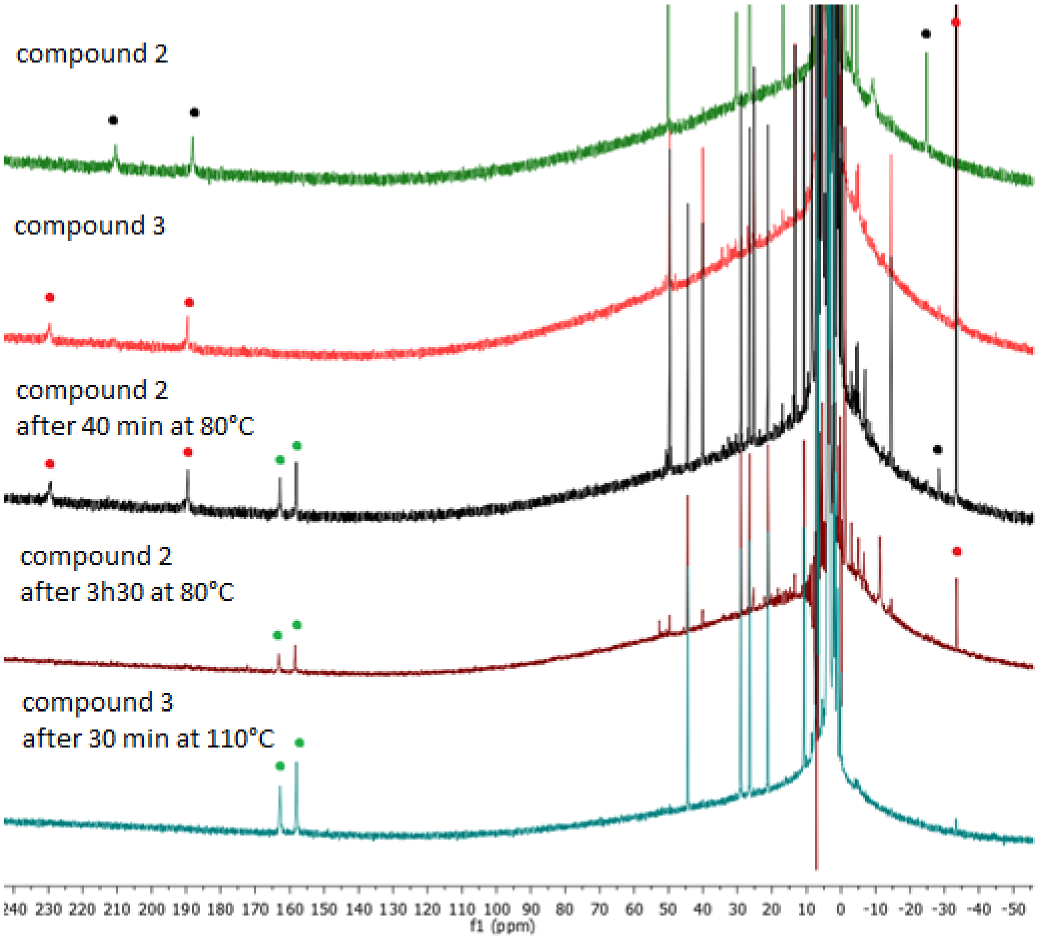

Figure S5. *The effect of heating on compounds **2** (black dots) and **3** (red dots). An unknown, but identical product, is formed in both cases (green dots).*

b) *In situ* studies of addition of $Cp*_2Yb(OEt_2)$

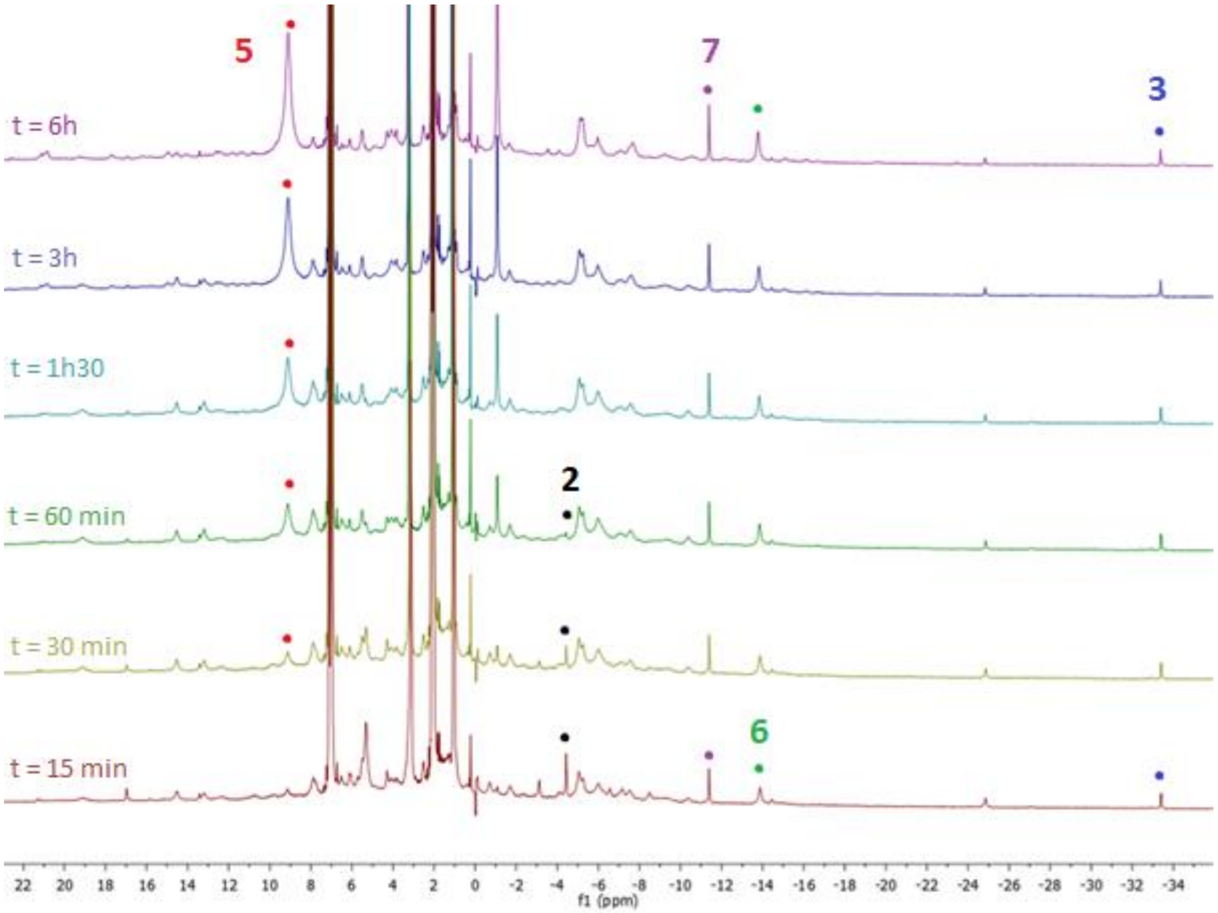

Figure S6. *In situ study of the addition of* $Cp*_2Yb(OEt_2)$ *to **2** over different reaction times. The signals consistent with the formation of **5**, **6** and **7** can be noted.*

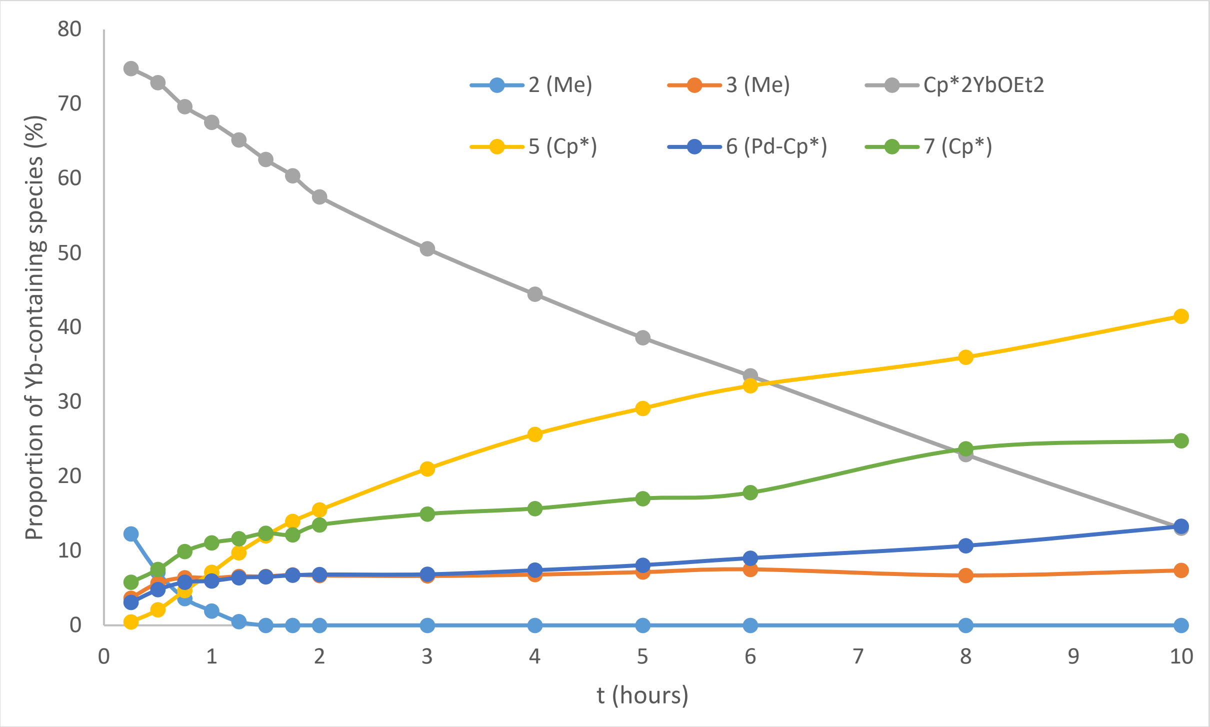

Figure S7. *In situ qualitative study of the addition of $Cp^*{}_2Yb(OEt_2)$ to* ***2.*** *For every compound, whenever possible, the signal with the cleanest integration was chosen. All the compounds were integrated with respect to an internal reference. Lines are only for guiding the eye.*

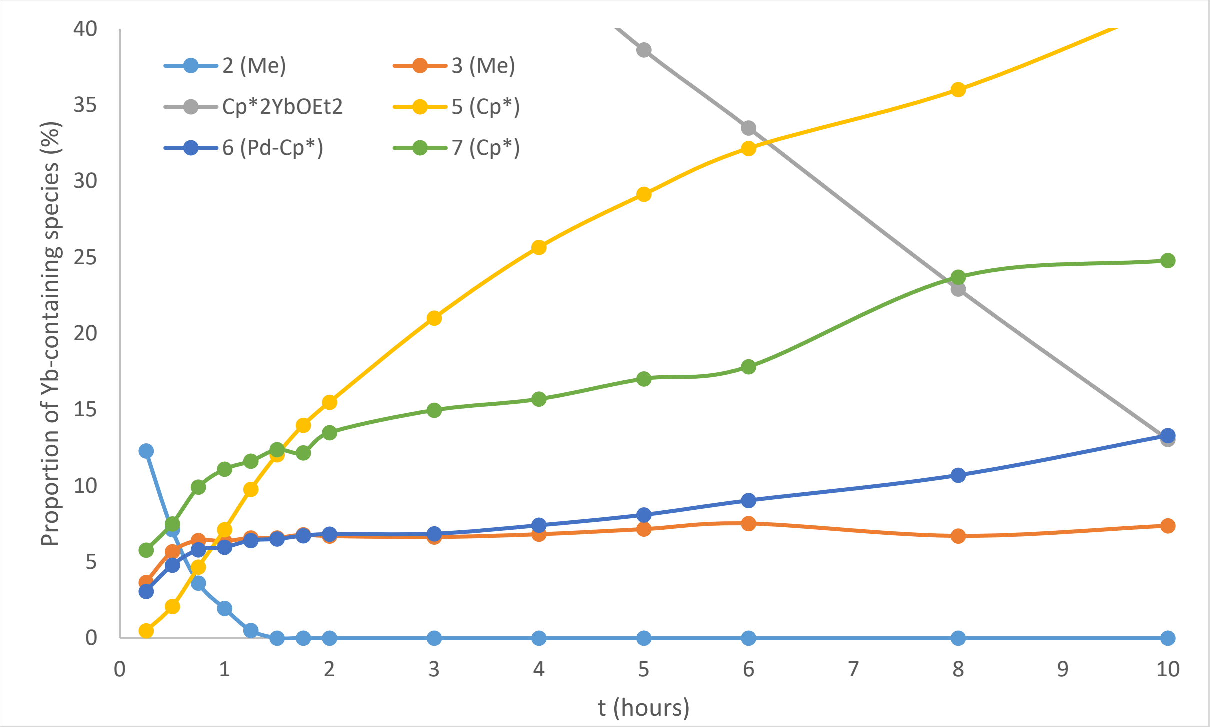

Figure S8. *In situ qualitative study of the addition of $Cp^*{}_2Yb(OEt_2)$ to* ***2.*** *For every compound, whenever possible, the signal with the cleanest integration was chosen. All the compounds were integrated with respect to an internal reference. Lines are only for guiding the eye.*

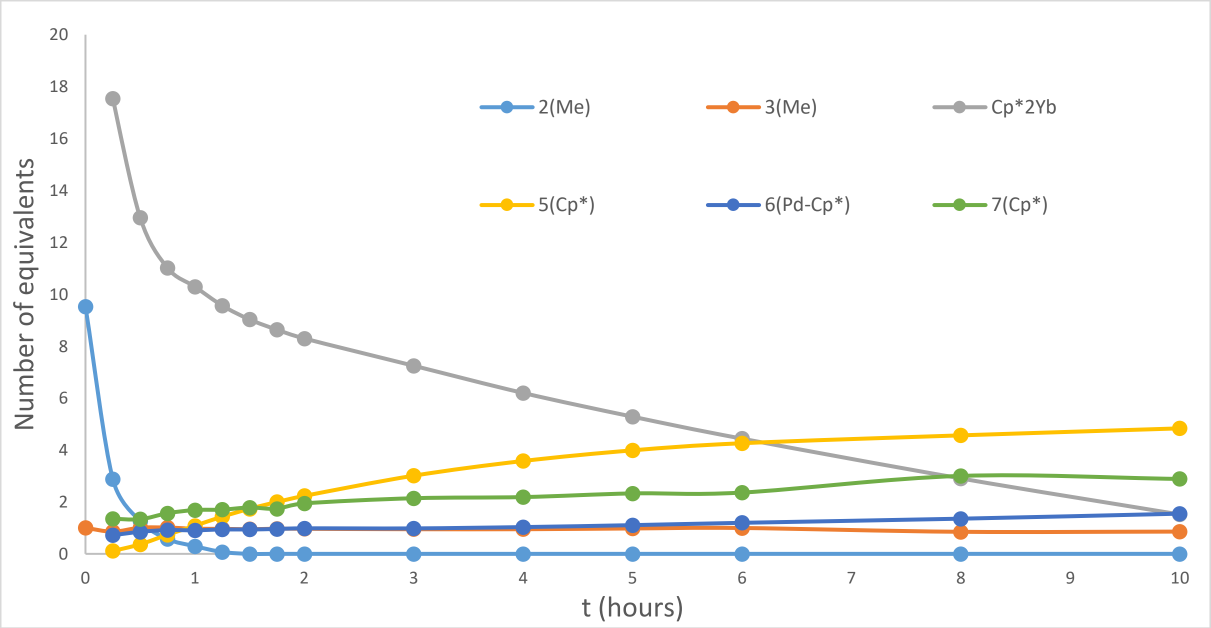

*Kinetic study of the reaction between **2** and an excess of ytterbocene in tol-d8. t_0 is the scan prior to the addition of ytterbocene, included to show the relative proportions of **2** and **3** in the initial mixture. For every compound, whenever possible, the signal with the cleanest integration was chosen. All the compounds were integrated with respect to an internal reference. Lines are only for guiding the eye.*

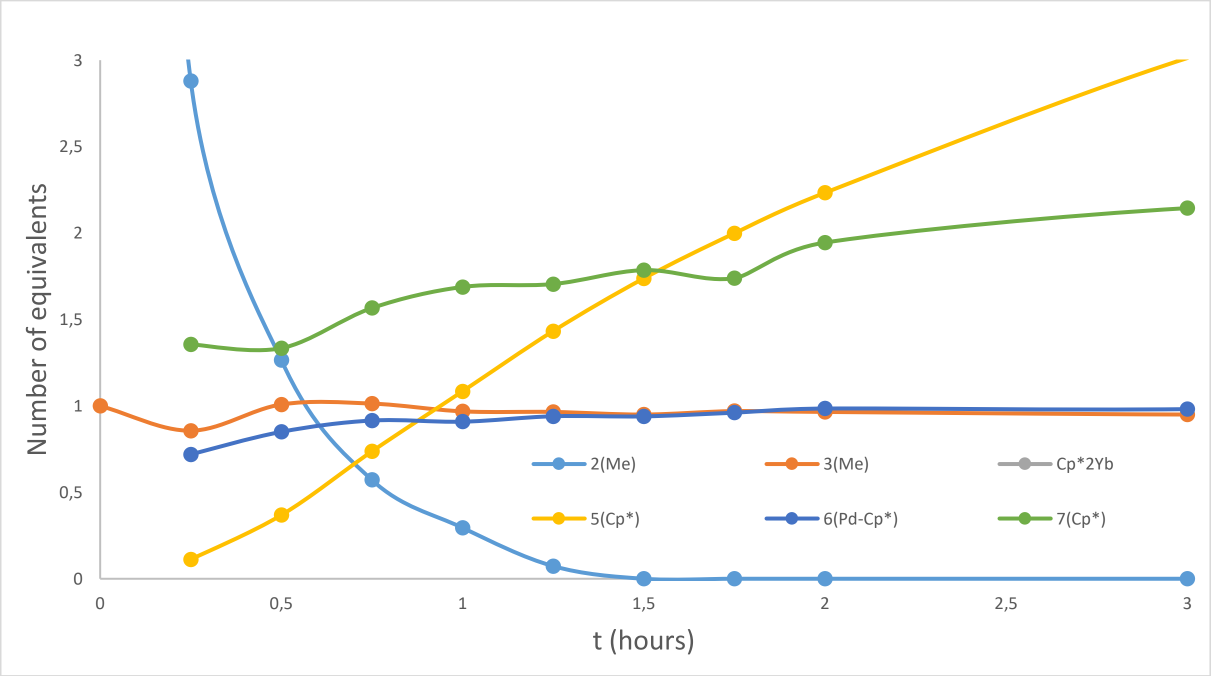

*Kinetic study of the reaction between **2** and an excess of ytterbocene in tol-d_8 – zoom into the evolution for the first three hours. t_0 is the scan prior to the addition of ytterbocene, included to show the relative proportions of **2** and **3** in the initial mixture. For every compound, whenever possible, the signal with the cleanest integration was chosen. All the compounds were integrated with respect to an internal reference. Lines are only for guiding the eye.*

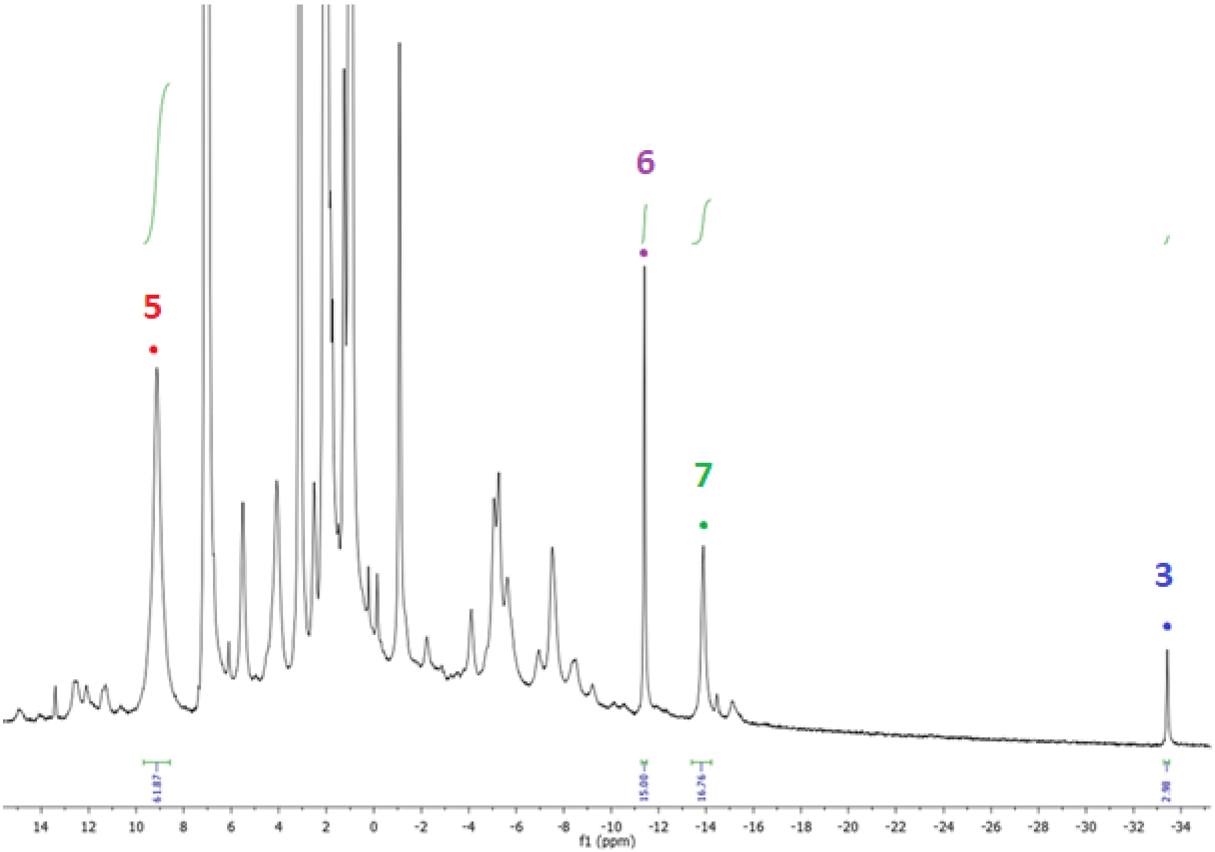

Figure S9. *In situ qualitative study of the addition of Cp*$_2$Yb(OEt$_2$) to **3*** after two weeks. *The signals consistent with the formation of **5**, **6** and **7** are observed. Note that after two weeks, not all the starting material is consumed.*

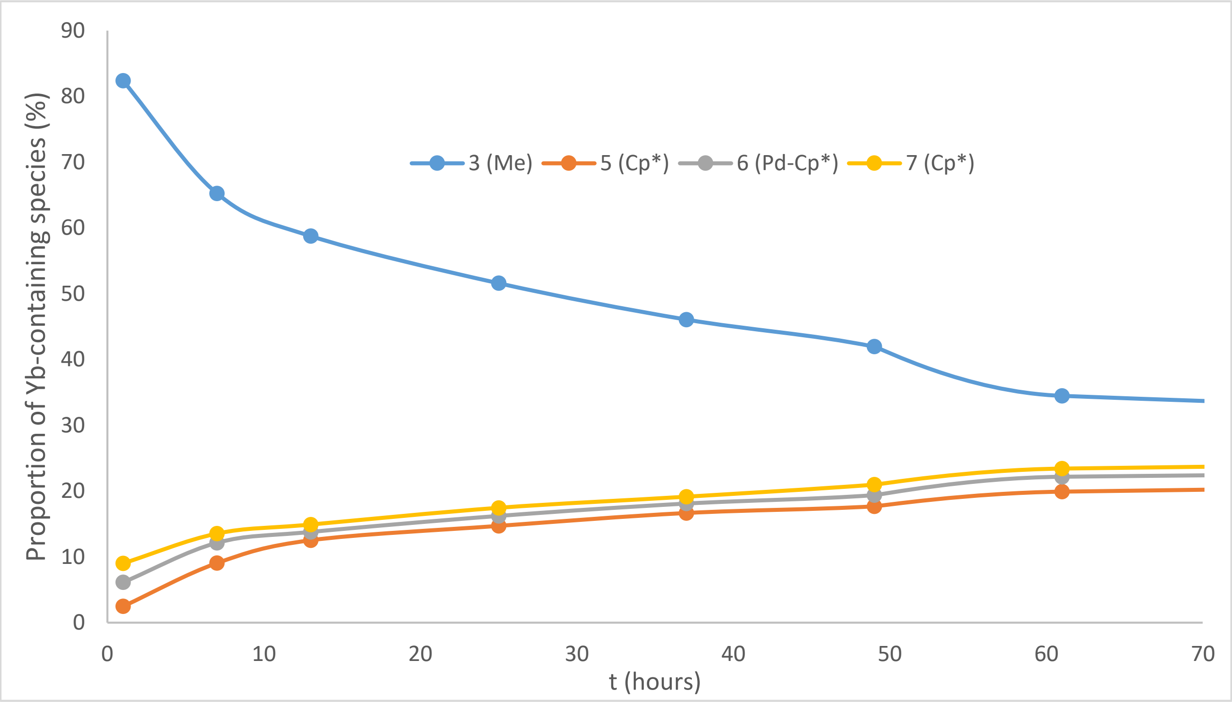

Figure S10 *: In-situ qualitative study of the addition of Cp*$_2$Yb(OEt$_2$) to* ***3.*** *All the compounds were integrated with respect to an internal reference. Lines are only for guiding the eye. Due to the lesser solubility of the compound, a reliable integration of the Cp*$_2$Yb(OEt$_2$) was not possible.*

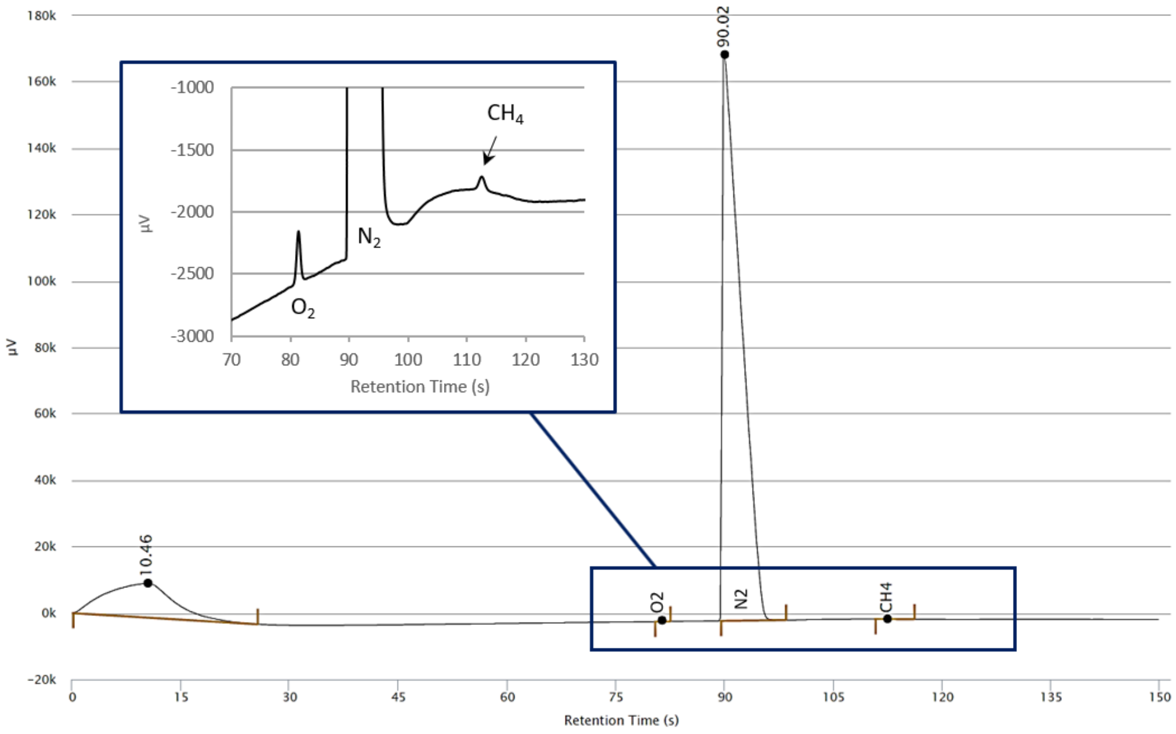

Figure S11 *: Gas evolution GC analysis by addition of Cp*$_2$Yb(OEt$_2$) to* ***3.***

3. Complex **4**

a) NMR of compounds

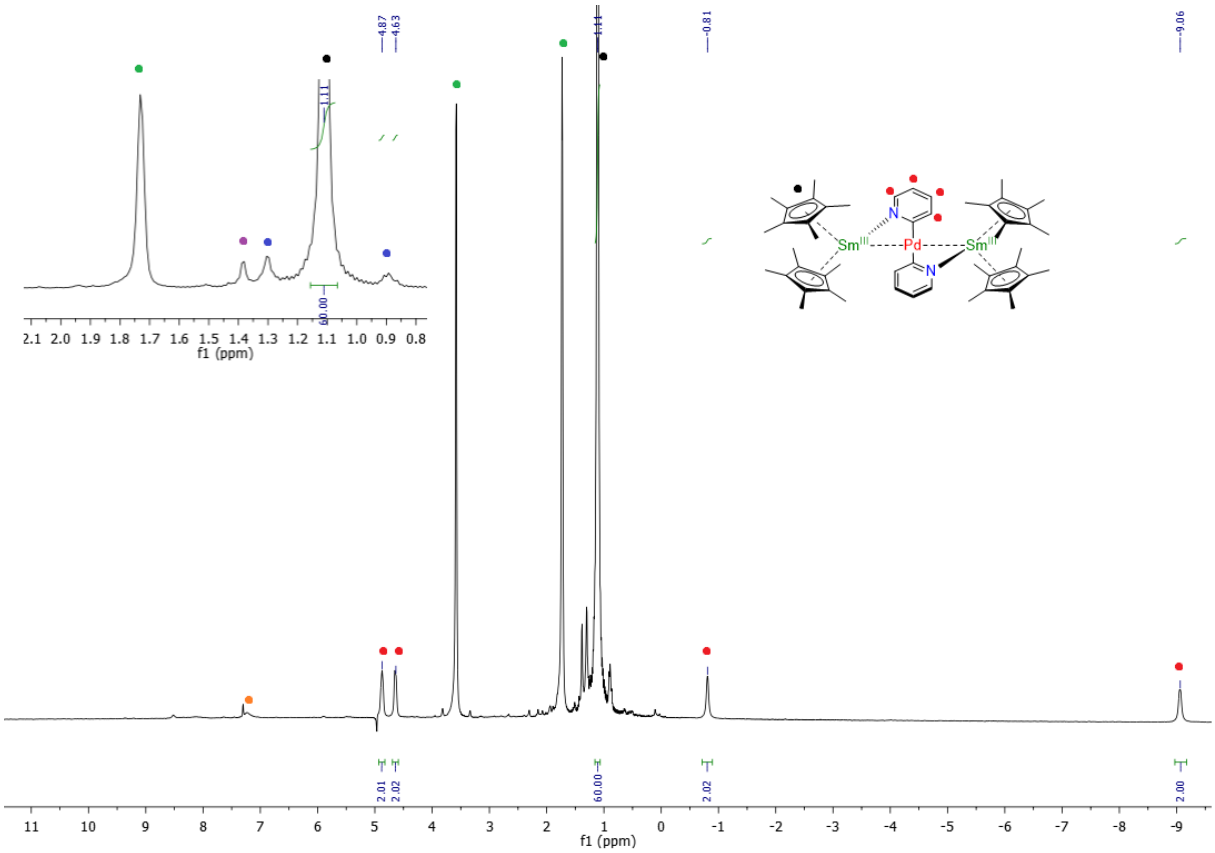

Figure S12. *^{1}H NMR of* ***4*** *in THF-d$_8$ at 293 K. The pyridyl signals are marked by red dots, the Cp* signal by a black dot. The trace impurities have the following colours: residual protio solvent signals – green, n-pentane – blue, toluene – orange, unknown impurity – purple.*

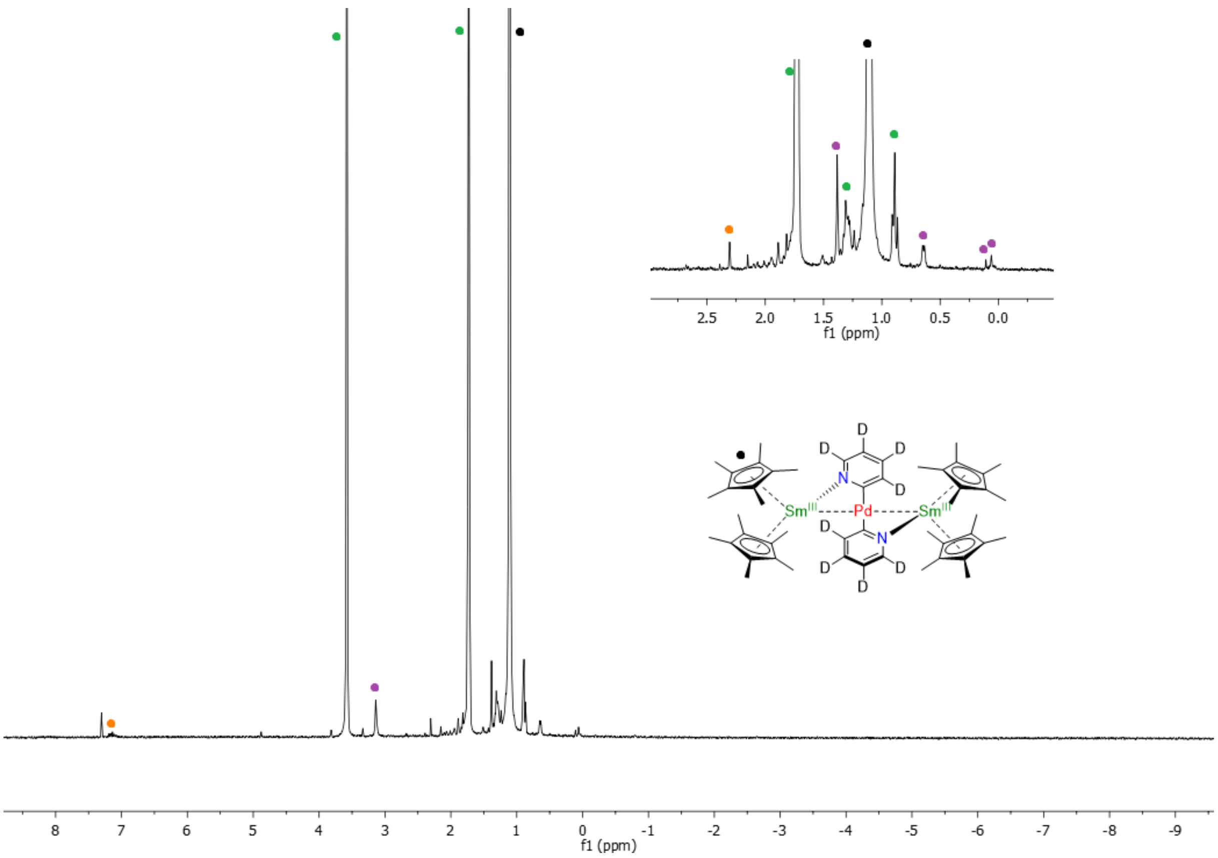

Figure S13. *^{1}H NMR of the complex **4-py-d₄** in THF-d₈ at 293 K. The Cp* signal is marked by a black dot. The trace impurities have the following colours: residual protio solvent signals – green, n-pentane – blue, toluene – orange, silicon grease – light brown, unknown impurities – purple.*

b) Variable temperature NMR of complex **4**

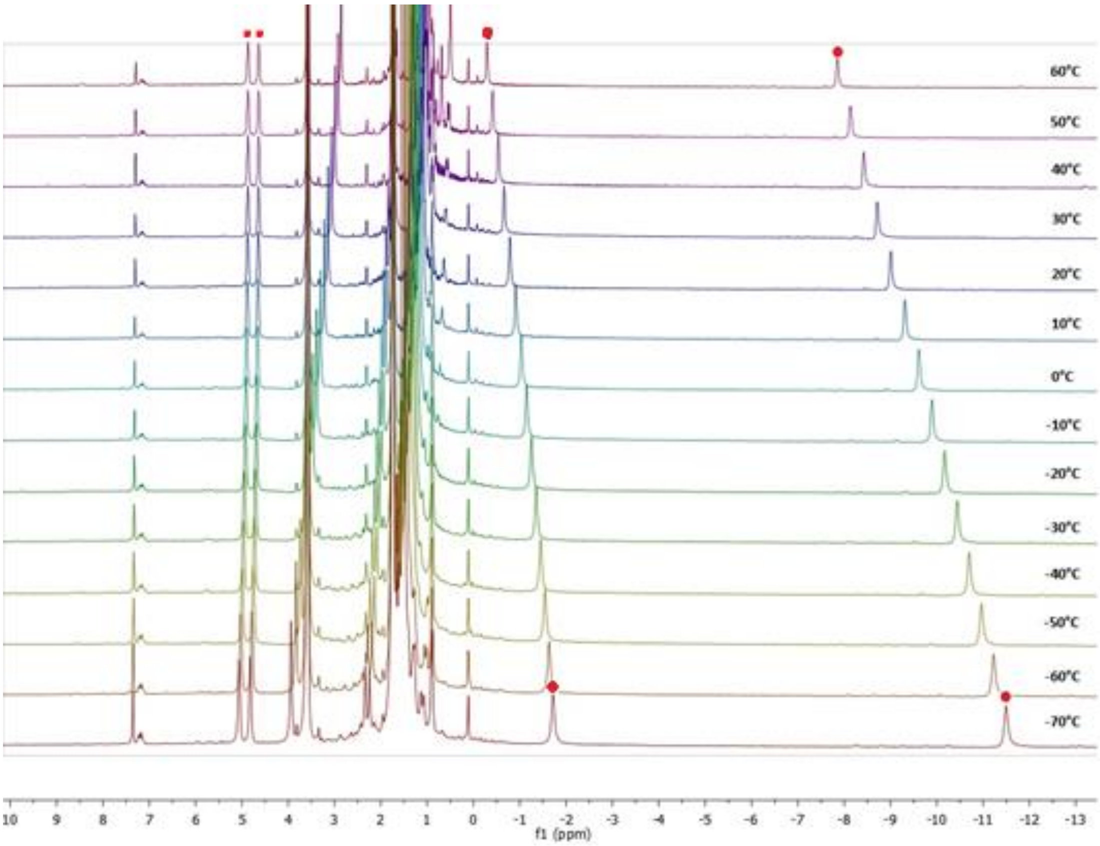

Figure S14. *Variable temperature ^1H NMR of **4** in THF-d_8. Pyridyl peaks are marked by red dots.*

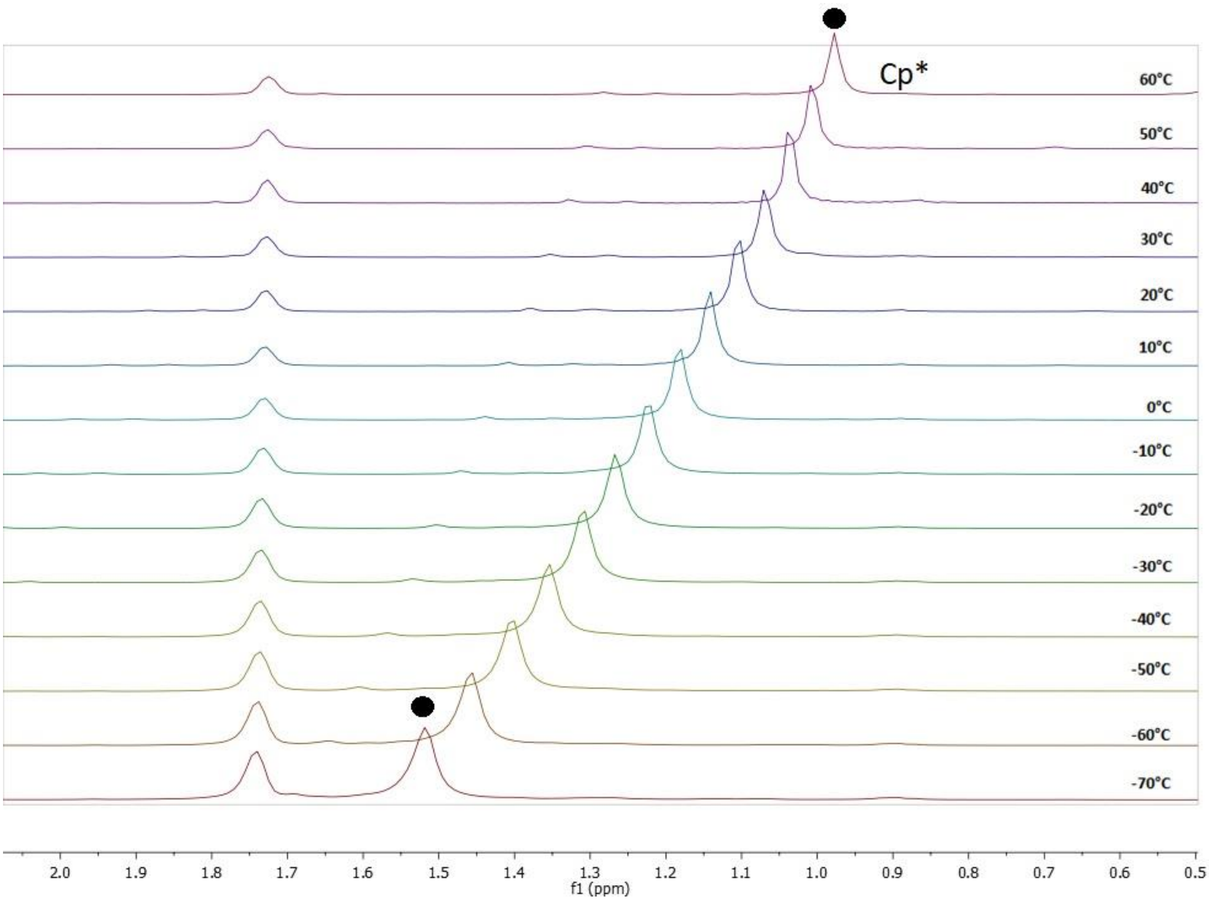

Figure S15. *Variable temperature ^{1}H NMR of **4** in THF-d_8 – Following of the Cp* peak.*

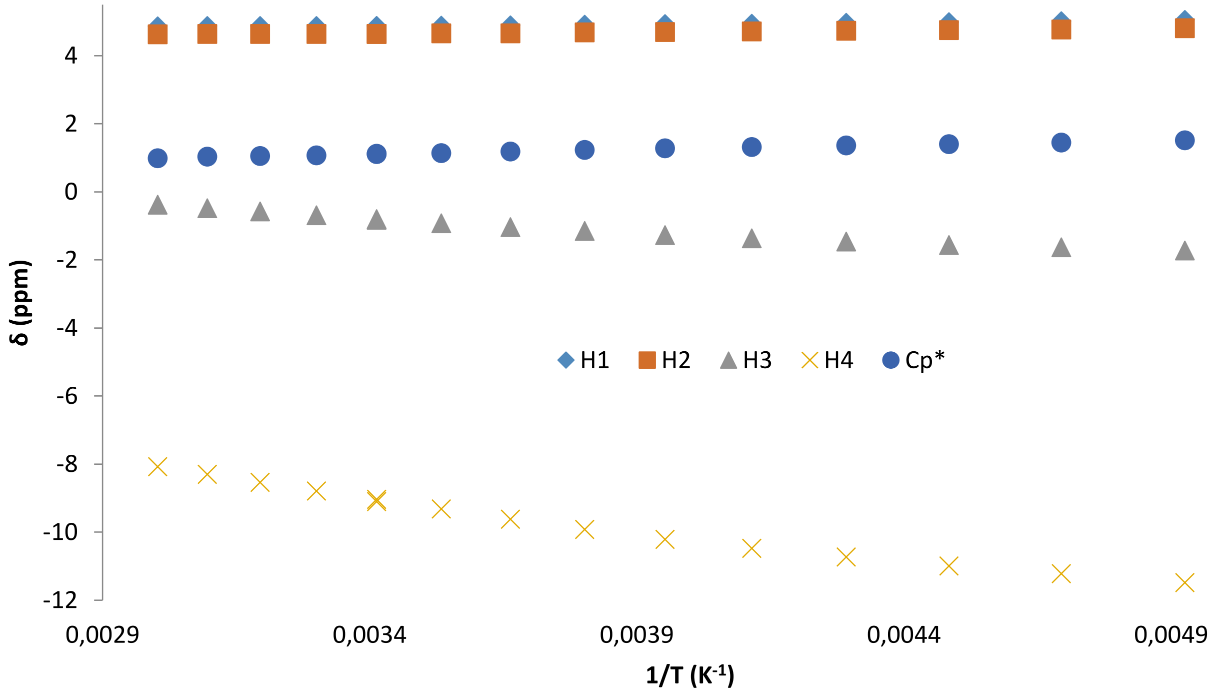

Figure S16. *δ vs 1/T plot for **4** in THF-d_8. H1-H4 indicate the protons on the pyridyl.*

c) Formation reactions of compound **4**

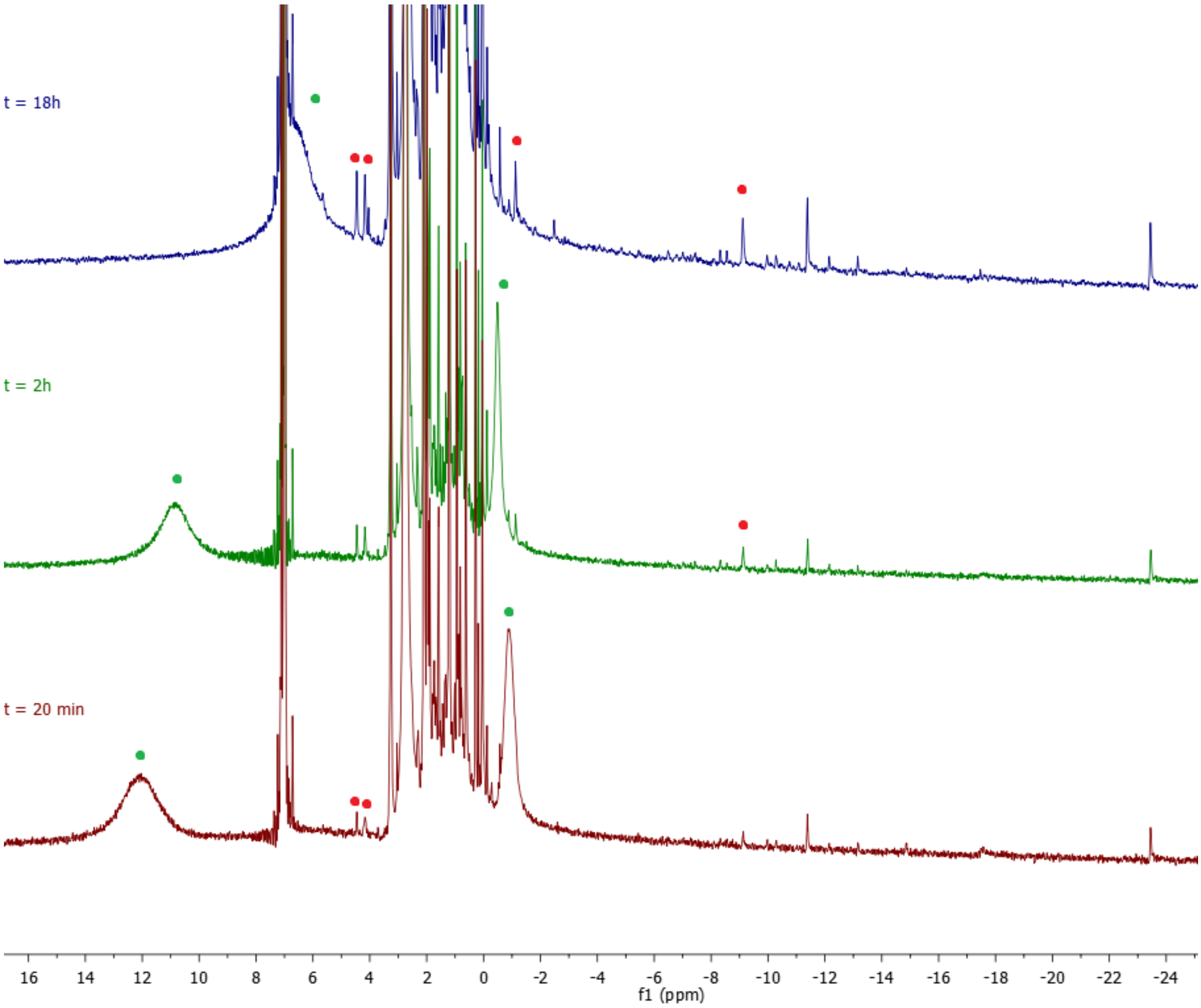

Figure S17. *In situ formation of **4** using **two equivalents** of $Cp^*_2SmOEt_2$ added to **1** in tol-d_8. Peaks corresponding to **4** are formed within a few minutes. Pyridyl peaks are marked by red dots, $Cp^*_2SmOEt_2$ peaks are marked by green dots.*

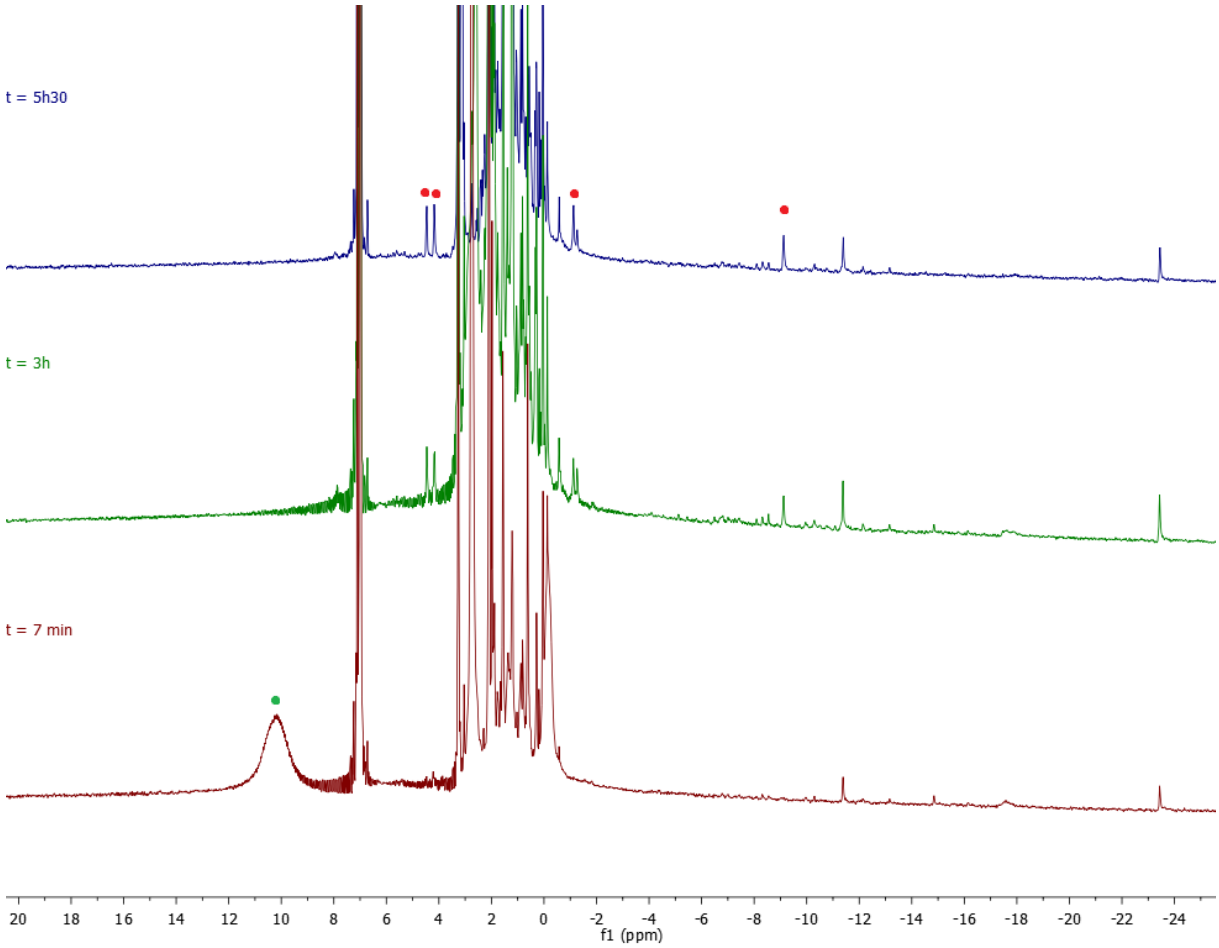

Figure S18. *In situ formation of **4** using **one equivalent** of Cp*$_2$SmOEt$_2$ added to **1** in tol-d$_8$. Peaks corresponding to **4** are formed within 3 hours. Pyridyl peaks are marked by red dots, Cp*$_2$SmOEt$_2$ peaks are marked by green dots.*

4. Complex **5**

a) NMR of compounds

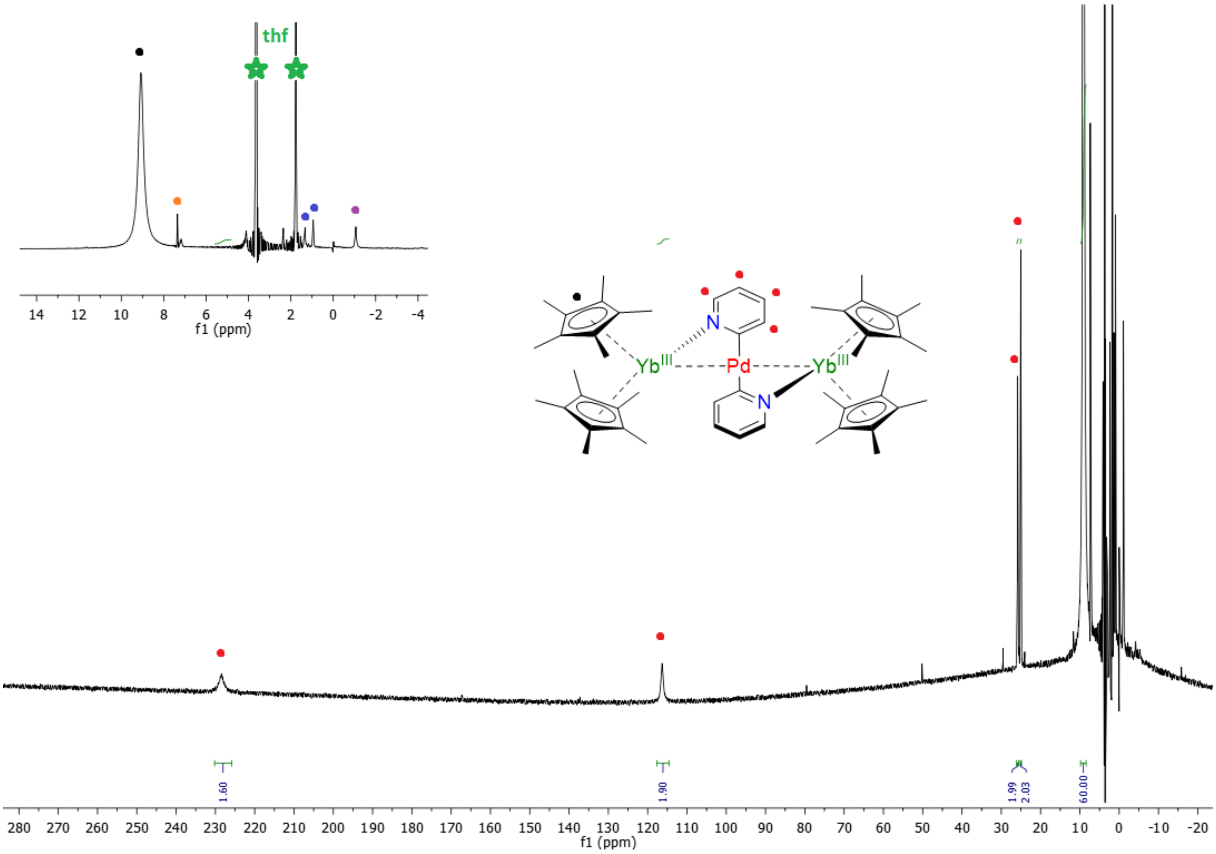

Figure S19. *^{1}H NMR of complex **5** in THF-d_8 at 293 K. Red dots indicate the pyridyl peaks, the black dot indicates the Cp* peak, the blue peak indicates H grease, the orange dot residual toluene and the purple peak indicates an unknown impurity.*

b) Variable temperature NMR of complex **5**

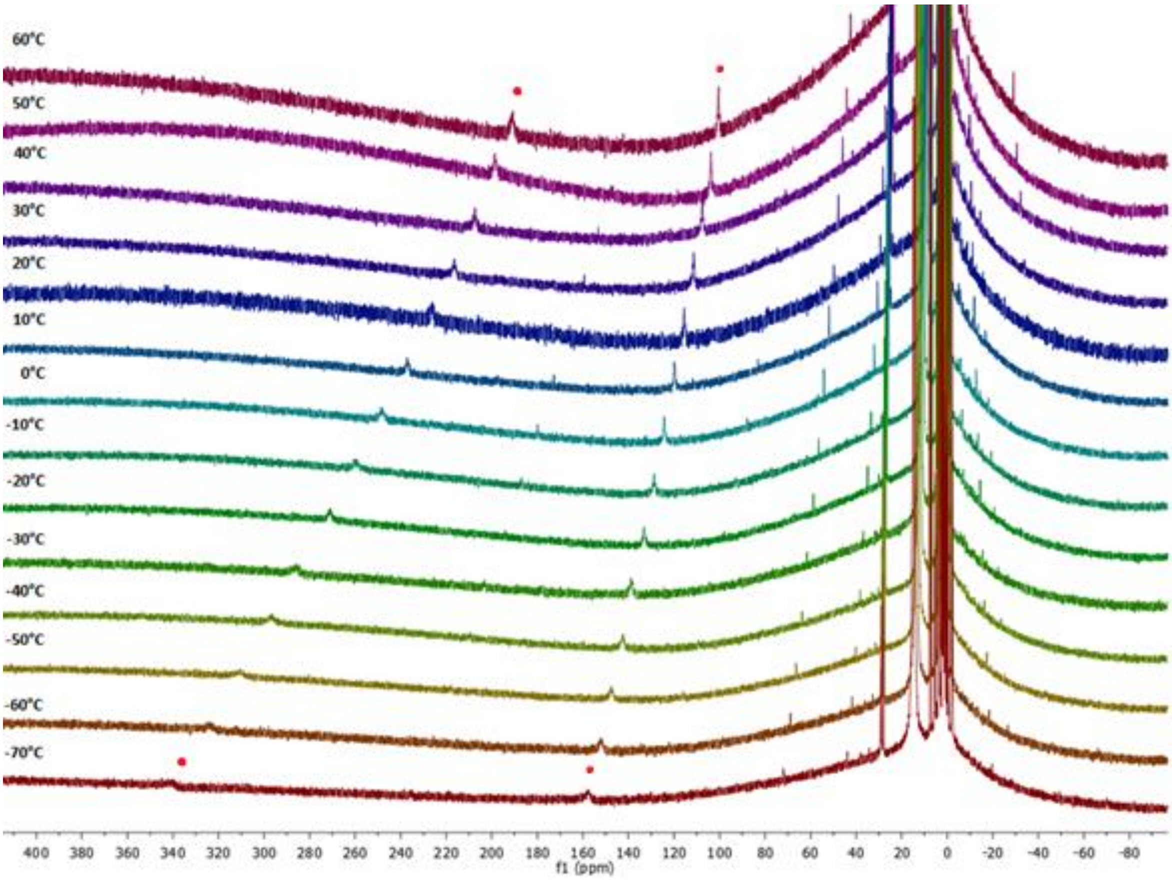

***Figure S20**. Variable temperature ¹H NMR of **5** in THF-d$_8$. Red dots correspond to the pyridyl.*

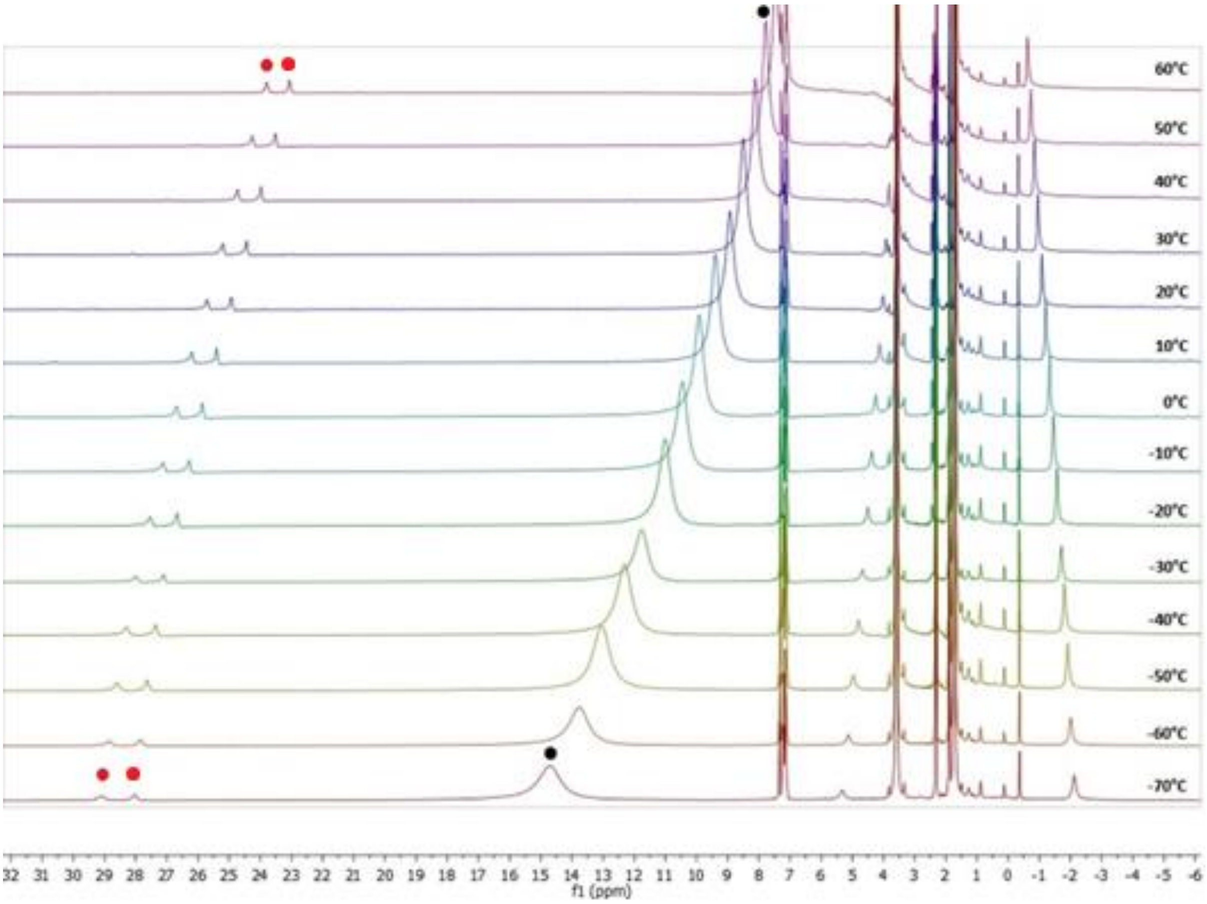

Figure S21. *Variable temperature ^{1}H NMR of **5**. Zoom in the -6 to +32 ppm range. Red dots correspond to the pyridyl and the black dot corresponds to the Cp*.*

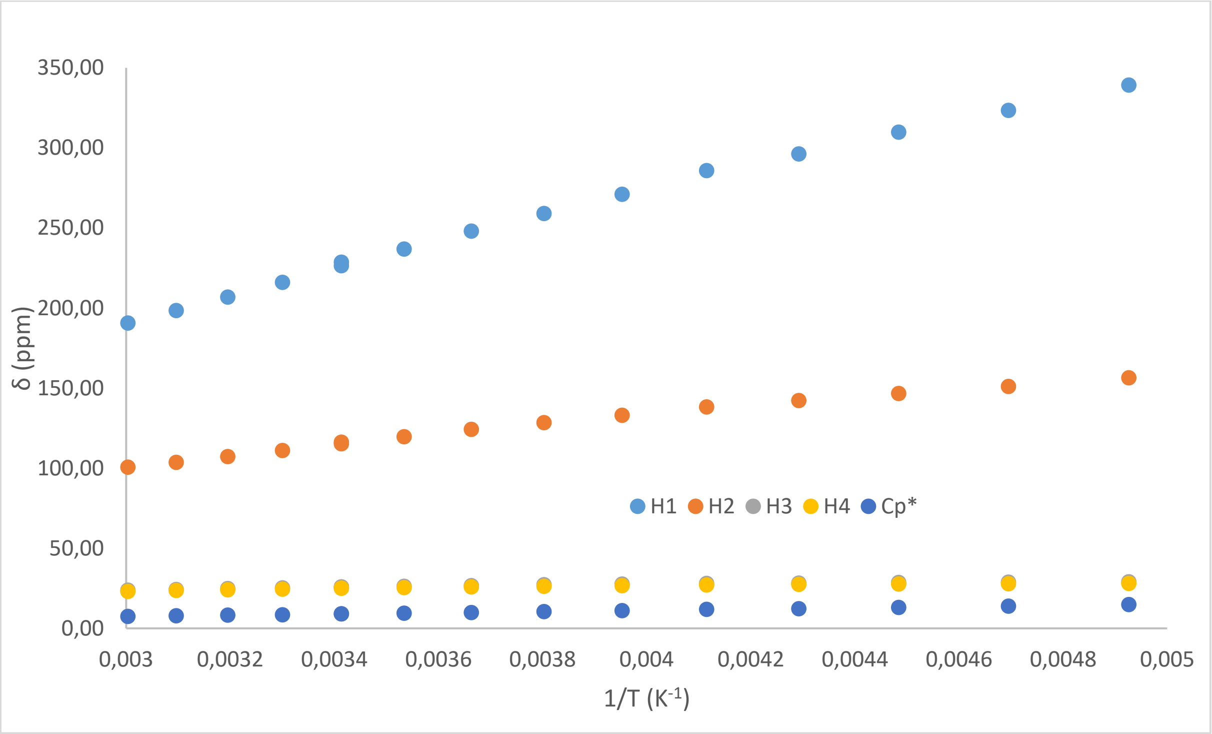

Figure S22*. δ vs 1/T plot for **5** in THF-d_8. H1-H4 indicate the protons on the pyridyl.*

c) *In situ* formation reactions of **5**

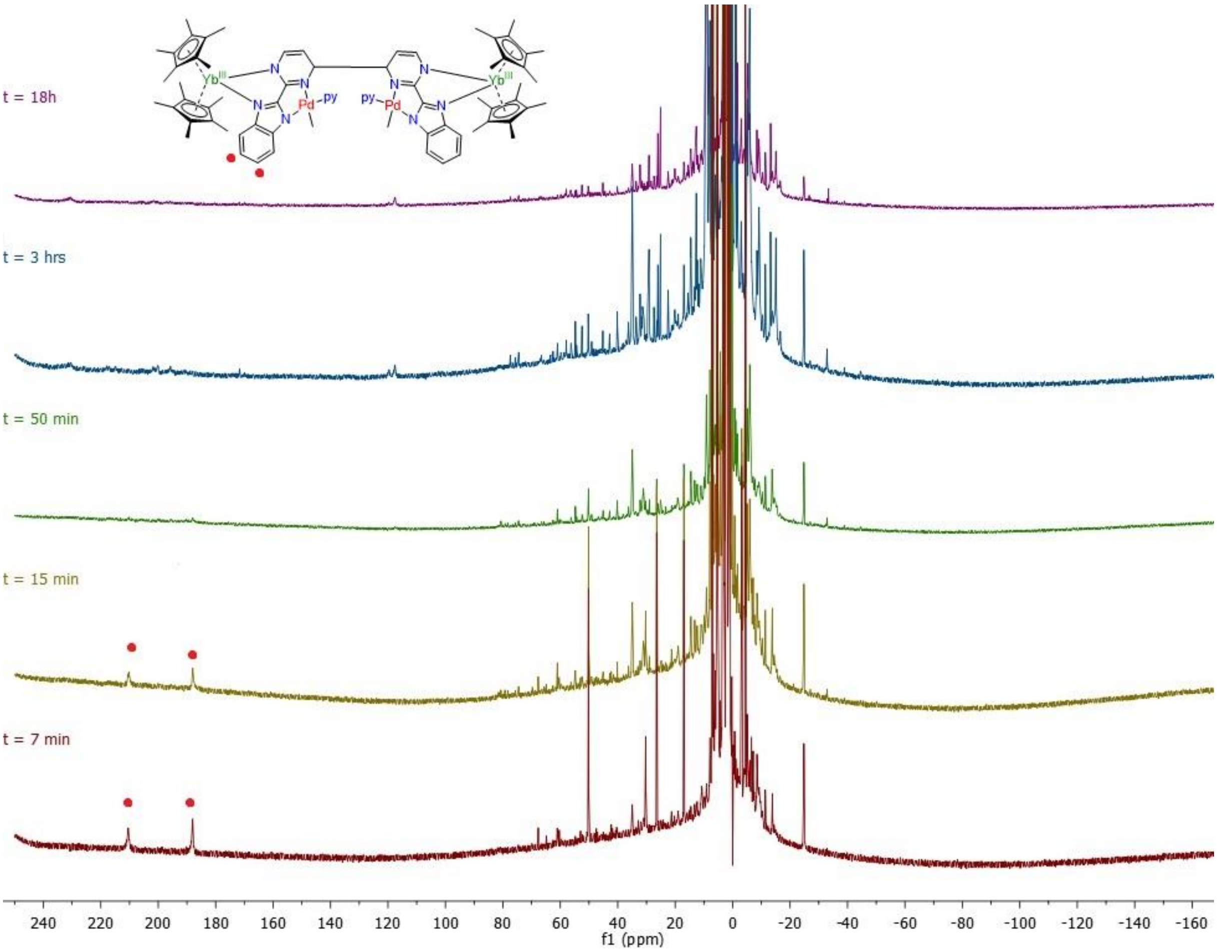

Figure S23*. In situ formation of* ***5*** *using* ***one equivalent*** *of $Cp^*{}_2YbOEt_2$ added to* ***1*** *in tol-d_8 at 293 K over different periods of time.*

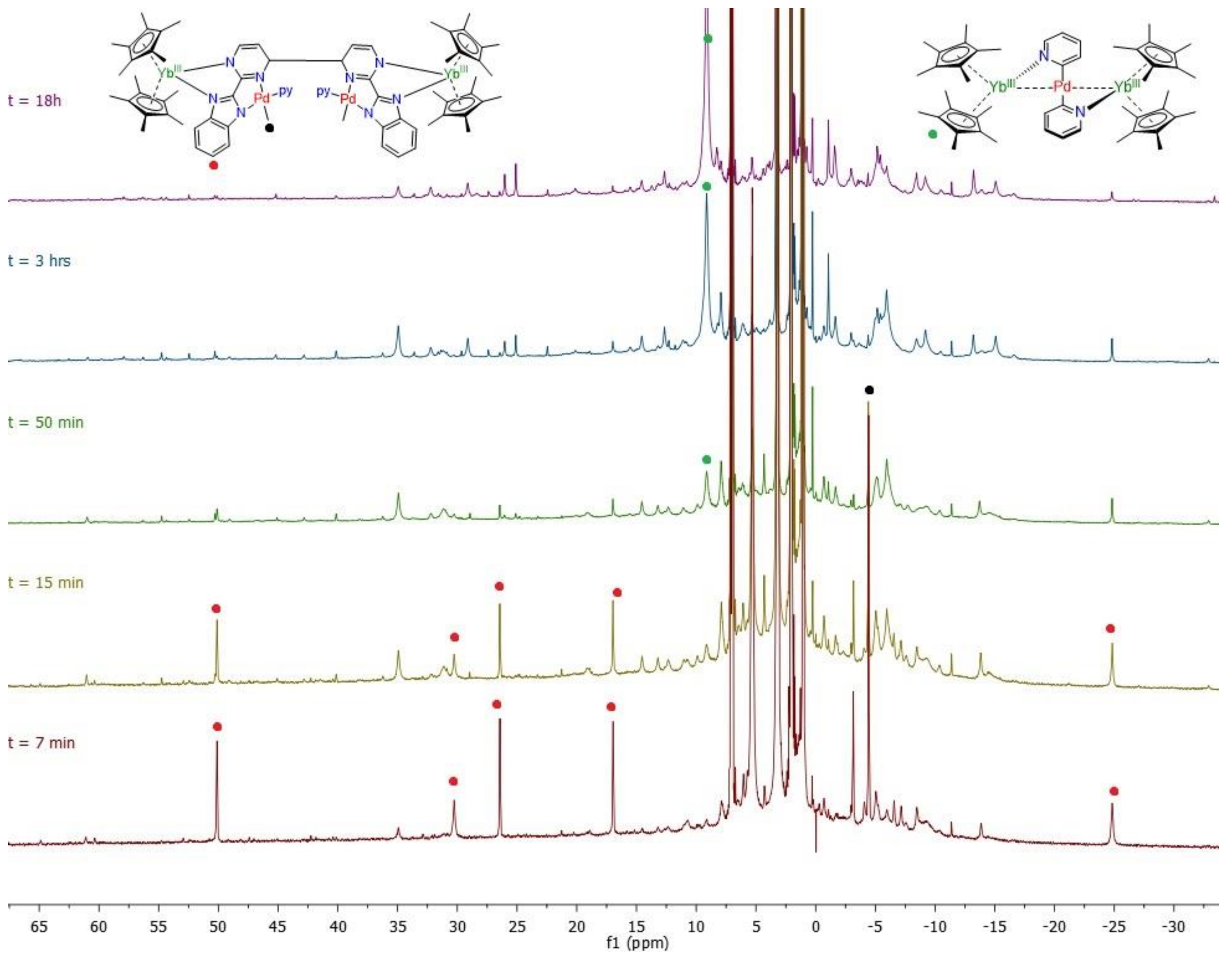

Figure S24. *In situ formation of **5** using **one equivalent** of $Cp^*_2YbOEt_2$ added to **1** in tol-d_8 at 293 K over different periods of time. Zoom in the -30 to +65 ppm range.*

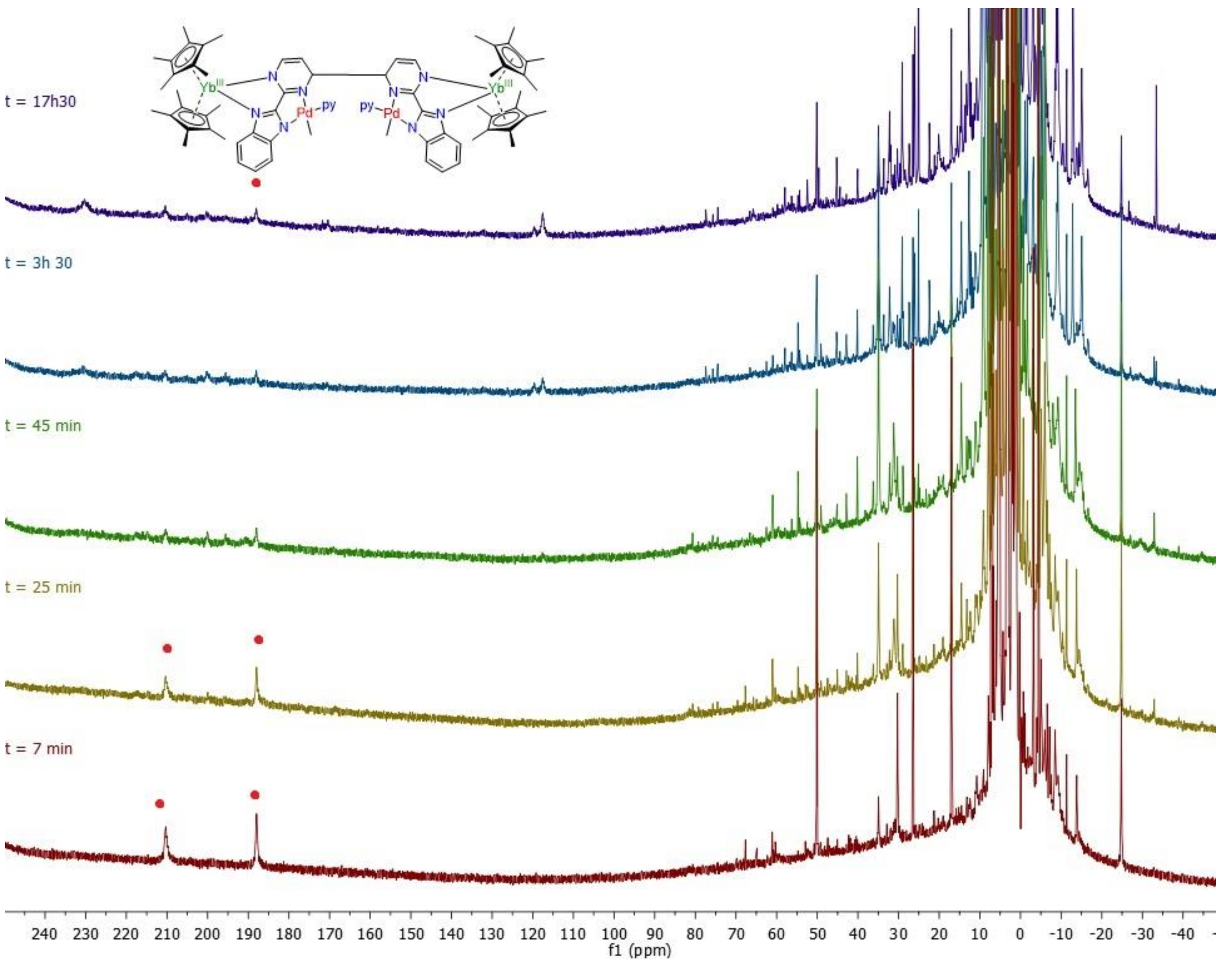

Figure S25. *In situ formation of **5** using **two equivalents** of Cp*$_2$YbOEt$_2$ added to **1** in tol-d$_8$ at 293 K over different periods of time.*

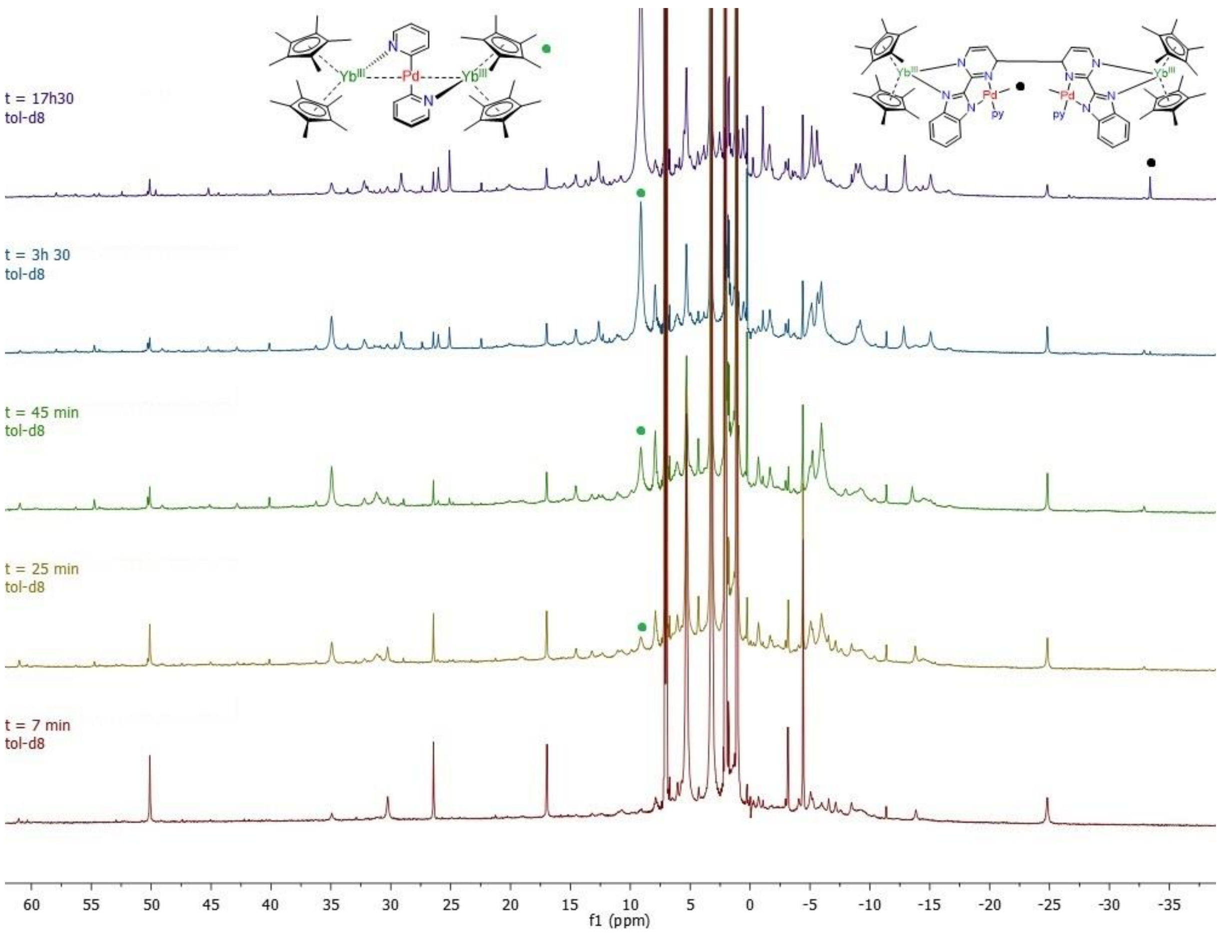

Figure S26. *In situ formation of **5** using **two equivalents** of* $Cp^*_2YbOEt_2$ *added to **1** in tol-*d_8 *at 293 K over different periods of time. Zoom in the -35 to +60 ppm range.*

5. Compound **6**

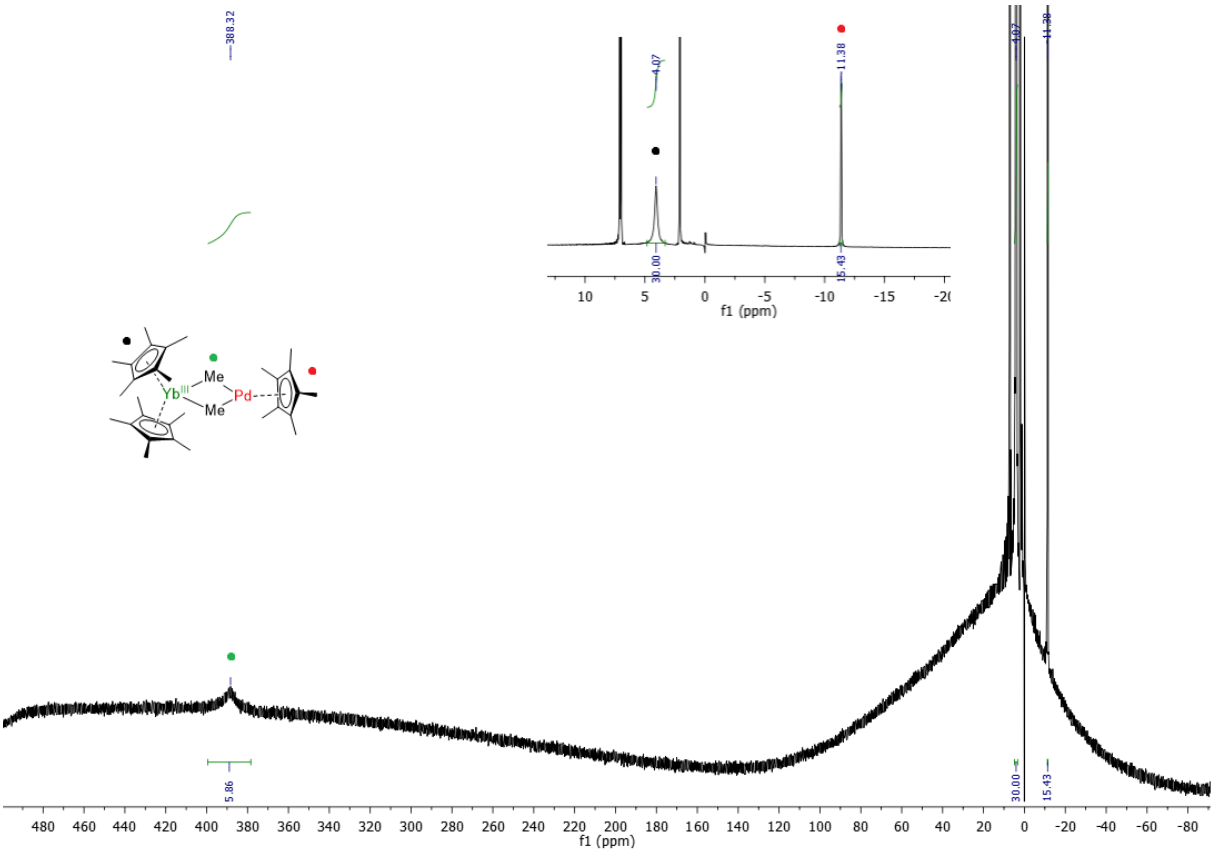

Figure S27. *^{1}H NMR of complex **6** in tol-d_8 at 293 K.*

III. Magnetism

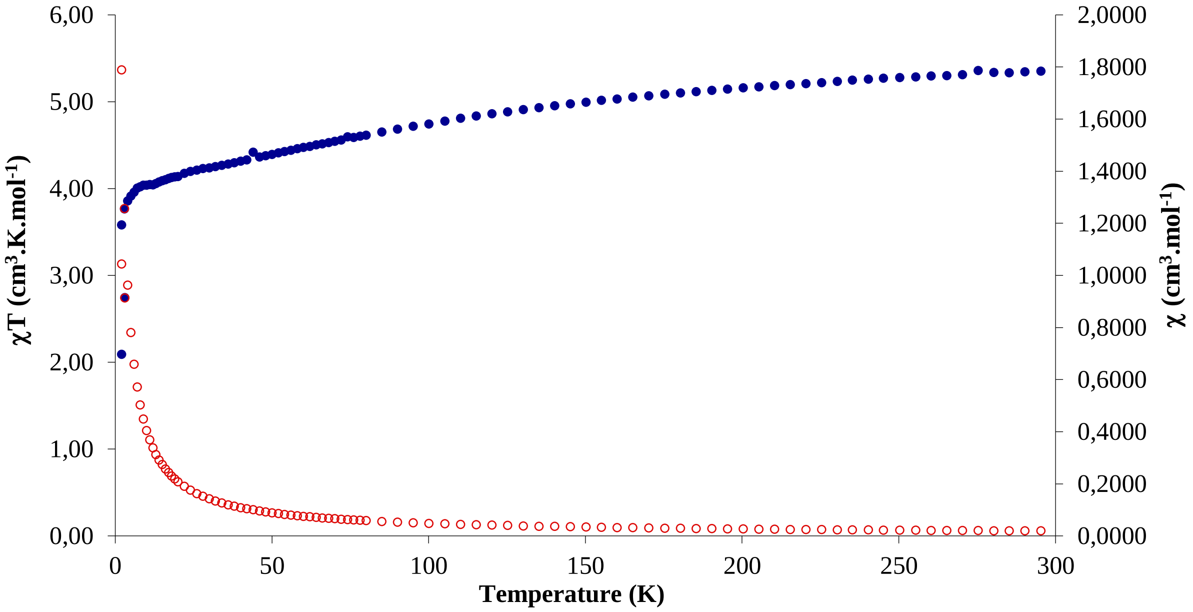

Figure S28. *Temperature-dependent magnetic data for* ***5****. χ vs T is given as unfilled red dots, χT vs. T as filled blue dots.*

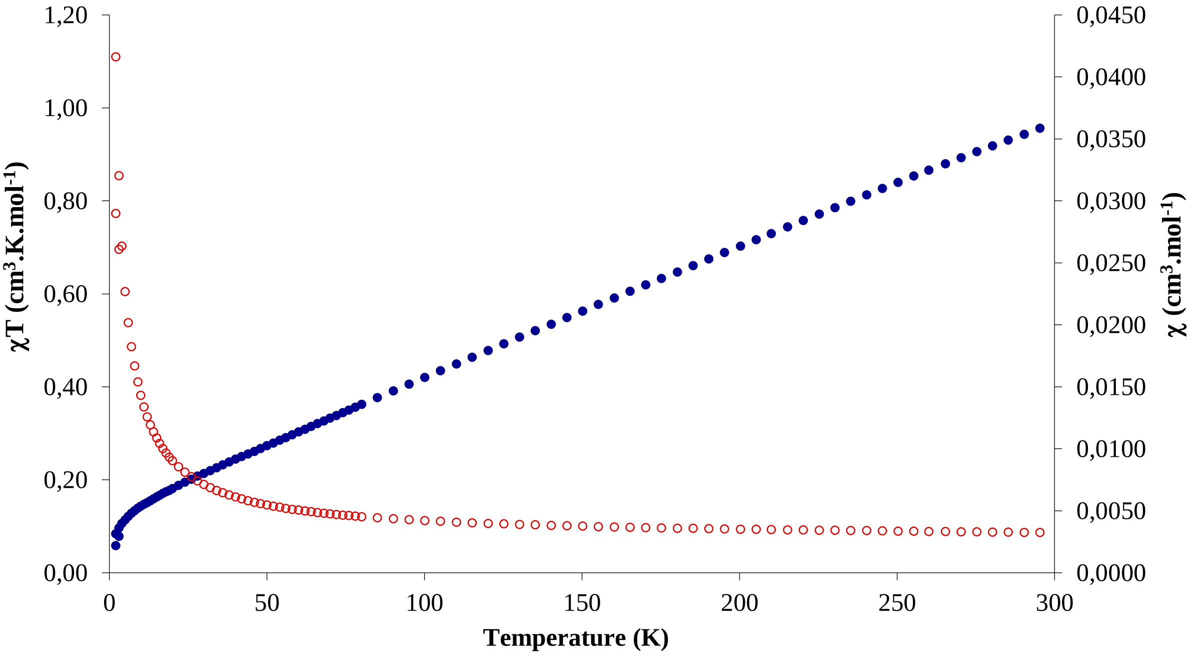

Figure S29. *Temperature-dependent magnetic data for* ***4****. χ vs T is given as unfilled red dots, χT vs. T as filled blue dots.*

IV. Crystallographic data

Single crystals were mounted on a Kapton loop using Paratone N oil. An APEX III CCD BRUKER detector and a graphite Mo-Kα monochromator were used for the data acquisition. All measurements were done at 150 K and a refinement method was used for solving the structures. The structure resolution was accomplished using the SHELXS-97 and SHELXT[5] programs, and the refinement was done with the SHELXL program.[6,7] The structure solutions and the refinements were achieved using the Olex2 software.[8] Pictures of the molecular structures in the solid state were obtained using the MERCURY software.[9] During the refinement steps, all atoms except hydrogens were refined anisotropically. The hydrogen atoms were placed on calculated positions, riding on the parent atom, unless otherwise stated. Finally, in order to obtain a complete refinement, a weighting step followed by multiple loops of refinement was done.

Specific comments for each data set are given below. Summary of the crystal data, data collection and refinement for the different complexes is given in Tables S1. Crystallographic data for the structures reported in this paper have been deposited with the Cambridge Crystallographic Data Centre (CCDC #2215195-2215200).

The following special comments apply to the models of the structures:

- Complex **2** crystallizes with 3 disordered molecules of toluene in the asymmetric unit. The four Cp* rings are disordered over two positions with freely refined occupancy ratios. For the disordered fragments, similarity restraints on the displacement parameters (SIMU) and/or rigid body restraints (RIGU) were applied on the corresponding atoms.
  An alert B (11 missing FCF reflections below Theta(Min)) is arising in the CheckCif and might originate from low angle reflections that are missing due to the beamstop as the unit cell is reasonably large.

- Complex **3** crystallizes with 6 molecules of THF in the asymmetric unit. Two of them could be satisfactorily modelled using similarity restraints on the corresponding bond distances (SADI) and displacement parameters (SIMU). The four other are disordered over multiple positions and were removed from the electron density map using the Olex2 solvent mask command: 340 electrons were found in a volume of 864 Å^3 in 1 void per unit cell. This is consistent with the presence of 4 [C_4H_8O] per asymmetric unit which account for 320 electrons per unit cell.
  Two Alerts B are arising (R_{int} value of 0.195 > 0.18 and low C-C bond precision) which is due to the needle shape of the crystal and its very small dimensions resulting in very weak diffraction at high theta angles.

- The asymmetric unit of **4**·(2 C_7H_8) contains half a molecule of **4** and one molecule of toluene.

- The asymmetric unit of **5**·(2 C_7H_8) contains half a molecule of **5** and one molecule of toluene.
  An Alert A concerning a residual density peak of 3.65 e.Å^{-3} at 0.77 Å from the heavy Yb centre is present and may result from suboptimal absorption correction and Fourier truncation error.

- In the molecular structure of **6**, the $Pd(CH_3)_2$ protons have been located in the difference Fourier map and treated as riding on the corresponding parent carbon atoms.

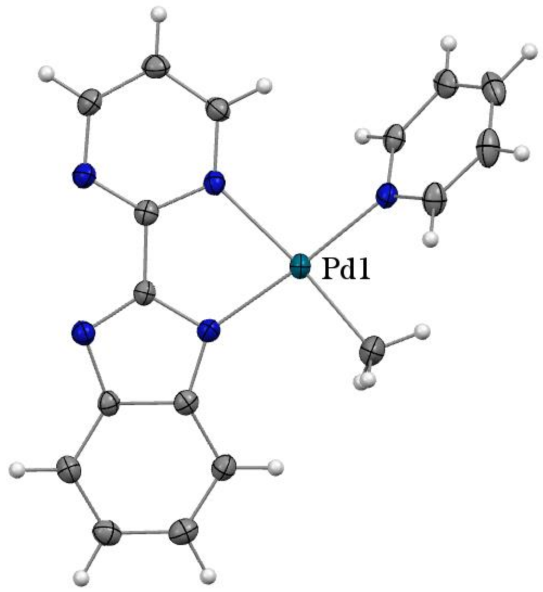

Figure S30. *ORTEP of* ***1*** *with 50% probability ellipsoids.*

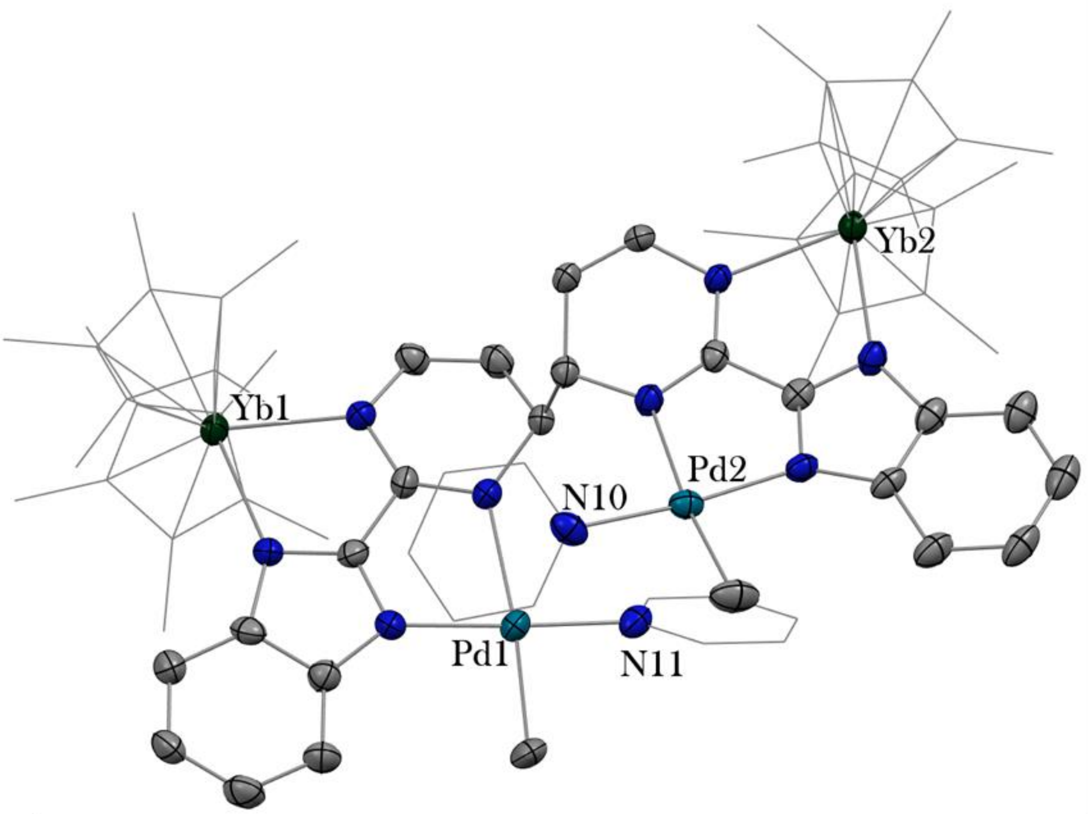

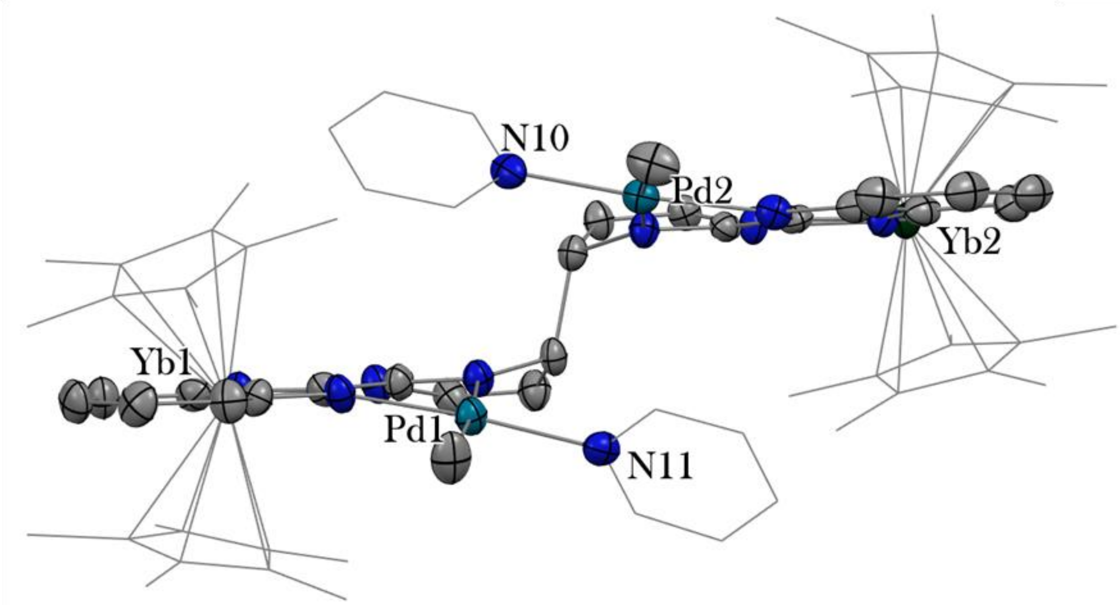

Figure S31. *ORTEP of* ***2*** *(view from top, above; side view, below) with 50% probability ellipsoids (hydrogen atoms have been removed for clarity). The Cp* and pyridine moieties are shown in wireframe to avoid clutter.*

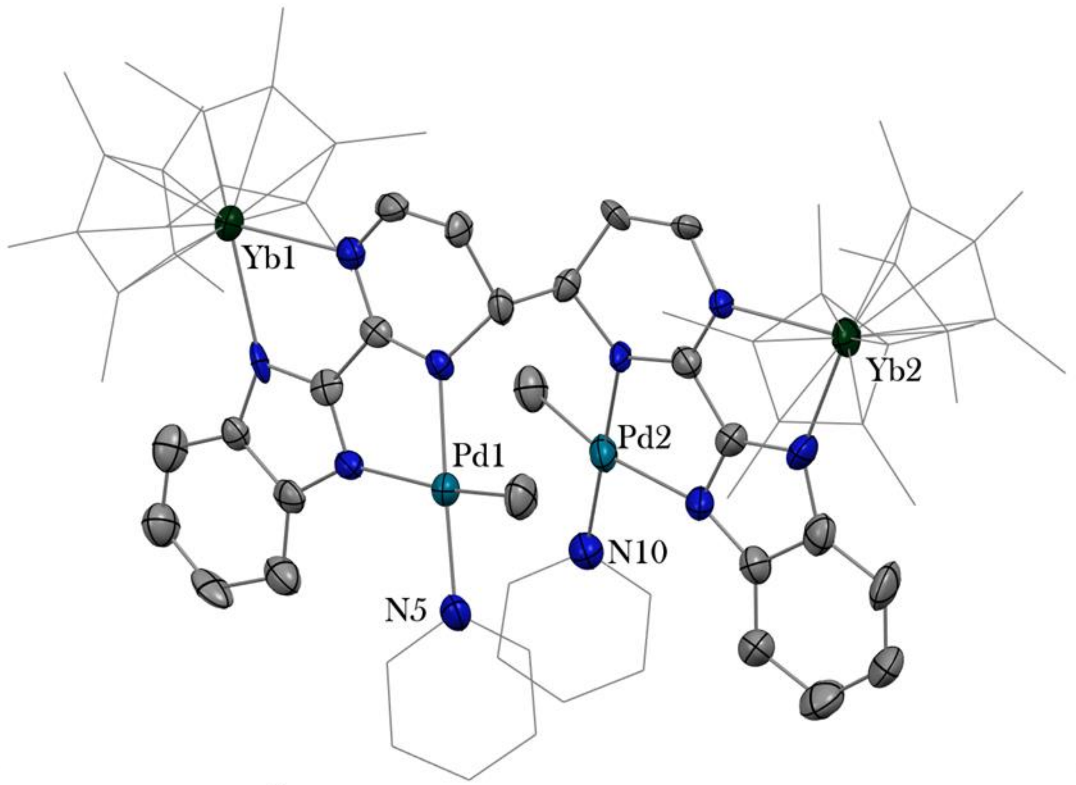

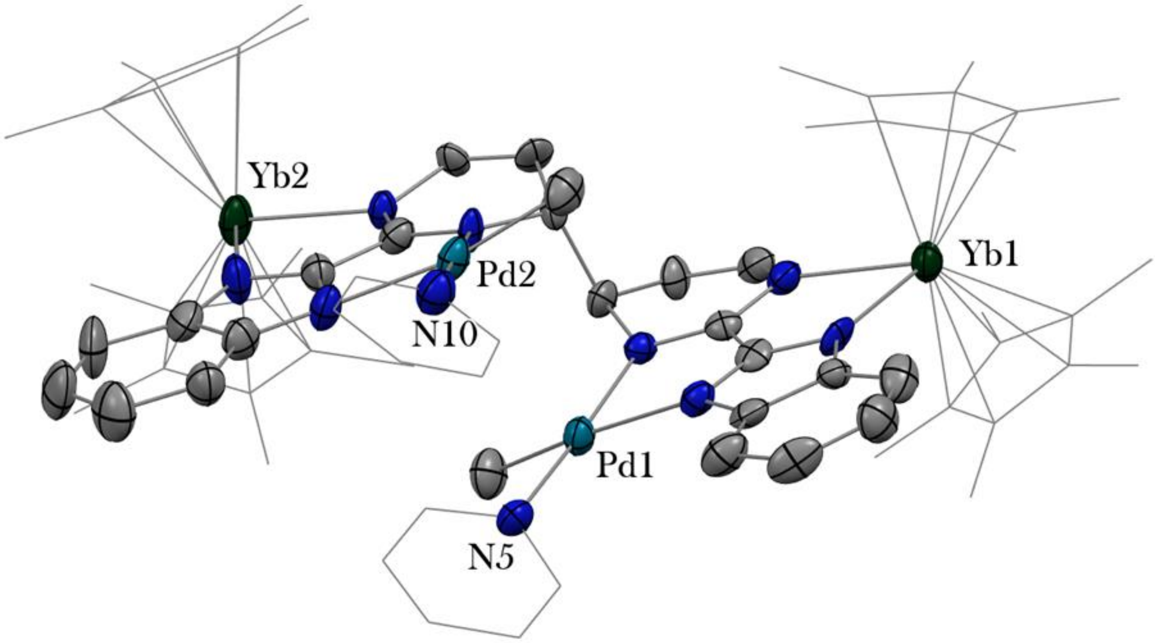

Figure S32. *ORTEP of* ***3*** *(view from top, above; side view, below) with 50% probability ellipsoids (hydrogen atoms have been removed for clarity). The Cp* and pyridine moieties are shown in wireframe to avoid clutter.*

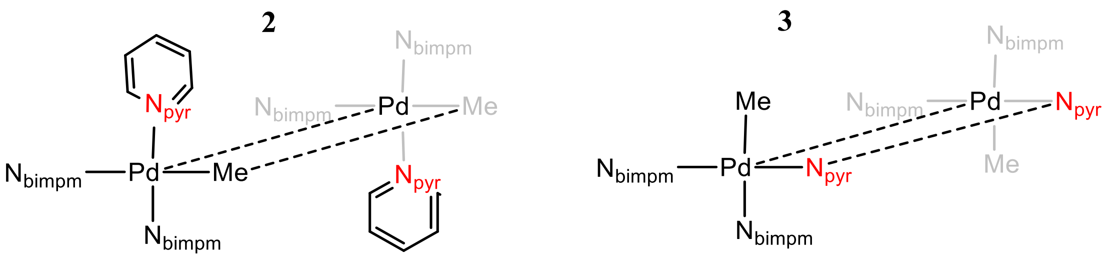

Figure S33. *Schematic representation of the relative orientations of the ligands in the two planes of the Pd coordination sphere in compounds **2** and **3**.*

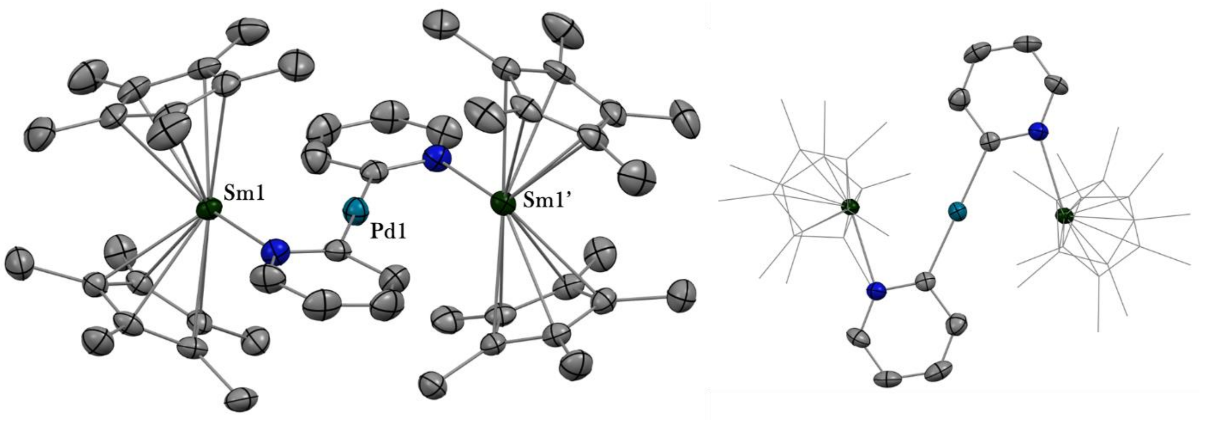

Figure S34. *ORTEP of **4** with 50% probability ellipsoids (hydrogen atoms have been removed for clarity). Atoms with the prime character in the atom labels (') are at equivalent position (1-x, y, 3/2-z).*

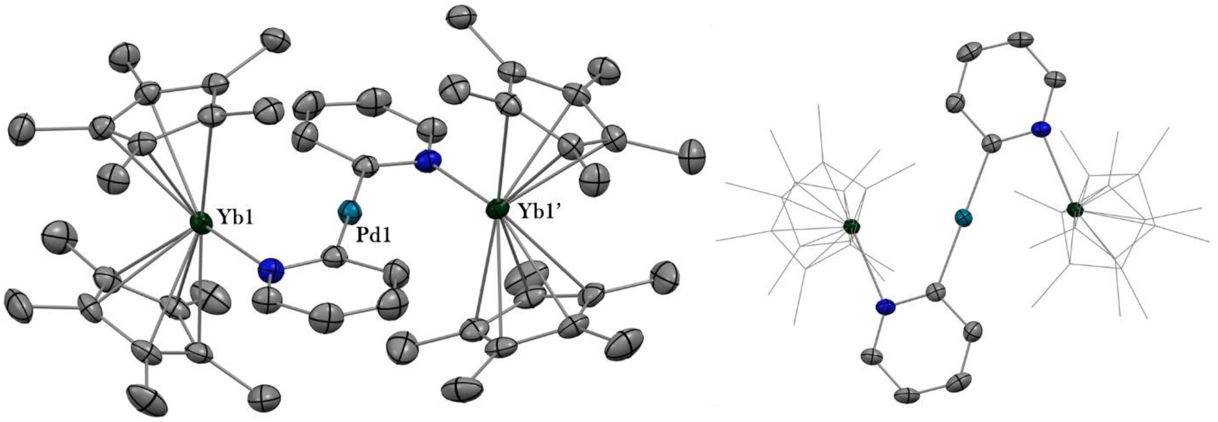

Figure S35. *ORTEP of **5** with 50% probability ellipsoids (hydrogen atoms have been removed for clarity). Atoms with the prime character in the atom labels (') are at equivalent position (1-x, y, 3/2-z).*

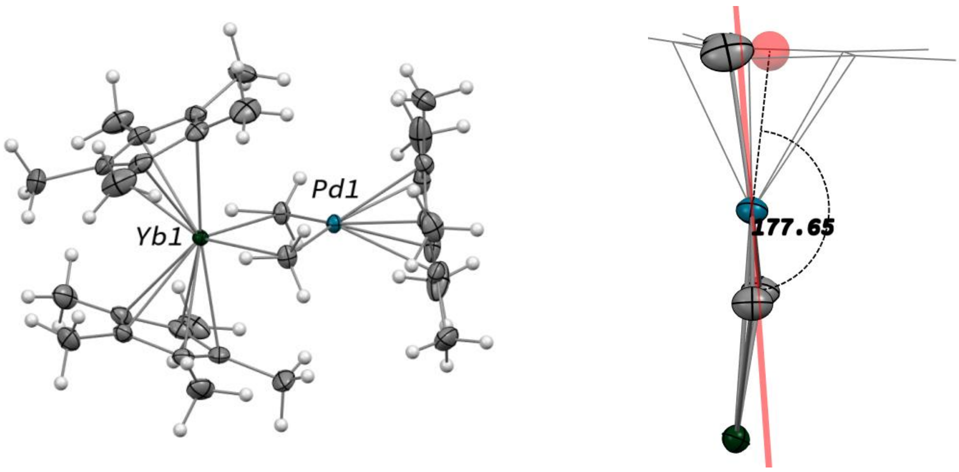

Figure S36. *ORTEP of **6** with 50% probability ellipsoids.*

Table S1. *Crystallographic data for compounds **1-6**.*

Compound	**1**	**2·(3 C_7H_8)**	**3·(4 C_4H_8O)**	**4·(2 C_7H_8)**	**5·(2 C_7H_8)**	**6**
Empirical formula	$C_{17}H_{15}N_5Pd$	$C_{95}H_{114}N_{10}Pd_2Yb_2$	$C_{98}H_{138}N_{10}O_6Pd_2Yb_2$	$C_{64}H_{84}N_2PdSm_2$	$C_{64}H_{84}N_2PdYb_2$	$C_{32}H_{51}PdYb$
Formula weight	395.74	1954.84	2111.06	1288.43	1333.81	715.16
Temperature/K	150.15	149.99	149.99	150.0	150.0	150.0
Crystal system	monoclinic	monoclinic	triclinic	monoclinic	monoclinic	monoclinic
Space group	$P2_1/c$	$P2_1/n$	P-1	C2/c	C2/c	$P2_1/n$
a/Å	10.4114(14)	14.7945(4)	14.758(4)	15.7006(11)	15.7166(4)	8.5080(9)
b/Å	8.5418(11)	34.6792(12)	17.469(5)	13.8198(8)	13.6208(3)	16.1094(16)
c/Å	17.017(2)	17.8510(5)	20.212(6)	26.6978(17)	26.5397(6)	21.445(2)
α/°	90	90	101.902(7)	90	90	90
β/°	92.924(4)	111.427(2)	102.431(7)	91.602(3)	91.6370(10)	91.493(2)
γ/°	90	90	113.691(7)	90	90	90
Volume/Å^3	1511.4(3)	8525.6(5)	4403(2)	5790.6(6)	5679.1(2)	2938.2(5)
Z	4	4	2	4	4	4
ρ_{calc} /g.cm^{-3}	1.739	1.523	1.592	1.478	1.560	1.617
μ/mm^{-1}	1.234	2.640	2.568	2.348	3.619	3.793
F(000)	792.0	3944.0	2152.0	2608.0	2672.0	1436.0
Crystal size/mm^3	0.16 × 0.08 × 0.02	0.34 × 0.28 × 0.06	0.28 × 0.04 × 0.02	0.12 × 0.08 × 0.04	0.12 × 0.04 × 0.04	0.60 × 0.32 × 0.28
Radiation	MoKα (λ = 0.71069)	MoKα (λ = 0.71073)	MoKα (λ = 0.71073)	MoKα (λ = 0.71069)	MoKα (λ = 0.71069)	MoKα (λ = 0.71073)
2Θ range for data collection/°	6.036 to 55.242	4.63 to 57.4	2.694 to 50.138	4.93 to 52.738	4.964 to 55.002	5.106 to 68.674
Index ranges	-13 ≤ h ≤ 13, -11 ≤ k ≤ 11, -21 ≤ l ≤ 22	-18 ≤ h ≤ 19, -38 ≤ k ≤ 46, -24 ≤ l ≤ 22	-17 ≤ h ≤ 17, -20 ≤ k ≤ 20, -24 ≤ l ≤ 24	-19 ≤ h ≤ 19, -17 ≤ k ≤ 17, -33 ≤ l ≤ 33	-20 ≤ h ≤ 18, -17 ≤ k ≤ 17, -34 ≤ l ≤ 26	-13 ≤ h ≤ 13, -25 ≤ k ≤ 25, -34 ≤ l ≤ 34
Reflections collected	37495	100236	96195	46629	21838	117934
Independent reflections	3490 [R_{int} = 0.0749, R_{sigma} = 0.0372]	21851 [R_{int} = 0.0964, R_{sigma} = 0.0795]	15419 [R_{int} = 0.1951, R_{sigma} = 0.1656]	5915 [R_{int} = 0.1212, R_{sigma} = 0.0864]	6389 [R_{int} = 0.0340, R_{sigma} = 0.0316]	12295 [R_{int} = 0.0586, R_{sigma} = 0.0286]
Data/restraints/parameters	3490/0/209	21851/1848/1290	15419/61/905	5915/0/323	6389/0/323	12295/0/340
Goodness-of-fit on F^2	1.026	1.020	0.999	0.891	1.076	1.106
Final R indexes [I>=2σ (I)]	R_1 = 0.0274, wR_2 = 0.0621	R_1 = 0.0515, wR_2 = 0.1148	R_1 = 0.0674, wR_2 = 0.1502	R_1 = 0.0378, wR_2 = 0.0685	R_1 = 0.0294, wR_2 = 0.0709	R_1 = 0.0275, wR_2 = 0.0588
Final R indexes [all data]	R_1 = 0.0451, wR_2 = 0.0682	R_1 = 0.0902, wR_2 = 0.1371	R_1 = 0.1530, wR_2 = 0.1898	R_1 = 0.0594, wR_2 = 0.0739	R_1 = 0.0324, wR_2 = 0.0725	R_1 = 0.0386, wR_2 = 0.0645
Largest diff. peak/hole / e Å^{-3}	0.39/-0.59	1.65/-1.64	2.94/-1.17	1.13/-1.28	2.88/-0.96	1.50/-1.96

Table S2. *Bond Lengths for* ***1***.

Atom	Atom	Length/Å	Atom	Atom	Length/Å
Pd1	N1	2.176(2)	C1	C2	1.460(4)
Pd1	N2	2.035(2)	C3	C4	1.402(4)
Pd1	N5	2.041(2)	C3	C8	1.420(3)
Pd1	C17	2.021(3)	C4	C5	1.380(4)
N1	C1	1.354(3)	C5	C6	1.405(4)
N1	C9	1.340(3)	C6	C7	1.378(4)
N2	C2	1.367(3)	C7	C8	1.403(3)
N2	C3	1.374(3)	C9	C10	1.376(4)
N3	C1	1.330(3)	C10	C11	1.380(4)
N3	C11	1.335(3)	C12	C13	1.378(4)
N4	C2	1.331(3)	C13	C14	1.384(4)
N4	C8	1.377(3)	C14	C15	1.374(4)
N5	C12	1.347(3)	C15	C16	1.371(4)
N5	C16	1.349(3)			

Table S3. *Bond Angles for* ***1***.

Atom	Atom	Atom	Angle/˚	Atom	Atom	Atom	Angle/˚
N2	Pd1	N1	79.38(8)	N4	C2	N2	116.4(2)
N2	Pd1	N5	174.97(8)	N4	C2	C1	124.4(2)
N5	Pd1	N1	96.40(8)	N2	C3	C4	132.7(2)
C17	Pd1	N1	174.62(10)	N2	C3	C8	107.2(2)
C17	Pd1	N2	95.34(11)	C4	C3	C8	120.0(2)
C17	Pd1	N5	88.83(11)	C5	C4	C3	117.7(2)
C1	N1	Pd1	112.15(17)	C4	C5	C6	122.3(3)
C9	N1	Pd1	131.46(18)	C7	C6	C5	120.8(3)
C9	N1	C1	116.4(2)	C6	C7	C8	117.9(2)
C2	N2	Pd1	113.94(17)	N4	C8	C3	109.8(2)
C2	N2	C3	103.7(2)	N4	C8	C7	129.0(2)
C3	N2	Pd1	142.31(17)	C7	C8	C3	121.1(2)
C1	N3	C11	116.3(2)	N1	C9	C10	121.9(2)
C2	N4	C8	102.9(2)	C9	C10	C11	117.1(3)
C12	N5	Pd1	121.31(18)	N3	C11	C10	122.6(3)
C12	N5	C16	117.4(2)	N5	C12	C13	122.8(3)
C16	N5	Pd1	120.92(19)	C12	C13	C14	118.7(3)
N1	C1	C2	114.9(2)	C15	C14	C13	118.9(3)
N3	C1	N1	125.7(2)	C16	C15	C14	119.4(3)
N3	C1	C2	119.4(2)	N5	C16	C15	122.7(3)
N2	C2	C1	119.0(2)				

Table S4. *Bond Lengths for **2**.*

Atom	Atom	Length/Å	Atom	Atom	Length/Å
Yb1	N4	2.317(5)	C40	C41	1.485(7)
Yb1	N12	2.353(5)	C42	C43	1.402(8)
Yb1	C13	3.090(6)	C42	C47	1.400(9)
Yb1	C14	3.146(5)	C43	C44	1.379(9)
Yb1	C70	2.636(5)	C44	C45	1.393(11)
Yb1	C69	2.657(5)	C45	C46	1.373(9)
Yb1	C68	2.648(5)	C46	C47	1.411(9)
Yb1	C67	2.621(5)	C48	C49	1.387(9)
Yb1	C66	2.614(5)	C49	C50	1.385(11)
Yb1	C74	2.617(7)	C50	C51	1.386(12)
Yb1	C75	2.609(6)	C51	C52	1.383(10)
Yb1	C76	2.669(6)	C54	C55	1.369(10)
Yb1	C77	2.716(7)	C55	C56	1.383(11)
Yb1	C79	2.684(8)	C56	C57	1.383(11)
Yb1	C79A	2.723(8)	C57	C58	1.365(9)
Yb1	C74A	2.703(9)	C60	C61	1.403(9)
Yb1	C75A	2.668(8)	C60	C65	1.407(8)
Yb1	C76A	2.666(7)	C61	C62	1.380(9)
Yb1	C77A	2.701(7)	C62	C63	1.401(10)
Yb1	C66A	2.673(10)	C63	C64	1.372(9)
Yb1	C70A	2.701(9)	C64	C65	1.401(9)
Yb1	C69A	2.716(9)	C70	C69	1.413(3)
Yb1	C68A	2.697(10)	C70	C66	1.413(3)
Yb1	C67A	2.671(10)	C70	C71	1.503(3)
Yb2	N6	2.318(5)	C69	C68	1.414(3)
Yb2	N7	2.324(5)	C69	C1	1.503(3)
Yb2	C25	2.643(9)	C68	C67	1.413(3)
Yb2	C27	2.628(10)	C68	C72	1.501(3)
Yb2	C20	2.655(10)	C67	C66	1.414(3)
Yb2	C21	2.687(9)	C67	C2	1.502(3)
Yb2	C23	2.679(8)	C66	C73	1.502(3)
Yb2	C35	2.755(6)	C74	C75	1.418(3)
Yb2	C37	2.684(6)	C74	C79	1.418(3)
Yb2	C30	2.627(6)	C74	C83	1.507(4)
Yb2	C32	2.665(6)	C75	C76	1.418(3)
Yb2	C33	2.743(6)	C75	C82	1.507(4)
Yb2	C40	3.091(6)	C76	C77	1.418(3)
Yb2	C41	3.148(6)	C76	C81	1.506(4)
Yb2	C32A	2.619(8)	C77	C79	1.417(3)
Yb2	C33A	2.606(9)	C77	C78	1.508(4)
Yb2	C35A	2.609(9)	C79	C80	1.506(4)
Yb2	C37A	2.624(8)	C87	C89	1.3900
Yb2	C30A	2.630(8)	C87	C86	1.3900
Yb2	C25A	2.689(7)	C87	C88	1.37(3)
Yb2	C27A	2.647(8)	C89	C90	1.3900
Yb2	C20A	2.627(9)	C90	C84	1.3900
Yb2	C21A	2.657(8)	C84	C85	1.3900
Yb2	C23A	2.695(7)	C85	C86	1.3900
Pd1	N2	2.035(5)	C32A	C33A	1.409(4)
Pd1	N3	2.151(5)	C32A	C30A	1.409(4)

Atom	Atom	Length/Å	Atom	Atom	Length/Å
Pd1	N11	2.027(5)	C32A	C39A	1.498(5)
Pd1	C59	2.032(6)	C33A	C35A	1.409(4)
Pd2	N8	2.175(5)	C33A	C34A	1.498(5)
Pd2	N9	2.030(5)	C35A	C37A	1.408(4)
Pd2	N10	2.026(5)	C35A	C36A	1.499(5)
Pd2	C53	2.022(6)	C37A	C30A	1.409(4)
N2	C13	1.338(7)	C37A	C38A	1.497(5)
N2	C60	1.376(8)	C30A	C31A	1.497(5)
N3	C14	1.316(7)	C25A	C27A	1.418(4)
N3	C16	1.467(7)	C25A	C23A	1.419(4)
N4	C5	1.395(8)	C25A	C26A	1.506(4)
N4	C14	1.346(7)	C27A	C20A	1.418(4)
C5	C15	1.330(8)	C27A	C28A	1.507(4)
N6	C19	1.390(7)	C20A	C21A	1.418(4)
N6	C41	1.343(7)	C20A	C29A	1.507(4)
N7	C40	1.348(8)	C21A	C23A	1.417(4)
N7	C47	1.390(7)	C21A	C22A	1.507(4)
N8	C17	1.489(7)	C23A	C24A	1.507(4)
N8	C41	1.299(8)	C79A	C74A	1.408(4)
N9	C40	1.324(7)	C79A	C77A	1.408(4)
N9	C42	1.394(7)	C79A	C80A	1.497(4)
N10	C48	1.355(8)	C74A	C75A	1.408(4)
N10	C52	1.337(8)	C74A	C83A	1.497(4)
N11	C54	1.343(9)	C75A	C76A	1.408(4)
N11	C58	1.353(8)	C75A	C82A	1.498(4)
N12	C13	1.342(7)	C76A	C77A	1.409(4)
N12	C65	1.385(8)	C76A	C81A	1.497(4)
C6	C7	1.369(15)	C77A	C78A	1.496(4)
C6	C11	1.384(15)	C66A	C70A	1.402(5)
C7	C8	1.368(15)	C66A	C67A	1.404(5)
C8	C9	1.383(15)	C66A	C73A	1.493(6)
C9	C10	1.503(17)	C70A	C69A	1.404(5)
C9	C12	1.386(15)	C70A	C71A	1.492(6)
C11	C12	1.338(15)	C69A	C68A	1.403(5)
C13	C14	1.465(9)	C69A	C1A	1.491(6)
C15	C16	1.506(8)	C68A	C67A	1.404(5)
C16	C17	1.583(9)	C68A	C72A	1.493(6)
C17	C18	1.494(8)	C67A	C2A	1.492(6)
C18	C19	1.341(8)	C3	C4	1.369(11)
C25	C27	1.416(4)	C3	C94	1.368(11)
C25	C23	1.416(4)	C4	C91	1.368(11)
C25	C26	1.507(5)	C91	C92	1.368(11)
C27	C20	1.416(4)	C92	C93	1.368(11)
C27	C28	1.506(5)	C92	C95	1.56(3)
C20	C21	1.418(4)	C93	C94	1.369(11)
C20	C29	1.505(5)	C92A	C91A	1.3900
C21	C23	1.417(4)	C92A	C93A	1.3900
C21	C22	1.505(5)	C92A	C95A	1.36(3)
C23	C24	1.505(5)	C91A	C4A	1.3900
C35	C37	1.414(3)	C4A	C3A	1.3900
C35	C33	1.414(3)	C3A	C94A	1.3900
C35	C36	1.504(3)	C94A	C93A	1.3900

Atom	Atom	Length/Å	Atom	Atom	Length/Å
C37	C30	1.414(3)	C87A	C89A	1.3900
C37	C38	1.503(3)	C87A	C86A	1.3900
C30	C32	1.414(3)	C87A	C88A	1.56(4)
C30	C31	1.504(3)	C89A	C90A	1.3900
C32	C33	1.415(3)	C90A	C84A	1.3900
C32	C39	1.503(3)	C84A	C85A	1.3900
C33	C34	1.503(3)	C85A	C86A	1.3900

Table S5. *Bond Angles for **2**.*

Atom	Atom	Atom	Angle/˚	Atom	Atom	Atom	Angle/˚
N4	Yb1	N12	73.68(17)	C16	N3	Pd1	132.5(3)
N4	Yb1	C13	49.82(17)	C5	N4	Yb1	129.7(4)
N4	Yb1	C14	22.67(17)	C14	N4	Yb1	115.8(4)
N4	Yb1	C70	119.44(18)	C14	N4	C5	112.5(5)
N4	Yb1	C69	132.84(17)	C15	C5	N4	123.7(6)
N4	Yb1	C68	106.84(17)	C19	N6	Yb2	130.2(4)
N4	Yb1	C67	81.52(17)	C41	N6	Yb2	116.0(3)
N4	Yb1	C66	88.68(17)	C41	N6	C19	112.8(5)
N4	Yb1	C74	85.08(18)	C40	N7	Yb2	111.9(4)
N4	Yb1	C75	113.53(19)	C40	N7	C47	101.8(5)
N4	Yb1	C76	134.94(18)	C47	N7	Yb2	146.1(4)
N4	Yb1	C77	114.86(18)	C17	N8	Pd2	132.0(4)
N4	Yb1	C79	86.54(17)	C41	N8	Pd2	111.1(4)
N4	Yb1	C79A	88.29(19)	C41	N8	C17	116.9(5)
N4	Yb1	C74A	78.72(19)	C40	N9	Pd2	112.2(4)
N4	Yb1	C75A	102.0(2)	C40	N9	C42	103.7(5)
N4	Yb1	C76A	128.8(2)	C42	N9	Pd2	143.1(4)
N4	Yb1	C77A	118.3(2)	C48	N10	Pd2	117.5(4)
N4	Yb1	C66A	78.8(2)	C52	N10	Pd2	124.0(5)
N4	Yb1	C70A	101.1(2)	C52	N10	C48	118.5(6)
N4	Yb1	C69A	128.3(2)	C54	N11	Pd1	118.9(4)
N4	Yb1	C68A	120.3(3)	C54	N11	C58	118.4(6)
N4	Yb1	C67A	89.9(2)	C58	N11	Pd1	122.7(5)
N12	Yb1	C13	24.02(16)	C13	N12	Yb1	110.5(4)
N12	Yb1	C14	51.08(16)	C13	N12	C65	103.3(5)
N12	Yb1	C70	87.39(17)	C65	N12	Yb1	145.7(4)
N12	Yb1	C69	117.25(17)	C7	C6	C11	117.5(11)
N12	Yb1	C68	134.03(17)	C8	C7	C6	120.5(10)
N12	Yb1	C67	109.83(17)	C7	C8	C9	121.9(10)
N12	Yb1	C66	82.91(17)	C8	C9	C10	121.9(12)
N12	Yb1	C74	90.65(18)	C8	C9	C12	116.7(11)
N12	Yb1	C75	86.22(19)	C12	C9	C10	121.4(12)
N12	Yb1	C76	112.79(19)	C12	C11	C6	122.2(11)
N12	Yb1	C77	136.87(19)	C11	C12	C9	121.2(10)

Atom	Atom	Atom	Angle/˚	Atom	Atom	Atom	Angle/˚
N12	Yb1	C79	120.47(18)	N2	C13	Yb1	160.2(4)
N12	Yb1	C79A	118.78(19)	N2	C13	N12	115.6(6)
N12	Yb1	C74A	88.71(19)	N2	C13	C14	120.6(5)
N12	Yb1	C75A	76.9(2)	N12	C13	Yb1	45.5(3)
N12	Yb1	C76A	99.3(2)	N12	C13	C14	123.7(5)
N12	Yb1	C77A	126.7(2)	C14	C13	Yb1	78.6(3)
N12	Yb1	C66A	90.0(2)	N3	C14	Yb1	170.1(5)
N12	Yb1	C70A	77.0(2)	N3	C14	N4	129.0(6)
N12	Yb1	C69A	97.8(2)	N3	C14	C13	115.1(5)
N12	Yb1	C68A	125.9(3)	N4	C14	Yb1	41.6(3)
N12	Yb1	C67A	120.4(3)	N4	C14	C13	115.8(5)
C13	Yb1	C14	27.15(16)	C13	C14	Yb1	74.3(3)
C70	Yb1	C13	101.96(17)	C5	C15	C16	119.5(6)
C70	Yb1	C14	113.74(17)	N3	C16	C15	109.3(5)
C70	Yb1	C69	30.97(8)	N3	C16	C17	111.3(5)
C70	Yb1	C68	51.31(12)	C15	C16	C17	111.1(5)
C70	Yb1	C76	105.55(18)	N8	C17	C16	111.2(4)
C70	Yb1	C77	117.65(18)	N8	C17	C18	108.8(5)
C70	Yb1	C79	147.57(17)	C18	C17	C16	112.0(5)
C69	Yb1	C13	132.82(17)	C19	C18	C17	119.9(5)
C69	Yb1	C14	138.80(16)	C18	C19	N6	123.2(5)
C69	Yb1	C76	85.77(18)	C27	C25	Yb2	73.8(3)
C69	Yb1	C77	88.57(18)	C27	C25	C26	126.0
C69	Yb1	C79	117.14(17)	C23	C25	Yb2	76.0(3)
C68	Yb1	C13	134.83(16)	C23	C25	C27	108.1
C68	Yb1	C14	122.15(16)	C23	C25	C26	126.0
C68	Yb1	C69	30.91(8)	C26	C25	Yb2	116.3(3)
C68	Yb1	C76	98.84(19)	C25	C27	Yb2	75.0(3)
C68	Yb1	C77	86.02(18)	C25	C27	C20	108.0
C68	Yb1	C79	105.27(17)	C25	C27	C28	126.0
C67	Yb1	C13	104.23(16)	C20	C27	Yb2	75.5(3)
C67	Yb1	C14	92.33(16)	C20	C27	C28	126.0
C67	Yb1	C70	51.58(12)	C28	C27	Yb2	115.7(3)
C67	Yb1	C69	51.34(12)	C27	C20	Yb2	73.4(3)
C67	Yb1	C68	31.11(8)	C27	C20	C21	108.0
C67	Yb1	C76	129.95(18)	C27	C20	C29	126.0
C67	Yb1	C77	113.23(18)	C21	C20	Yb2	75.9(3)
C67	Yb1	C79	122.11(18)	C21	C20	C29	126.0
C66	Yb1	C13	86.24(16)	C29	C20	Yb2	116.8(3)
C66	Yb1	C14	87.62(16)	C20	C21	Yb2	73.4(3)
C66	Yb1	C70	31.23(8)	C20	C21	C22	126.0
C66	Yb1	C69	51.41(12)	C23	C21	Yb2	74.4(3)
C66	Yb1	C68	51.53(12)	C23	C21	C20	107.9
C66	Yb1	C67	31.33(8)	C23	C21	C22	126.0

Atom	Atom	Atom	Angle/˚	Atom	Atom	Atom	Angle/˚
C66	Yb1	C74	172.09(16)	C22	C21	Yb2	118.1(3)
C66	Yb1	C76	135.63(17)	C25	C23	Yb2	73.2(3)
C66	Yb1	C77	136.73(17)	C25	C23	C21	108.0
C66	Yb1	C79	153.41(18)	C25	C23	C24	126.1
C74	Yb1	C13	86.02(17)	C21	C23	Yb2	75.0(3)
C74	Yb1	C14	84.75(17)	C21	C23	C24	125.9
C74	Yb1	C70	153.52(18)	C24	C23	Yb2	117.8(3)
C74	Yb1	C69	136.35(17)	C37	C35	Yb2	72.19(19)
C74	Yb1	C68	135.25(17)	C37	C35	C36	126.0
C74	Yb1	C67	151.02(18)	C33	C35	Yb2	74.66(18)
C74	Yb1	C76	51.42(14)	C33	C35	C37	108.1
C74	Yb1	C77	50.91(15)	C33	C35	C36	126.0
C74	Yb1	C79	30.99(10)	C36	C35	Yb2	119.05(19)
C75	Yb1	C13	94.46(18)	C35	C37	Yb2	77.7(2)
C75	Yb1	C14	105.84(18)	C35	C37	C30	108.0
C75	Yb1	C70	122.08(18)	C35	C37	C38	126.0
C75	Yb1	C69	112.91(18)	C30	C37	Yb2	72.3(2)
C75	Yb1	C68	129.98(18)	C30	C37	C38	126.0
C75	Yb1	C67	161.09(18)	C38	C37	Yb2	116.02(18)
C75	Yb1	C66	151.34(18)	C37	C30	Yb2	76.8(2)
C75	Yb1	C74	31.48(9)	C37	C30	C32	108.1
C75	Yb1	C76	31.14(9)	C37	C30	C31	126.0
C75	Yb1	C77	50.99(14)	C32	C30	Yb2	75.98(19)
C75	Yb1	C79	51.34(15)	C32	C30	C31	126.0
C76	Yb1	C13	125.24(19)	C31	C30	Yb2	113.6(2)
C76	Yb1	C14	135.18(18)	C30	C32	Yb2	73.03(19)
C76	Yb1	C77	30.51(9)	C30	C32	C33	107.9
C76	Yb1	C79	50.74(14)	C30	C32	C39	126.0
C77	Yb1	C13	136.88(18)	C33	C32	Yb2	77.9(2)
C77	Yb1	C14	128.01(17)	C33	C32	C39	126.0
C79	Yb1	C13	110.01(17)	C39	C32	Yb2	115.21(19)
C79	Yb1	C14	97.58(17)	C35	C33	Yb2	75.55(18)
C79	Yb1	C77	30.43(9)	C35	C33	C32	108.0
C79A	Yb1	C13	109.49(19)	C35	C33	C34	126.0
C79A	Yb1	C14	98.38(18)	C32	C33	Yb2	71.8(2)
C74A	Yb1	C13	81.83(19)	C32	C33	C34	126.0
C74A	Yb1	C14	78.84(19)	C34	C33	Yb2	118.56(18)
C74A	Yb1	C79A	30.07(11)	N7	C40	Yb2	44.2(3)
C74A	Yb1	C69A	153.0(3)	N7	C40	C41	122.5(5)
C75A	Yb1	C13	82.4(2)	N9	C40	Yb2	161.3(4)
C75A	Yb1	C14	93.0(2)	N9	C40	N7	117.4(5)
C75A	Yb1	C79A	50.00(17)	N9	C40	C41	120.1(5)
C75A	Yb1	C74A	30.40(11)	C41	C40	Yb2	78.3(3)
C75A	Yb1	C77A	50.23(16)	N6	C41	Yb2	41.4(3)

Atom	Atom	Atom	Angle/°	Atom	Atom	Atom	Angle/°
C75A	Yb1	C66A	165.9(3)	N6	C41	C40	115.5(5)
C75A	Yb1	C70A	138.3(3)	N8	C41	Yb2	170.8(4)
C75A	Yb1	C69A	126.1(3)	N8	C41	N6	129.3(5)
C75A	Yb1	C68A	135.4(3)	N8	C41	C40	115.1(5)
C75A	Yb1	C67A	161.6(3)	C40	C41	Yb2	74.1(3)
C76A	Yb1	C13	111.1(2)	N9	C42	C43	131.6(6)
C76A	Yb1	C14	123.5(2)	N9	C42	C47	107.3(5)
C76A	Yb1	C79A	50.01(16)	C47	C42	C43	121.1(6)
C76A	Yb1	C74A	50.21(17)	C44	C43	C42	117.2(7)
C76A	Yb1	C75A	30.61(10)	C43	C44	C45	121.4(6)
C76A	Yb1	C77A	30.43(10)	C46	C45	C44	122.6(6)
C76A	Yb1	C66A	152.3(2)	C45	C46	C47	116.5(7)
C76A	Yb1	C70A	127.2(3)	N7	C47	C42	109.7(5)
C76A	Yb1	C69A	102.8(3)	N7	C47	C46	129.2(6)
C76A	Yb1	C68A	105.0(3)	C42	C47	C46	121.1(6)
C76A	Yb1	C67A	132.1(3)	N10	C48	C49	121.9(7)
C77A	Yb1	C13	130.08(19)	C50	C49	C48	119.0(7)
C77A	Yb1	C14	127.13(19)	C49	C50	C51	119.0(7)
C77A	Yb1	C79A	30.08(10)	C52	C51	C50	119.0(7)
C77A	Yb1	C74A	49.86(17)	N10	C52	C51	122.6(7)
C77A	Yb1	C70A	137.6(2)	N11	C54	C55	122.3(7)
C77A	Yb1	C69A	107.6(3)	C54	C55	C56	119.5(7)
C66A	Yb1	C13	87.7(2)	C57	C56	C55	118.1(6)
C66A	Yb1	C14	82.5(2)	C58	C57	C56	120.0(7)
C66A	Yb1	C79A	143.8(3)	N11	C58	C57	121.7(7)
C66A	Yb1	C74A	156.9(2)	N2	C60	C61	131.8(6)
C66A	Yb1	C77A	141.7(3)	N2	C60	C65	107.6(5)
C66A	Yb1	C70A	30.25(14)	C61	C60	C65	120.6(6)
C66A	Yb1	C69A	49.8(2)	C62	C61	C60	117.9(6)
C66A	Yb1	C68A	50.0(2)	C61	C62	C63	121.6(6)
C70A	Yb1	C13	86.7(2)	C64	C63	C62	120.8(6)
C70A	Yb1	C14	95.3(2)	C63	C64	C65	118.8(6)
C70A	Yb1	C79A	163.6(2)	N12	C65	C60	108.8(5)
C70A	Yb1	C74A	165.0(2)	N12	C65	C64	131.0(6)
C70A	Yb1	C69A	30.04(14)	C64	C65	C60	120.2(6)
C69A	Yb1	C13	113.8(2)	C69	C70	Yb1	75.34(16)
C69A	Yb1	C14	125.3(2)	C69	C70	C66	108.0
C69A	Yb1	C79A	135.4(3)	C69	C70	C71	126.0
C68A	Yb1	C13	135.2(3)	C66	C70	Yb1	73.52(17)
C68A	Yb1	C14	131.5(2)	C66	C70	C71	126.0
C68A	Yb1	C79A	113.9(3)	C71	C70	Yb1	117.18(16)
C68A	Yb1	C74A	142.9(3)	C70	C69	Yb1	73.69(16)
C68A	Yb1	C77A	94.2(3)	C70	C69	C68	108.0
C68A	Yb1	C70A	49.7(2)	C70	C69	C1	126.0

Atom	Atom	Atom	Angle/˚	Atom	Atom	Atom	Angle/˚
C68A	Yb1	C69A	30.04(14)	C68	C69	Yb1	74.18(16)
C67A	Yb1	C13	116.0(2)	C68	C69	C1	126.0
C67A	Yb1	C14	103.0(2)	C1	C69	Yb1	118.07(16)
C67A	Yb1	C79A	117.5(3)	C69	C68	Yb1	74.91(17)
C67A	Yb1	C74A	144.6(3)	C69	C68	C72	126.0
C67A	Yb1	C77A	111.7(3)	C67	C68	Yb1	73.40(17)
C67A	Yb1	C66A	30.46(14)	C67	C68	C69	108.0
C67A	Yb1	C70A	50.0(2)	C67	C68	C72	126.0
C67A	Yb1	C69A	49.9(2)	C72	C68	Yb1	117.70(16)
C67A	Yb1	C68A	30.32(14)	C68	C67	Yb1	75.49(17)
N6	Yb2	N7	73.89(16)	C68	C67	C66	108.0
N6	Yb2	C25	96.2(2)	C68	C67	C2	126.0
N6	Yb2	C27	78.1(2)	C66	C67	Yb1	74.05(16)
N6	Yb2	C20	95.2(2)	C66	C67	C2	126.0
N6	Yb2	C21	125.5(2)	C2	C67	Yb1	116.53(16)
N6	Yb2	C23	126.3(2)	C70	C66	Yb1	75.25(17)
N6	Yb2	C35	130.69(18)	C70	C66	C67	108.0
N6	Yb2	C37	119.70(19)	C70	C66	C73	126.0
N6	Yb2	C30	89.05(19)	C67	C66	Yb1	74.61(17)
N6	Yb2	C32	80.79(18)	C67	C66	C73	126.0
N6	Yb2	C33	105.03(17)	C73	C66	Yb1	116.21(16)
N6	Yb2	C40	50.04(15)	C75	C74	Yb1	73.9(2)
N6	Yb2	C41	22.54(15)	C75	C74	C79	108.0
N6	Yb2	C32A	83.3(2)	C75	C74	C83	126.0
N6	Yb2	C33A	113.5(2)	C79	C74	Yb1	77.1(2)
N6	Yb2	C35A	130.6(2)	C79	C74	C83	126.0
N6	Yb2	C37A	107.0(2)	C83	C74	Yb1	115.2(2)
N6	Yb2	C30A	79.7(2)	C74	C75	Yb1	74.6(2)
N6	Yb2	C25A	104.3(2)	C74	C75	C76	108.0
N6	Yb2	C27A	82.34(19)	C74	C75	C82	126.0
N6	Yb2	C20A	93.91(19)	C76	C75	Yb1	76.8(2)
N6	Yb2	C21A	125.03(19)	C76	C75	C82	126.0
N6	Yb2	C23A	132.6(2)	C82	C75	Yb1	114.8(2)
N7	Yb2	C25	78.5(2)	C75	C76	Yb1	72.1(2)
N7	Yb2	C27	97.6(2)	C75	C76	C81	126.0
N7	Yb2	C20	127.6(2)	C77	C76	Yb1	76.5(2)
N7	Yb2	C21	124.8(2)	C77	C76	C75	108.0
N7	Yb2	C23	94.5(2)	C77	C76	C81	126.0
N7	Yb2	C35	118.47(19)	C81	C76	Yb1	117.3(2)
N7	Yb2	C37	89.05(18)	C76	C77	Yb1	72.9(2)
N7	Yb2	C30	83.08(19)	C76	C77	C78	126.0
N7	Yb2	C32	109.10(19)	C79	C77	Yb1	73.6(2)
N7	Yb2	C33	133.26(19)	C79	C77	C76	108.0
N7	Yb2	C40	23.85(15)	C79	C77	C78	126.0

Atom	Atom	Atom	Angle/˚	Atom	Atom	Atom	Angle/˚
N7	Yb2	C41	51.35(15)	C78	C77	Yb1	119.35(19)
N7	Yb2	C32A	118.6(2)	C74	C79	Yb1	71.9(2)
N7	Yb2	C33A	133.5(2)	C74	C79	C80	126.0
N7	Yb2	C35A	107.6(2)	C77	C79	Yb1	76.0(2)
N7	Yb2	C37A	81.9(2)	C77	C79	C74	108.0
N7	Yb2	C30A	88.1(2)	C77	C79	C80	126.0
N7	Yb2	C25A	81.3(2)	C80	C79	Yb1	118.1(2)
N7	Yb2	C27A	95.7(2)	C89	C87	C86	120.0
N7	Yb2	C20A	126.8(2)	C88	C87	C89	113.6(17)
N7	Yb2	C21A	130.3(2)	C88	C87	C86	126.1(17)
N7	Yb2	C23A	100.6(2)	C90	C89	C87	120.0
C25	Yb2	C20	51.3(2)	C84	C90	C89	120.0
C25	Yb2	C21	50.92(19)	C85	C84	C90	120.0
C25	Yb2	C23	30.84(12)	C86	C85	C84	120.0
C25	Yb2	C35	132.2(2)	C85	C86	C87	120.0
C25	Yb2	C37	137.1(2)	C33A	C32A	Yb2	73.8(3)
C25	Yb2	C32	170.4(2)	C33A	C32A	C39A	126.0
C25	Yb2	C33	145.4(2)	C30A	C32A	Yb2	74.9(3)
C25	Yb2	C40	83.1(2)	C30A	C32A	C33A	108.0
C25	Yb2	C41	91.1(2)	C30A	C32A	C39A	126.0
C27	Yb2	C25	31.16(13)	C39A	C32A	Yb2	117.3(2)
C27	Yb2	C20	31.10(14)	C32A	C33A	Yb2	74.9(3)
C27	Yb2	C21	51.1(2)	C32A	C33A	C35A	108.0
C27	Yb2	C23	51.1(2)	C32A	C33A	C34A	126.0
C27	Yb2	C35	137.6(2)	C35A	C33A	Yb2	74.5(2)
C27	Yb2	C37	162.2(2)	C35A	C33A	C34A	126.0
C27	Yb2	C32	139.6(2)	C34A	C33A	Yb2	116.7(3)
C27	Yb2	C33	128.5(2)	C33A	C35A	Yb2	74.2(3)
C27	Yb2	C40	90.6(2)	C33A	C35A	C36A	125.9
C27	Yb2	C41	83.9(2)	C37A	C35A	Yb2	75.0(3)
C20	Yb2	C21	30.77(13)	C37A	C35A	C33A	108.0
C20	Yb2	C23	50.9(2)	C37A	C35A	C36A	126.1
C20	Yb2	C35	107.4(2)	C36A	C35A	Yb2	116.8(2)
C20	Yb2	C37	136.5(2)	C35A	C37A	Yb2	73.8(3)
C20	Yb2	C32	119.7(2)	C35A	C37A	C30A	108.0
C20	Yb2	C33	99.2(2)	C35A	C37A	C38A	126.0
C20	Yb2	C40	121.2(2)	C30A	C37A	Yb2	74.7(2)
C20	Yb2	C41	109.0(2)	C30A	C37A	C38A	126.0
C21	Yb2	C35	88.3(2)	C38A	C37A	Yb2	117.5(3)
C21	Yb2	C33	94.5(2)	C32A	C30A	Yb2	74.0(3)
C21	Yb2	C40	134.0(2)	C32A	C30A	C37A	108.0
C21	Yb2	C41	135.0(2)	C32A	C30A	C31A	126.0
C23	Yb2	C21	30.63(12)	C37A	C30A	Yb2	74.2(2)
C23	Yb2	C35	101.3(2)	C37A	C30A	C31A	126.0

Atom	Atom	Atom	Angle/˚	Atom	Atom	Atom	Angle/˚
C23	Yb2	C37	112.1(2)	C31A	C30A	Yb2	117.8(3)
C23	Yb2	C33	119.0(2)	C27A	C25A	Yb2	73.0(3)
C23	Yb2	C40	107.7(2)	C27A	C25A	C23A	108.0
C23	Yb2	C41	121.3(2)	C27A	C25A	C26A	126.0
C35	Yb2	C40	131.33(18)	C23A	C25A	Yb2	75.0(2)
C35	Yb2	C41	135.50(17)	C23A	C25A	C26A	126.0
C37	Yb2	C21	111.9(2)	C26A	C25A	Yb2	118.1(3)
C37	Yb2	C35	30.09(9)	C25A	C27A	Yb2	76.2(2)
C37	Yb2	C33	49.85(14)	C25A	C27A	C20A	108.0
C37	Yb2	C40	101.67(18)	C25A	C27A	C28A	126.0
C37	Yb2	C41	112.81(18)	C20A	C27A	Yb2	73.6(2)
C30	Yb2	C25	158.6(2)	C20A	C27A	C28A	126.0
C30	Yb2	C27	166.3(2)	C28A	C27A	Yb2	116.3(2)
C30	Yb2	C20	149.0(2)	C27A	C20A	Yb2	75.2(2)
C30	Yb2	C21	138.5(2)	C27A	C20A	C21A	108.0
C30	Yb2	C23	142.5(2)	C27A	C20A	C29A	126.0
C30	Yb2	C35	50.22(14)	C21A	C20A	Yb2	75.6(2)
C30	Yb2	C37	30.85(9)	C21A	C20A	C29A	126.0
C30	Yb2	C32	30.99(9)	C29A	C20A	Yb2	115.5(2)
C30	Yb2	C33	50.36(14)	C20A	C21A	Yb2	73.3(2)
C30	Yb2	C40	84.55(17)	C20A	C21A	C22A	126.0
C30	Yb2	C41	85.91(17)	C23A	C21A	Yb2	76.1(2)
C32	Yb2	C21	123.9(2)	C23A	C21A	C20A	108.0
C32	Yb2	C23	149.0(2)	C23A	C21A	C22A	126.0
C32	Yb2	C35	49.91(14)	C22A	C21A	Yb2	116.6(2)
C32	Yb2	C37	50.66(14)	C25A	C23A	Yb2	74.5(2)
C32	Yb2	C33	30.28(9)	C25A	C23A	C24A	126.0
C32	Yb2	C40	101.60(17)	C21A	C23A	Yb2	73.2(3)
C32	Yb2	C41	89.51(17)	C21A	C23A	C25A	108.0
C33	Yb2	C35	29.79(9)	C21A	C23A	C24A	126.0
C33	Yb2	C40	131.44(17)	C24A	C23A	Yb2	118.2(2)
C33	Yb2	C41	118.50(17)	C74A	C79A	Yb1	74.2(2)
C40	Yb2	C41	27.52(14)	C74A	C79A	C80A	126.0
C32A	Yb2	C40	109.8(2)	C77A	C79A	Yb1	74.1(2)
C32A	Yb2	C41	95.1(2)	C77A	C79A	C74A	108.0
C32A	Yb2	C37A	51.54(19)	C77A	C79A	C80A	126.0
C32A	Yb2	C30A	31.13(12)	C80A	C79A	Yb1	117.7(2)
C32A	Yb2	C25A	160.1(2)	C79A	C74A	Yb1	75.8(2)
C32A	Yb2	C27A	137.1(2)	C79A	C74A	C75A	108.0
C32A	Yb2	C20A	110.8(2)	C79A	C74A	C83A	126.0
C32A	Yb2	C21A	109.7(3)	C75A	C74A	Yb1	73.4(2)
C32A	Yb2	C23A	134.3(3)	C75A	C74A	C83A	125.9
C33A	Yb2	C40	136.2(2)	C83A	C74A	Yb1	116.9(2)
C33A	Yb2	C41	126.3(2)	C74A	C75A	Yb1	76.2(3)

Atom	Atom	Atom	Angle/˚	Atom	Atom	Atom	Angle/˚
C33A	Yb2	C32A	31.28(13)	C74A	C75A	C82A	126.0
C33A	Yb2	C35A	31.36(13)	C76A	C75A	Yb1	74.6(2)
C33A	Yb2	C37A	51.7(2)	C76A	C75A	C74A	108.0
C33A	Yb2	C30A	51.61(19)	C76A	C75A	C82A	126.0
C33A	Yb2	C25A	133.7(3)	C82A	C75A	Yb1	115.4(2)
C33A	Yb2	C27A	130.4(2)	C75A	C76A	Yb1	74.8(2)
C33A	Yb2	C20A	99.3(2)	C75A	C76A	C77A	108.0
C33A	Yb2	C21A	85.0(3)	C75A	C76A	C81A	126.0
C33A	Yb2	C23A	104.0(3)	C77A	C76A	Yb1	76.1(2)
C35A	Yb2	C40	121.4(2)	C77A	C76A	C81A	126.0
C35A	Yb2	C41	130.0(2)	C81A	C76A	Yb1	115.3(3)
C35A	Yb2	C32A	51.7(2)	C79A	C77A	Yb1	75.8(3)
C35A	Yb2	C37A	31.21(12)	C79A	C77A	C76A	108.0
C35A	Yb2	C30A	51.57(19)	C79A	C77A	C78A	126.0
C35A	Yb2	C25A	125.0(3)	C76A	C77A	Yb1	73.4(2)
C35A	Yb2	C27A	143.4(2)	C76A	C77A	C78A	126.0
C35A	Yb2	C20A	118.3(2)	C78A	C77A	Yb1	116.8(2)
C35A	Yb2	C21A	92.5(3)	C70A	C66A	Yb1	76.0(3)
C35A	Yb2	C23A	96.3(3)	C70A	C66A	C67A	108.0
C37A	Yb2	C40	91.0(2)	C70A	C66A	C73A	126.1
C37A	Yb2	C41	99.9(2)	C67A	C66A	Yb1	74.7(3)
C37A	Yb2	C30A	31.11(12)	C67A	C66A	C73A	125.9
C37A	Yb2	C25A	138.5(2)	C73A	C66A	Yb1	115.5(3)
C37A	Yb2	C27A	169.1(2)	C66A	C70A	Yb1	73.8(3)
C37A	Yb2	C20A	149.0(2)	C66A	C70A	C69A	108.1
C37A	Yb2	C21A	123.1(3)	C66A	C70A	C71A	126.0
C37A	Yb2	C23A	119.0(2)	C69A	C70A	Yb1	75.6(3)
C30A	Yb2	C40	84.6(2)	C69A	C70A	C71A	125.9
C30A	Yb2	C41	80.3(2)	C71A	C70A	Yb1	116.7(3)
C30A	Yb2	C25A	167.1(2)	C70A	C69A	Yb1	74.4(3)
C30A	Yb2	C27A	159.8(2)	C70A	C69A	C1A	126.1
C30A	Yb2	C21A	136.6(2)	C68A	C69A	Yb1	74.2(3)
C30A	Yb2	C23A	147.7(2)	C68A	C69A	C70A	107.9
C25A	Yb2	C40	88.63(19)	C68A	C69A	C1A	126.0
C25A	Yb2	C41	98.6(2)	C1A	C69A	Yb1	117.4(3)
C25A	Yb2	C23A	30.57(10)	C69A	C68A	Yb1	75.7(3)
C27A	Yb2	C40	90.83(18)	C69A	C68A	C67A	108.0
C27A	Yb2	C41	86.60(18)	C69A	C68A	C72A	126.0
C27A	Yb2	C25A	30.80(10)	C67A	C68A	Yb1	73.8(3)
C27A	Yb2	C21A	51.27(18)	C67A	C68A	C72A	126.0
C27A	Yb2	C23A	50.87(16)	C72A	C68A	Yb1	116.6(3)
C20A	Yb2	C40	119.98(18)	C66A	C67A	Yb1	74.8(3)
C20A	Yb2	C41	107.58(18)	C66A	C67A	C68A	107.9
C20A	Yb2	C30A	141.5(2)	C66A	C67A	C2A	126.0

Atom	Atom	Atom	Angle/˚	Atom	Atom	Atom	Angle/˚
C20A	Yb2	C25A	51.12(17)	C68A	C67A	Yb1	75.9(3)
C20A	Yb2	C27A	31.19(12)	C68A	C67A	C2A	126.0
C20A	Yb2	C21A	31.13(12)	C2A	C67A	Yb1	115.4(3)
C20A	Yb2	C23A	51.05(17)	C94	C3	C4	119.9
C21A	Yb2	C40	138.8(2)	C91	C4	C3	120.0
C21A	Yb2	C41	137.0(2)	C4	C91	C92	120.0
C21A	Yb2	C25A	50.84(16)	C91	C92	C95	123.9(16)
C21A	Yb2	C23A	30.70(11)	C93	C92	C91	120.0
C23A	Yb2	C40	115.2(2)	C93	C92	C95	115.9(17)
C23A	Yb2	C41	129.1(2)	C92	C93	C94	119.9
N2	Pd1	N3	80.50(19)	C3	C94	C93	120.1
N11	Pd1	N2	173.8(2)	C91A	C92A	C93A	120.0
N11	Pd1	N3	96.1(2)	C95A	C92A	C91A	118.6(16)
N11	Pd1	C59	88.8(2)	C95A	C92A	C93A	121.3(16)
C59	Pd1	N2	94.2(2)	C92A	C91A	C4A	120.0
C59	Pd1	N3	173.8(2)	C91A	C4A	C3A	120.0
N9	Pd2	N8	79.82(18)	C94A	C3A	C4A	120.0
N10	Pd2	N8	96.7(2)	C93A	C94A	C3A	120.0
N10	Pd2	N9	174.1(2)	C94A	C93A	C92A	120.0
C53	Pd2	N8	173.4(3)	C89A	C87A	C86A	120.0
C53	Pd2	N9	93.8(3)	C89A	C87A	C88A	127.3(15)
C53	Pd2	N10	89.5(3)	C86A	C87A	C88A	112.7(15)
C13	N2	Pd1	110.2(4)	C90A	C89A	C87A	120.0
C13	N2	C60	104.7(5)	C89A	C90A	C84A	120.0
C60	N2	Pd1	143.5(4)	C90A	C84A	C85A	120.0
C14	N3	Pd1	110.2(4)	C84A	C85A	C86A	120.0
C14	N3	C16	117.2(5)	C85A	C86A	C87A	120.0

Table S6. *Bond Lengths for **3**.*

Atom	Atom	Length/Å	Atom	Atom	Length/Å
Yb1	N3	2.328(9)	C15	C16	1.394(17)
Yb1	N4	2.365(10)	C18	C22	1.464(16)
Yb1	C35	2.609(11)	C19	C20	1.473(14)
Yb1	C36	2.626(12)	C20	C21	1.339(15)
Yb1	C37	2.660(12)	C23	C24	1.411(16)
Yb1	C38	2.659(12)	C23	C28	1.423(17)
Yb1	C39	2.609(12)	C24	C25	1.414(17)
Yb1	C45	2.600(12)	C25	C26	1.400(18)
Yb1	C46	2.613(12)	C26	C27	1.401(19)
Yb1	C47	2.615(12)	C27	C28	1.426(18)
Yb1	C48	2.617(13)	C29	C30	1.374(17)
Yb1	C49	2.658(12)	C30	C31	1.39(2)

Atom	Atom	Length/Å	Atom	Atom	Length/Å
Yb2	N8	2.321(8)	C31	C32	1.42(2)
Yb2	N9	2.321(10)	C32	C33	1.412(18)
Yb2	C55	2.638(17)	C35	C36	1.455(17)
Yb2	C56	2.630(14)	C35	C39	1.404(17)
Yb2	C57	2.596(14)	C35	C41	1.477(16)
Yb2	C58	2.638(13)	C36	C37	1.382(17)
Yb2	C59	2.619(14)	C36	C42	1.467(17)
Yb2	C65	2.593(12)	C37	C38	1.385(17)
Yb2	C66	2.637(12)	C37	C43	1.517(17)
Yb2	C67	2.651(13)	C38	C39	1.433(17)
Yb2	C68	2.628(13)	C38	C44	1.530(18)
Yb2	C69	2.618(12)	C39	C40	1.537(17)
Pd1	N1	2.039(9)	C45	C46	1.431(18)
Pd1	N2	2.192(9)	C45	C49	1.396(18)
Pd1	N5	2.029(10)	C45	C51	1.485(17)
Pd1	C17	2.013(11)	C46	C47	1.363(16)
Pd2	N6	2.045(8)	C46	C52	1.492(18)
Pd2	N7	2.182(10)	C47	C48	1.425(17)
Pd2	N10	2.014(9)	C47	C53	1.554(19)
Pd2	C34	2.000(12)	C48	C49	1.407(17)
N1	C1	1.321(13)	C48	C54	1.535(17)
N1	C4	1.478(13)	C49	C50	1.525(18)
N2	C5	1.326(13)	C55	C56	1.39(2)
N2	C7	1.398(13)	C55	C59	1.39(2)
N3	C1	1.350(13)	C55	C61	1.53(2)
N3	C2	1.408(13)	C56	C57	1.410(19)
N4	C5	1.358(13)	C56	C62	1.553(19)
N4	C6	1.407(13)	C57	C58	1.38(2)
N5	C12	1.384(14)	C57	C63	1.511(19)
N5	C16	1.364(15)	C58	C59	1.410(19)
N6	C18	1.309(13)	C58	C64	1.561(19)
N6	C19	1.486(13)	C59	C60	1.59(2)
N7	C22	1.336(13)	C65	C66	1.409(17)
N7	C23	1.373(15)	C65	C69	1.438(16)
N8	C18	1.376(13)	C65	C71	1.520(18)
N8	C21	1.385(13)	C66	C67	1.456(19)
N9	C22	1.353(14)	C66	C72	1.511(16)
N9	C24	1.418(15)	C67	C68	1.415(15)
N10	C29	1.383(14)	C67	C73	1.507(17)
N10	C33	1.371(15)	C68	C69	1.408(17)
C1	C5	1.470(14)	C68	C74	1.512(16)
C2	C3	1.299(15)	C69	C70	1.527(15)
C3	C4	1.517(15)	O1	C75	1.49(3)
C4	C19	1.556(15)	O1	C78	1.38(3)

Atom	Atom	Length/Å	Atom	Atom	Length/Å
C6	C7	1.398(16)	C75	C76	1.41(2)
C6	C11	1.390(15)	C76	C77	1.63(3)
C7	C8	1.412(16)	C77	C78	1.37(2)
C8	C9	1.435(17)	O2	C79	1.30(2)
C9	C10	1.388(18)	O2	C82	1.42(2)
C10	C11	1.370(17)	C79	C80	1.40(2)
C12	C13	1.391(17)	C80	C81	1.46(2)
C13	C14	1.391(18)	C81	C82	1.48(2)
C14	C15	1.323(19)			

Table S7. *Bond Angles for **3**.*

Atom	Atom	Atom	Angle/˚	Atom	Atom	Atom	Angle/˚
N3	Yb1	N4	72.9(3)	C3	C4	C19	110.3(9)
N3	Yb1	C35	103.2(4)	N2	C5	Yb1	162.0(7)
N3	Yb1	C36	79.1(4)	N2	C5	N4	117.4(9)
N3	Yb1	C37	90.7(3)	N2	C5	C1	119.8(10)
N3	Yb1	C38	120.8(4)	N4	C5	Yb1	44.6(5)
N3	Yb1	C39	131.4(4)	N4	C5	C1	122.7(10)
N3	Yb1	C45	123.7(4)	C1	C5	Yb1	78.2(6)
N3	Yb1	C46	91.9(4)	C7	C6	N4	107.8(9)
N3	Yb1	C47	78.6(3)	C11	C6	N4	130.8(11)
N3	Yb1	C48	99.2(4)	C11	C6	C7	121.3(11)
N3	Yb1	C49	128.3(4)	N2	C7	C6	109.4(9)
N4	Yb1	C35	80.5(3)	N2	C7	C8	129.3(11)
N4	Yb1	C36	93.6(4)	C6	C7	C8	121.1(11)
N4	Yb1	C37	123.8(4)	C7	C8	C9	115.3(12)
N4	Yb1	C38	131.0(4)	C10	C9	C8	122.0(11)
N4	Yb1	C39	101.9(4)	C11	C10	C9	120.7(11)
N4	Yb1	C45	93.5(4)	C10	C11	C6	119.0(12)
N4	Yb1	C46	82.0(3)	N5	C12	C13	121.9(11)
N4	Yb1	C47	103.7(3)	C14	C13	C12	117.8(12)
N4	Yb1	C48	133.1(4)	C15	C14	C13	121.4(13)
N4	Yb1	C49	124.3(4)	C14	C15	C16	119.7(14)
C35	Yb1	C36	32.3(4)	N5	C16	C15	122.1(12)
C35	Yb1	C37	51.0(4)	N6	C18	Yb2	168.7(8)
C35	Yb1	C38	51.2(4)	N6	C18	N8	128.5(10)
C35	Yb1	C39	31.2(4)	N6	C18	C22	116.5(9)
C35	Yb1	C46	152.3(4)	N8	C18	Yb2	40.9(5)
C35	Yb1	C47	175.9(4)	N8	C18	C22	115.0(10)
C35	Yb1	C48	144.3(4)	C22	C18	Yb2	74.1(6)
C35	Yb1	C49	126.4(4)	N6	C19	C4	111.5(9)
C36	Yb1	C37	30.3(4)	C20	C19	N6	110.8(9)
C36	Yb1	C38	51.0(4)	C20	C19	C4	109.7(9)
C36	Yb1	C49	136.6(4)	C21	C20	C19	119.8(11)

Atom	Atom	Atom	Angle/˚	Atom	Atom	Atom	Angle/˚
C38	Yb1	C37	30.2(4)	C20	C21	N8	123.3(10)
C39	Yb1	C36	52.6(4)	N7	C22	Yb2	160.6(8)
C39	Yb1	C37	51.3(4)	N7	C22	N9	117.5(10)
C39	Yb1	C38	31.5(4)	N7	C22	C18	120.2(10)
C39	Yb1	C46	136.1(4)	N9	C22	Yb2	43.2(6)
C39	Yb1	C47	145.6(4)	N9	C22	C18	122.1(10)
C39	Yb1	C48	115.7(4)	C18	C22	Yb2	79.0(6)
C39	Yb1	C49	95.4(4)	N7	C23	C24	108.5(10)
C45	Yb1	C35	128.7(4)	N7	C23	C28	131.5(11)
C45	Yb1	C36	157.1(4)	C24	C23	C28	120.0(12)
C45	Yb1	C37	136.7(4)	C23	C24	N9	108.5(11)
C45	Yb1	C38	109.3(4)	C23	C24	C25	120.4(12)
C45	Yb1	C39	104.7(4)	C25	C24	N9	131.0(12)
C45	Yb1	C46	31.9(4)	C26	C25	C24	118.9(13)
C45	Yb1	C47	51.3(4)	C25	C26	C27	122.1(13)
C45	Yb1	C48	51.8(4)	C26	C27	C28	119.0(13)
C45	Yb1	C49	30.8(4)	C23	C28	C27	119.4(12)
C46	Yb1	C36	170.9(4)	C30	C29	N10	122.2(12)
C46	Yb1	C37	153.5(4)	C29	C30	C31	121.2(13)
C46	Yb1	C38	137.2(4)	C30	C31	C32	118.7(13)
C46	Yb1	C47	30.2(4)	C33	C32	C31	116.7(14)
C46	Yb1	C48	51.6(4)	N10	C33	C32	124.8(12)
C46	Yb1	C49	51.2(4)	C36	C35	Yb1	74.5(6)
C47	Yb1	C36	146.3(4)	C36	C35	C41	123.5(12)
C47	Yb1	C37	125.6(4)	C39	C35	Yb1	74.4(7)
C47	Yb1	C38	124.6(4)	C39	C35	C36	108.3(11)
C47	Yb1	C48	31.6(4)	C39	C35	C41	127.6(12)
C47	Yb1	C49	50.9(4)	C41	C35	Yb1	123.5(8)
C48	Yb1	C36	131.1(4)	C35	C36	Yb1	73.2(6)
C48	Yb1	C37	101.9(4)	C35	C36	C42	127.9(13)
C48	Yb1	C38	93.4(4)	C37	C36	Yb1	76.2(7)
C48	Yb1	C49	30.9(4)	C37	C36	C35	106.1(11)
C49	Yb1	C37	108.3(4)	C37	C36	C42	125.3(12)
C49	Yb1	C38	86.0(4)	C42	C36	Yb1	123.3(9)
N8	Yb2	C55	93.4(4)	C36	C37	Yb1	73.5(7)
N8	Yb2	C56	80.0(4)	C36	C37	C38	110.7(11)
N8	Yb2	C57	100.7(4)	C36	C37	C43	123.6(12)
N8	Yb2	C58	129.4(4)	C38	C37	Yb1	74.9(7)
N8	Yb2	C59	124.1(4)	C38	C37	C43	125.5(13)
N8	Yb2	C65	131.8(4)	C43	C37	Yb1	122.4(8)
N8	Yb2	C66	120.7(4)	C37	C38	Yb1	74.9(7)
N8	Yb2	C67	88.9(4)	C37	C38	C39	108.0(11)
N8	Yb2	C68	79.3(3)	C37	C38	C44	125.5(13)
N8	Yb2	C69	103.2(4)	C39	C38	Yb1	72.3(7)

Atom	Atom	Atom	Angle/˚	Atom	Atom	Atom	Angle/˚
N9	Yb2	N8	73.2(3)	C39	C38	C44	125.4(12)
N9	Yb2	C55	79.6(4)	C44	C38	Yb1	128.0(9)
N9	Yb2	C56	101.8(4)	C35	C39	Yb1	74.4(7)
N9	Yb2	C57	130.5(4)	C35	C39	C38	106.8(11)
N9	Yb2	C58	121.9(4)	C35	C39	C40	125.1(13)
N9	Yb2	C59	90.8(4)	C38	C39	Yb1	76.1(7)
N9	Yb2	C65	103.2(4)	C38	C39	C40	127.7(13)
N9	Yb2	C66	132.0(4)	C40	C39	Yb1	121.2(9)
N9	Yb2	C67	123.7(4)	C46	C45	Yb1	74.6(7)
N9	Yb2	C68	92.6(4)	C46	C45	C51	127.9(13)
N9	Yb2	C69	80.9(4)	C49	C45	Yb1	76.9(7)
C55	Yb2	C58	50.8(4)	C49	C45	C46	107.5(11)
C55	Yb2	C67	156.0(4)	C49	C45	C51	123.9(13)
C56	Yb2	C55	30.6(4)	C51	C45	Yb1	122.2(9)
C56	Yb2	C58	50.6(4)	C45	C46	Yb1	73.6(7)
C56	Yb2	C66	125.1(4)	C45	C46	C52	124.5(13)
C56	Yb2	C67	127.5(4)	C47	C46	Yb1	75.0(7)
C57	Yb2	C55	51.3(5)	C47	C46	C45	107.8(11)
C57	Yb2	C56	31.3(4)	C47	C46	C52	127.5(14)
C57	Yb2	C58	30.5(4)	C52	C46	Yb1	121.0(9)
C57	Yb2	C59	51.2(5)	C46	C47	Yb1	74.8(8)
C57	Yb2	C66	94.1(4)	C46	C47	C48	109.5(11)
C57	Yb2	C67	104.8(4)	C46	C47	C53	126.2(12)
C57	Yb2	C68	135.5(4)	C48	C47	Yb1	74.2(7)
C57	Yb2	C69	145.3(4)	C48	C47	C53	124.0(13)
C58	Yb2	C67	110.8(4)	C53	C47	Yb1	122.3(8)
C59	Yb2	C55	30.7(4)	C47	C48	Yb1	74.1(7)
C59	Yb2	C56	50.7(5)	C47	C48	C54	125.4(12)
C59	Yb2	C58	31.1(4)	C49	C48	Yb1	76.1(7)
C59	Yb2	C66	109.9(5)	C49	C48	C47	106.3(11)
C59	Yb2	C67	139.6(4)	C49	C48	C54	127.8(12)
C59	Yb2	C68	156.3(4)	C54	C48	Yb1	120.9(9)
C65	Yb2	C55	134.0(5)	C45	C49	Yb1	72.3(7)
C65	Yb2	C56	144.3(4)	C45	C49	C48	108.7(11)
C65	Yb2	C57	114.9(4)	C45	C49	C50	124.7(12)
C65	Yb2	C58	94.2(4)	C48	C49	Yb1	72.9(7)
C65	Yb2	C59	103.8(5)	C48	C49	C50	126.2(13)
C65	Yb2	C66	31.3(4)	C50	C49	Yb1	126.8(9)
C65	Yb2	C67	52.3(4)	C56	C55	Yb2	74.4(9)
C65	Yb2	C68	52.6(4)	C56	C55	C59	107.9(13)
C65	Yb2	C69	32.0(4)	C56	C55	C61	128.5(16)
C66	Yb2	C55	137.1(4)	C59	C55	Yb2	73.9(10)
C66	Yb2	C58	86.3(4)	C59	C55	C61	123.2(16)
C66	Yb2	C67	32.0(4)	C61	C55	Yb2	123.4(11)

Atom	Atom	Atom	Angle/˚	Atom	Atom	Atom	Angle/˚
C68	Yb2	C55	170.7(4)	C55	C56	Yb2	75.0(10)
C68	Yb2	C56	150.0(4)	C55	C56	C57	108.1(13)
C68	Yb2	C58	138.5(4)	C55	C56	C62	125.7(13)
C68	Yb2	C66	52.3(4)	C57	C56	Yb2	73.0(8)
C68	Yb2	C67	31.1(3)	C57	C56	C62	126.1(15)
C69	Yb2	C55	149.4(5)	C62	C56	Yb2	122.1(9)
C69	Yb2	C56	176.3(4)	C56	C57	Yb2	75.7(8)
C69	Yb2	C58	125.8(4)	C56	C57	C63	125.0(15)
C69	Yb2	C59	127.2(5)	C58	C57	Yb2	76.5(8)
C69	Yb2	C66	51.9(4)	C58	C57	C56	107.9(13)
C69	Yb2	C67	51.4(4)	C58	C57	C63	126.4(14)
C69	Yb2	C68	31.1(4)	C63	C57	Yb2	121.7(10)
N1	Pd1	N2	79.5(3)	C57	C58	Yb2	73.1(8)
N5	Pd1	N1	177.1(4)	C57	C58	C59	108.0(13)
N5	Pd1	N2	100.2(3)	C57	C58	C64	128.0(14)
C17	Pd1	N1	92.8(4)	C59	C58	Yb2	73.7(8)
C17	Pd1	N2	168.5(4)	C59	C58	C64	123.3(14)
C17	Pd1	N5	87.9(5)	C64	C58	Yb2	126.3(11)
N6	Pd2	N7	80.2(3)	C55	C59	Yb2	75.4(9)
N10	Pd2	N6	177.9(4)	C55	C59	C58	107.9(14)
N10	Pd2	N7	98.6(4)	C55	C59	C60	124.7(15)
C34	Pd2	N6	93.1(4)	C58	C59	Yb2	75.2(8)
C34	Pd2	N7	168.6(4)	C58	C59	C60	126.1(14)
C34	Pd2	N10	88.3(5)	C60	C59	Yb2	125.6(11)
C1	N1	Pd1	115.0(7)	C66	C65	Yb2	76.1(7)
C1	N1	C4	115.5(9)	C66	C65	C69	107.7(12)
C4	N1	Pd1	129.1(7)	C66	C65	C71	126.8(11)
C5	N2	Pd1	107.7(7)	C69	C65	Yb2	74.9(7)
C5	N2	C7	102.8(9)	C69	C65	C71	124.8(12)
C7	N2	Pd1	147.8(8)	C71	C65	Yb2	122.8(10)
C1	N3	Yb1	116.9(7)	C65	C66	Yb2	72.7(7)
C1	N3	C2	112.1(9)	C65	C66	C67	107.6(10)
C2	N3	Yb1	129.0(7)	C65	C66	C72	127.1(13)
C5	N4	Yb1	111.6(7)	C67	C66	Yb2	74.5(7)
C5	N4	C6	102.5(9)	C67	C66	C72	123.4(13)
C6	N4	Yb1	145.8(7)	C72	C66	Yb2	130.9(10)
C12	N5	Pd1	121.2(8)	C66	C67	Yb2	73.5(8)
C16	N5	Pd1	121.9(8)	C66	C67	C73	123.3(11)
C16	N5	C12	116.9(10)	C68	C67	Yb2	73.6(7)
C18	N6	Pd2	113.9(7)	C68	C67	C66	107.7(11)
C18	N6	C19	115.7(8)	C68	C67	C73	128.4(12)
C19	N6	Pd2	130.3(6)	C73	C67	Yb2	125.3(9)
C22	N7	Pd2	106.0(8)	C67	C68	Yb2	75.3(7)
C22	N7	C23	104.0(9)	C67	C68	C74	125.3(11)

Atom	Atom	Atom	Angle/˚	Atom	Atom	Atom	Angle/˚
C23	N7	Pd2	146.9(7)	C69	C68	Yb2	74.0(7)
C18	N8	Yb2	116.2(7)	C69	C68	C67	108.2(10)
C18	N8	C21	112.8(9)	C69	C68	C74	126.1(10)
C21	N8	Yb2	128.6(7)	C74	C68	Yb2	123.0(8)
C22	N9	Yb2	113.3(7)	C65	C69	Yb2	73.0(7)
C22	N9	C24	101.4(9)	C65	C69	C70	125.3(12)
C24	N9	Yb2	145.3(8)	C68	C69	Yb2	74.8(7)
C29	N10	Pd2	121.8(8)	C68	C69	C65	108.8(10)
C33	N10	Pd2	121.6(8)	C68	C69	C70	125.9(11)
C33	N10	C29	116.2(10)	C70	C69	Yb2	121.4(8)
N1	C1	Yb1	168.9(7)	C78	O1	C75	109(2)
N1	C1	N3	128.5(9)	C76	C75	O1	108(2)
N1	C1	C5	116.0(10)	C75	C76	C77	101(2)
N3	C1	Yb1	40.8(5)	C78	C77	C76	105(2)
N3	C1	C5	115.5(10)	C77	C78	O1	110(2)
C5	C1	Yb1	74.8(6)	C79	O2	C82	108.0(14)
C3	C2	N3	125.3(11)	O2	C79	C80	112.6(19)
C2	C3	C4	117.7(10)	C79	C80	C81	106.0(17)
N1	C4	C3	110.0(9)	C80	C81	C82	104.0(16)
N1	C4	C19	111.8(9)	O2	C82	C81	105.5(15)

Table S8. *Bond Lengths for* ***4****.*

Atom	Atom	Length/Å	Atom	Atom	Length/Å
Sm1	Pd1	2.9078(3)	C7	C8	1.420(6)
Sm1	N1	2.386(4)	C7	C12	1.498(7)
Sm1	C1	2.861(4)	C8	C9	1.411(7)
Sm1	C6	2.711(4)	C8	C13	1.514(6)
Sm1	C7	2.710(5)	C9	C10	1.405(6)
Sm1	C8	2.754(4)	C9	C14	1.507(6)
Sm1	C9	2.746(4)	C10	C15	1.497(6)
Sm1	C10	2.734(4)	C16	C17	1.403(6)
Sm1	C16	2.693(4)	C16	C20	1.418(7)
Sm1	C17	2.712(5)	C16	C21	1.513(7)
Sm1	C18	2.743(4)	C17	C18	1.411(7)
Sm1	C19	2.762(5)	C17	C22	1.520(6)
Sm1	C20	2.707(4)	C18	C19	1.407(6)
Pd1	C1	2.080(5)	C18	C23	1.500(7)
Pd1	$C1^1$	2.080(5)	C19	C20	1.399(7)
N1	C1	1.348(6)	C19	C24	1.527(7)
N1	C5	1.351(6)	C20	C25	1.508(6)
C1	C2	1.431(6)	C26	C27	1.458(8)
C2	C3	1.355(7)	C27	C28	1.378(7)

Atom	Atom	Length/Å	Atom	Atom	Length/Å
C3	C4	1.391(8)	C27	C32	1.389(7)
C4	C5	1.369(6)	C28	C29	1.381(8)
C6	C7	1.422(6)	C29	C30	1.372(8)
C6	C10	1.417(6)	C30	C31	1.346(8)
C6	C11	1.528(6)	C31	C32	1.362(8)

[1]1-X,+Y,3/2-Z

Table S9. *Bond Angles for **4**.*

Atom	Atom	Atom	Angle/˚	Atom	Atom	Atom	Angle/˚
N1	Sm1	Pd1	70.16(10)	C1	Pd1	C1[1]	179.8(2)
N1	Sm1	C1	27.95(13)	C1	N1	Sm1	96.0(3)
N1	Sm1	C6	78.94(13)	C1	N1	C5	122.3(4)
N1	Sm1	C7	87.61(14)	C5	N1	Sm1	141.4(3)
N1	Sm1	C8	117.65(14)	Pd1	C1	Sm1	70.07(12)
N1	Sm1	C9	128.12(13)	N1	C1	Sm1	56.0(2)
N1	Sm1	C10	102.58(14)	N1	C1	Pd1	126.0(3)
N1	Sm1	C16	113.17(14)	N1	C1	C2	115.1(4)
N1	Sm1	C17	84.23(14)	C2	C1	Sm1	170.0(4)
N1	Sm1	C18	80.79(14)	C2	C1	Pd1	118.9(4)
N1	Sm1	C19	107.09(15)	C3	C2	C1	122.6(5)
N1	Sm1	C20	129.78(14)	C2	C3	C4	120.2(5)
C1	Sm1	Pd1	42.26(10)	C5	C4	C3	116.4(5)
C6	Sm1	Pd1	93.98(11)	N1	C5	C4	123.4(5)
C6	Sm1	C1	82.86(13)	C7	C6	Sm1	74.7(3)
C6	Sm1	C8	49.31(13)	C7	C6	C11	125.0(5)
C6	Sm1	C9	49.21(14)	C10	C6	Sm1	75.8(3)
C6	Sm1	C10	30.16(13)	C10	C6	C7	108.9(4)
C6	Sm1	C17	159.99(16)	C10	C6	C11	125.8(5)
C6	Sm1	C18	134.53(16)	C11	C6	Sm1	121.1(3)
C6	Sm1	C19	126.77(15)	C6	C7	Sm1	74.8(3)
C7	Sm1	Pd1	124.16(11)	C6	C7	C12	127.2(4)
C7	Sm1	C1	103.41(13)	C8	C7	Sm1	76.6(3)
C7	Sm1	C6	30.42(14)	C8	C7	C6	106.7(4)
C7	Sm1	C8	30.11(13)	C8	C7	C12	125.8(5)
C7	Sm1	C9	49.74(14)	C12	C7	Sm1	118.6(3)
C7	Sm1	C10	50.20(15)	C7	C8	Sm1	73.2(3)
C7	Sm1	C17	138.96(16)	C7	C8	C13	124.6(5)
C7	Sm1	C18	109.00(16)	C9	C8	Sm1	74.8(3)
C7	Sm1	C19	96.36(15)	C9	C8	C7	108.3(4)
C8	Sm1	Pd1	133.79(10)	C9	C8	C13	125.7(4)
C8	Sm1	C1	131.36(13)	C13	C8	Sm1	128.5(3)
C8	Sm1	C19	85.38(14)	C8	C9	Sm1	75.4(3)

Atom	Atom	Atom	Angle/˚	Atom	Atom	Atom	Angle/˚
C9	Sm1	Pd1	107.07(11)	C8	C9	C14	124.7(4)
C9	Sm1	C1	124.73(13)	C10	C9	Sm1	74.7(3)
C9	Sm1	C8	29.73(14)	C10	C9	C8	108.8(4)
C9	Sm1	C19	105.84(14)	C10	C9	C14	126.0(5)
C10	Sm1	Pd1	84.65(10)	C14	C9	Sm1	123.0(3)
C10	Sm1	C1	95.03(13)	C6	C10	Sm1	74.0(3)
C10	Sm1	C8	49.30(14)	C6	C10	C15	126.4(4)
C10	Sm1	C9	29.70(13)	C9	C10	Sm1	75.6(3)
C10	Sm1	C18	158.09(16)	C9	C10	C6	107.3(4)
C10	Sm1	C19	133.83(14)	C9	C10	C15	125.8(5)
C16	Sm1	Pd1	92.55(11)	C15	C10	Sm1	122.4(3)
C16	Sm1	C1	108.89(14)	C17	C16	Sm1	75.7(3)
C16	Sm1	C6	167.66(14)	C17	C16	C20	108.0(5)
C16	Sm1	C7	142.80(15)	C17	C16	C21	124.9(5)
C16	Sm1	C8	119.50(14)	C20	C16	Sm1	75.3(3)
C16	Sm1	C9	118.70(15)	C20	C16	C21	127.0(5)
C16	Sm1	C10	140.82(16)	C21	C16	Sm1	118.0(3)
C16	Sm1	C17	30.08(14)	C16	C17	Sm1	74.2(3)
C16	Sm1	C18	49.63(15)	C16	C17	C18	108.4(4)
C16	Sm1	C19	49.16(15)	C16	C17	C22	126.4(5)
C16	Sm1	C20	30.45(14)	C18	C17	Sm1	76.3(3)
C17	Sm1	Pd1	90.39(11)	C18	C17	C22	125.0(5)
C17	Sm1	C1	87.35(13)	C22	C17	Sm1	119.6(3)
C17	Sm1	C8	134.22(14)	C17	C18	Sm1	73.8(3)
C17	Sm1	C9	146.62(14)	C17	C18	C23	125.8(5)
C17	Sm1	C10	169.58(16)	C19	C18	Sm1	75.9(3)
C17	Sm1	C18	29.97(14)	C19	C18	C17	107.2(4)
C17	Sm1	C19	48.95(14)	C19	C18	C23	126.4(5)
C18	Sm1	Pd1	116.43(11)	C23	C18	Sm1	123.1(3)
C18	Sm1	C1	97.23(14)	C18	C19	Sm1	74.5(3)
C18	Sm1	C8	109.75(15)	C18	C19	C24	122.9(5)
C18	Sm1	C9	134.57(15)	C20	C19	Sm1	73.0(3)
C18	Sm1	C19	29.61(13)	C20	C19	C18	109.1(5)
C19	Sm1	Pd1	138.58(11)	C20	C19	C24	127.5(5)
C19	Sm1	C1	126.72(14)	C24	C19	Sm1	125.7(3)
C20	Sm1	Pd1	121.00(12)	C16	C20	Sm1	74.2(3)
C20	Sm1	C1	136.82(14)	C16	C20	C25	125.4(5)
C20	Sm1	C6	139.22(15)	C19	C20	Sm1	77.4(3)
C20	Sm1	C7	112.69(16)	C19	C20	C16	107.3(4)
C20	Sm1	C8	89.93(14)	C19	C20	C25	126.7(5)
C20	Sm1	C9	97.12(14)	C25	C20	Sm1	120.9(3)
C20	Sm1	C10	126.08(14)	C28	C27	C26	121.5(6)
C20	Sm1	C17	49.81(14)	C28	C27	C32	118.5(6)
C20	Sm1	C18	49.58(14)	C32	C27	C26	120.0(6)

Atom	Atom	Atom	Angle/˚	Atom	Atom	Atom	Angle/˚
C20	Sm1	C19	29.63(15)	C27	C28	C29	120.9(5)
Sm1[1]	Pd1	Sm1	175.61(2)	C30	C29	C28	119.3(6)
C1	Pd1	Sm1[1]	112.32(13)	C31	C30	C29	119.8(6)
C1[1]	Pd1	Sm1	112.32(13)	C30	C31	C32	122.1(6)
C1[1]	Pd1	Sm1[1]	67.67(13)	C31	C32	C27	119.4(5)
C1	Pd1	Sm1	67.67(13)				

[1]1-X,+Y,3/2-Z

Table S10. *Bond Lengths for **5**.*

Atom	Atom	Length/Å	Atom	Atom	Length/Å
Yb1	Pd1	2.92441(15)	C7	C8	1.416(5)
Yb1	N1	2.275(3)	C7	C12	1.506(5)
Yb1	C1	2.772(4)	C8	C9	1.423(5)
Yb1	C6	2.612(3)	C8	C13	1.512(5)
Yb1	C7	2.668(4)	C9	C10	1.414(5)
Yb1	C8	2.643(4)	C9	C14	1.503(5)
Yb1	C9	2.643(3)	C10	C15	1.502(5)
Yb1	C10	2.620(3)	C16	C17	1.410(6)
Yb1	C16	2.643(3)	C16	C20	1.420(5)
Yb1	C17	2.655(3)	C16	C21	1.498(5)
Yb1	C18	2.624(4)	C17	C18	1.423(5)
Yb1	C19	2.604(3)	C17	C22	1.505(5)
Yb1	C20	2.623(4)	C18	C19	1.414(6)
Pd1	C1	2.079(4)	C18	C23	1.508(5)
Pd1	C1[1]	2.079(4)	C19	C20	1.412(5)
N1	C1	1.359(5)	C19	C24	1.515(5)
N1	C5	1.352(5)	C20	C25	1.498(6)
C1	C2	1.417(5)	C26	C27	1.388(7)
C2	C3	1.379(6)	C26	C31	1.381(6)
C3	C4	1.390(7)	C26	C32	1.487(8)
C4	C5	1.368(6)	C27	C28	1.378(7)
C6	C7	1.424(5)	C28	C29	1.369(7)
C6	C10	1.424(5)	C29	C30	1.366(8)
C6	C11	1.501(5)	C30	C31	1.397(7)

[1]1-X,+Y,3/2-Z

*Table S11. Bond Angles for **5**.*

Atom	Atom	Atom	Angle/˚	Atom	Atom	Atom	Angle/˚
N1	Yb1	Pd1	71.84(8)	C1	Pd1	C1[1]	178.90(18)
N1	Yb1	C1	29.18(11)	C1	N1	Yb1	96.2(2)
N1	Yb1	C6	87.95(11)	C5	N1	Yb1	141.5(3)
N1	Yb1	C7	119.09(11)	C5	N1	C1	122.2(3)
N1	Yb1	C8	130.39(11)	Pd1	C1	Yb1	72.56(11)
N1	Yb1	C9	103.17(11)	N1	C1	Yb1	54.66(18)
N1	Yb1	C10	78.71(11)	N1	C1	Pd1	127.1(3)
N1	Yb1	C16	80.98(11)	N1	C1	C2	116.0(3)
N1	Yb1	C17	107.98(12)	C2	C1	Yb1	170.0(3)
N1	Yb1	C18	131.99(11)	C2	C1	Pd1	116.9(3)
N1	Yb1	C19	114.75(12)	C3	C2	C1	121.9(4)
N1	Yb1	C20	84.62(11)	C2	C3	C4	119.6(4)
C1	Yb1	Pd1	42.70(8)	C5	C4	C3	117.5(4)
C6	Yb1	Pd1	125.07(8)	N1	C5	C4	122.8(4)
C6	Yb1	C1	104.63(11)	C7	C6	Yb1	76.5(2)
C6	Yb1	C7	31.26(11)	C7	C6	C10	108.0(3)
C6	Yb1	C8	51.76(11)	C7	C6	C11	125.4(3)
C6	Yb1	C9	51.80(11)	C10	C6	Yb1	74.5(2)
C6	Yb1	C10	31.59(11)	C10	C6	C11	126.4(3)
C6	Yb1	C16	110.03(12)	C11	C6	Yb1	119.0(2)
C6	Yb1	C17	95.86(12)	C6	C7	Yb1	72.2(2)
C6	Yb1	C18	112.61(12)	C6	C7	C12	125.3(3)
C6	Yb1	C20	141.30(12)	C8	C7	Yb1	73.6(2)
C7	Yb1	Pd1	134.10(8)	C8	C7	C6	107.8(3)
C7	Yb1	C1	133.61(11)	C8	C7	C12	125.2(4)
C8	Yb1	Pd1	105.82(8)	C12	C7	Yb1	131.7(3)
C8	Yb1	C1	125.89(11)	C7	C8	Yb1	75.5(2)
C8	Yb1	C7	30.93(11)	C7	C8	C9	108.2(3)
C8	Yb1	C9	31.23(11)	C7	C8	C13	125.0(3)
C8	Yb1	C17	104.22(12)	C9	C8	Yb1	74.4(2)
C9	Yb1	Pd1	83.18(8)	C9	C8	C13	125.5(3)
C9	Yb1	C1	94.69(11)	C13	C8	Yb1	126.6(3)
C9	Yb1	C7	51.31(11)	C8	C9	Yb1	74.4(2)
C9	Yb1	C17	133.80(12)	C8	C9	C14	124.7(3)
C10	Yb1	Pd1	93.59(8)	C10	C9	Yb1	73.52(19)
C10	Yb1	C1	82.66(11)	C10	C9	C8	108.1(3)
C10	Yb1	C7	51.63(11)	C10	C9	C14	126.8(4)
C10	Yb1	C8	51.72(11)	C14	C9	Yb1	124.2(2)
C10	Yb1	C9	31.16(12)	C6	C10	Yb1	73.9(2)
C10	Yb1	C16	136.31(12)	C6	C10	C15	126.0(3)
C10	Yb1	C17	127.45(12)	C9	C10	Yb1	75.3(2)
C10	Yb1	C18	139.37(12)	C9	C10	C6	108.0(3)
C10	Yb1	C20	161.71(12)	C9	C10	C15	125.5(3)

Atom	Atom	Atom	Angle/°	Atom	Atom	Atom	Angle/°
C16	Yb1	Pd1	115.99(9)	C15	C10	Yb1	123.1(2)
C16	Yb1	C1	97.49(11)	C17	C16	Yb1	75.0(2)
C16	Yb1	C7	109.85(12)	C17	C16	C20	108.1(3)
C16	Yb1	C8	134.70(12)	C17	C16	C21	126.2(4)
C16	Yb1	C9	160.50(12)	C20	C16	Yb1	73.6(2)
C16	Yb1	C17	30.87(12)	C20	C16	C21	125.0(4)
C17	Yb1	Pd1	138.67(9)	C21	C16	Yb1	124.7(3)
C17	Yb1	C1	128.22(11)	C16	C17	Yb1	74.1(2)
C17	Yb1	C7	83.56(12)	C16	C17	C18	108.0(3)
C18	Yb1	Pd1	118.89(9)	C16	C17	C22	123.5(4)
C18	Yb1	C1	137.66(11)	C18	C17	Yb1	73.2(2)
C18	Yb1	C7	87.77(12)	C18	C17	C22	127.3(4)
C18	Yb1	C8	93.67(12)	C22	C17	Yb1	128.6(3)
C18	Yb1	C9	123.91(12)	C17	C18	Yb1	75.6(2)
C18	Yb1	C16	51.58(12)	C17	C18	C23	126.8(4)
C18	Yb1	C17	31.28(12)	C19	C18	Yb1	73.5(2)
C19	Yb1	Pd1	89.80(9)	C19	C18	C17	107.7(3)
C19	Yb1	C1	108.40(11)	C19	C18	C23	124.8(4)
C19	Yb1	C6	143.86(12)	C23	C18	Yb1	124.1(3)
C19	Yb1	C7	117.98(12)	C18	C19	Yb1	75.1(2)
C19	Yb1	C8	114.78(12)	C18	C19	C24	125.6(4)
C19	Yb1	C9	137.01(12)	C20	C19	Yb1	75.1(2)
C19	Yb1	C10	166.48(12)	C20	C19	C18	108.3(3)
C19	Yb1	C16	51.72(12)	C20	C19	C24	125.7(4)
C19	Yb1	C17	51.66(12)	C24	C19	Yb1	121.4(2)
C19	Yb1	C18	31.39(12)	C16	C20	Yb1	75.1(2)
C19	Yb1	C20	31.34(12)	C16	C20	C25	124.9(4)
C20	Yb1	Pd1	88.27(8)	C19	C20	Yb1	73.6(2)
C20	Yb1	C1	86.47(11)	C19	C20	C16	107.9(3)
C20	Yb1	C7	134.67(12)	C19	C20	C25	127.0(4)
C20	Yb1	C8	144.63(12)	C25	C20	Yb1	121.6(3)
C20	Yb1	C9	165.92(12)	C27	C26	C32	120.8(5)
C20	Yb1	C16	31.28(12)	C31	C26	C27	119.2(5)
C20	Yb1	C17	51.45(12)	C31	C26	C32	120.0(5)
C20	Yb1	C18	51.77(12)	C28	C27	C26	119.8(4)
Yb1	Pd1	Yb1[1]	177.247(15)	C29	C28	C27	121.3(5)
C1	Pd1	Yb1[1]	115.29(10)	C30	C29	C28	119.5(5)
C1[1]	Pd1	Yb1[1]	64.74(10)	C29	C30	C31	120.3(5)
C1	Pd1	Yb1	64.74(10)	C26	C31	C30	120.0(5)
C1[1]	Pd1	Yb1	115.29(10)				

[1]1-X,+Y,3/2-Z

*Table S12. Bond Lengths for **6**.*

Atom	Atom	Length/Å	Atom	Atom	Length/Å
Yb1	Pd1	2.8766(3)	C5	C6	1.408(3)
Yb1	C1	2.559(2)	C5	C10	1.497(4)
Yb1	C2	2.552(2)	C6	C7	1.419(3)
Yb1	C13	2.622(2)	C6	C11	1.492(4)
Yb1	C14	2.626(2)	C7	C12	1.496(3)
Yb1	C15	2.629(2)	C13	C14	1.426(4)
Yb1	C16	2.638(2)	C13	C17	1.414(3)
Yb1	C17	2.627(2)	C13	C18	1.501(3)
Yb1	C23	2.606(2)	C14	C15	1.418(4)
Yb1	C24	2.611(2)	C14	C19	1.496(4)
Yb1	C25	2.615(2)	C15	C16	1.426(3)
Yb1	C26	2.639(2)	C15	C20	1.503(3)
Yb1	C27	2.637(2)	C16	C17	1.414(3)
Pd1	C1	2.123(2)	C16	C21	1.499(3)
Pd1	C2	2.123(2)	C17	C22	1.505(3)
Pd1	C3	2.425(2)	C23	C24	1.420(4)
Pd1	C4	2.413(2)	C23	C27	1.416(4)
Pd1	C5	2.303(2)	C23	C28	1.499(4)
Pd1	C6	2.352(2)	C24	C25	1.413(3)
Pd1	C7	2.271(2)	C24	C29	1.502(4)
C3	C4	1.382(4)	C25	C26	1.415(3)
C3	C7	1.457(3)	C25	C30	1.503(4)
C3	C8	1.492(4)	C26	C27	1.415(3)
C4	C5	1.467(4)	C26	C31	1.498(3)
C4	C9	1.497(4)	C27	C32	1.496(4)

*Table S13. Bond Angles for **6**.*

Atom	Atom	Atom	Angle/˚	Atom	Atom	Atom	Angle/˚
C1	Yb1	C13	84.89(8)	C7	Pd1	C6	35.70(8)
C1	Yb1	C14	115.72(8)	Pd1	C1	Yb1	75.18(7)
C1	Yb1	C15	130.91(7)	Pd1	C2	Yb1	75.31(7)
C1	Yb1	C16	105.68(7)	C4	C3	Pd1	72.91(13)
C1	Yb1	C17	79.39(7)	C4	C3	C7	107.2(2)
C1	Yb1	C23	91.97(8)	C4	C3	C8	128.9(3)
C1	Yb1	C24	123.56(8)	C7	C3	Pd1	66.24(12)
C1	Yb1	C25	130.48(8)	C7	C3	C8	123.9(3)
C1	Yb1	C26	101.21(8)	C8	C3	Pd1	124.14(18)
C1	Yb1	C27	79.56(8)	C3	C4	Pd1	73.90(14)
C2	Yb1	C1	90.78(7)	C3	C4	C5	107.9(2)
C2	Yb1	C13	85.06(7)	C3	C4	C9	127.9(3)
C2	Yb1	C14	79.02(7)	C5	C4	Pd1	67.85(12)

Atom	Atom	Atom	Angle/˚	Atom	Atom	Atom	Angle/˚
C2	Yb1	C15	105.42(7)	C5	C4	C9	124.2(3)
C2	Yb1	C16	130.71(7)	C9	C4	Pd1	124.30(18)
C2	Yb1	C17	115.73(7)	C4	C5	Pd1	76.01(14)
C2	Yb1	C23	130.22(7)	C4	C5	C10	124.1(3)
C2	Yb1	C24	121.86(8)	C6	C5	Pd1	74.30(13)
C2	Yb1	C25	90.50(7)	C6	C5	C4	108.5(2)
C2	Yb1	C26	78.97(7)	C6	C5	C10	126.5(3)
C2	Yb1	C27	101.33(8)	C10	C5	Pd1	124.01(17)
C13	Yb1	C14	31.53(8)	C5	C6	Pd1	70.51(13)
C13	Yb1	C15	51.95(7)	C5	C6	C7	106.4(2)
C13	Yb1	C16	51.83(7)	C5	C6	C11	127.3(2)
C13	Yb1	C17	31.26(8)	C7	C6	Pd1	69.03(12)
C13	Yb1	C26	162.93(7)	C7	C6	C11	126.3(2)
C13	Yb1	C27	163.22(8)	C11	C6	Pd1	124.03(17)
C14	Yb1	C15	31.32(8)	C3	C7	Pd1	77.82(13)
C14	Yb1	C16	51.86(7)	C3	C7	C12	124.0(2)
C14	Yb1	C17	51.76(8)	C6	C7	Pd1	75.27(13)
C14	Yb1	C26	136.85(8)	C6	C7	C3	109.0(2)
C14	Yb1	C27	164.71(8)	C6	C7	C12	125.4(2)
C15	Yb1	C16	31.41(7)	C12	C7	Pd1	125.01(17)
C15	Yb1	C26	127.03(7)	C14	C13	Yb1	74.37(13)
C15	Yb1	C27	138.09(8)	C14	C13	C18	125.5(2)
C16	Yb1	C26	138.55(7)	C17	C13	Yb1	74.58(12)
C17	Yb1	C15	51.71(7)	C17	C13	C14	107.7(2)
C17	Yb1	C16	31.16(7)	C17	C13	C18	126.6(2)
C17	Yb1	C26	165.29(7)	C18	C13	Yb1	121.52(16)
C17	Yb1	C27	137.24(8)	C13	C14	Yb1	74.10(12)
C23	Yb1	C13	144.67(8)	C13	C14	C19	127.5(2)
C23	Yb1	C14	140.95(8)	C15	C14	Yb1	74.49(13)
C23	Yb1	C15	109.67(8)	C15	C14	C13	108.0(2)
C23	Yb1	C16	95.97(8)	C15	C14	C19	123.9(2)
C23	Yb1	C17	113.61(8)	C19	C14	Yb1	124.83(16)
C23	Yb1	C24	31.58(8)	C14	C15	Yb1	74.19(13)
C23	Yb1	C25	52.17(7)	C14	C15	C16	108.0(2)
C23	Yb1	C26	51.79(7)	C14	C15	C20	123.9(2)
C23	Yb1	C27	31.34(8)	C16	C15	Yb1	74.61(12)
C24	Yb1	C13	136.85(8)	C16	C15	C20	127.3(2)
C24	Yb1	C14	114.96(8)	C20	C15	Yb1	125.20(16)
C24	Yb1	C15	86.59(8)	C15	C16	Yb1	73.97(12)
C24	Yb1	C16	87.10(7)	C15	C16	C21	127.4(2)
C24	Yb1	C17	115.76(8)	C17	C16	Yb1	74.02(12)
C24	Yb1	C25	31.37(8)	C17	C16	C15	107.6(2)
C24	Yb1	C26	51.60(7)	C17	C16	C21	123.8(2)
C24	Yb1	C27	51.73(8)	C21	C16	Yb1	127.46(16)

Atom	Atom	Atom	Angle/˚	Atom	Atom	Atom	Angle/˚
C25	Yb1	C13	144.47(8)	C13	C17	Yb1	74.16(12)
C25	Yb1	C14	113.08(8)	C13	C17	C22	127.0(2)
C25	Yb1	C15	96.06(7)	C16	C17	Yb1	74.82(12)
C25	Yb1	C16	110.43(7)	C16	C17	C13	108.7(2)
C25	Yb1	C17	141.58(8)	C16	C17	C22	123.5(2)
C25	Yb1	C26	31.25(8)	C22	C17	Yb1	125.00(16)
C25	Yb1	C27	51.76(8)	C24	C23	Yb1	74.42(13)
C27	Yb1	C16	126.96(8)	C24	C23	C28	126.1(3)
C27	Yb1	C26	31.10(8)	C27	C23	Yb1	75.56(13)
C1	Pd1	Yb1	59.30(7)	C27	C23	C24	107.7(2)
C1	Pd1	C3	102.60(9)	C27	C23	C28	125.5(3)
C1	Pd1	C4	134.91(10)	C28	C23	Yb1	123.55(17)
C1	Pd1	C5	149.06(9)	C23	C24	Yb1	74.00(13)
C1	Pd1	C6	115.20(9)	C23	C24	C29	124.3(3)
C1	Pd1	C7	90.66(9)	C25	C24	Yb1	74.47(13)
C2	Pd1	Yb1	59.13(6)	C25	C24	C23	108.3(2)
C2	Pd1	C1	117.93(9)	C25	C24	C29	126.0(3)
C2	Pd1	C3	126.26(9)	C29	C24	Yb1	128.33(17)
C2	Pd1	C4	96.28(9)	C24	C25	Yb1	74.16(13)
C2	Pd1	C5	92.53(9)	C24	C25	C26	107.8(2)
C2	Pd1	C6	121.83(9)	C24	C25	C30	127.6(2)
C2	Pd1	C7	151.33(9)	C26	C25	Yb1	75.33(13)
C3	Pd1	Yb1	148.13(6)	C26	C25	C30	124.2(2)
C4	Pd1	Yb1	147.66(6)	C30	C25	Yb1	121.96(16)
C4	Pd1	C3	33.19(9)	C25	C26	Yb1	73.43(12)
C5	Pd1	Yb1	148.86(6)	C25	C26	C31	125.6(2)
C5	Pd1	C3	58.24(9)	C27	C26	Yb1	74.37(13)
C5	Pd1	C4	36.14(10)	C27	C26	C25	108.2(2)
C5	Pd1	C6	35.19(9)	C27	C26	C31	125.6(2)
C6	Pd1	Yb1	150.76(6)	C31	C26	Yb1	124.67(16)
C6	Pd1	C3	58.66(9)	C23	C27	Yb1	73.10(13)
C6	Pd1	C4	58.63(9)	C23	C27	C32	126.3(3)
C7	Pd1	Yb1	149.46(6)	C26	C27	Yb1	74.53(12)
C7	Pd1	C3	35.95(8)	C26	C27	C23	108.0(2)
C7	Pd1	C4	58.30(8)	C26	C27	C32	125.2(3)
C7	Pd1	C5	59.32(8)	C32	C27	Yb1	124.45(17)

Table S14. *Main distances (in Å) and angles (in °) in the compound* ***1.*** *"pym" and "imid" designate the pyrimidine and the 2-(benzimidazol-2-yl) moieties on the ligand, respectively.*

Atoms	Av. Values (Å or °)
Pd-Me	2.021(3)
Pd-N_{py}	2.041(2)
Pd-N_{pym}	2.176(2)
Pd-N_{imid}	2.035(2)
Ligand plane^py	60.68°

Table S15. *Main distances (in Å) in the compounds* ***2*** *and* ***3****. "pym" and "imid" designate the pyrimidine and the 2-(benzimidazol-2-yl) moieties on the ligand, respectively.*

	Av. Values (Å)	
**Atoms**	**2**	**3**
Yb_1-N_{pym}	2.317(5)	2.328(9)
Yb_2-N_{pym}	2.318(5)	2.321(8)
Yb_1-N_{imid}	2.353(5)	2.365(10)
Yb_2-N_{imid}	2.324(5)	2.321(10)
Yb_1 – Cp^*_{ctr}	2.319	2.337
Yb_2 – Cp^*_{ctr}	2.336	2.334
Pd_1-N_{pym}	2.151(5)	2.039(9)
Pd_2-N_{pym}	2.175(5)	2.045(8)
Pd_1-N_{imid}	2.035(4)	2.192(9)
Pd_2-N_{imid}	2.030(5)	2.182(10)
Pd_1-N_{py}	2.027(5)	2.029(10)
Pd_2-N_{py}	2.026(5)	2.014(9)
Pd_1-C_{Me}	2.032(6)	2.013(11)
Pd_2-C_{Me}	2.022(6)	2.000(12)

Table S16. *Main distances (in Å) and angles (in °) in the compounds* ***4*** *and* ***5****.*

	Av. Values (Å or °)	
**Atoms**	**4 (Sm)**	**5 (Yb)**
Ln-Pd	2.9078(3)	2.9244(2)
Ln-$N_{pyridyl}$	2.386(4)	2.275(3)
Ln-$C_{pyridyl}$	2.861(4)	2.772(4)
Ln – Cp^*_{ctr}	2.449	2.341
Pd-$C_{pyridyl}$	2.080(5)	2.079(4)
<($C_{pyriyl1}$-Pd-$C_{pyridyl2}$)	178.87°	179.72°
<($Cp^*_{1\ ctr}$ -Ln- $Cp^*_{2\ ctr}$)	134.70°	134.52°

Table S17. *Main distances (in Å) in the compound* ***6****.*

**Atoms**	**Av. Values (Å)**
Yb-Pd	2.8766(3)
Pd-Me (avg)	2.123(2)
Yb-Me (avg)	2.556(2)
Yb-H (avg)	2.206
Pd-Cp^*_{ctr}	2.017
Yb-Cp^*_{ctr} (avg)	2.332

V. Theoretical calculations

DFT calculations were mainly done using the ORCA 4.2.1 software[10]. The geometry optimizations calculations were performed with the “Tight” convergence criterion using the PBE functional and the scalar relativistic ZORA Hamiltonian.[11] The ZORA-def2-TZVP basis set[12] was used for the geometry optimization calculations of **1** and for the single-point energy calculations. The ZORA-def2-SVP basis set[12] was used for main group elements (C, H, N) for the geometry optimization calculations of the lanthanide-bearing complexes. SARC/J auxiliary basis sets for Coulomb fitting were used in all cases.[13–15] Dispersion corrections were added to the functional used in the D3 framework proposed by Grimme with the addition of the Becke–Johnson damping (D3BJ) in all cases.[16,17] Frequencies were calculated (analytically for the precursors and numerically for the complexes containing lanthanides) to ensure these structures corresponded to energy minima. Single-point energy calculations starting from PBE optimized geometries were then performed in the gas phase using the PBE, PBE0[18], TPSSh[19,20] and ωB97X-D3[21] functionals associated with the D3BJ dispersion corrections.

The transition state calculations were performed at the DFT level using the ORCA software. The transition state was confirmed by the presence of one imaginary frequency of high numeric value.

Complete active space self-consistent field (CASSCF) calculations were performed using the OpenMolcas 18.09 software,[22,23] using a DKH2 Hamiltonian to account for relativistic effects and ANO-RCC-VDZP basis sets for all elements.[24] The Cholesky decomposition framework[25] was also used to accelerate the calculation of the two-electron integrals.

All quantum chemical topological analyses[26,27] were performed with the Multiwfn code[28] by analyzing the electron density generated from the Orca code. The analyses of both covalent and non-covalent interactions were calculated based on the Density Overlap Regions Indicator (DORI).[29] Calculations of bond critical points in the quantum theory of atoms in molecule (QTAIM) framework were performed[30,31] associated to the computation of several descriptors, such as the electron density ρ(r), the energy density H(r), and the Laplacian of the electron density to characterize the bonding.[32] Then, the electron localization function (ELF) was calculated[33] to analyze the valence basins.

The ADF program package[34] was used for molecular orbital and energy decomposition

1. Geometry optimisations and electronic structure

a) Compound **1**

Table S18. *Main distances (in Å) for the compound **1**, as observed in the crystal structure and in the DFT optimized geometry with several different density functionals. "pym" and "imid" designate the pyrimidine and the 2-(benzimidazol-2-yl) moieties on the ligand, respectively.*

Distance \ source	XRD structure	PBE-D3	ωB97X-D3	PBE0-D3
Pd-Me	2.022(3)	2.035	2.008	2.008
Pd-N_{py}	2.040(2)	2.041	2.086	2.049
Pd-N_{pym}	2.176(2)	2.162	2.203	2.166
Pd-N_{imid}	2.034(2)	2.020	2.019	2.005

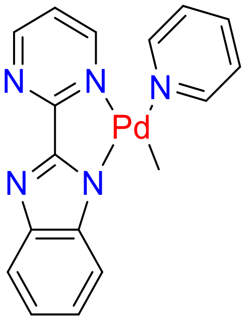

Table S19. *Orbital energies of **1** after single point energy calculation using geometry optimized at the DFT/PBE-D3 level of theory using several different density functionals.*

Calculation type / Orbital energy (eV)	PBE-D3	PBE0-D3	TPSSh-D3	ωB97X-D3
HOMO-2	-5.14	-6.42	-5.62	-8.56
HOMO-1	-4.95	-5.67	-5.17	-7.39
HOMO	-4.68	-5.55	-4.98	-7.37
LUMO	-3.01	-2.22	-2.56	-0.33
LUMO+1	-2.57	-1.78	-2.11	0.003
LUMO+2	-2.51	-1.67	-2.02	0.23
HOMO-LUMO gap	1.66	3.34	2.41	7.07
HOMO-LUMO gap (kcal/mol)	38.34	76.92	55.63	163.09
LUMO-LUMO+1 gap (kcal/mol)	10.25	10.10	10.44	7.56

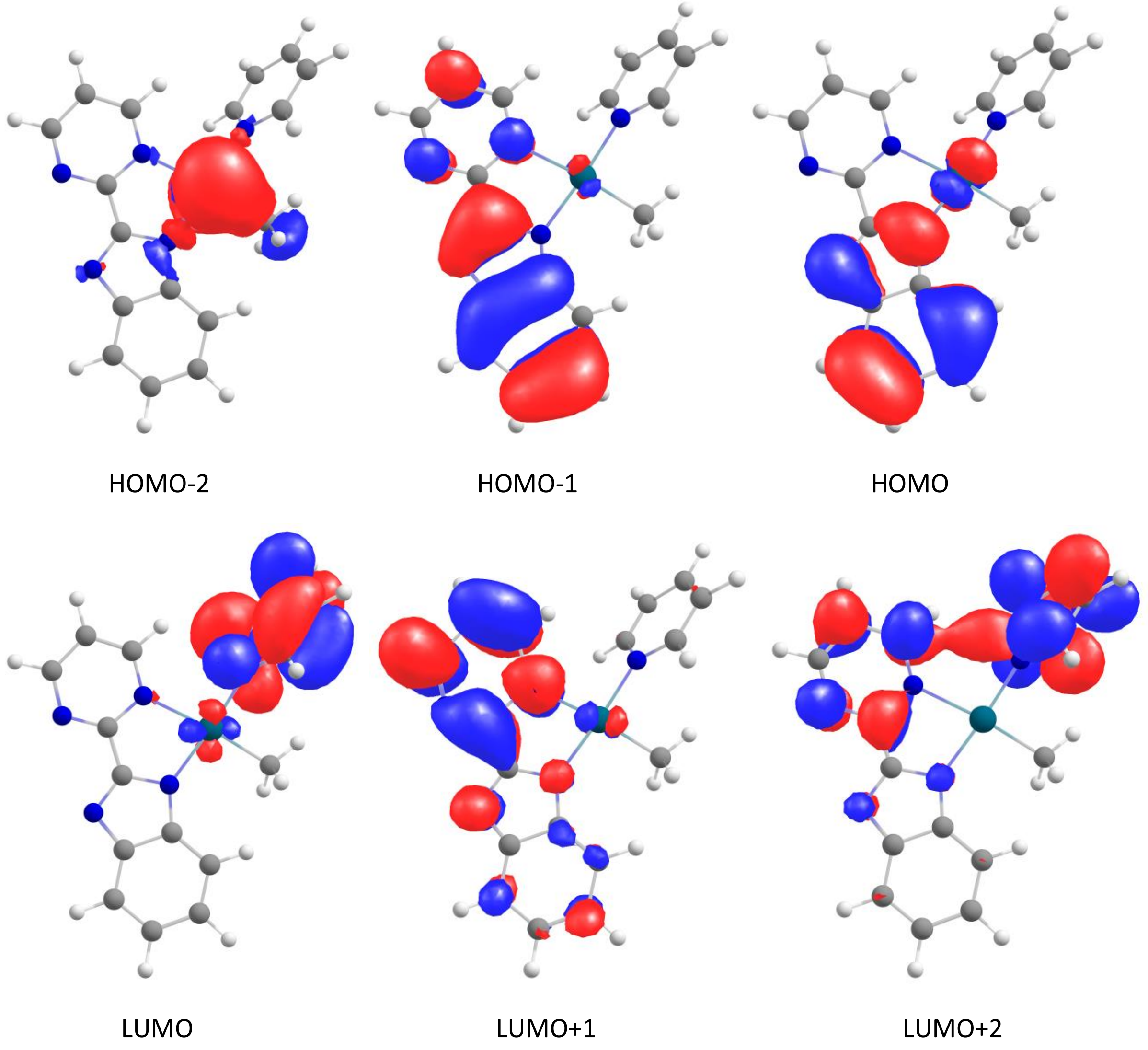

Figure S37. *Preview of main orbitals (isocontour value = 0.3) of* ***1****, following single-point energy calculation using TPSSh-D3 density functional. The shape of the main orbitals is invariant of the functional used.*

b) Compound **4**

1) DFT calculations

Table S20 *: Main distances (in Å) and angles (in °), as observed in the crystal structure and in the DFT optimized geometries using the PBE-D3 density functional.*

Distance \ source	XRD structure	PBE-D3
Sm_1-Pd	2.908(1)	2.924
Sm_2-Pd	2.908(1)	2.876
Sm_1-$N_{pyridyl}$	2.386(3)	2.428
Sm_2-$N_{pyridyl}$	2.386(3)	2.418
Sm_1-$C_{pyridyl}$	2.861(4)	2.952
Sm_2-$C_{pyridyl}$	2.861(4)	2.907
Sm_1 – $Cp^*_{1\ ctr}$	2.452	2.465
Sm_1 – $Cp^*_{2\ ctr}$	2.446	2.456
Sm_2 – $Cp^*_{1\ ctr}$	2.452	2.455
Sm_2 – $Cp^*_{2\ ctr}$	2.446	2.456
Pd-$C_{pyridyl1}$	2.081(4)	2.075
Pd-$C_{pyridyl2}$	2.081(4)	2.093
<($C_{pyridyl1}$-Pd-$C_{pyridyl2}$)	179.8(2)°	177.39°
<($Cp^*_{1\ ctr}$ -Sm_1-$Cp^*_{2\ ctr}$)	134.52°	135.09°
<($Cp^*_{1\ ctr}$ -Sm_2-$Cp^*_{2\ ctr}$)	134.52°	131.87°

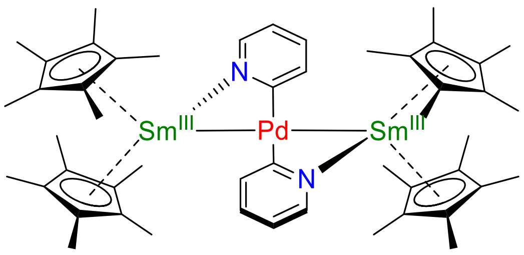

Table S21. *Preview of main alpha orbitals of* ***4*** *(isocontour value = 0.3), following single point energy calculation using several different density functionals*

Functional / Orbital	PBE-D3	PBE0-D3	TPSSh-D3	ωB97X-D3
SOMO-3	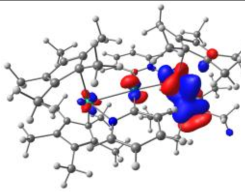	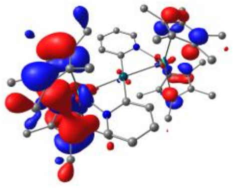	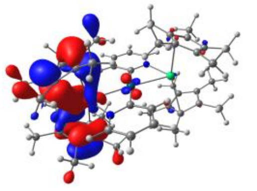	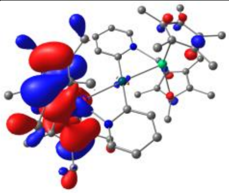
SOMO-2	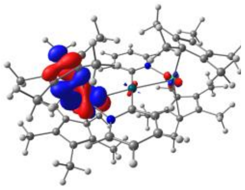	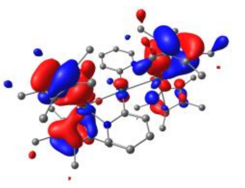	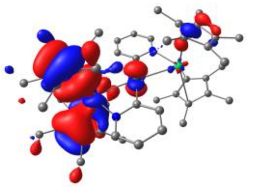	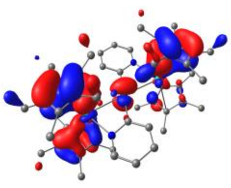
SOMO-1	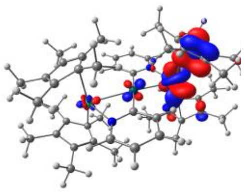	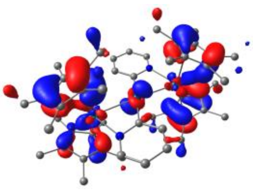	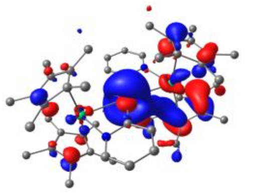	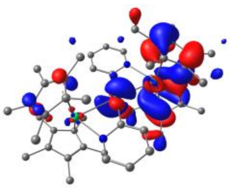
SOMO	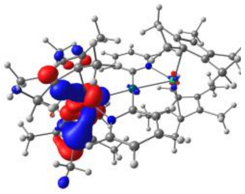	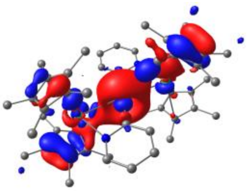	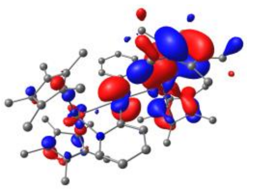	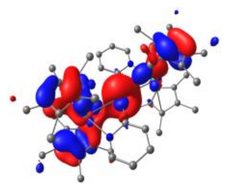
LUMO	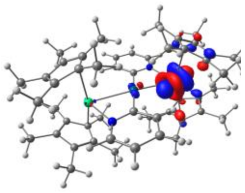	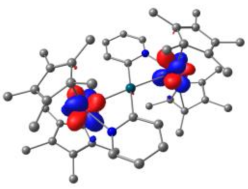	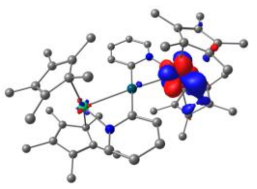	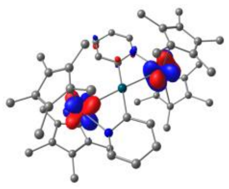
LUMO+1	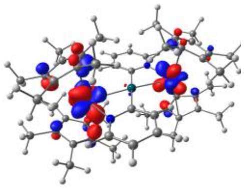	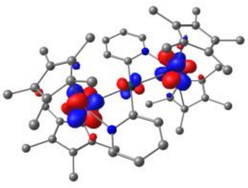	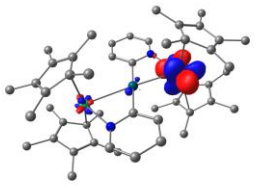	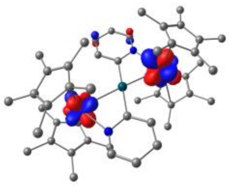
LUMO+2	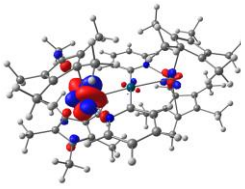	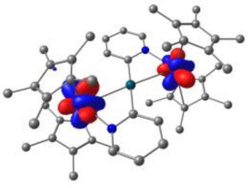	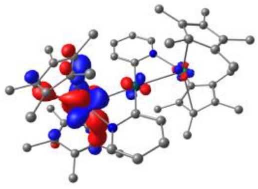	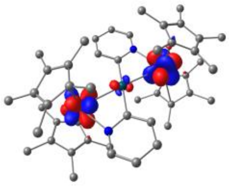

LUMO+3

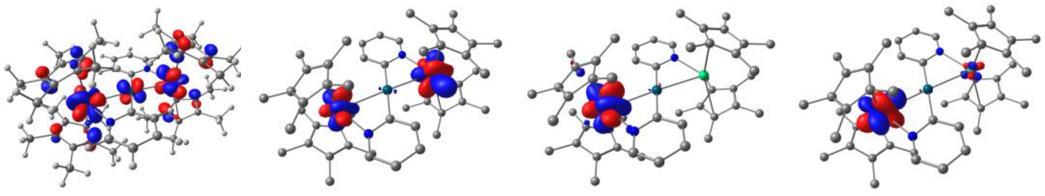

*Table S22. Preview of main beta orbitals of **4** (isocontour value = 0.3), following single point energy calculation using several different density functionals*

Functional / Orbital	PBE-D3	PBE0-D3	TPSSh-D3	ωB97X-D3
SOMO-3	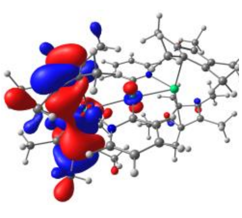	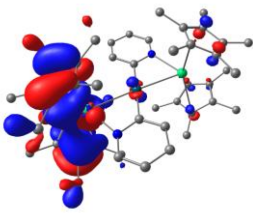	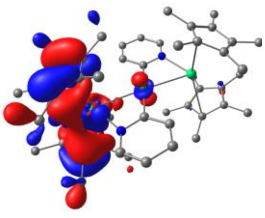	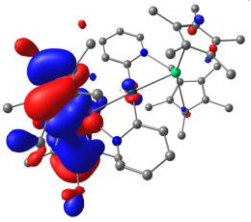
SOMO-2	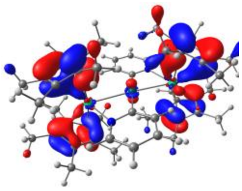	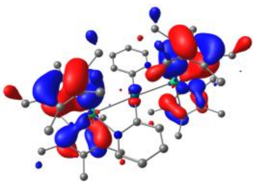	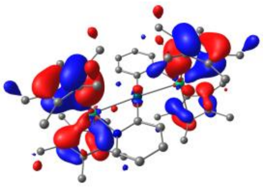	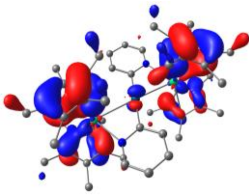
SOMO-1	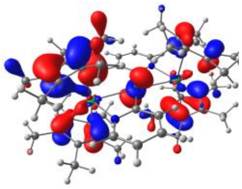	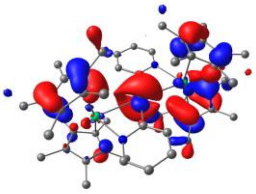	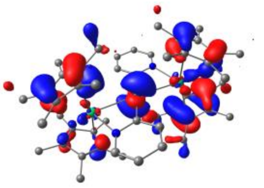	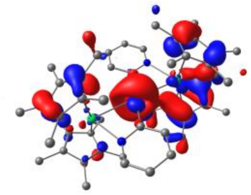
SOMO	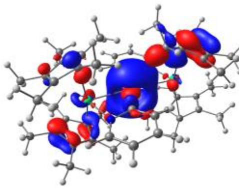	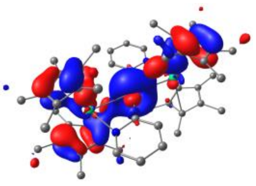	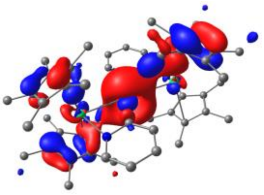	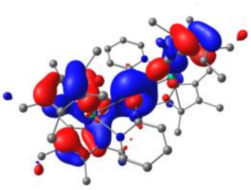
LUMO	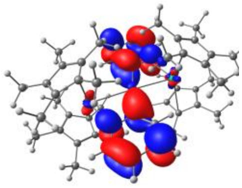	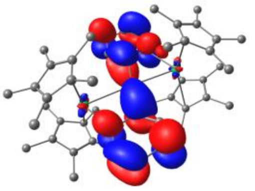	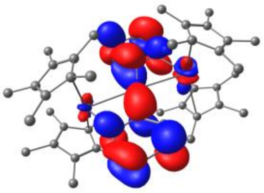	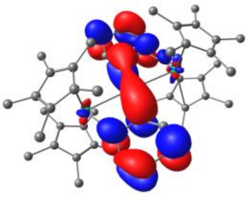
LUMO+1	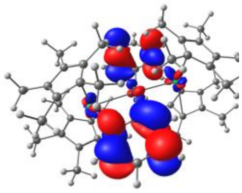	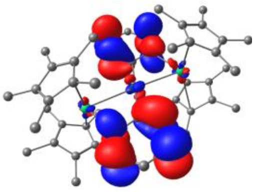	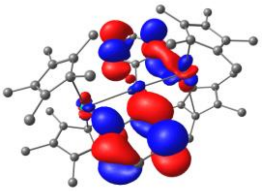	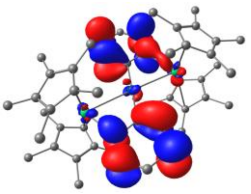
LUMO+2	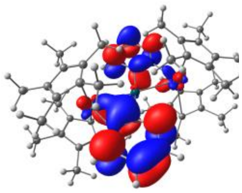	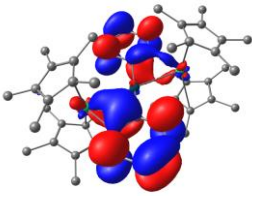	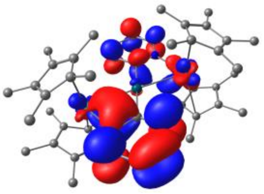	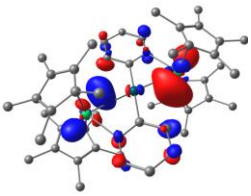

LUMO+3 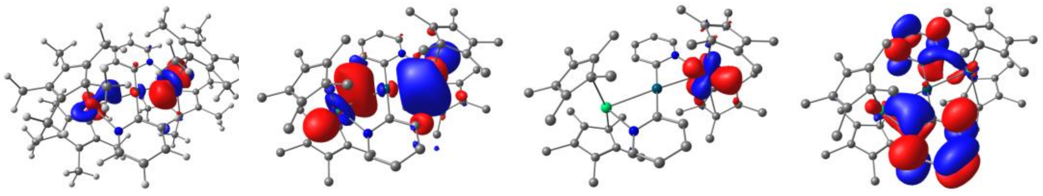

Orbital	ΔE (SOMO vs orbital, Eh)	
SOMO-18	-0.3090	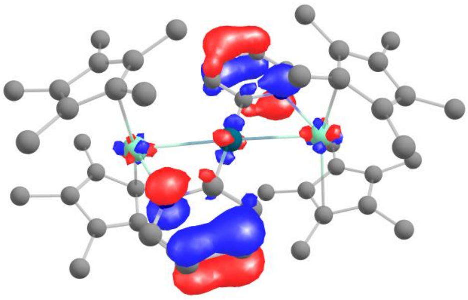
SOMO-17	-0.3074	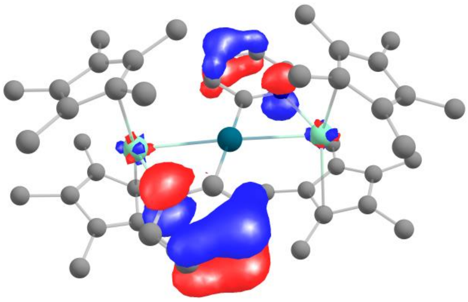
SOMO-16	-0.2994	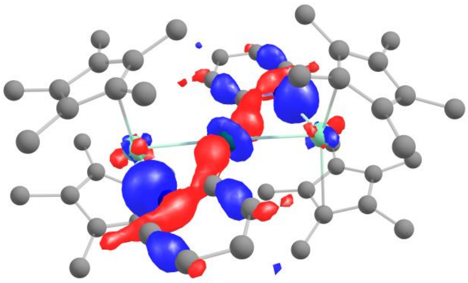
SOMO-13	-0.2676	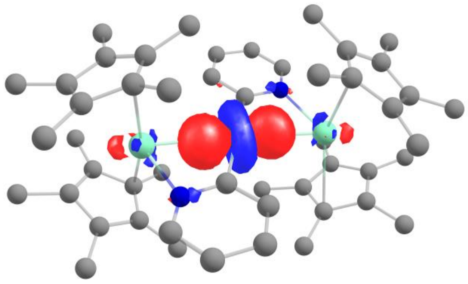
SOMO-8	-0.2147	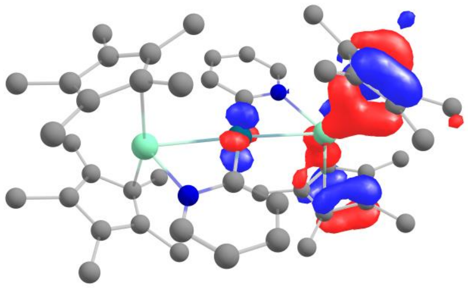

Figure S38. *Preview of miscellaneous orbitals (isocontour value = 0.5) of interest in compound* ***4****, computed at the DFT/PBE0-D3 level of theory.*

2) CASSCF calculations

CASSCF computations were performed with an initial active space of 10 electrons in 12 orbitals (10,12). This active space was progressively increased up to the (18,18) configuration, in order to take into account the Sm 4f electrons and Pd 4d electrons. Manual orbital permutations were undertaken in order to attempt to promote the Pd 4d electrons into the active space. Despite several attempts, including varying the size of the active space, these permutations were not successful.

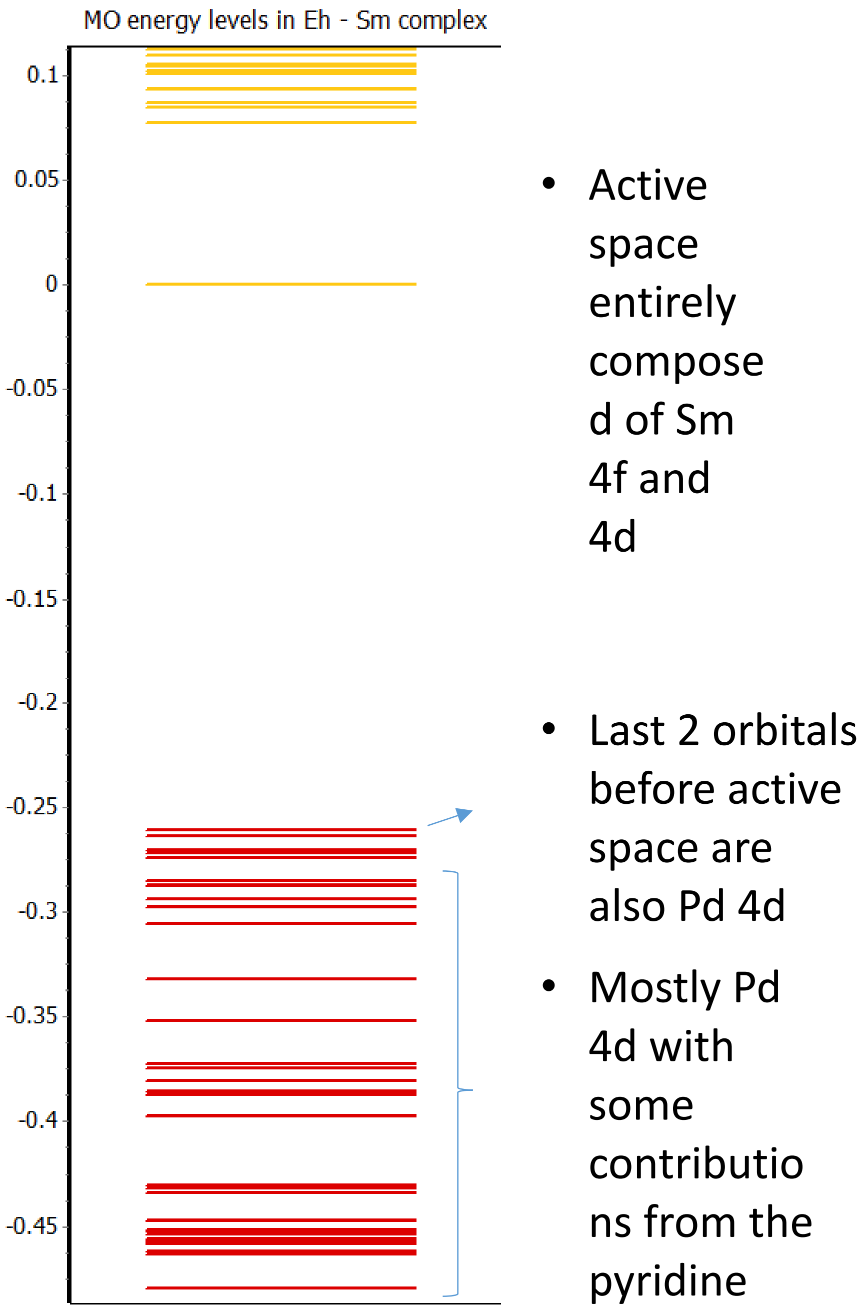

Figure S39. *Schematic representation of the relative energies of the orbitals involving contributions from the metals in compound* ***4*** *at the CASSCF level of theory.*

***Table S23**: CASSCF-derived orbitals of compound **4**.*

Orbital type	ΔE (active space vs orbital, Eh)	Orbital
Orbitals with contributions from Pd 4d	0.4798	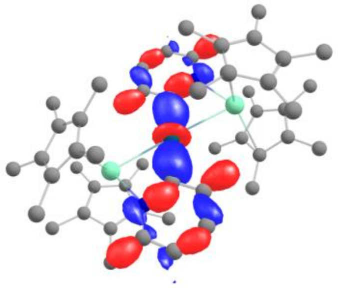
	10 consecutive comparable orbitals between 0.4308-0.3060 Eh from active space	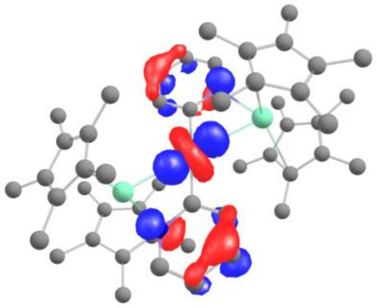
	0.2642	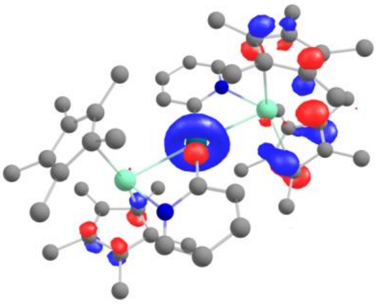
Active space	0	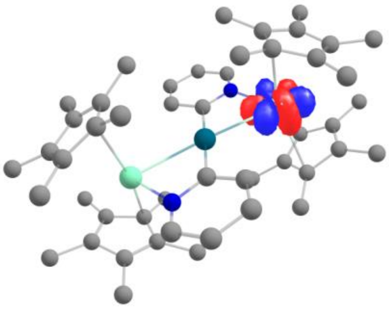
	0	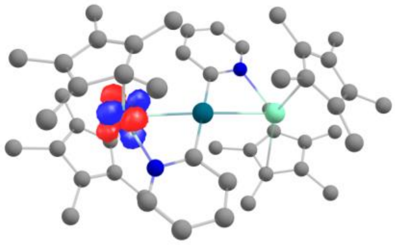

0

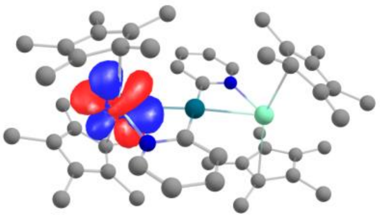

c) Compound **5**

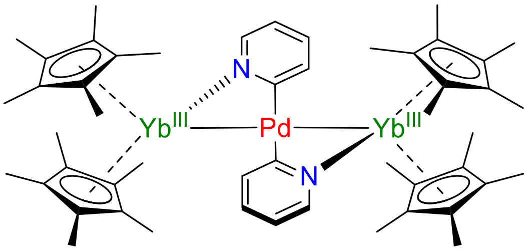

1) DFT calculations

Table S24. *Main distances (in Å) and angles (in °) in **5**, as observed in the crystal structure and in the DFT optimized geometries using the PBE-D3 density functional.*

Distance \ source	XRD structure	PBE-D3
Yb_1-Pd	2.924(1)	2.902
Yb_2-Pd	2.924(1)	2.902
Yb_1-$N_{pyridyl}$	2.275(3)	2.320
Yb_2-$N_{pyridyl}$	2.275(3)	2.320
Yb_1-$C_{pyridyl}$	2.773(4)	2.844
Yb_2-$C_{pyridyl}$	2.773(4)	2.843
Yb_1 – $Cp^*_{1\ ctr}$	2.345	2.370
Yb_1 – $Cp^*_{2\ ctr}$	2.339	2.371
Yb_2 – $Cp^*_{1\ ctr}$	2.345	2.370
Yb_2 – $Cp^*_{2\ ctr}$	2.339	2.371
Pd-$C_{pyridyl1}$	2.078(4)	2.077
Pd-$C_{pyridyl2}$	2.078(4)	2.077
<($C_{pyridyl1}$-Pd-$C_{pyridyl2}$)	178.88(18)°	176.44°
<($Cp^*_{1\ ctr}$ -Yb_1- $Cp^*_{2\ ctr}$)	134.70°	131.92°
<($Cp^*_{1\ ctr}$ -Yb_2- $Cp^*_{2\ ctr}$)	134.70°	131.92°

Table S25. *Preview of main alpha orbitals of* ***5*** *(isocontour value = 0.3), following single point energy calculation using different density functionals*

Functional / Orbital	PBE0-D3	TPSSh-D3	ωB97X-D3
SOMO-3	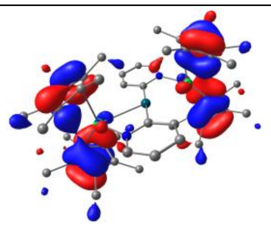	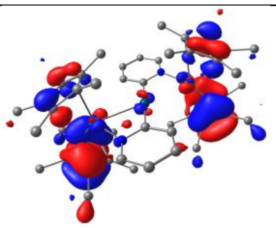	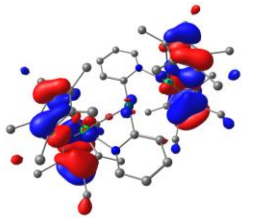
SOMO-2	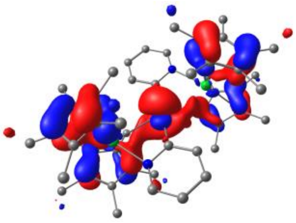	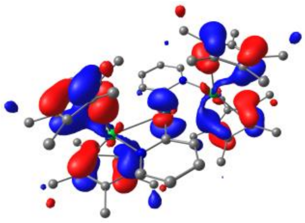	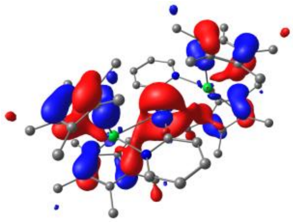
SOMO-1	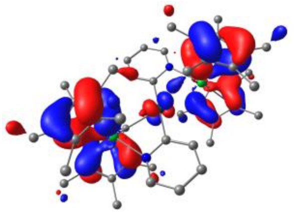	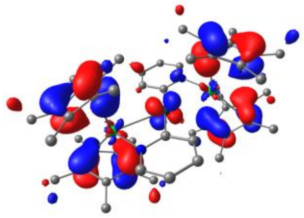	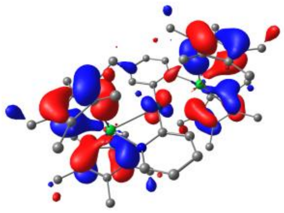
SOMO	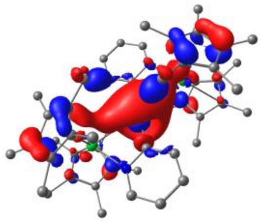	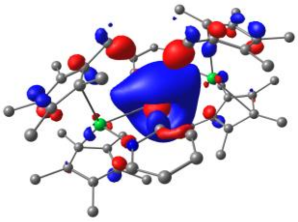	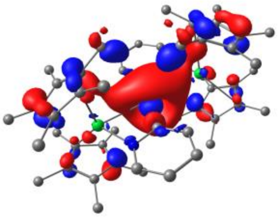
LUMO	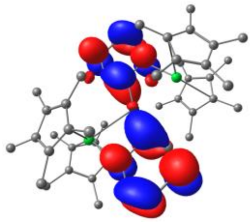	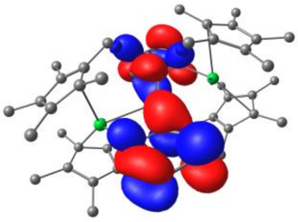	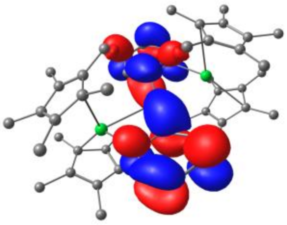
LUMO+1	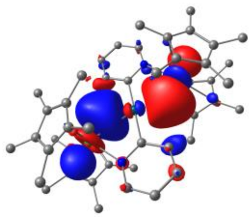	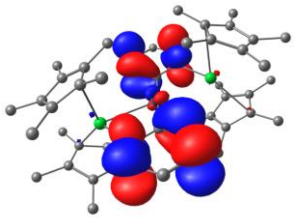	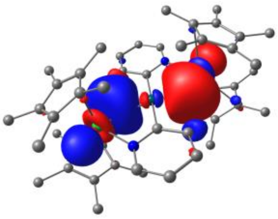

LUMO+2

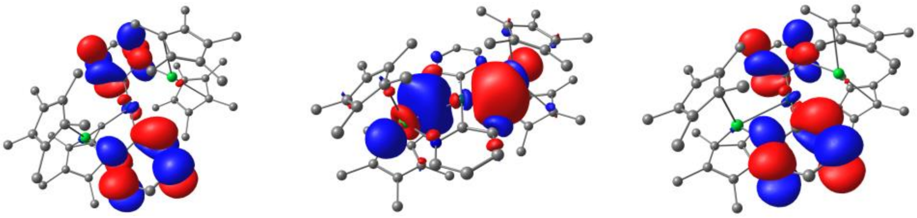

LUMO+3

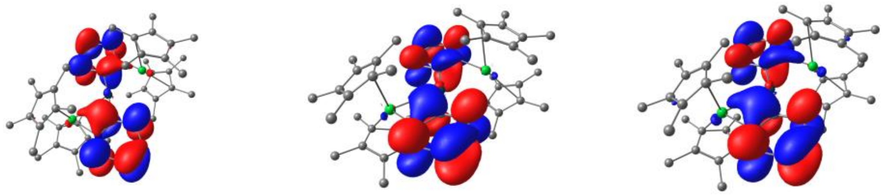

Table S26. *Preview of main beta orbitals of* ***5*** *(isocontour value = 0.3), following single point energy calculation using different density functionals*

Functional / Orbital	PBE0-D3	TPSSh-D3	ωB97X-D3
SOMO-3	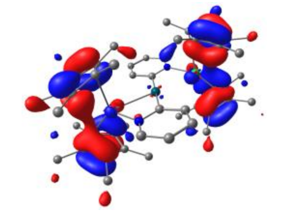	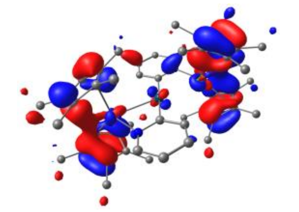	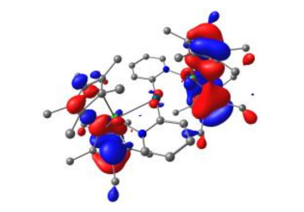
SOMO-2	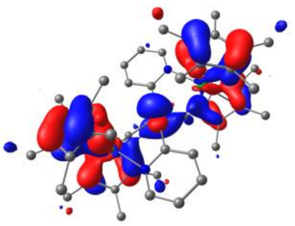	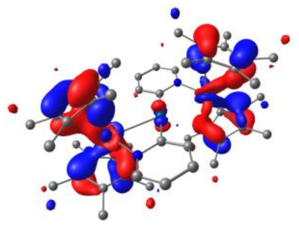	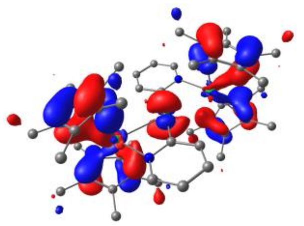
SOMO-1	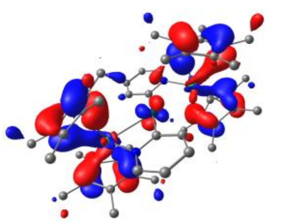	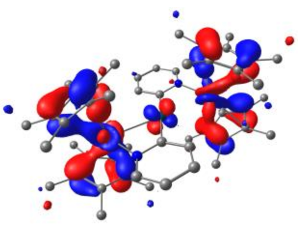	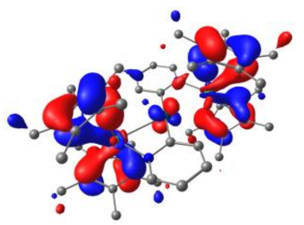
SOMO	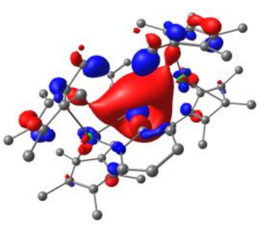	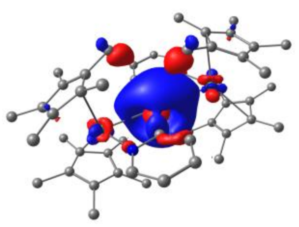	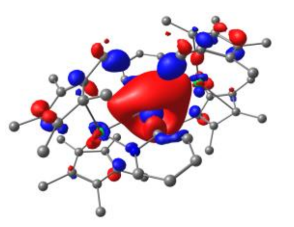
LUMO	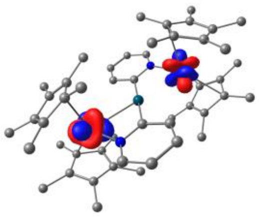	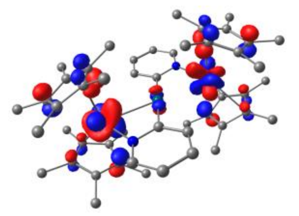	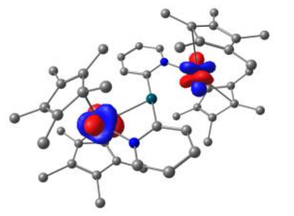
LUMO+1	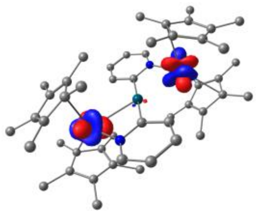	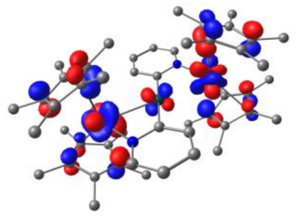	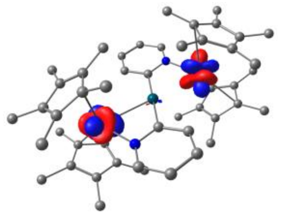
LUMO+2	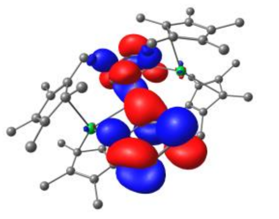	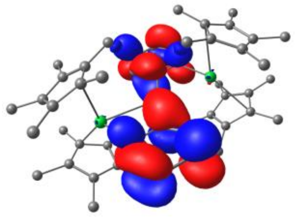	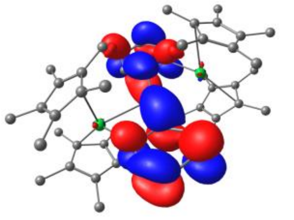

LUMO+3

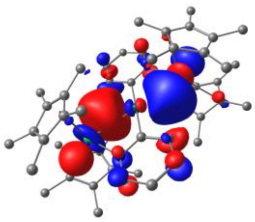

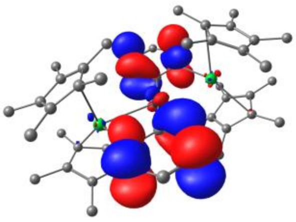

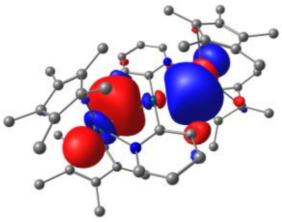

Orbital	ΔE (SOMO vs orbital, Eh)	
SOMO-64	0.2214	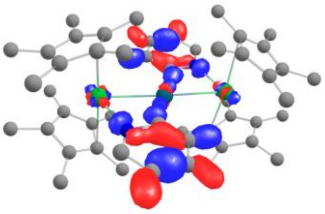
SOMO-32	0.1654	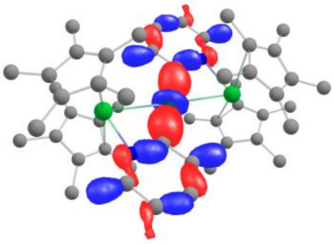
SOMO-18	0.1194	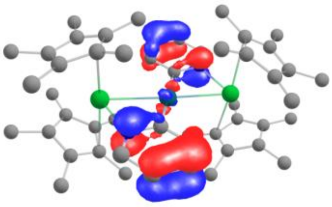
SOMO-16	0.113	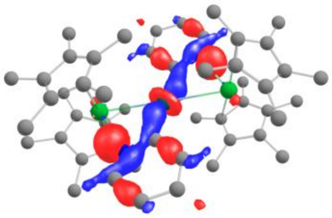
SOMO-13	0.0731	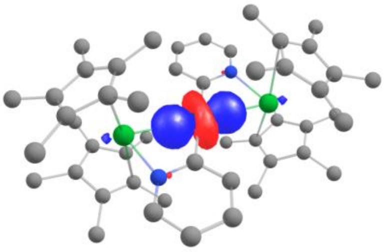

Figure S40. *Preview of miscellaneous orbitals (isocontour value = 0.5) of interest in compound **5**, computed at the DFT/PBE0-D3 level of theory.*

2) CASSCF calculations

CASSCF computations were performed with an initial active space of 6 electrons in 12 orbitals (6,12). This active space was progressively increased up to the (12,16) configuration, in order to take into account the Yb 4f electrons and Pd 4d electrons. Manual orbital permutations were undertaken in order to attempt to promote the Yb 4f electrons into the active space. Despite several attempts, including varying the size of the active space, these permutations were not successful.

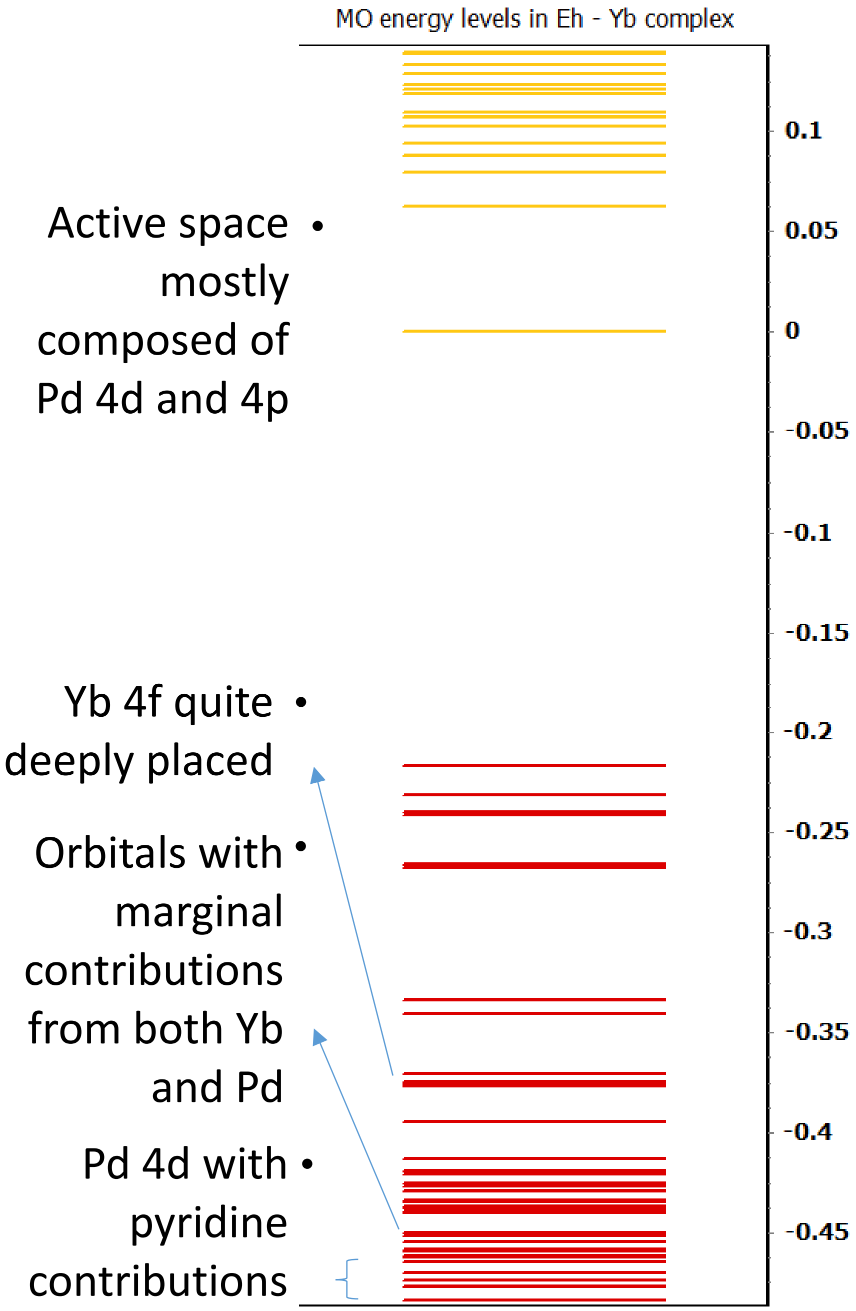

Figure S41. *Schematic representation of the relative energies of the orbitals involving contributions from the metals in compound **5** at the CASSCF level of theory.*

Table S27. CASSCF-derived orbitals of compound 5.

Orbital type	ΔE (active space vs orbital, Eh)	Orbital
Orbitals with marginal contributions from both Yb and Pd	0.4612	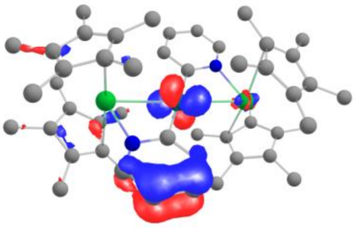
	0.4590	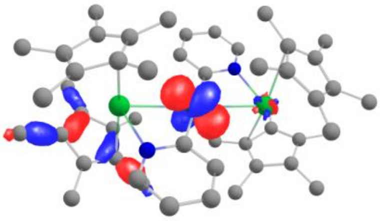
	0.4579	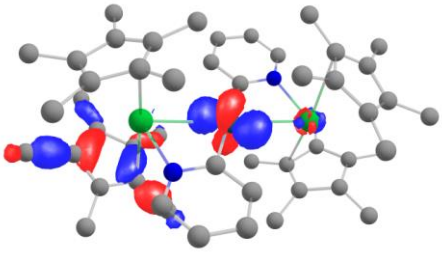
	0.4499	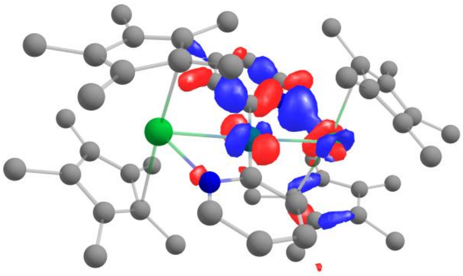
	Several such orbitals between 0.4397-0.4370	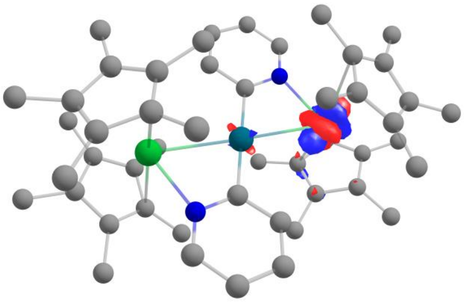

0.4368

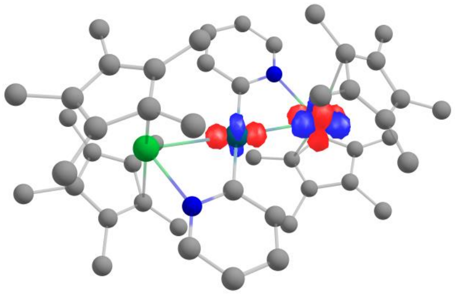

0.4341

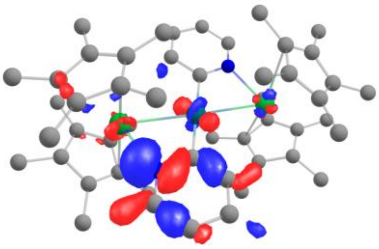

0.4252

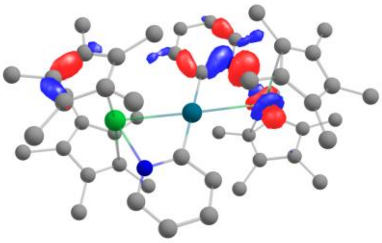

Active space

0

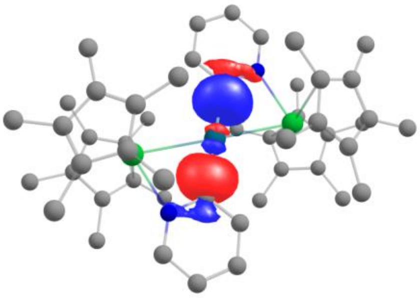

0

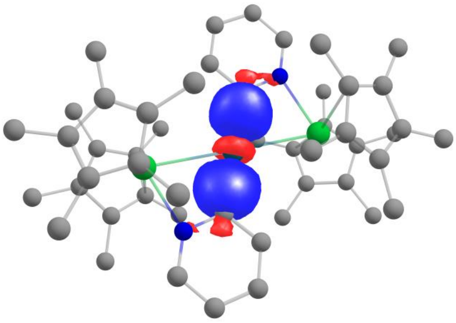

	0	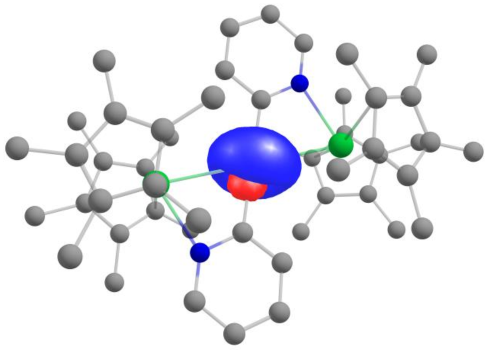
Vacant active space	0	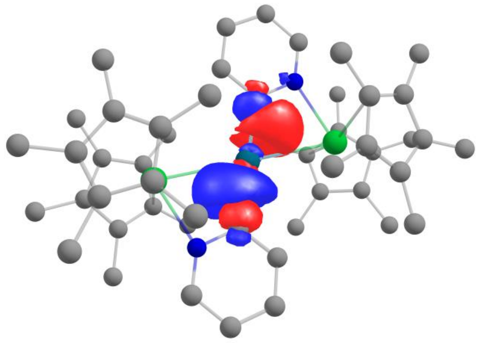

d) Compound **6**

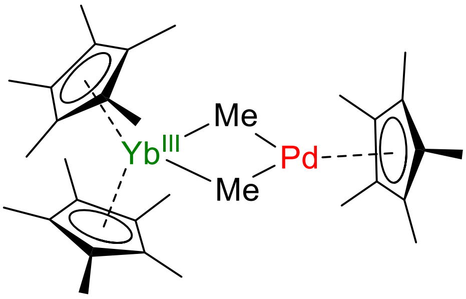

Table S28. *Summary of main distances (in Å) and angles in* ***6*** *in the crystal structure and in the DFT optimized geometry.*

Distance \ source	XRD structure	PBE-D3 doublet
Yb-Pd	2.877(1)	2.922
Pd-Me_1	2.123(2)	2.116
Pd-Me_2	2.123(2)	2.120
Yb-H_{Me1}	2.179	2.196
Yb-H_{Me2}	2.233	2.205
Yb-C_{Me1}	2.552(2)	2.602
Yb-C_{Me2}	2.559(2)	2.602
Pd – Cp*ctr	2.017	2.036
Yb – Cp*ctr (avg)	2.332	2.359

Table S29. *Preview of miscellaneous orbitals (isocontour value = 0.5) of interest in compound* ***6****, computed at the DFT/PBE0-D3 level of theory.*

Orbital	Alpha	Beta
SOMO-47	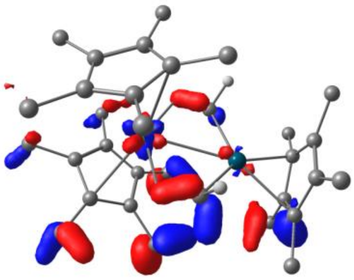	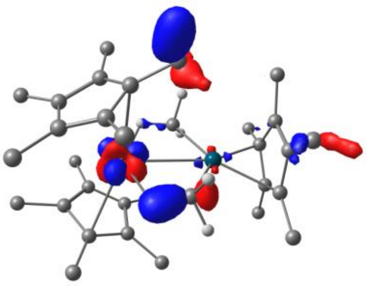
SOMO-31	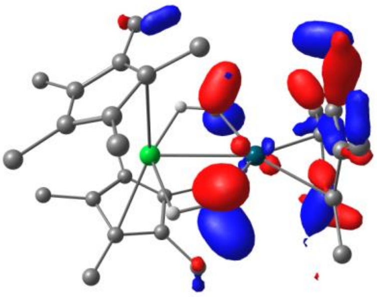	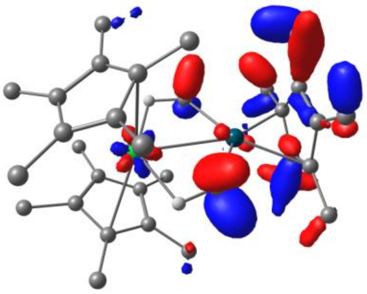
SOMO-26	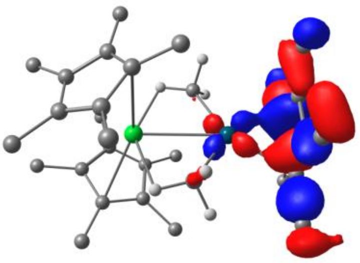	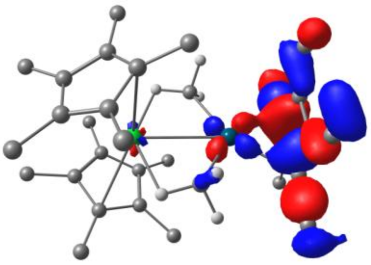
SOMO-9	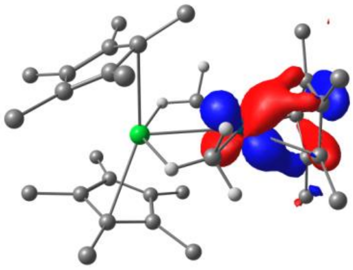	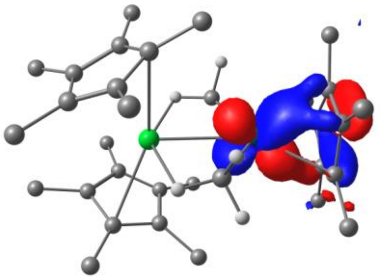
SOMO-8	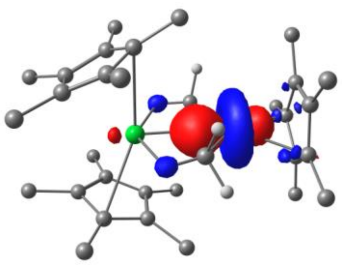	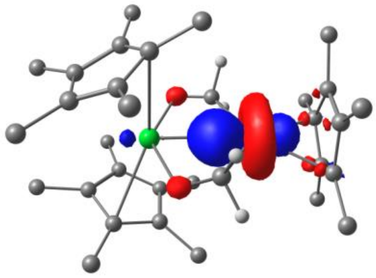

SOMO

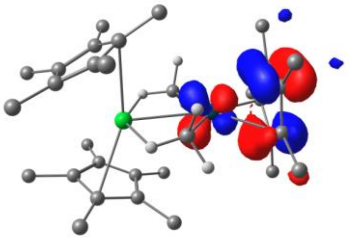

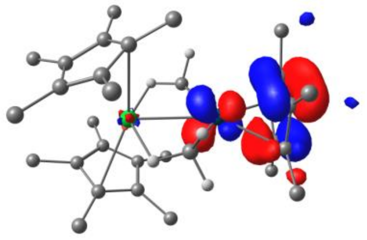

LUMO

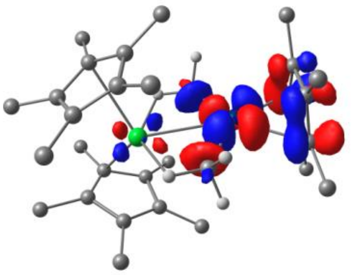

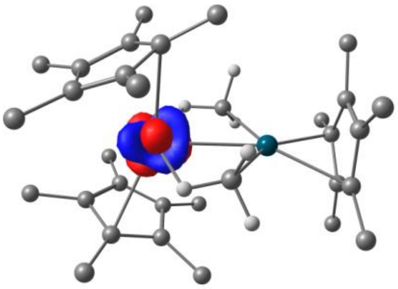

Table S30. *Preview of representative CASSCF-generated orbitals of the compound **6**.*

Orbital type	ΔE (active space vs orbital, Eh)	Orbital
Pd 4d – Cp*	0.4748	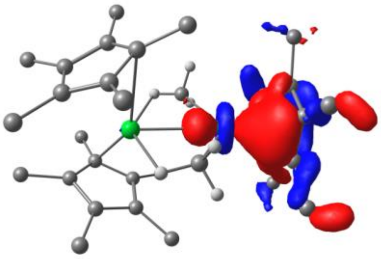
	0.4551	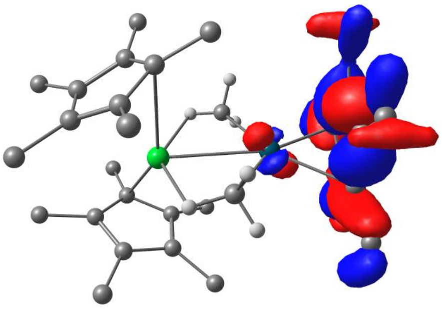
Orbitals with contributions from both Pd 4d and Yb 4f	0.4063	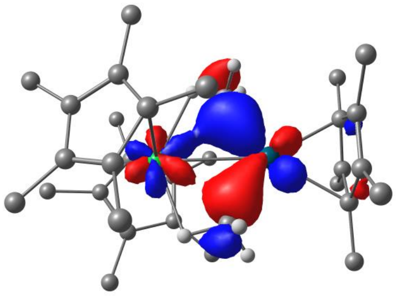
	0.3970	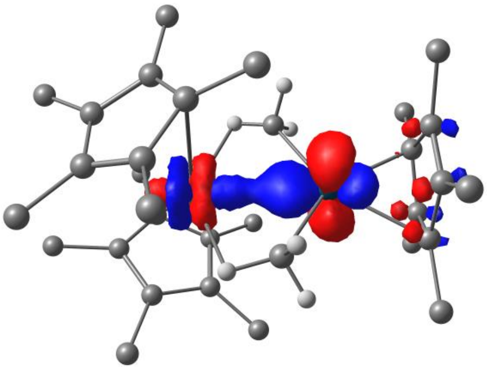

	0.3949	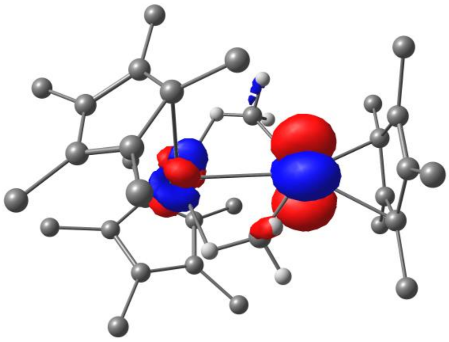
	0.3837	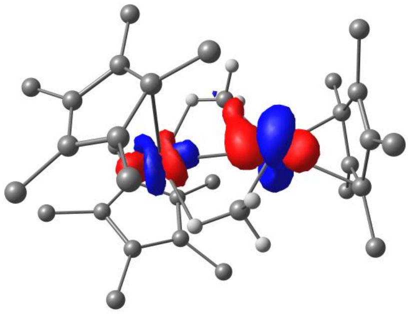
	0.3810	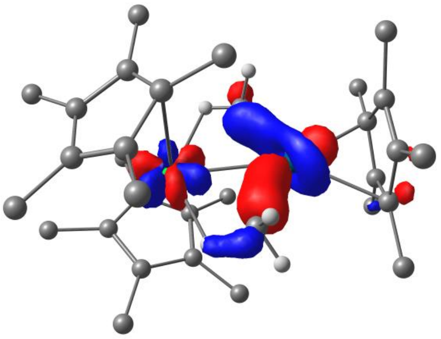
Active space	0	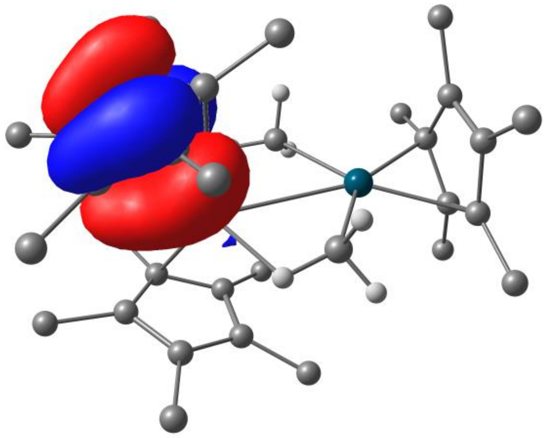

	0	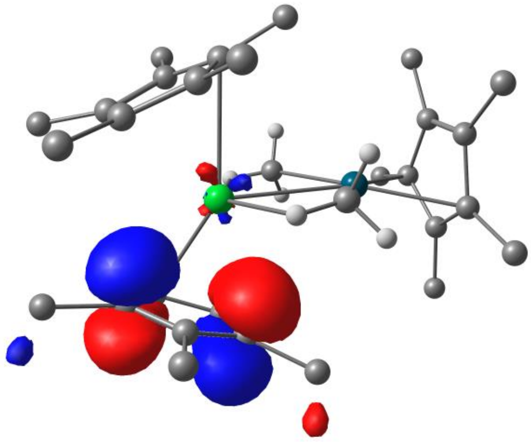

2. Topological analyses

a) **Density Overlap Regions Indicator (DORI) results**

Color code: non-covalent interaction in green, repulsion in red, covalent interaction in blue.

DORI plot for **4** : signature of a covalent interaction between Sm and N.

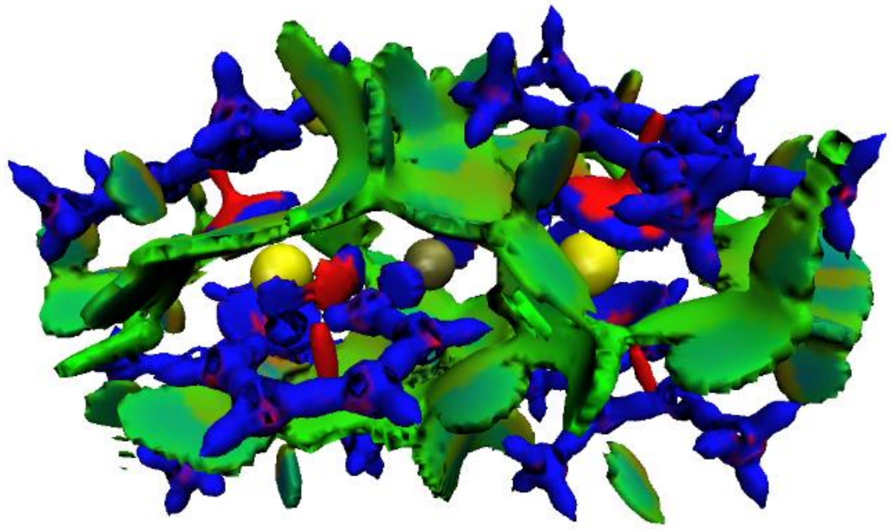

***Figure S42**: DORI plot for **4**.*

DORI plot for **5** : signature of a covalent interaction between Yb and N.

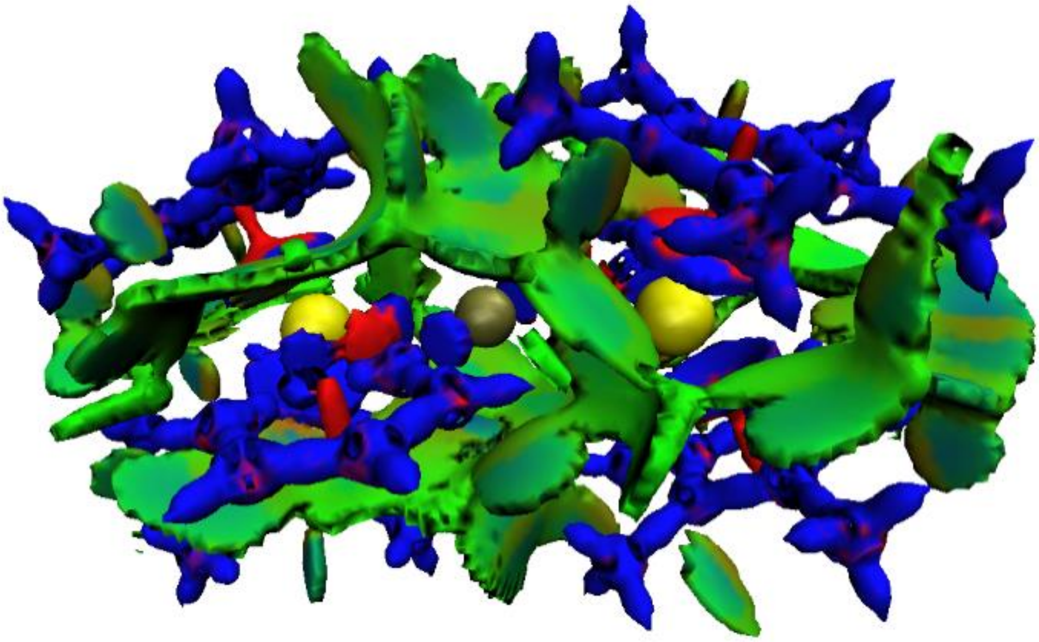

***Figure S43**: DORI plot for **5**.*

b) **QTAIM** analysis

The value of the Laplacian $\nabla^2(\rho(BCP))$ is an indication of the type of interactions, which is to be paired with the value of the total energy density H(BCP). The absolute values are related to the strength of the interactions. For example, a covalent bond is described by large negative values for both metrics, whilst positive ones correspond to ionic interactions. In our case, the values correspond to weak dative interactions.[35] Further, the delocalisation index (DI) is a measure of the quantitative sharing of electrons between the two considered atoms, but, according to Bader, is not equivalent to a bond order.[36]

Table S31: QTAIM analysis of the intermetallic adducts. Nomenclature: ρ(BCP) – electron density at the BCP; $\nabla^2(\rho(BCP))$ – Laplacian of the density; H(BCP) – energy density at the BCP; DI – delocalisation index.

	Property	ρ(BCP)	$\nabla^2(\rho(BCP))$	H(BCP)	DI
**4**	Sm-N_{pyr}	0.06	0.183	-0.007	0.346
	Sm-Pd	0.04	0.083	-0.008	0.354
	Pd-C_{pyr}	0.11	0.305	-0.032	0.847
**5**	Yb-N_{pyr}	0.06	0.217	-0.008	0.339
	Yb-Pd	0.03	0.058	-0.007	0.267
	Pd-C_{pyr}	0.11	0.313	-0.033	0.860
**6**	Yb-C_{Me}	0.04	0.129	-0.002	0.192
	Yb-Pd	0.04	0.064	-0.007	0.200

c) **ELF** analysis

Description and plot of the valence basins for the interaction in **4**

*Table S32: Main ELF basins in **4**.*

Basins	Integration	Nature	Localization Index
50	56.6	Sm	4.22
51 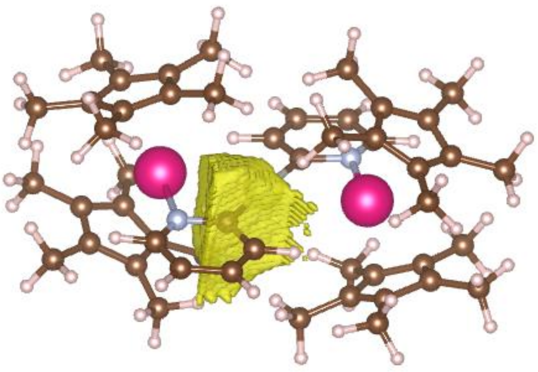	5.8	Pd-C	0.395
53 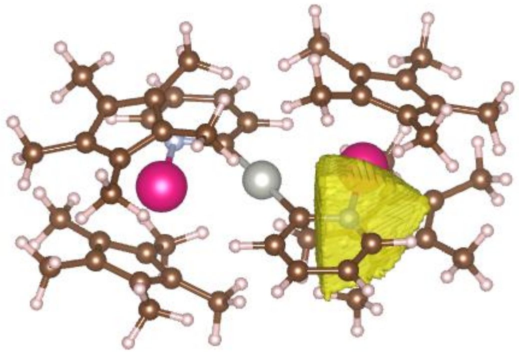	8.2	Sm-N	0.401
63	45.6	Pd-C	0.417
83	8.3	Sm-N	0.340
84	59.6	Sm	3.949
85	5.8	Pd-C	0.412

Description and plot of the valence basins for the interaction in **5**

Table S33: Main ELF basins in ***5****.*

Basins	Integration	Nature	Localization Index
78	2.81	Yb-N	4.31
88 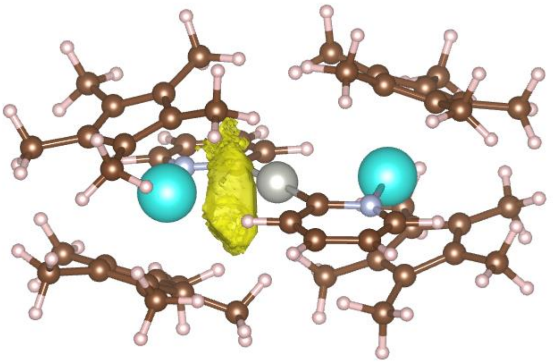	2.15	Yb-Pd	1.89
92	2.14	Yb-(N)	0.226
105 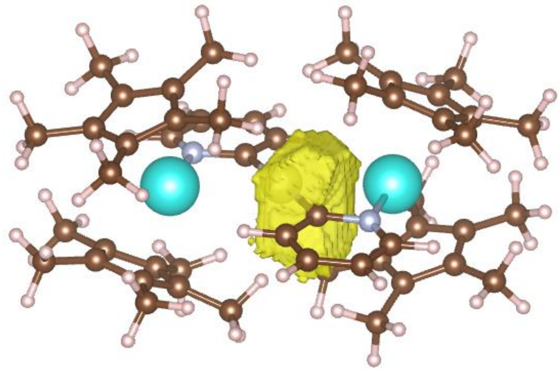	2.29	Pd-C	1.046
108	2.11	Pd-C	1.041
120	2.84	Yb-N	1.533
127 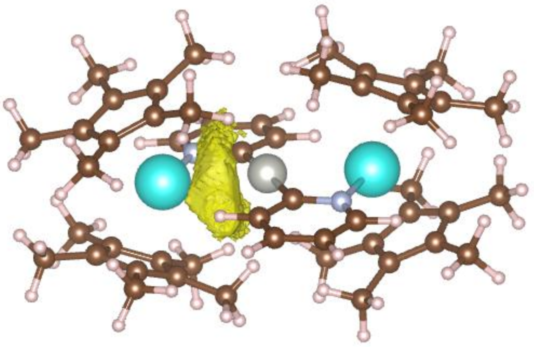	0.32	Yb-Pd	0.019
148	2.11	Yb-(N)	1.863

152 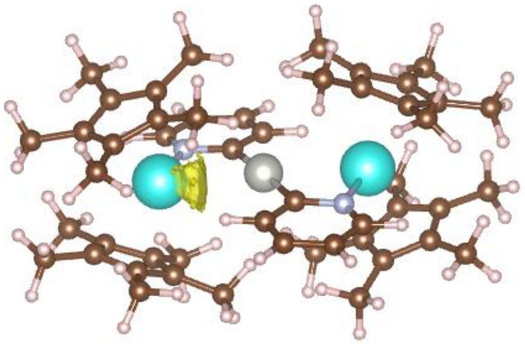	0.77	Yb-(Pd)	0.142
158	0.73	Yb-(N)	1.317

d) **Energy decomposition analysis (EDA)**

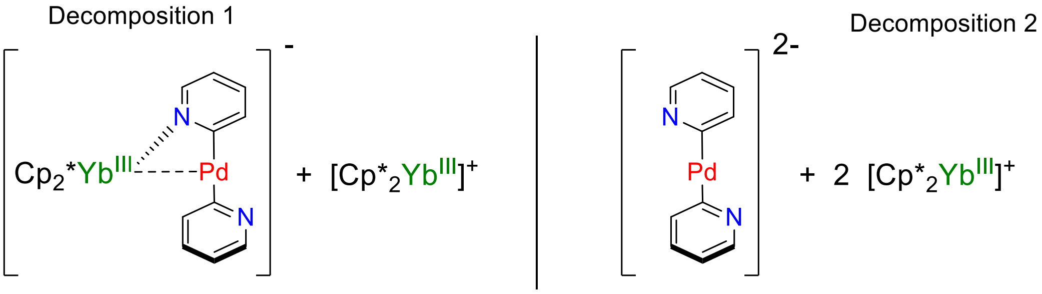

Figure S44. *EDA decomposition schemes for the trimetallic complexes (**5** is shown as an example).*

Table S34. *EDA results for complex **5**, following the two proposed decomposition schemes. The fragments are computed as neutral fragments. All energies are given in kcal/mol.*

	**Decomposition 1**		**Decomposition 2**	
	**4**	**5**	**4**	**5**
Pauli repulsion (steric)	239.88 (67)	227.55 (62.07)	466.72 (41.49)	466.59 (42.44)
Electrostatic	-172.88	-165.47	-425.23	-404.15
Orbital	-169.31	-160.41	-201.36	-203.61
Dispersion	-22.01	-22.59	-31.57	-32.69
Bonding Energy	-124.32	-120.92	-191.44	-193.87
Orbital %	*46*	*46*	*31*	*32*

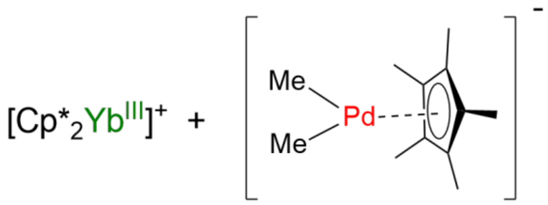

Figure S45. *EDA decomposition schemes for **6**.*

Table S35. *EDA results for complex **6**, computed as either neutral and charged fragments. Hirshfeld charges and Mayer bond order as well as "WBO" – Wiberg bond order; "FSR" – formal shortness ratio. All energies are given in kcal/mol.*

	**Neutral fragments**	**Charged fragments**
Pauli repulsion (steric)	424.94 (261.15)	102.54 (-65.62)
Electrostatic	-163.79	-168.16
Orbital	-354.5	-83.75
Dispersion	-17.94	-17.94
Bonding Energy	-111.29	-167.31
Orb%	*66*	*31*

Hirshfeld charges

*Table S36: Hirshfeld charges computed for the trimetallic adducts **4** and **5**.*

**4**	**5**
Sm 0.47 / 0.48	Yb 0.57
Pd 0.17	Pd 0.17
N -0.13	N -0.14

Mayer bond order

*Table S37: Mayer bond orders computed for the trimetallic adducts **4** and **5**.*

**4**	**5**
Sm-N 0.08	Yb-N 0.31
Pd-C 0.94	Pd-C 1.31
Sm-Pd 0.09	Yb-Pd <0.05

Bond Indices

*Table S38: Commonly used bond indices, computed for **4**-**6**.*

	**4**	**5**	**6**
WBO	0.77	0.62	0.53
FSR	0.99	1.01	0.99

3. C-H activation – calculating the transition state

In the interests of reducing the computational cost, transition state computations were initially undertaken on simplified structures, as shall be described below.

1. Computations on the compound **1**

The calculated transition state – henceforth referred to as **1-TS** – had a single imaginary frequency of 1065 cm^{-1}. This frequency corresponds to an oscillatory motion of the activated hydrogen (in the α position of the pyridine) between the two carbons. The methyl also displays bending motions of the three H atoms, in order to adopt a geometry closer to tetrahedral, characteristic of free methane.

a) Transition state calculations

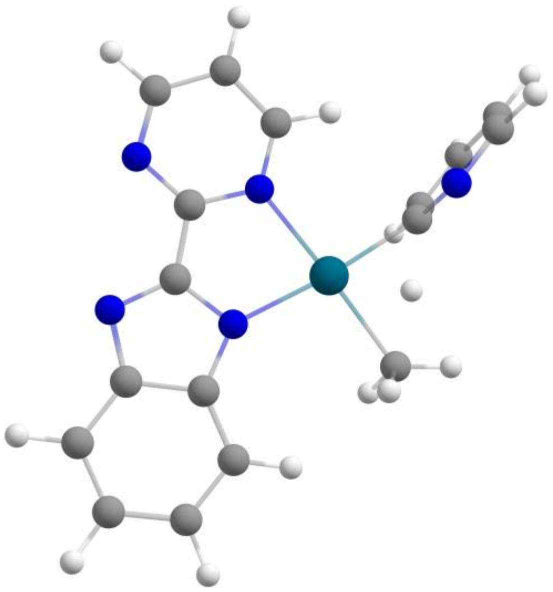

***Figure S46**: Structure of **1-TS**.*

Both the difference in enthalpy (ΔH) and in the Gibbs free energy (ΔG) are computed. These show that the **1-TS** is higher in energy by approximately 29.5-36 kcal/mol (depending on the density functional).

b) Second possible transition state

Multiple starting geometries were tested to probe whether there are other TS available on the potential energy surface. Across these trials, only one configuration led to a different structure, shown in **Figure S47**, where the pyridyl is fully coordinated to the C in the α position and the N acts as a proton shuttle. The methyl is rearranged in an intermediate configuration between that of the GS and that of free methane. Frequency calculations resulted in a single imaginary frequency (1488 cm^{-1}), which is consistent with a second possible TS, **1-TS2**.

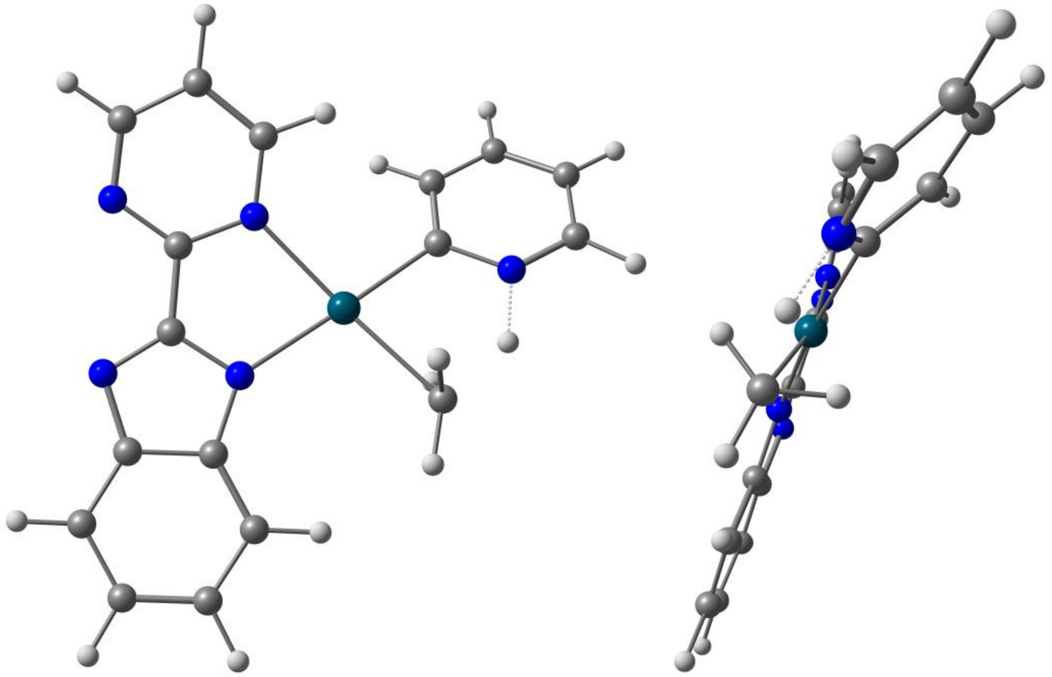

Figure S47 *: Second possible TS, front and side view.*

The steric repulsion that is inherent to the second TS configuration exerts a cost from an energetic standpoint, as the computations show it to be approximately 12-20 kcal/mol higher in energy than **1-TS**, depending on the density functional that was used. Overall, it is around 45-50 kcal/mol higher in energy than the GS. This energy barrier is very large and unfeasible for a transformation in ambient conditions. Because of this difference, **1-TS2** is not considered in further computations.

c) Computing the departure of the methyl

The next step, consistent with the expected reorganisation leading to the structures of the trimetallic edifices, would be the departure of the methyl moiety as methane. To this effect, they were removed and the structure was reoptimized.

The reduction of the number of substituents in the coordination sphere of the Pd metal centre results in an η^2-coordination mode, where the pyridyl is coordinated by both the N atom and the activated vicinal C atom. This configuration shall henceforth be referred to as **1-η²**.

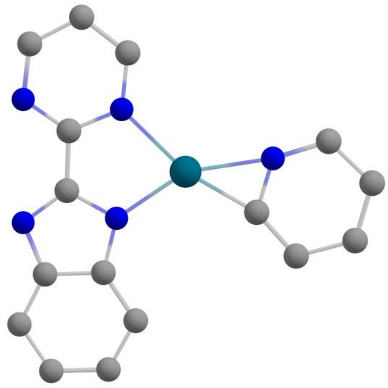

Figure S48: *Structure of **1-η²**.*

d) Comparison between the two possible transition states

Table S39. *Main distances (in Å) in the crystal structure, as well as in the two possible TS. "pym" and "imid" designate the pyrimidine and the 2-(benzimidazol-2-yl) moieties on the ligand, respectively. The computations were performed at the DFT/PBE-D3 level of theory.*

Distance \ source	XRD structure	**1-TS**	**1-TS2**
Pd-C_{Me}	2.022(3)	2.148	2.224
Pd-N_{py}	2.040(2)		
Pd-C_{py}		2.098	2.043
Pd-H_{Me}		1.650	1.975
N-H_{Me}			1.282
Pd-N_{pym}	2.176(2)	2.115	2.137
Pd-N_{imid}	2.034(2)	2.060	2.070

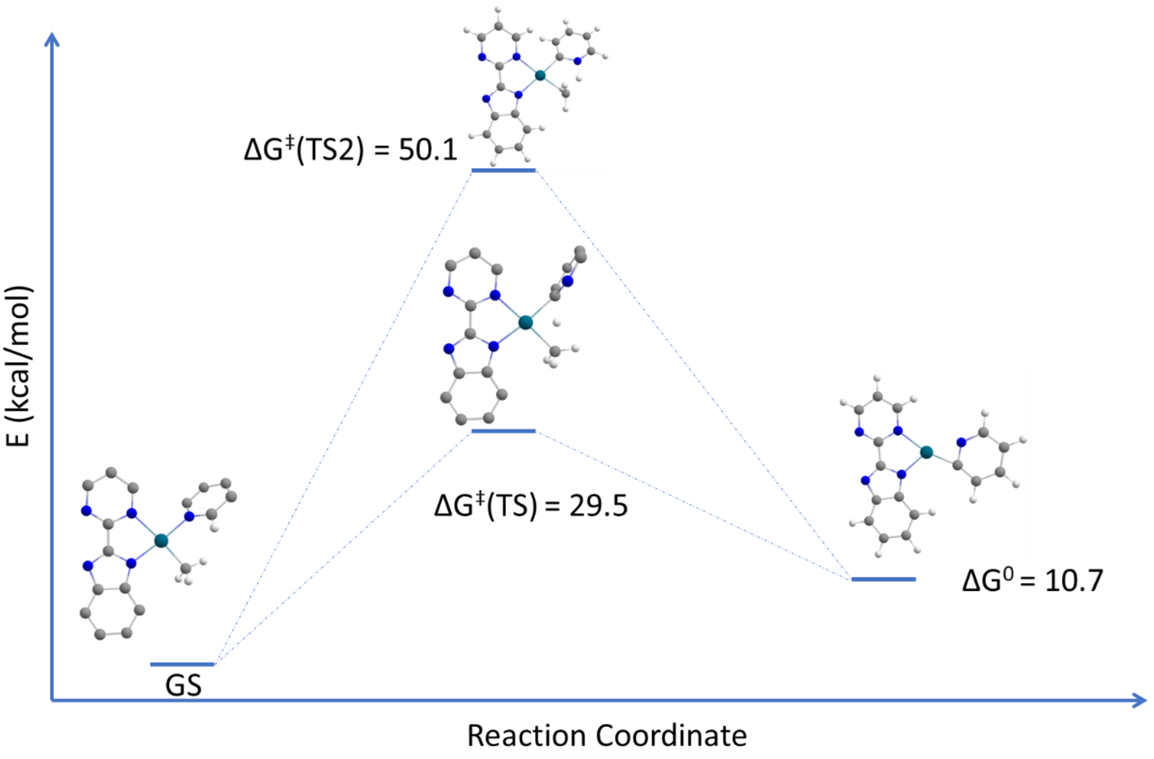

***Figure S49**: Schematic representation of the two computed TS with respect to the energy of the transformation.*

The energy profile is consistent with the fact that the C-H activation does not occur on 1 under the attempted experimental conditions (UV light and/or temperatures above 80 °C). Closer approximations to the experimental system were sought out.

2. Computations with addition of lanthanides

a) Modelling an interaction between Cp^*_2Ln and compound **1**

Computations were performed on putative heterobimetallic adducts between the Cp^*_2Sm precursor and compound **1** with a spin state S = 5/2, which will henceforth be designated as **1-Sm** and the analogous Yb (with a spin state of S = 1) adduct, **1-Yb**. The geometries were optimized at the DFT/PBE-D3 level of theory.

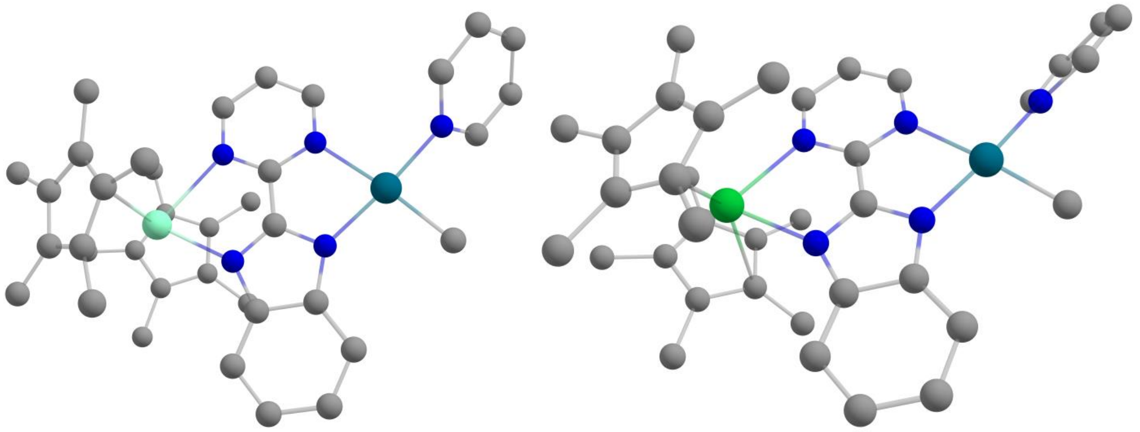

Figure S50. *Optimized geometry of **1-Sm** (left) and **1-Yb** (right).*

Table S40. *Main distances (in Å) and angles (in °) in the XRD structure of **1** and in the optimized geometry of **1-Sm**, **1-Yb** and **1-Eu.** "pym" and "imid" designate the pyrimidine and the 2-(benzimidazol-2-yl) moieties on the ligand, respectively.*

	**Av. Values**		
**Atoms**	**1**	**1-Sm**	**1-Yb**
Pd-Me	2.022(3)	2.033	2.034
Pd-N_{pyr}	2.040(2)	2.032	2.031
Pd-N_{pym}	2.176(2)	2.177	2.176
Pd-N_{imid}	2.034(2)	2.035	2.032
C_{pym}-C_{imid}	1.462(4)	1.444	1.419
Ligand plane^pyr	60.68	59.50	58.74
Ln-N_{pym}		2.521	2.422
Ln-N_{imid}		2.500	2.326
Ln – Cp^*_{ctr} (avg)		2.458	2.349

Table S41. *Preview of main alpha orbitals (isocontour value = 0.3) of **1-Sm**, following single point energy calculation using different density functionals*

Functional / Orbital	PBE0-D3	TPSSh-D3	ωB97X-D3
SOMO-1	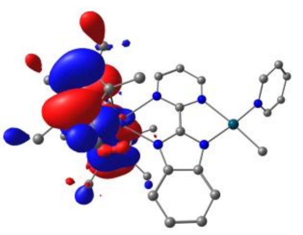	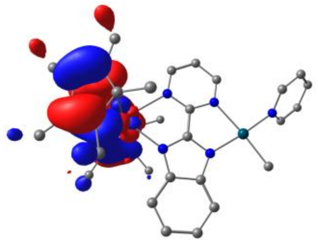	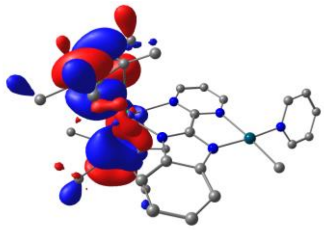
SOMO	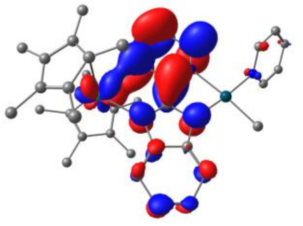	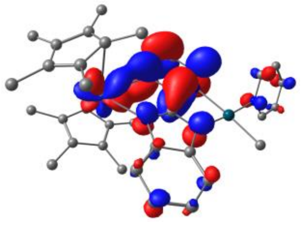	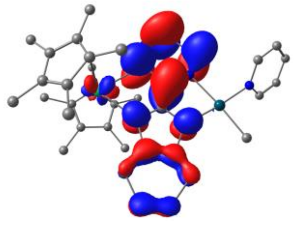
LUMO	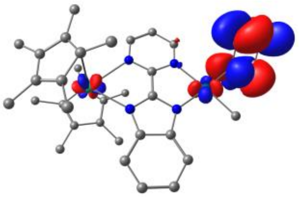	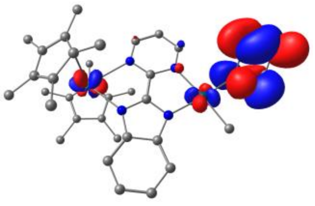	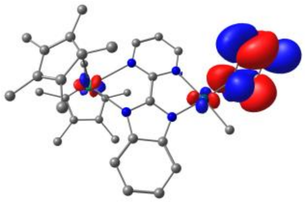
LUMO+1	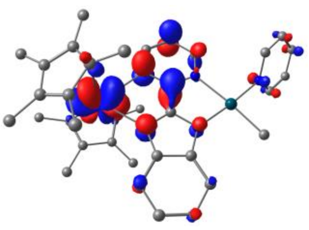	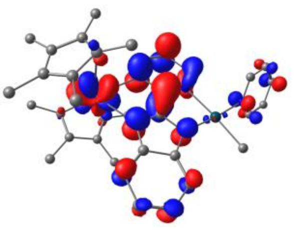	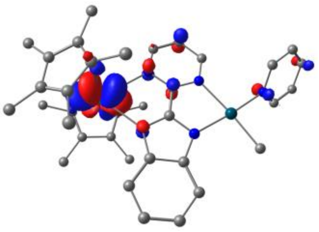

__Table S42__. Preview of main beta orbitals (isocontour value = 0.3) of __1-Sm__, following Single Point calculation using multiple density functionals.

Functional / Orbital	PBE0-D3	TPSSh-D3	ωB97X-D3
SOMO-1	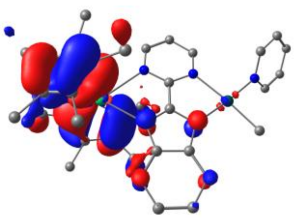	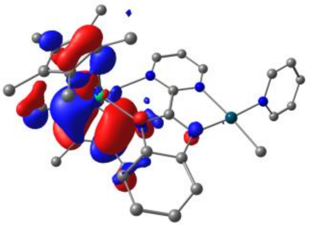	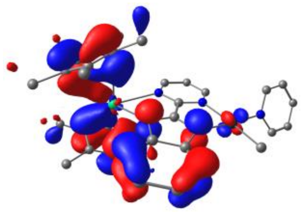
SOMO	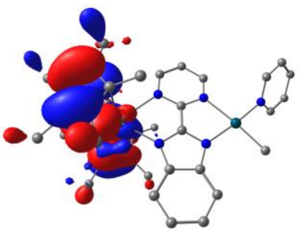	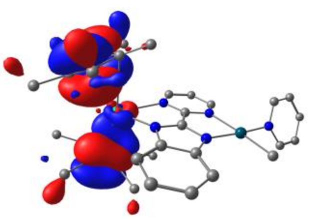	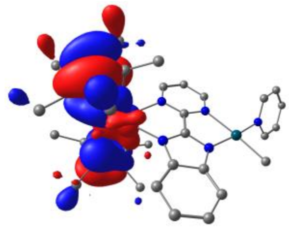
LUMO	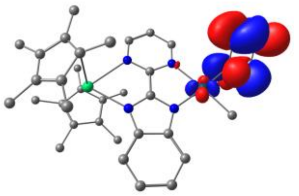	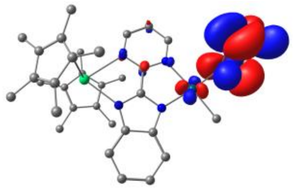	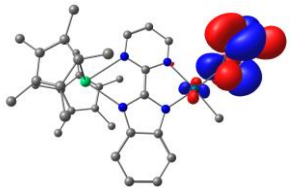
LUMO+1	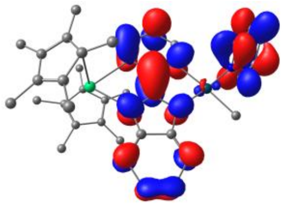	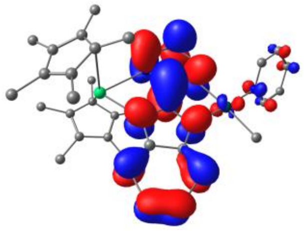	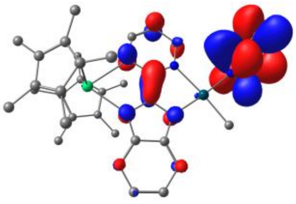

Table S43. *Preview of main alpha orbitals (isocontour value = 0.5) of* ***1-Yb****, following single point energy calculation using different density functionals.*

Functional / Orbital	PBE0-D3	TPSSh-D3	ωB97X-D3
SOMO-1	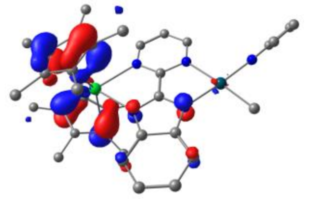	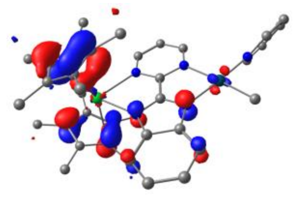	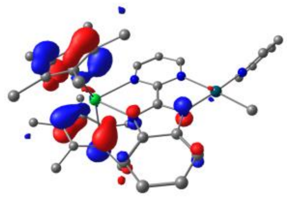
SOMO	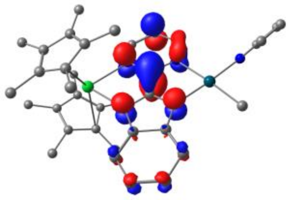	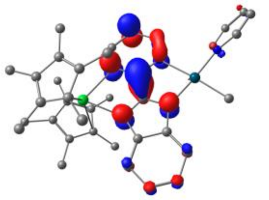	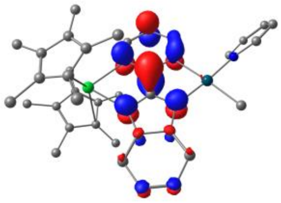
LUMO	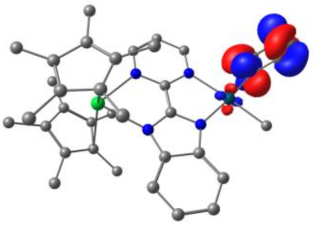	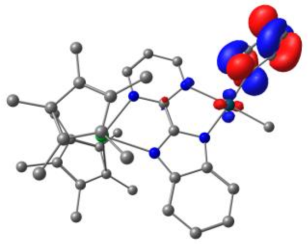	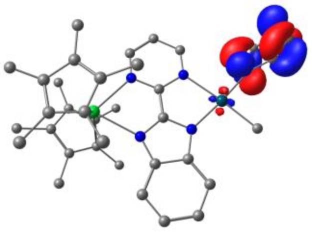
LUMO+1	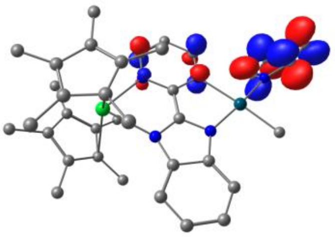	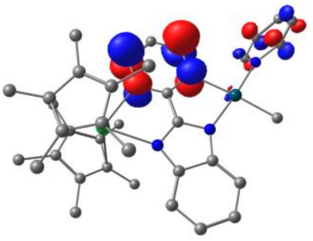	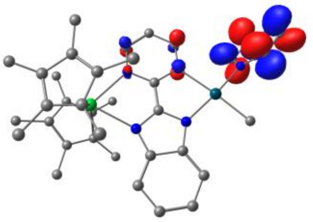

Table S44. *Preview of main beta orbitals (isocontour value = 0.5) of **1-Yb**, following single point energy calculation using different density functionals.*

Functional / Orbital	PBE0-D3	TPSSh-D3	ωB97X-D3
SOMO-1	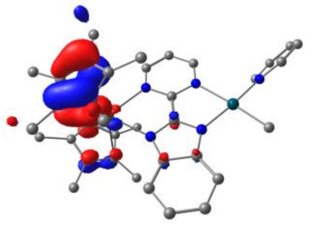	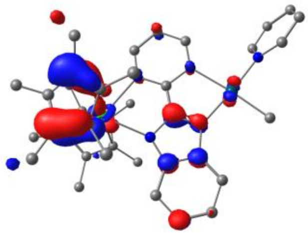	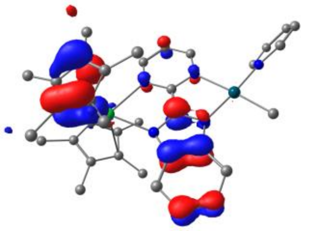
SOMO	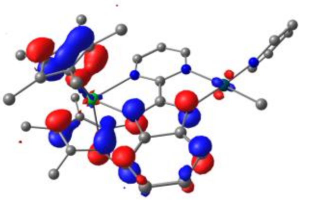	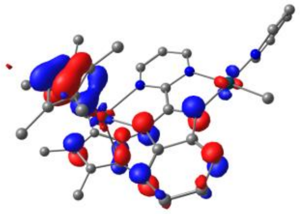	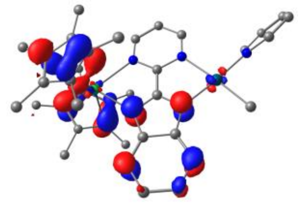
LUMO	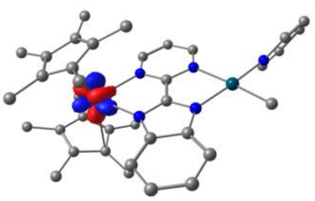	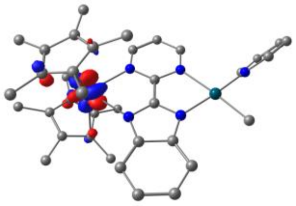	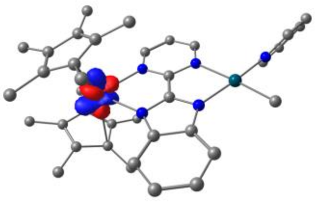
LUMO+1	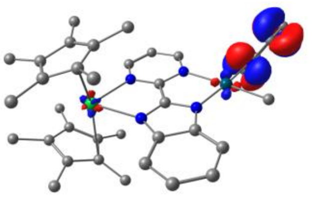	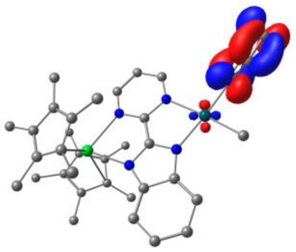	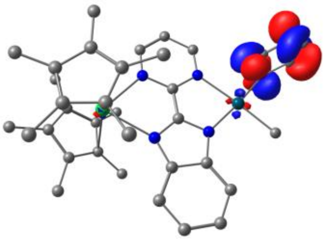

3. Computations with the explicit addition of another lanthanide

a) Modelling the addition of a further lanthanide

One further molecule of Cp*$_2$Ln was added to **1-Ln** by nestling it in the cavity formed by C$_{Me}$-Pd-py so that steric hindrance is minimized. Geometry optimizations were then performed to optimize the resulting structures. These complexes shall henceforth be referred to as **8** (Yb) and **14** (Sm). In both cases, the Cp*$_2$Ln fragment remained tilted below the plane formed by the ligand.

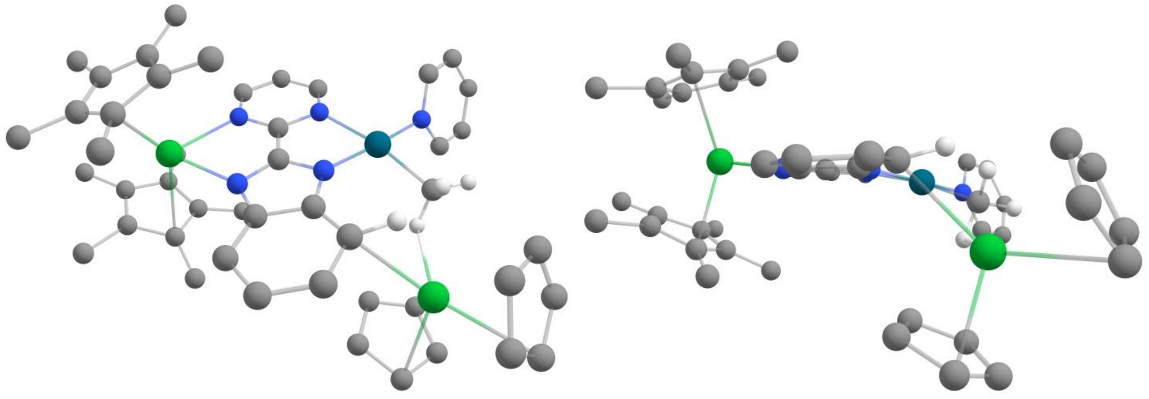

Figure S51. *Optimized geometry of **8**.*

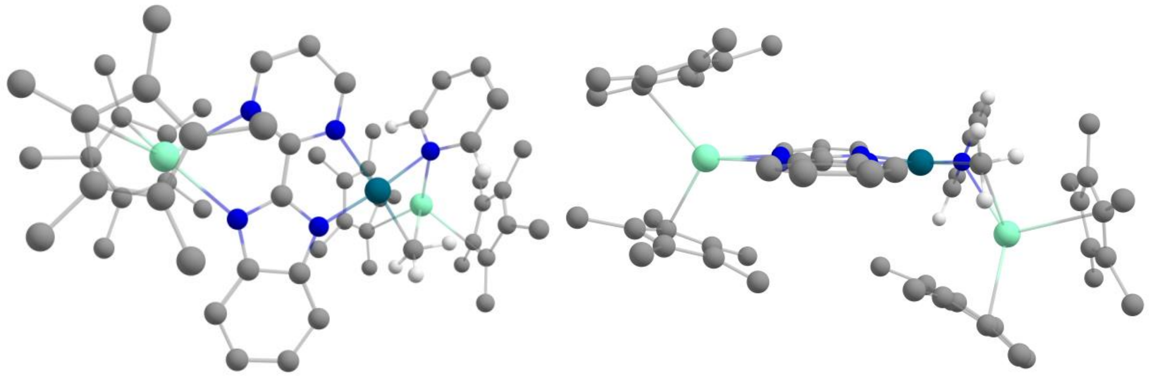

Figure S52. *Optimized geometry of **14**.*

Table S45. *Comparison of the main distances (in Å) and angles (in °) in* ***1*** *and in all the computed heterobimetallic species. "pym" and "imid" designate the pyrimidine and the 2-(benzimidazol-2-yl) moieties on the ligand, respectively.*

	**Av. Values**					
**Atoms**	**1**	**TS**	**1-Sm**	**1-Yb**	**14**	**8**
Pd-Me	2.022(3)	2.149	2.033	2.034	2.053	2.036
Pd-N_{py} (or C_{py})	2.040(2)	2.105	2.032	2.031	2.083	2.037
Pd-N_{pym}	2.176(2)	2.127	2.177	2.176	2.164	2.154
Pd-N_{imid}	2.034(2)	2.065	2.034	2.032	2.056	2.027
C_{pym}-C_{imid}	1.462(4)	1.445	1.444	1.419	1.434	1.418
Ligand plane^py	60.68°	89.25°	59.50°	58.74°	70.89°	69.19°
Ln-N_{pym}			2.521	2.422	2.557	2.450
Ln-N_{imid}			2.500	2.326	2.482	2.398
Ln_1 – Cp^*_{ctr} (avg)			2.458	2.349	2.448	2.345
Ln_2 – Cp^*_{ctr} (avg)					2.486	2.350
Pd-H_α		1.653				
Ln_2-ligand plane					~55°	~49°
Ln_2-H_α					2.816	2.672
Ln_2-H_{1Me}					2.531	2.369
Ln_2-H_{1Me}					2.680	2.692
Ln_2-C_{Me}					2.796	2.936
(Pyr plane)^(Pd-N bond)					148.17°	176.3°

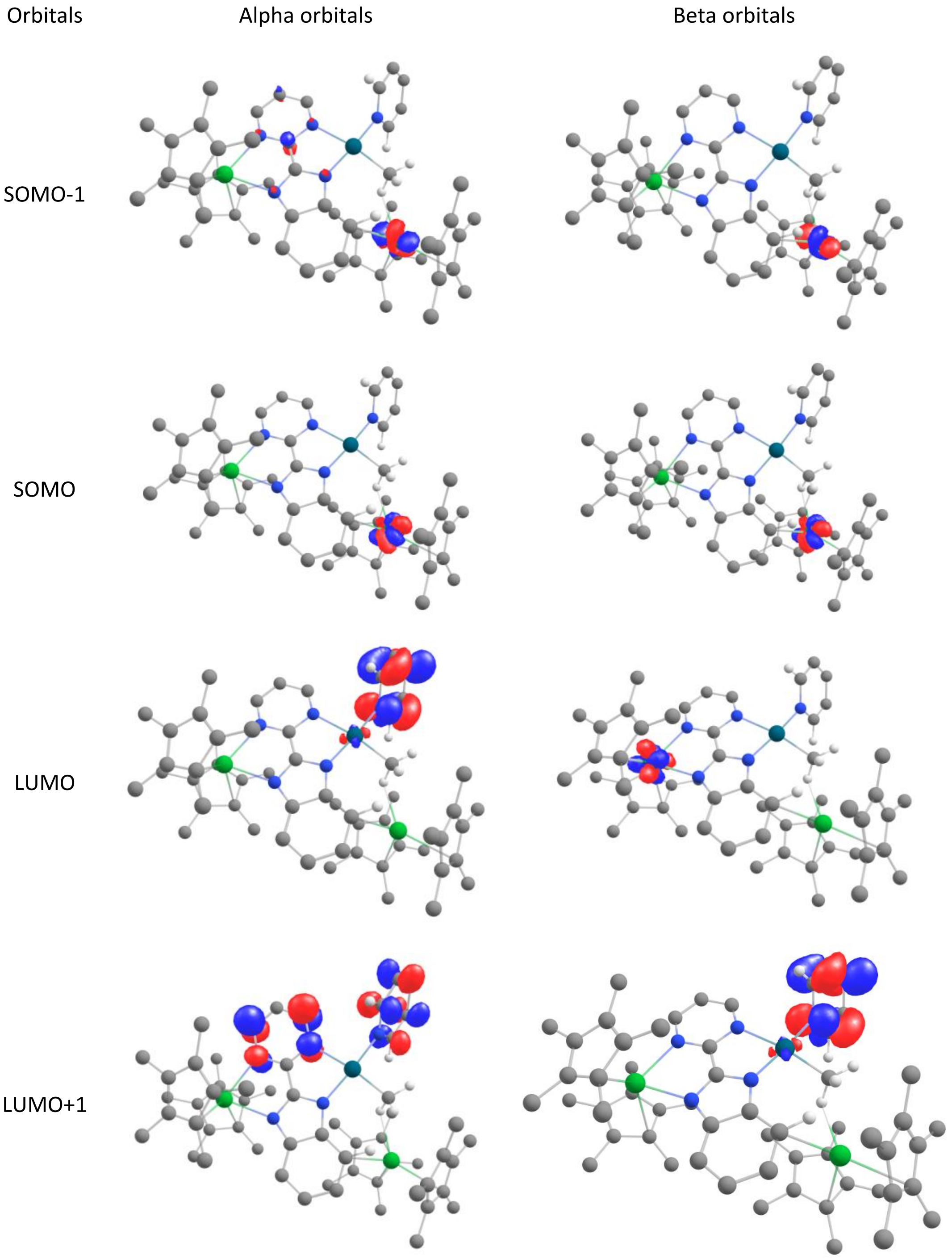

Figure S42. *Preview of main orbitals (isocontour value = 0.5) of **8**, following single point energy calculation at the DFT/PBE0-D3 level of theory.*

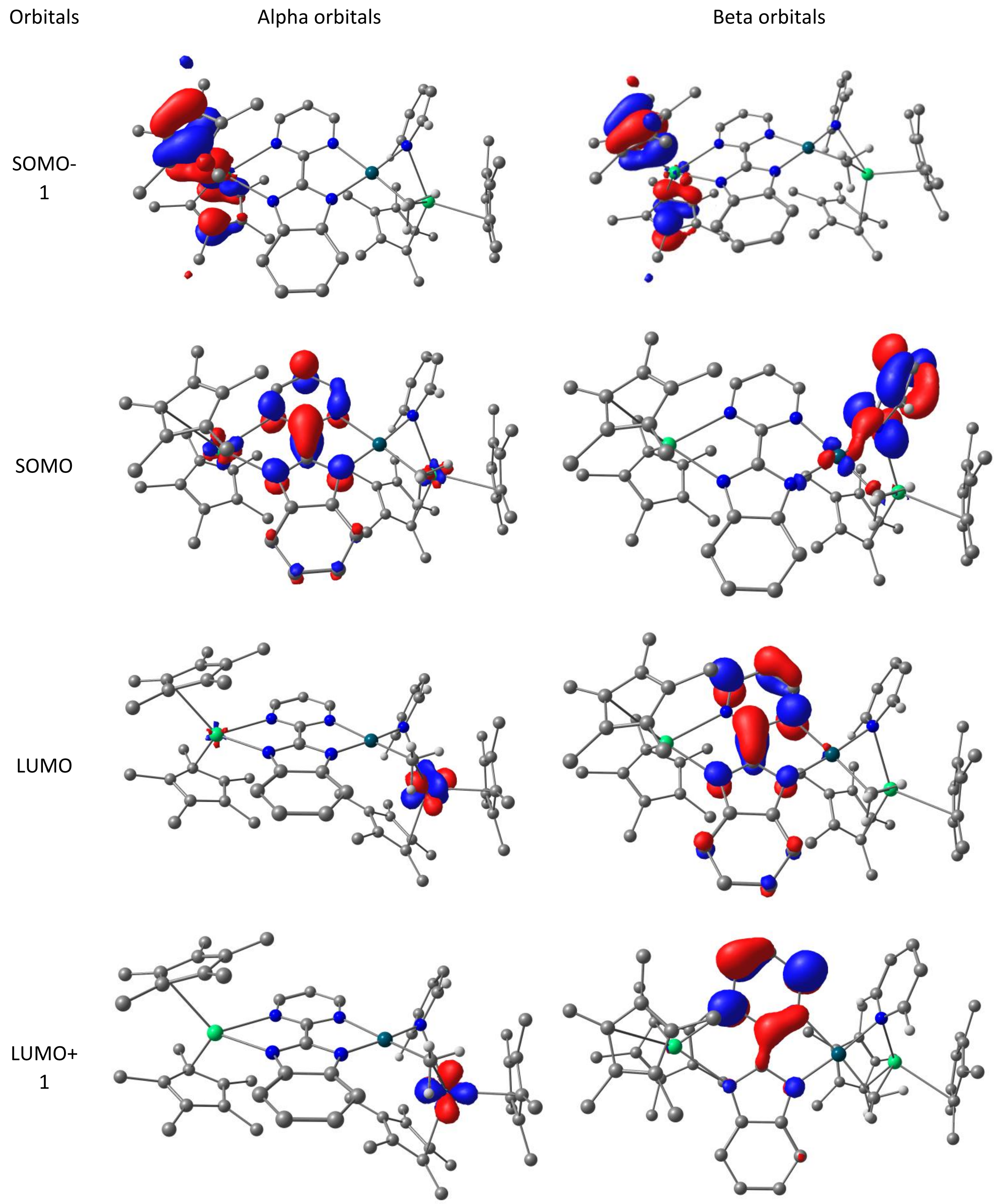

Figure S54. *Preview of main orbitals (isocontour value = 0.5) of* ***14****, following single point energy calculation at the DFT/PBE0-D3 level of theory.*

b) Species on the full C-H activation energy profile

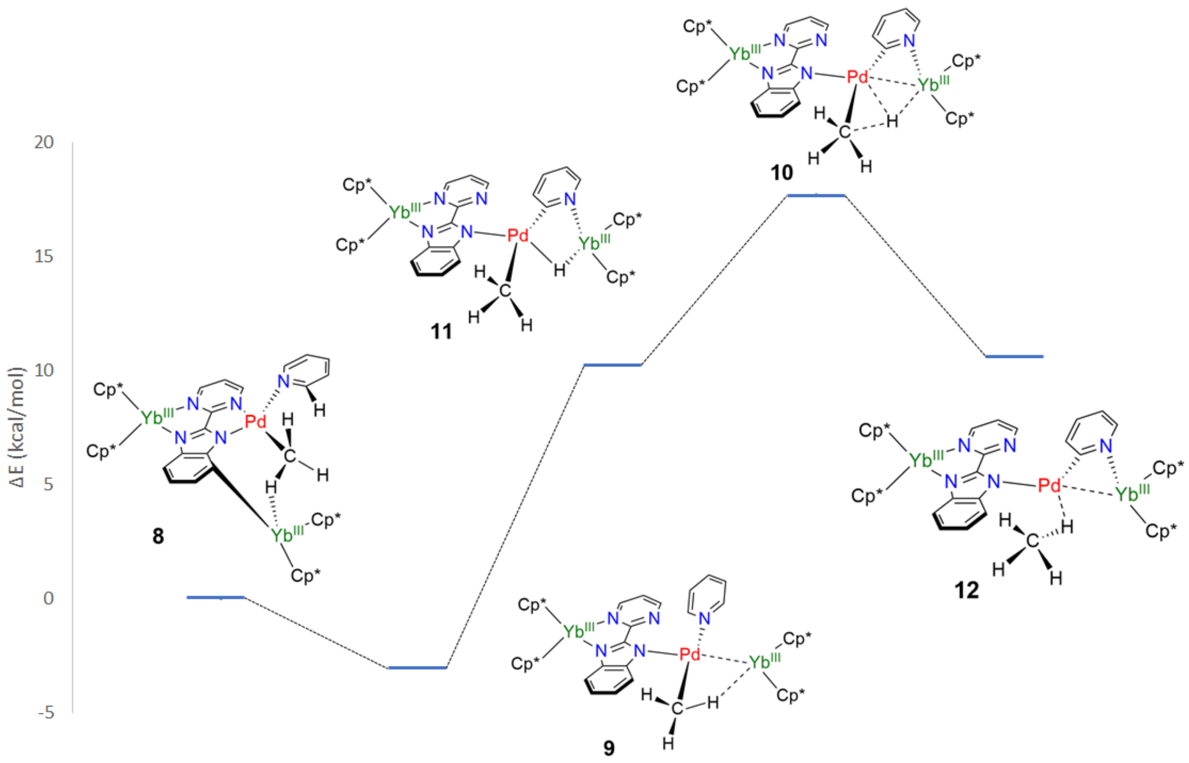

***Figure S43**: Energy profile of the C-H activation step.*

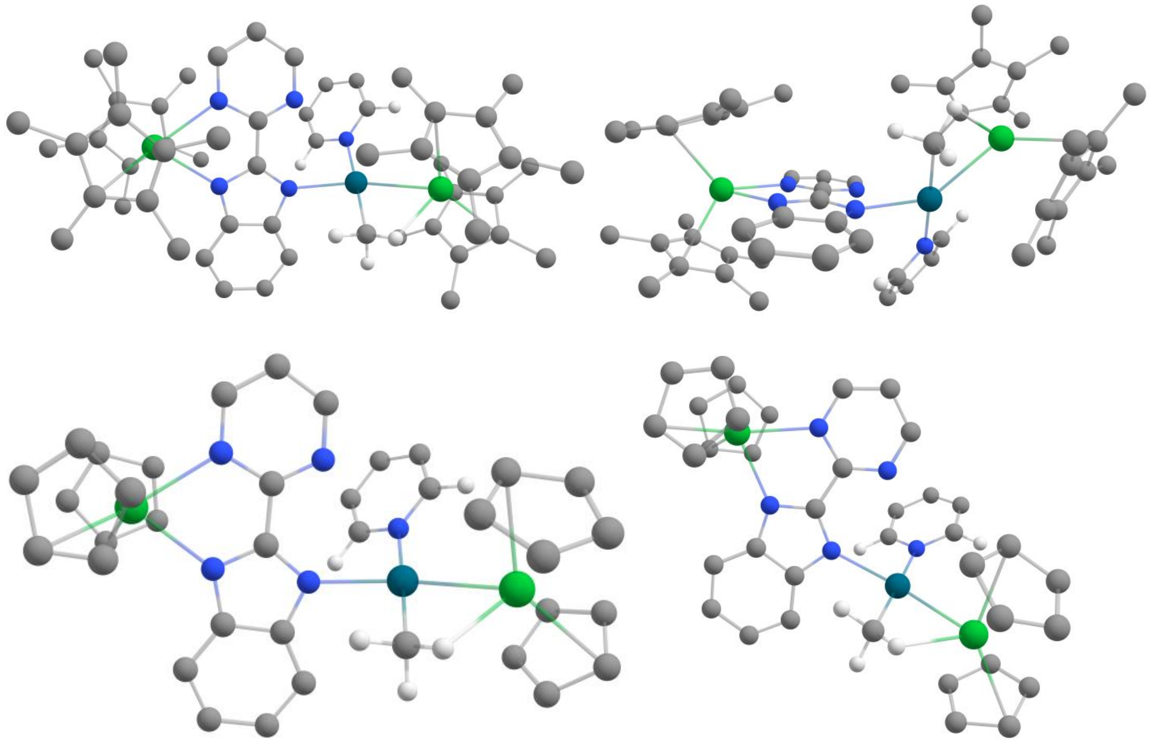

***Figure S44**: Structure of **9**. The majority of H atoms and the C_{Me} of the Cp* co-ligands are hidden in the interest of clarity.*

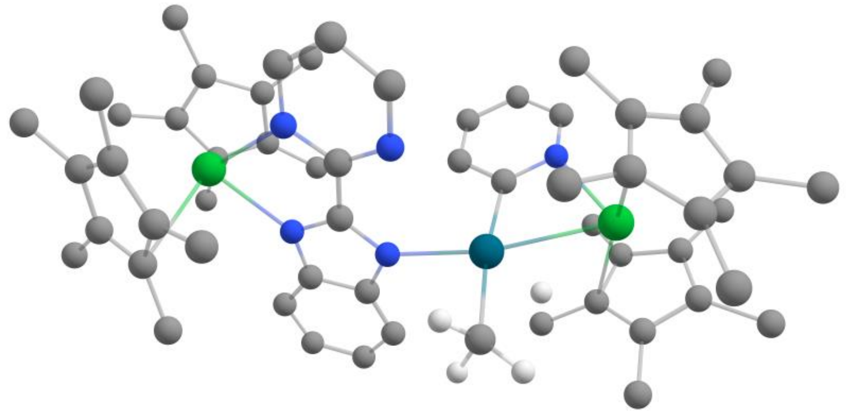

Figure S57: *Structure of the transition state,* ***10****. The majority of H atoms are hidden in the interest of clarity.*

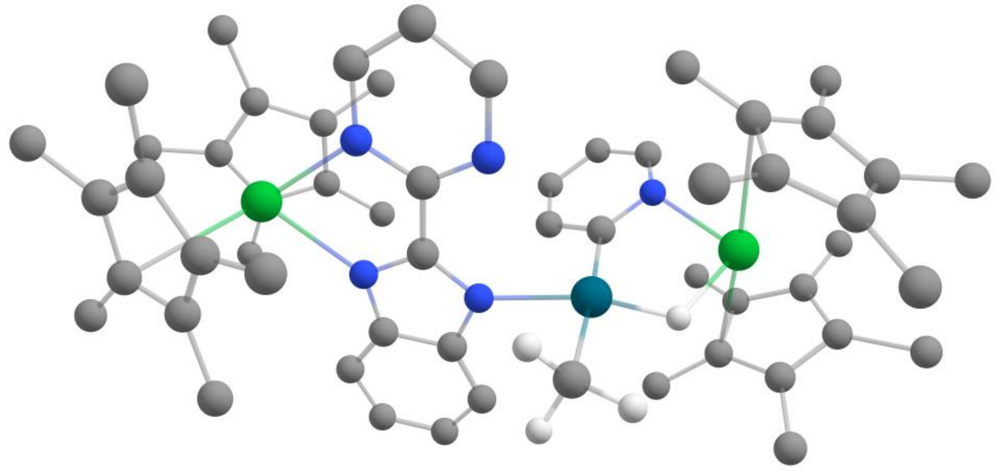

Figure S58: *Structure of* ***11****, obtained following an intrinsic reaction coordinate computation performed in the backward direction starting from the TS,* ***10****. The majority of H atoms are hidden in the interest of clarity.*

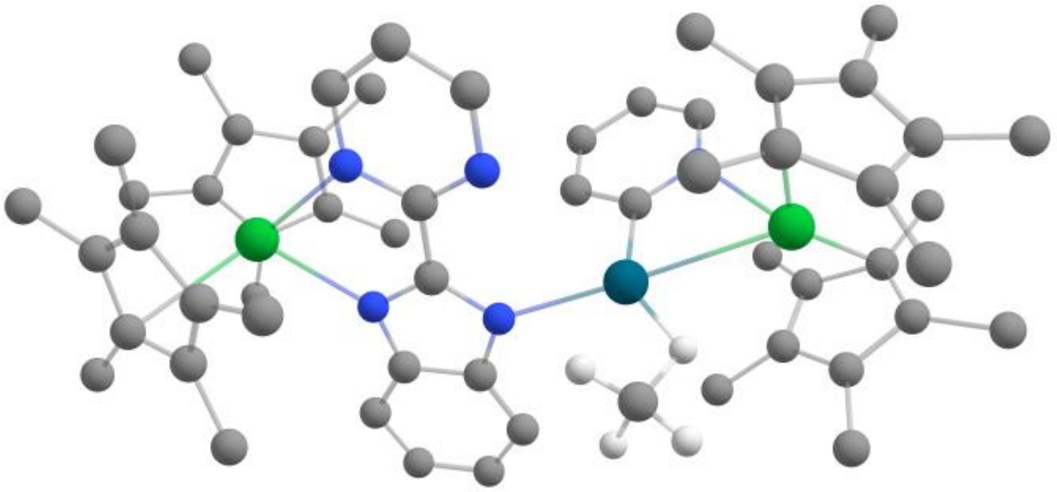

Figure S45: *Structure of* ***12****, obtained following an intrinsic reaction coordinate computation performed in the forward direction starting from the TS,* ***10****. The majority of H atoms are hidden in the interest of clarity. 12 was reoptimized with a fixed Pd-H_{α} distance of 1.891 Å.*

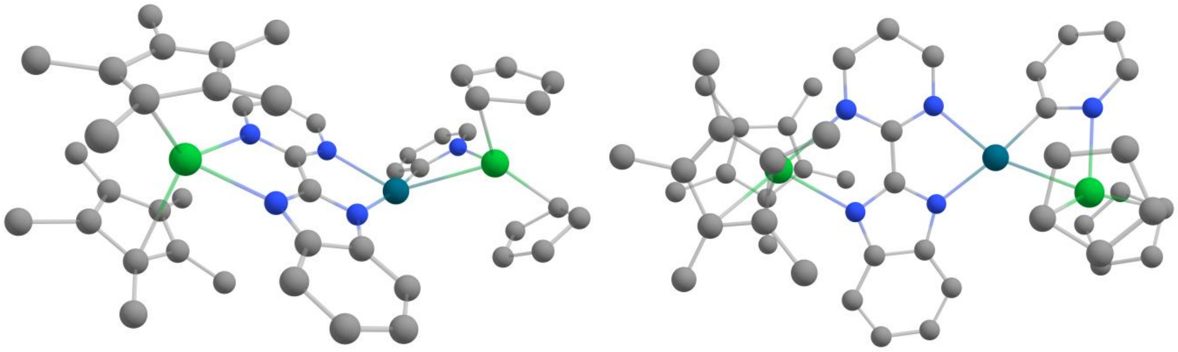

***Figure S60**: Structure of **13**, obtained following removal of the methane from **12**. The majority of H atoms and the C_{Me} of the Cp* co-ligands are hidden in the interest of clarity.*

Table S46. *Main distances (in Å) and angles (in °) in the heterotrimetallic complexes* ***8-13***.

	Av. Values					
**Atoms**	**8**	**9**	**10**	**11**	**12**	**13**
Pd-C_{Me}	2.036	2.106	2.192	2.089	2.668	
Pd-N_{py} (or C_{py})	2.037	2.139	2.106	2.158	2.003	
Pd-H_{α}	2.949	3.011	1.623	1.593	1.891	
Pd-N_{pym}	2.154	3.449	3.162	3.220	2.980	2.319
Pd-N_{imid}	2.027	2.254	2.270	2.134	2.246	2.142
Yb-Pd		2.817	2.838	2.991	2.952	2.867
Yb_1-N_{pym}	2.450	2.410	2.420	2.417	2.416	2.429
Yb_1-N_{imid}	2.398	2.344	2.332	2.337	2.359	2.379
Yb_1 – Cp*$_{ctr}$ (avg)	2.345	2.343	2.344	2.342	2.344	2.340
Yb_2 – Cp*$_{ctr}$ (avg)	2.350	2.332	2.371	2.368	2.342	2.351
Yb_2-ligand plane	~49°	~39°				~46°
Yb_2-H_{α}	2.672	3.679	2.436	2.117	3.341	
Yb_2-H_{1Me}	2.369	2.270				
Yb_2-H_{2Me}	2.692	3.238				
Yb_2-C_{Me}	2.936	2.659				

References

1 M. Haga, M. Ishizuya, T. Kanesugi, T. Yutaka, D. Sakiyama, J. Fees and W. Kaim, *Indian J. Chem Sect. A*, 2003, **42A**, 2290-2299.

2 Wim. De Graaf, Jaap. Boersma, W. J. J. Smeets, A. L. Spek and Gerard. Van Koten, *Organometallics*, 1989, **8**, 2907–2917.

3 D. J. Berg, C. J. Burns, R. A. Andersen and A. Zalkin, *Organometallics*, 1989, **8**, 1865–1870.

4 T. D. Tilley, J. M. Boncella, D. J. Berg, C. J. Burns, R. A. Andersen, G. A. Lawless, M. A. Edelman and M. F. Lappert, in *Inorganic Syntheses*, ed. A. P. Ginsberg, John Wiley & Sons, Inc., Hoboken, NJ, USA, 2007, pp. 146–150.

5 G. M. Sheldrick, *Acta Crystallogr. Sect. Found. Adv.*, 2015, **71**, 3–8.

6 G. M. Sheldrick, *Acta Crystallogr. A*, 2008, **64**, 112–122.

7 G. M. Sheldrick, *Acta Crystallogr. Sect. C Struct. Chem.*, 2015, **71**, 3–8.

8 O. V. Dolomanov, L. J. Bourhis, R. J. Gildea, J. A. K. Howard and H. Puschmann, *J. Appl. Crystallogr.*, 2009, **42**, 339–341.

9 C. F. Macrae, I. J. Bruno, J. A. Chisholm, P. R. Edgington, P. McCabe, E. Pidcock, L. Rodriguez-Monge, R. Taylor, J. van de Streek and P. A. Wood, *J. Appl. Crystallogr.*, 2008, **41**, 466–470.

10 F. Neese, *WIREs Comput. Mol. Sci.*, 2012, **2**, 73–78.

11 E. van Lenthe, E. J. Baerends and J. G. Snijders, *J. Chem. Phys.*, 1994, **101**, 9783–9792.

12 F. Weigend and R. Ahlrichs, *Phys. Chem. Chem. Phys.*, 2005, **7**, 3297.

13 F. Weigend, *Phys. Chem. Chem. Phys.*, 2006, **8**, 1057.

14 D. A. Pantazis and F. Neese, *J. Chem. Theory Comput.*, 2009, **5**, 2229–2238.

15 D. A. Pantazis, X.-Y. Chen, C. R. Landis and F. Neese, *J. Chem. Theory Comput.*, 2008, **4**, 908–919.

16 S. Grimme, S. Ehrlich and L. Goerigk, *J. Comput. Chem.*, 2011, **32**, 1456–1465.

17 S. Grimme, J. Antony, S. Ehrlich and H. Krieg, *J. Chem. Phys.*, 2010, **132**, 154104.

18 C. Adamo and V. Barone, *J. Chem. Phys.*, 1999, **110**, 6158–6170.

19 J. Tao, J. P. Perdew, V. N. Staroverov and G. E. Scuseria, *Phys. Rev. Lett.*, 2003, **91**, 146401.

20 O. A. Vydrov and G. E. Scuseria, *J. Chem. Phys.*, 2006, **125**, 234109.

21 J.-D. Chai and M. Head-Gordon, *J. Chem. Phys.*, 2008, **128**, 084106.

22 F. Aquilante, J. Autschbach, R. K. Carlson, L. F. Chibotaru, M. G. Delcey, L. De Vico, I. Fdez. Galván, N. Ferré, L. M. Frutos, L. Gagliardi, M. Garavelli, A. Giussani, C. E. Hoyer, G. Li Manni, H. Lischka, D. Ma, P. Å. Malmqvist, T. Müller, A. Nenov, M. Olivucci, T. B. Pedersen, D. Peng, F. Plasser, B. Pritchard, M. Reiher, I. Rivalta, I. Schapiro, J. Segarra-Martí, M. Stenrup, D. G. Truhlar, L. Ungur, A. Valentini, S. Vancoillie, V. Veryazov, V. P. Vysotskiy, O. Weingart, F. Zapata and R. Lindh, *J. Comput. Chem.*, 2016, **37**, 506–541.

23 V. Veryazov, P.-O. Widmark, L. Serrano-Andrés, R. Lindh and B. O. Roos, *Int. J. Quantum Chem.*, 2004, **100**, 626–635.

24 B. O. Roos, R. Lindh, P.-Å. Malmqvist, V. Veryazov, P.-O. Widmark and A. C. Borin, *J. Phys. Chem. A*, 2008, **112**, 11431–11435.

25 F. Aquilante, T. B. Pedersen, R. Lindh, B. O. Roos, A. Sánchez de Merás and H. Koch, *J. Chem. Phys.*, 2008, **129**, 024113.

26 P. L. A. Popelier, in *The Chemical Bond*, eds. G. Frenking and S. Shaik, Wiley-VCH Verlag GmbH & Co. KGaA, Weinheim, Germany, 2014, pp. 271–308.

27 P. L. A. Popelier, J. Burke and N. O. J. Malcolm, *Int. J. Quantum Chem.*, 2003, **92**, 326–336.

28 T. Lu and F. Chen, *J. Comput. Chem.*, 2012, **33**, 580–592.
29 P. de Silva and C. Corminboeuf, *J. Chem. Theory Comput.*, 2014, **10**, 3745–3756.
30 R. F. W. Bader, *J. Phys. Chem. A*, 1998, **102**, 7314–7323.
31 R. F. W. Bader, *Acc. Chem. Res.*, 1985, **18**, 9–15.
32 R. Bianchi, G. Gervasio and D. Marabello, *Inorg. Chem.*, 2000, **39**, 2360–2366.
33 A. D. Becke and K. E. Edgecombe, *J. Chem. Phys.*, 1990, **92**, 5397–5403.
34 G. te Velde, F. M. Bickelhaupt, E. J. Baerends, C. Fonseca Guerra, S. J. A. van Gisbergen, J. G. Snijders and T. Ziegler, *J. Comput. Chem.*, 2001, **22**, 931–967.
35 B. M. Gardner, D. Patel, A. D. Cornish, J. McMaster, W. Lewis, A. J. Blake, S. T. Liddle, *Chem. - Eur. J.* 2011, **17**, 11266–11273.
36 X. Fradera, M. A. Austen, R. F. W. Bader, *J. Phys. Chem. A* 1999, **103**, 304–314.
